# Analysis of Virus-Derived siRNAs in Strawberry Plants Co-Infected with Multiple Viruses and Their Genotypes

**DOI:** 10.3390/plants12132564

**Published:** 2023-07-06

**Authors:** Igor Koloniuk, Alena Matyášová, Sára Brázdová, Jana Veselá, Jaroslava Přibylová, Eva Várallyay, Jana Fránová

**Affiliations:** 1 Institute of Plant Molecular Biology, Department of Plant Virology, Biology Centre CAS, 370 05 Ceske Budejovice, Czech Republic; matyasova@umbr.cas.cz (A.M.); sara.souckova@umbr.cas.cz (S.B.); jana.vesela@umbr.cas.cz (J.V.); pribyl@umbr.cas.cz (J.P.); jana@umbr.cas.cz (J.F.); 2 Faculty of Agriculture, University of South Bohemia, 370 05 Ceske Budejovice, Czech Republic; 3 Genomics Research Group, Institute of Plant Protection, Department of Plant Pathology, Hungarian University of Agriculture and Life Sciences, Szent-Gyorgyi Albert Street 4, 2100 Gödöllő, Hungary; varallyay.eva@uni-mate.hu

**Keywords:** strawberry virus, vsiRNA, RNA silencing, multistrain virus infection

## Abstract

Plants can be infected with multiple viruses. High-throughput sequencing tools have enabled numerous discoveries of multi-strain infections, when more than one viral strain or divergent genomic variant infects a single plant. Here, we investigated small interfering RNAs (siRNAs) in a single strawberry plant co-infected with several strains of strawberry mottle virus (SMoV), strawberry crinkle virus (SCV) and strawberry virus 1 (StrV-1). A range of plants infected with subsets of the initial viral species and strains that were obtained by aphid-mediated transmission were also evaluated. Using high-throughput sequencing, we characterized the small RNA fractions associated with different genotypes of these three viruses and determined small RNA hotspot regions in viral genomes. A comparison of virus-specific siRNA (vsiRNA) abundance with relative viral concentrations did not reveal any consistent agreement. Strawberry mottle virus strains exhibiting considerable variations in concentrations were found to be associated with comparable quantities of vsiRNAs. Additionally, by estimating the specificity of siRNAs to different viral strains, we observed that a substantial pool of vsiRNAs could target all SMoV strains, while strain-specific vsiRNAs predominantly targeted rhabdoviruses, SCV and StrV-1. This highlights the intricate nature and potential interference of the antiviral response within a single infected plant when multiple viruses are present.

## 1. Introduction

RNA silencing is a conserved mechanism invoked not only to counteract invading viral and viroid species, but also to regulate gene expression [1]. Double-stranded RNA (dsRNA) synthesized during viral replication, as well as highly structured single-stranded RNA of the viral genome can trigger RNA silencing pathways [1,2,3]. Host DICER-like enzymes cleave triggering viral RNA templates into 21–24 nucleotide-long virus-derived small interfering RNA (vsiRNA) products [4]. As a component of RNA-induced silencing complexes, after vsiRNAs are loaded, they are used as blueprints for sequence recognition, ensuring precise degradation and/or translational inhibition of the targeted RNAs [1,4]. Furthermore, vsiRNAs bind to target RNAs during viral infection and direct RNA-dependent RNA polymerases to synthesize new dsRNA products; this in turn produces transitive or secondary vsiRNAs and amplifies silencing potency in plants [5,6].

Analyzing the vsiRNA pattern of virus-infected plants has shown that specific genomic regions give rise to greater numbers of vsiRNAs, termed RNA silencing ‘hotspots’ [7].

In practice, the characteristics of virus-specific siRNAs can be used for developing induced virus resistance [7,8,9], with hotspot regions being first choice of template for the artificial production of vsiRNAs against selected pathogens [10].

The use of high-throughput sequencing has revealed that mixed viral infections are common in nature [11,12,13,14,15]. Moreover, the abundance of sequencing reads produced to date allows for the detection of not only virus species, but also their sequence variants and strains replicating in a single host, presenting as viral clouds [16,17,18]. Such co-existence may impact various aspects of viral infection and evolution. The presence of diverse populations of viral mutants provides a favorable reservoir for the emergence of new viral strains [19,20]. The existence of multiple viral strains in a viral cloud can also impact the transmission dynamics of an infection [16,18,21]. Some strains may be more transmissible than others, leading to differences in the rate of spread within the population [22,23]. Nevertheless, knowledge about the plant RNA silencing response to such viral infections is scarce. The majority of research data on viral strains infecting a single host come from the citrus tristeza virus (*Closteroviridae*) and potato virus Y (*Potyviridae*) [24,25,26,27]. The phenomenon in which a single host is sequentially infected by several distinct isolates of the same viral species is called ‘superinfection’ [25,28], but not all viral strains are capable of superinfection, which practically protects plants infected with mild viral strains from more aggressive ones, an approach called cross-protection or superinfection exclusion [28,29].

The mechanisms underlying cross-protection are not fully understood. Remarkably, while some studies suggest that RNA silencing is not involved in the process [25,30], others do not exclude its contribution [31,32,33]. However, a mechanistic model has been proposed that explains superinfection exclusion as a side effect of inhibited replication of progeny viruses in the cells of their ‘parents’ [28].

Over thirty different viruses are able to infect strawberry [34,35,36,37,38]. Although a single infection by a given species does not usually lead to symptom development [39,40], the combination of these viruses in mixed infections has been recognized to reduce yield and are thus economically important [36,37]. In our previous studies, we noted a moderately high incidence of mixed virus infections in strawberry under field conditions [41,42]. In an interesting case, a single strawberry plant was infected with multiple strains of three different viruses: the strawberry mottle virus (SMoV—*Secoviridae*), strawberry crinkle virus (SCV—*Rhabdoviridae*) and strawberry virus 1 (StrV-1—*Rhabdoviridae*) [41,43,44]. All three viruses are aphid-borne viruses, and preferences in the transmission of distinct viral strains by certain aphid species have been reported [44]. SMoV is highly variable, and several variants of different geographical origins have been sequenced thus far [45,46,47]; however, an absence of recombination interactions between the strains of the virus has been demonstrated [44]. In the case of the rhabdoviruses, we may expect the existing diversity of these viruses to be high, although the number of studied samples has been rather small. We previously showed that selected strains of SMoV and StrV-1 are capable of superinfection [44], and their transmission via insect vectors has been estimated to be the most important factor in viral spreading in strawberry under field conditions [37,48].

In the current study, we focused on the vsiRNA analysis of strawberry plants infected with several viruses in different combinations, characterized the specificity of vsiRNAs against individual viral strains, and evaluated whether the relative viral concentrations correspond to the levels of vsiRNA.

## 2. Results and Discussion

### 2.1. Analysis of vsiRNA Associated with the Original Complex of Viral Strains

**Figure 1:**
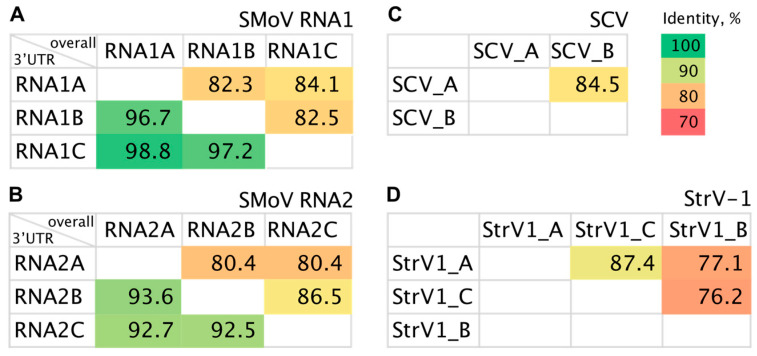
Pairwise intraspecies nucleotide identities of the strawberry mottle virus (SMoV) (**A**,**B**), strawberry crinkle virus (SCV) (**C**) and strawberry virus 1 (StrV-1) (**D**) strains. For SMoV segments, a comparison of 3′ untranslated regions (3′ UTRs) is given in the lower triangular part. Color scales correspond to percent nucleotide identity.

The original *Fragaria ananassa* cv Cacanska rana CRM plant (CRM is an acronym for Cacanska rana mother) was infected with a mixture of three viral species: SMoV, SCV and StrV-1. In total, eleven distinct viral RNAs were detected: three for each genomic component of SMoV, two strains of SCV and three strains of StrV-1 [41,43]. The intraspecies nucleotide identities of these strains range from 77% to 87% (Figure 1), and most of the observed differences were synonymous, i.e., not involving amino acid changes.

Stolons from the original plant were used for vegetative propagation. Three randomly selected daughter plants were used for small RNA sequencing library preparation. From each plant, three to four libraries were prepared from different leaves, resulting in eleven datasets (1B—four datasets (1B_1—1B_4), 2A—three datasets (2A_2—2A_4), and 3K—four datasets (3K_1—3K_4)). The samples were verified in parallel with RT-qPCR (Table S1, detailed view). Additionally, aphid-mediated transmission was used to produce strawberry plants infected with only a subset of viruses. From these plants, eleven additional small RNA sequencing libraries were prepared to evaluate plant vsiRNA specificity against individual viral strains (Table S1, datasets). The obtained data were deposited at the NCBI repository under accession number SAMN34995356-77.

Among 10 million randomly selected trimmed reads, 29,832–69,742 could be mapped to the genome of any of the infecting viral strains, with virus-specific reads comprising less than 1% of the total small RNA population (Table S1). Of these viral reads, 82–89% were found to be strain-specific and mapped to only one of the presenting viral genomes.

Certain reads were not unique and mapped to more than one strain. The exclusion of such reads resulted in a smaller set of strain-specific reads (Table 1). This difference was the largest in the case of SMoV, which has long 3′ untranslated regions (3′ UTRs) that are moderately conserved not only between its two genomic segments, but also between the different strains included in the current study (Figure 1A,B, lower triangular parts). These regions showed a notably higher vsiRNA coverage than the remaining genomic parts. To some extent, this might be caused by the presence of secondary structures, which are known to play an important role in siRNA genesis [49] and have an indispensable role in the viral replication of RNA viruses by serving as 3′ cap-independent translation enhancers [50,51].

### 2.2. Comparison of Viral Concentrations and vsiRNA Abundances

**Figure 2:**
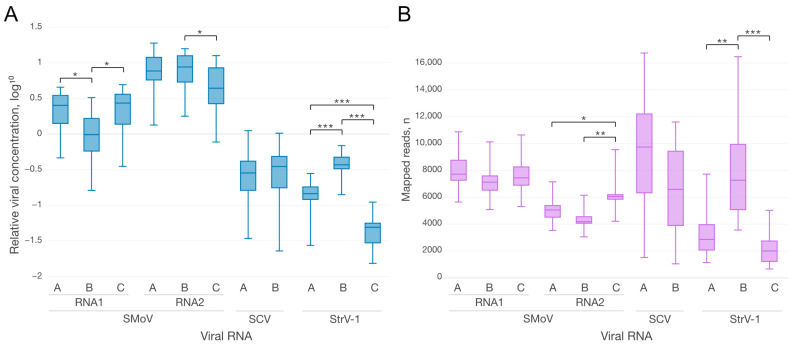
Boxplots of relative viral concentrations ((**A**), log 10 scale) and mapped reads (**B**) calculated for eleven viral references (axis of abscissas) from three CRM daughter plants. Only statistically significant differences between strains of the same species are denoted by square brackets with asterisks (* *p* ≤ 0.05, ** *p* ≤ 0.01, *** *p* ≤ 0.001). Relative viral concentration data are listed in Table S2.

Using biological replicates of CRM plants, significant differences were established between the relative RNA concentrations of some, but not all of the strains of the studied viruses (Figure 2A). At the same time, the differences in the numbers of vsiRNA (Figure 2B) did not fully correspond to the differences in viral concentrations.

For SMoV RNAs, significant differences in both the concentrations and plant vsiRNA quantities were observed only between viral RNA1^A^ and RNA1^B^. The relative concentrations of RNA2 segments were higher than those of RNA1s segments, but the corresponding small RNAs showed the opposite trend: the number of vsiRNA reads generated from RNA1 variants was higher than that generated from RNA2 variants. This opposite correlation can be explained by the fact that vsiRNAs are not only produced from, but also target viral RNAs. Hence, their increased level might result in a greater abundance of SMoV-specific loaded Argonaute proteins, which target and cleave the corresponding viral RNAs, resulting in a decrease in their concentrations.

Both strains of SCV had comparable viral concentrations and numbers of strain-specific vsiRNAs. In contrast, for the other rhabdovirus, StrV-1, significant differences in the concentrations and vsiRNAs between all three strains were observed, with the only exception being nonsignificant changes in the levels of vsiRNAs between strains A and C.

**Figure 3:**
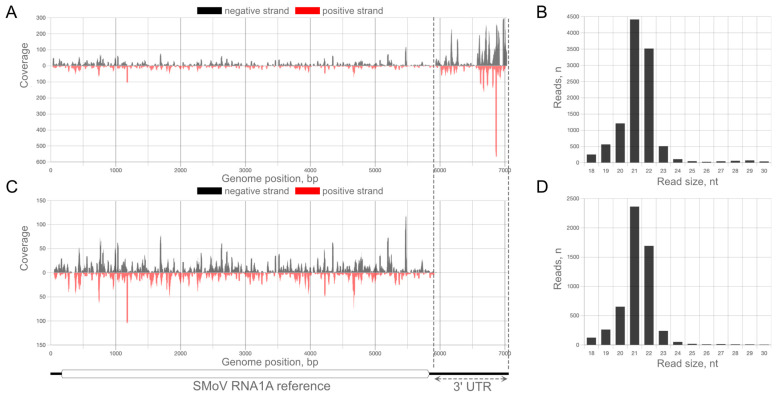
Distribution of viral small interfering RNAs (vsiRNAs) from the 1B_1 sample of the SMoV RNA1A reference, (**A**) with their size profiling (**B**) and distribution of the exclusive RNA1A strain-specific vsiRNA fraction (**C**) and their size profiling (**D**). Note the absence of reads targeting the 3′ untranslated region in panel (**C**). Color legends show the strand specificity of the mapped reads. The axes of abscissas show a genomic position in nucleotides, and axes of ordinary the sequence (mapping) coverage. The genomic organization of the reference is shown at the bottom side of the figure. The boxed rectangle denotes the open reading frame. Distribution plots for all samples are shown in Figure S1.

The numbers of mapped reads for SCV and StrV-1 did not differ much when comparing total and strain-specific mapped reads. However, due to the long conserved 3′ UTRs, the difference for SMoV was twofold on average (Table 1 and Table S1). This was clear based on the comparison of plots for all mapped (Figure 3A), as well as only strain-specific (Figure 3C) reads.

**Figure 4:**
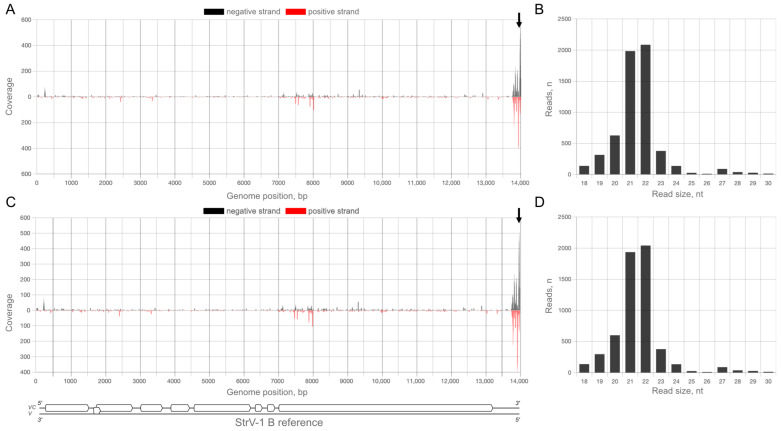
Coverage plots of the reads from the 1B_1 sample mapped to the StrV-1B reference (the genome complimentary strand is shown), (**A**) with their size profiling (**B**) and distribution of the exclusive StrV-1B strain-specific fraction (**C**) with their size profiling (**D**). Note the hotspot regions in the 5′ untranslated region (an arrow annotation). Color legends show the strand specificity of the mapped reads. The axes of abscissas show genomic position in nucleotides, and axes of ordinary the sequence (mapping) coverage. The genomic organization of the reference is shown at the bottom side of the figure. Boxed rectangles denote open reading frames. Distribution plots for all samples are shown in Figures S1 and S3.

Another irregularity was observed in the StrV-1 and SCV mappings. The StrV-1B strain shows a hotspot region at the 5′ end of the genomic RNA (Figure 4) that is absent in the A and C strains, but invariably present in all samples (Figures S1 and S3).

A closer inspection of the peak showed that it predominantly contains 27 nt long reads mapped to the terminus. In an RNA silencing study of tomato yellow leaf curl Sardinia virus, a begomovirus with a DNA genome, Miozzi et al. identified host-derived siRNAs that cross-react with the major virus hotspot [52], and these siRNAs have been suggested to originate from integrated ancient remains of geminiviral-related DNA that is part of the RNA silencing machinery [52,53]. To determine whether this might explain our results, a BLASTn search against a Fragaria GenBank database (taxid:3746) was performed but did not return a 100% match, indicating a non-Fragaria origin of this sequence (searched on 12 November 2022). For StrV-1, further analyses showed that in addition to the plants with multiple infections, some StrV-1-negative samples (see Section 2) also contained these 27 nt long reads, yet in much lower quantities (Figures S1 and S3). It may be speculated that such reads were assigned to the sample due to index hopping or read misidentification [54], as library construction was performed with a single index [54,55]. Nevertheless, to obtain a clear explanation, further investigation is needed.

**Figure 5:**
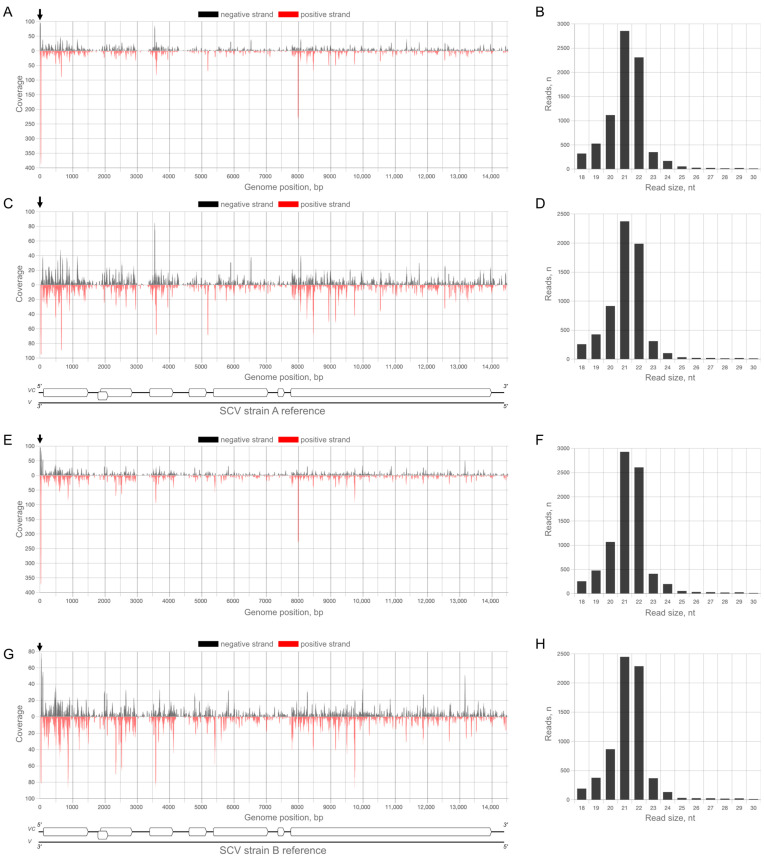
Coverage plots of reads from the 1B_1 sample mapped to the SCV references (genome complimentary strands are shown) (**A**,**E**) and the strain-specific (exclusive) fraction (**C**,**G**). Corresponding size profiles of vsiRNAs are shown in panels (**B**,**D**,**F**,**H**). Note the hotspot region in the 3′ untranslated region (black arrows). Color legends show the strand specificity of the mapped reads. Axes of abscissas show genomic position in nucleotides, axes of ordinary—sequence (mapping) coverage. The genomic organization of the references is shown at the bottom side of the figure. Boxed rectangles denote open reading frames. Distribution plots for all samples are shown in Figure S1.

Unlike StrV-1, SCV exhibits hotspot vsiRNA regions near the 3′ end of the genomic RNA, as detected for both strains (Figure 5) across all samples (Figure S1).

This region is not conserved between A and B strains of SCV, meaning that the mapped reads are unique to both strains. Furthermore, there are no mapped reads in the SCV-negative samples.

### 2.3. The Abundance of vsiRNAs against Individual Strains of SMoV Does Not Correspond to Their Relative Concentration

**Figure 6:**
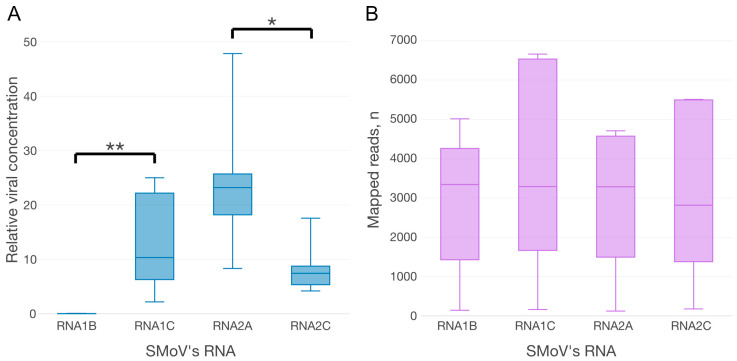
(**A**) Boxplot of estimated relative viral concentrations of SMoV RNAs for four endogenous plant references. The Wilcoxon rank sum exact test indicated a significant difference between the levels of RNA1^B^ and RNA1^C^ (**—*p* = 0.007), and RNA2^A^ and RNA2^C^ (*—*p* = 0.032). The plot data and Cq values are shown in Table S2. (**B**) Boxplot of mapped reads for SMoV RNAs.

During our previous study on aphid- and petiole-wedge grafting-mediated transmission of SMoV, SCV and StrV-1, we obtained a set of *Fragaria vesca* plants infected with either individual viruses and strains or their various combinations. During identification of viral strain composition after aphid-mediated transmission, we identified two plants infected with a combination of viral SMoV strains: RNA1^BC^ and RNA2^AC^, and found that RNA1^B^ showed a significantly lower concentration than RNA1^C^ (Figure 6A). Although we observed differences between different SMoV RNAs during analyses of other samples, in this case, the relative concentration of RNA1^B^ was on average 500-fold lower (Table S2, Figure 6A).

Therefore, we hypothesized that the RNA1^B^- and RNA1^C^-specific vsiRNAs should also quantitatively vary. However, there was no significant difference in the numbers of mapped reads (Figure 6B) between RNA1^B^ (M = 2835, SD = 2016) and RNA1^C^ (M = 3661, SD = 2898); t (7.1) = −0.52, *p* = 0.62. Correspondingly, no significant difference was observed for RNA2^A^ (M = 2836, SD = 1993) and RNA2^C^ (M = 3074, SD = 2404); t (7.7) = 0.17, *p* = 0.89. Thus, future studies of SMoV strain variability should further examine these findings. Taking into account the considerable difference in the titers of RNA1^B^ and RNA1^C^, it would be problematic to detect RNA1^B^ without prior knowledge of its existence. Nevertheless, as the actual variability of SMoV strains was uncovered only a few years ago, reliable tools for the identification of the full spectrum of SMoV strains may still be lacking. The existing SMoV detection system relies on the highly conserved stretch within the 3′UTR of both RNA1 and RNA2, which is a superb region from a high conservation perspective, but a poor choice for strain discrimination.

### 2.4. Analysis of vsiRNA against StrV-1 Strains in StrV-1-Infected Strawberry

Considering the observed 27 nt hotspot in the StrV-1_B strain, we analyzed the small RNA population in plants infected with either StrV-1_A or StrV-1_B, or a combination thereof (Figure S3). On average, more than 90% of the mapped reads in this study were recognized as either A- or B-strain-specific. Interestingly, although 21 nt and 22 nt vsiRNAs were dominant, all three datasets from single StrV-1_B infections showed a considerable 27 nt fraction among the mapped reads. After a close examination, it was revealed to consist of a single sequence 5′-ATTGATCGTATAGATGTTATCATCCGT-3′ aligned against the StrV-1 genomic strand at its 5′ end (positions 2–28). An analysis of potential secondary structures of the sequence showed only a weak structure with a four-base-long loop (underlined). The genesis of vsiRNA involves the production of mainly 21 nt and 22 nt long products, and the presence of 24 nt vsiRNAs has been reported only for some DNA and RNA viruses [56,57]. During the peer-reviewing of the current manuscript, one of the reviewers pointed out that this RNA might be a defective viral RNA. Defective interfering RNAs (DI RNAs) are produced during several passage of viral infection [58]; hence, it would be interesting to further investigate the viral derived RNA population for the presence of such DI elements. Moreover, siRNAs produced from DIs can saturate the siRNA binding capacity of viral VSRs, leading to the attenuation of viral symptoms [59], which would further alter the complex picture under co-infection. However, HTS in combination with validation via Sanger sequencing would be an appropriate tool for DI identification, as the building of chimeric contigs could generate false DI annotations. To determine if the detected 27 nt long small RNAs are or could act as DI RNAs is an interesting question that could be addressed in the future. Therefore, assuming that the 27 nt small RNAs are not sequencing artifacts, they might be produced in other pathways or could be an intermediate product of vsiRNA processing.

Furthermore, comparing the number of vsiRNAs per virus in the different samples showed that there were 14,000 mapped reads on average in a single StrV-1 infection, while the corresponding average when other viruses were present was much lower, at 3500 (Table S1). SMoV encodes two weak viral suppressors of RNA silencing, which were able to alter the potato virus X concentration in a co-expression study [60]. Thus, it is possible that SMoV coinfection with rhabdoviruses influences the RNA silencing reaction against them through this mechanism.

## 3. Summary and Conclusions

Our data show that plants infected with more than one viral strain produces strain-specific vsiRNAs against each viral RNA. For SCV, the vsiRNA hotspot was found at the 3′ terminus of the genomic RNA, whereas no universal hotspot was found for StrV-1. Furthermore, although both the SCV and StrV-1 vsiRNAs were quite specific, a large fraction of SMoV vsiRNAs matched more than one strain due to its long conserved 3′UTR regions. It seems that in the development of a plant protection method based on exogenous siRNAs against SMoV, 3′ UTRs might be a good target. However, there was no consistent agreement between the relative levels of viral RNA and the corresponding levels of vsiRNAs. Despite the very low abundance of SMoV RNA1B in Fv-SMoV_BC_AC-1 and -2 plants, the levels of RNA1B-specific vsiRNAs were not significantly lower than those of vsiRNAs targeting the other strain or other viruses. When loaded into the RISC, vsiRNAs in multiple infections can target related viral strains, which is why it is very difficult to find a direct correlation between the concentration of different viral strains and the number of vsiRNAs. The presence of viral silencing suppressors capable of interfering with antiviral silencing in different steps of RNAi can further alter this complex pattern, which should be investigated in future studies.

## 4. Materials and Methods

### 4.1. Plant Materials

The CRM3 isolate of *F. ananassa*, cultivar Cacanska rana, served as a plant source for stolon-mediated propagation (Table 2, plants 1B, 2A, 3K). The plant was previously reported to be infected with three viruses: SMoV, SCV and StrV-1. Each virus was represented by several strains that were arbitrarily named A, B and C, if applicable: SMoV—SMoV RNA1^A^, RNA1^B^, RNA1^C^, RNA2^A^, RNA2^B^, RNA2^C^; SCV—SCV-A, SCV-B; StrV-1—StrV-1 A, StrV-1 B, StrV-1 C. Note that there is no established correlation between names of RNA1s and RNA2s of SMoV.

The CRM daughter plants served as virus sources during the aphid-mediated transmission of SMoV and StrV-1 to Alpine strawberry, *F. vesca semperflorens*; for details, see Koloniuk et al. [44]. Briefly, *F. vesca* plants infected with some of the abovementioned strains of SMoV and StrV-1 were obtained (Table 2). The mixed infection of StrV-1-A and StrV-1-B strains was obtained using the consequent grafting-mediated infection [44].

The plants were maintained in an insect-proof greenhouse with a controlled temperature, under a 16 h light/8 h dark photoperiod.

### 4.2. Total and Small RNA Isolation

Combined total plant RNA and small RNA extractions were performed using a single leaf that was snap-frozen in liquid nitrogen, homogenized, divided into two approximately 50 mg aliquots and processed with a Thermo Scientific GeneJET Plant RNA Purification Mini Kit (Thermo Scientific, Waltham, MA, USA) and mirPremier microRNA Isolation Kit (Sigma-Aldrich, St. Louis, MI, USA), respectively, following manufacturers’ recommendations. The quantification and quality control of the RNA extracts were performed using a Nanodrop 1000 UV-Vis spectrophotometer and Qubit HS RNA and IQ assays (Invitrogen, Carlsbad, CA, USA).

### 4.3. cDNA Synthesis and Two-Step Reverse-Transcription qPCR (RT-qPCR)

Total RNA (200 ng—400 ng of the total plant RNA) was reverse-transcribed to cDNA using the Maxima H Minus First Strand cDNA Synthesis Kit with dsDNase in 20 µL reactions following the manufacturers’ recommendations. The cDNA was then diluted to a ratio of 1:10 with Milli-Q-grade water and subjected to qPCR assays.

The RT-qPCR assays were conducted using a CFX96 real-time PCR detection system (Bio-Rad, Hercules, CA, USA). The 10 µL reaction was prepared from 5 µL of tenfold-diluted cDNA, 0.25 µL forward and reverse primers (10 mM, final concentration 250 nM), 2.75 μL of nuclease-free water and 2 µL of 5x HOT FIREPol EvaGreen qPCR Mix Plus (Solis BioDyne, Taru, Estonia). The reaction conditions were 95 °C for 12 min, followed by 40 cycles of 95 °C for 10 s, 60 °C for 20 s and 72 °C for 20 s. The dissociation curve analysis was performed by ramping from 65 °C to 95 °C (with increments of 0.5 °C for 5 s) to verify the specificity of primer amplification and the presence of potential primer dimers based on a single peak. No template, positive, and if necessary, no reverse transcriptase controls were included to check for potential cross-contamination and the presence of genomic DNA. The amplification efficiency (E, Table S3) was assessed using a standard curve based on serial dilutions of the cDNA template. Each reaction was carried out in triplicate.

Relative viral concentrations were calculated using the formula efficiency ^ (Cq ^ref^ − Cq ^virus^), where efficiency is the experimentally calculated efficiency of the corresponding primers (Table S3), Cq^ref^ is the geometric mean of the Cq values of three endogenous reference mRNAs (encoding DNA-binding protein, histone H4 and pyruvate decarboxylase) and Cq^virus^ is the Cq value of the viral strain.

The data were analyzed using Bio-Rad CFX Maestro 1.1 (Bio-Rad) and R software 4.3.0 [61].

### 4.4. High-Throughput Sequencing and Data Analysis

The plants and respective sequencing libraries are listed in Table 2. The sequencing libraries were prepared from small RNA extracts following the manufacturer’s recommendations. The NEBNext Multiplex Small RNA Library Prep Kit for Illumina was used. The ready-to-load libraries were processed using either the NovaSeq6000 or HiSeq 2500 system. Raw reads were quality-, library adapter-(5′-AGACGTGTGCTCTTCCGATCT-3′), and length-trimmed (18–30 nt selection) and a random selection of either 7 or 10 million reads was performed (Table S1, detailed view). The obtained reads were then mapped to eleven viral sequences using the ‘Map to reference’ tool of Geneious (Biomatters, Inc., Auckland, New Zealand).

Specifically, the minimum identity was set to 100%, and the reads matching more than one reference sequence were mapped either to all or none of them, depending on the required result. Assuming the presence of more than one strain in the data, we set mapping conditions that were rather stringent, without allowed mismatches. The mapping settings allowed us to establish rules for a read matching more than one position (reference): (a) discard the read, (b) map it to all matching positions or (c) map it to only one position through a random algorithm. In the current study, we used the first two options. When such reads were mapped to all references, they were referred to as ‘mapped reads’; when such reads were discarded, thus retaining only reference-unique reads, they were referred to as ‘strain-specific’ (Table 1, Figures S1 and S3).

The read counts were analyzed using R software 4.3.0 [61] and Exploratory 6.12.4 (Exploratory, Inc., Mill Valley, CA, USA).

## Figures and Tables

**Table 1:** Overview of mapped reads per a million total reads in the different small RNA datasets of viral references: strawberry mottle virus (SMoV), strawberry crinkle virus (SCV) and strawberry virus 1 (StrV-1). Further mapping details are listed in Table S1.

Virus	Strain	Plant										
		1B				2A			3K			
		1B_1	1B_2	1B_3	1B_4	2A_2	2A_3	2A_4	3K_1	3K_2	3K_3	3K_4
SMoV	RNA1A	1087	772	732	722	799	750	606	846	906	564	927
	RNA1B	1013	712	686	656	716	649	537	751	767	508	818
	RNA1C	1064	744	709	671	777	724	581	823	829	531	884
	RNA2A	715	507	506	487	472	431	381	533	544	353	549
	RNA2B	615	406	416	418	407	418	309	441	470	305	486
	RNA2C	955	589	599	626	607	581	463	606	612	421	672
SCV	A	781	152	255	701	1319	1091	565	974	1122	1389	1674
	B	813	105	137	346	1081	1072	434	659	720	657	1161
StrV-1	A	265	171	114	138	772	770	301	286	394	244	398
	B	588	427	420	356	1300	1647	834	727	1115	642	872
	C	116	129	66	111	503	423	143	237	276	200	273

**Table 2:** Plants used in the study, their virus status and respective library identification.

Plant	Viruses	Obtained Via	Libraries
1B	SCV-A, -B, SMoV RNA1^ABC^, RNA2^ABC^, StrV-1-A, -B, -C	Stolon propagation	1B1, 1B2, 1B3, 1B4
2A	--‘’--	--‘’--	2A2, 2A3, 2A4
3K	--‘’--	--‘’--	3K1, 3K2, 3K3, 3K4
Fv_StrV-1_A	StrV-1-A	Aphid transmission	S98, S95
Fv_StrV-1_B	StrV-1-B	--‘’--	S107, S108, S99
Fv_StrV-1_AB	StrV-1-A, -B	Wedge grafting petiole	S101
Fv-SMoV_BC_AC-1	SMoV RNA1^BC^, RNA2^AC^	Aphid transmission	S1, S2
Fv-SMoV_BC_AC-2	--‘’--	--‘’--	S3, S4, S92

## Data Availability

Data generated during the study were deposited at the NCBI repository under accession number SAMN34995356-77.

## Supplementary Materials

The following supporting information can be downloaded at: https://www.mdpi.com/article/10.3390/plants12132564/s1, Table S1: Overview of the mapped reads to viral references: strawberry crinkle virus, strawberry mottle virus and strawberry virus 1; Table S2: Overview of Cq, ∆Cq and fold difference values between the viral targets and endogenous references; Table S3: List of primers used in the study; Figure S1: Mapping and distribution plots of vsiRNAs (reads) from the 1B, 2A and 3K samples; Figure S2: Mapping and distribution plots of vsiRNAs (reads) from the Fv-SMoV_BC_AC samples; Figure S3: Mapping and distribution plots of vsiRNAs (reads) from the Fv_StrV-1_A, Fv_StrV-1_B and Fv_StrV-1_AB samples. Reference [62] is cited in the supplementary materials.
