# Supplementary material for: Analysis of Virus-Derived siRNAs in Strawberry Plants Co-Infected with Multiple Viruses and Their Genotypes
Source: Plants (Basel). 2023 Jul 6;12(13):2564. doi: 10.3390/plants12132564 (PMC10346599; doi:10.3390/plants12132564)
Supplement: Supplementary file 1 [file plants-12-02564-s001.zip › Figure S1.pdf]

Figure S1 Distributionof viral small interferingRNAs (reads) fromthe 1B, 2A, and 3K samples.

Datasets were named after respective plant withan addition of a number suffix, i.e 1B1, 1B2 and so on.

Color legends show strand specificity of the mapped reads. Axes of abscissas show genomic position in nucleotides, axes of ordinary – sequence (mapping) coverage. Termini direction is from 5' to 3' (left to right).

qPCR detection: all strawberry crinkle (SCV), strawberry mottle virus (SMoV) and strawberry virus 1 (StrV1) strains were detected in all samples

Mappings of the 1B_1 reads against the viral references.

Dataset	Input reads	Virus	Reference	Mapped reads	Mapped reads, %	RPKM	Forward/Reverse balance	Nonredundant, %	Strain specific	Strain specific, % from total mapped reads	Strain specific, RPKM
1B_1	10000000	SCV	SCV_A	7814	0.078	53.7	0.8	55.9	6504	83.2	44.7

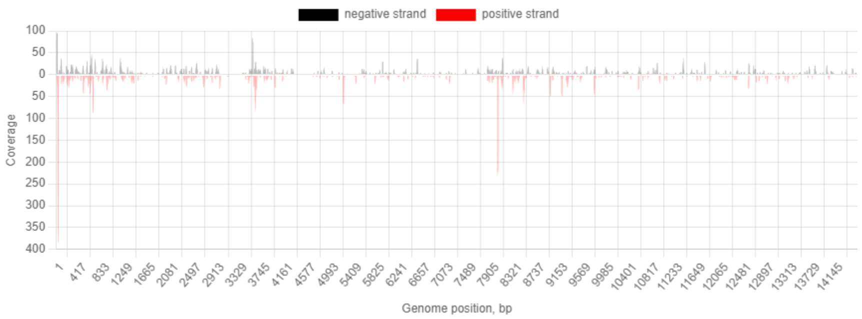

Mapping coverage of all reads matching SCV_A (7814reads)

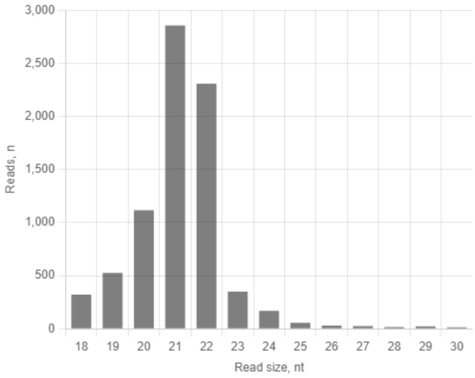

Length distribution of the reads

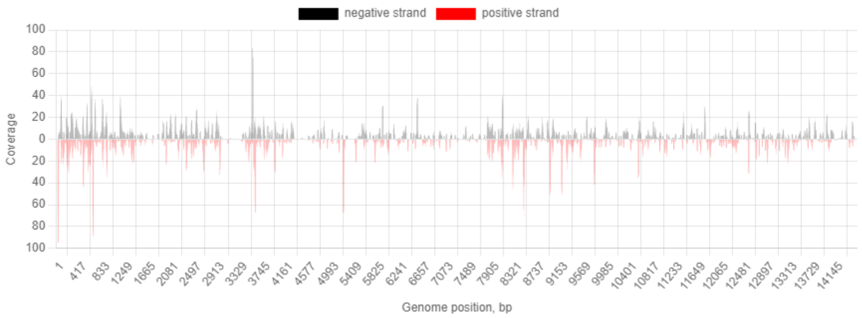

Mapping coverage of SCV_A specific reads (6504reads)

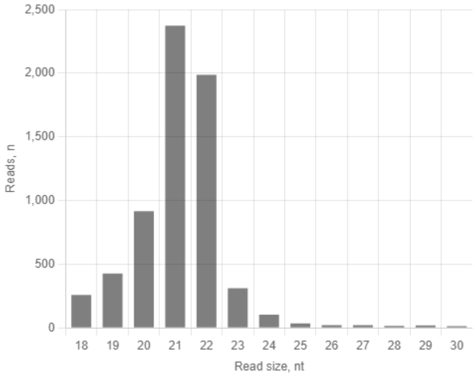

Length distribution of the reads

Dataset	Input reads	Virus	Reference	Mapped reads	Mapped reads, %	RPKM	Forward/Reverse balance	Nonredundant, %	Strain specific	Strain specific, % from total mapped reads	Strain specific, RPKM
1B_1	10000000	SCV	SCV_B	8134	0.081	55.9	0.7	55.2	6824	83.9	46.9

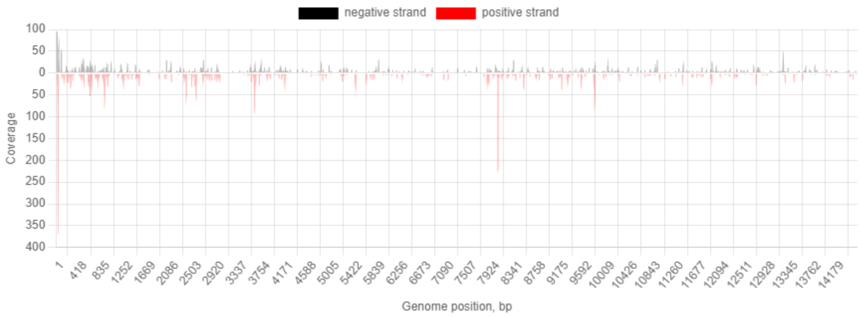

Mapping coverage of all reads matching SCV_B (8134reads)

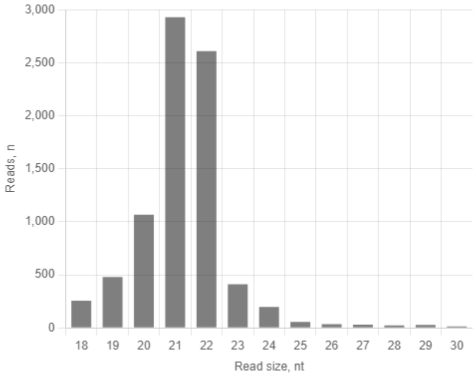

Length distribution of the reads

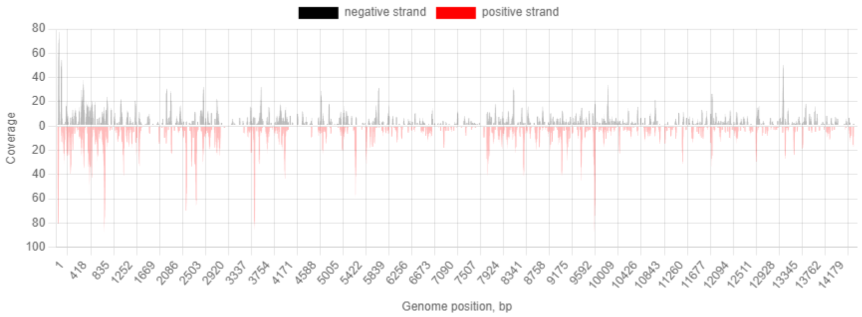

Mapping coverage of SCV_B specific reads (6824reads)

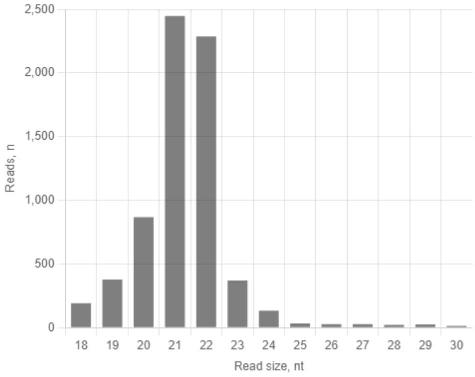

Length distribution of the reads

Dataset	Input reads	Virus	Reference	Mapped reads	Mapped reads, %	RPKM	Forward/Reverse balance	Nonredundant, %	Strain specific	Strain specific, % from total mapped reads	Strain specific, RPKM
1B_1	10000000	SMoV	SMoV_RNA1A	10871	0.109	154.5	1.5	40.2	5469	50.3	77.7

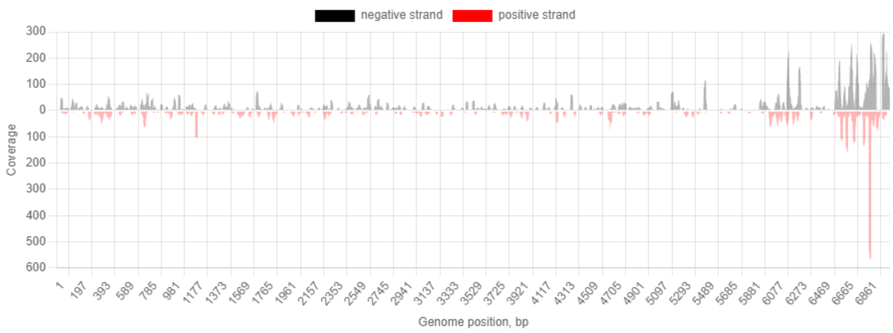

Mapping coverage of all reads matching SMoV_RNA1A (10871reads)

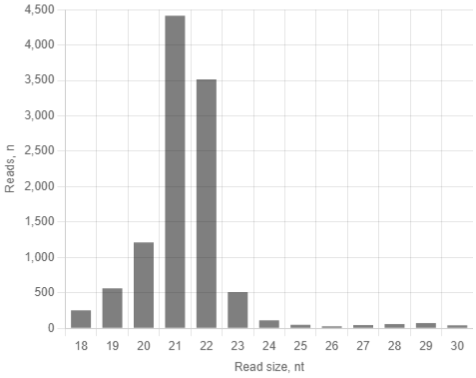

Length distribution of the reads

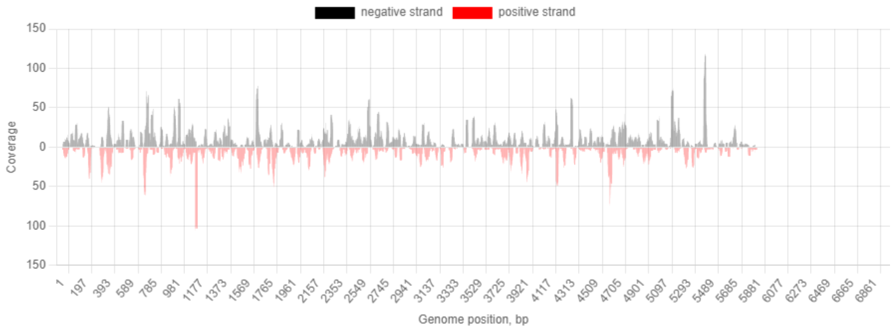

Mapping coverage of SMoV_RNA1A specific reads (5469reads)

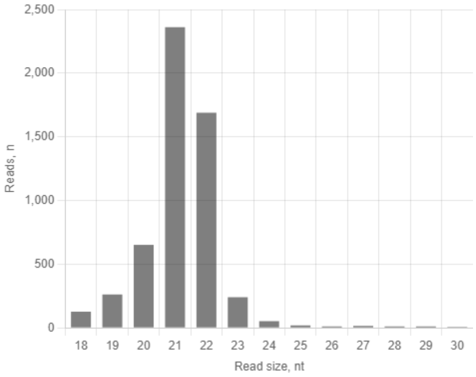

Length distribution of the reads

Dataset	Input reads	Virus	Reference	Mapped reads	Mapped reads, %	RPKM	Forward/Reverse balance	Nonredundant, %	Strain specific	Strain specific, % from total mapped reads	Strain specific, RPKM
1B_1	10000000	SMoV	SMoV_RNA1B	10125	0.101	143.6	1.4	39.1	4704	46.5	66.7

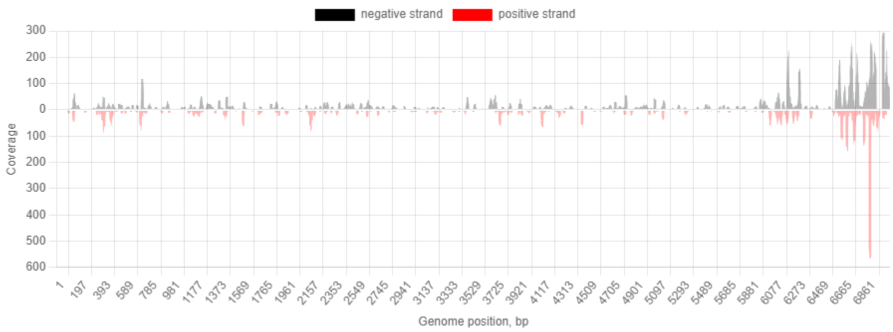

Mapping coverage of all reads matching SMoV_RNA1B (10125reads)

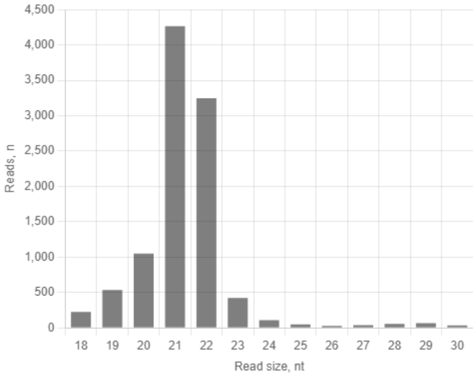

Length distribution of the reads

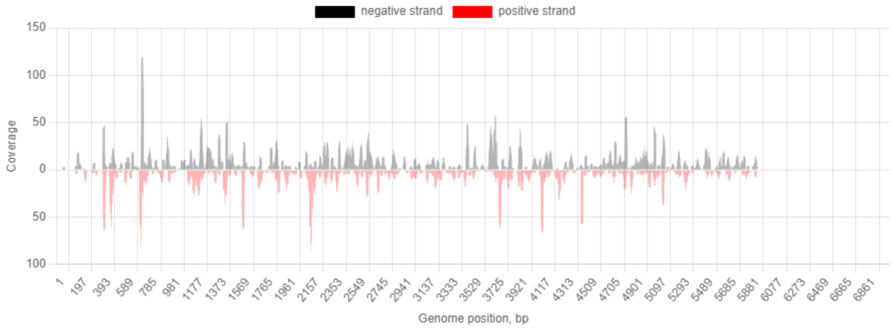

Mapping coverage of SMoV_RNA1B specific reads (4704reads)

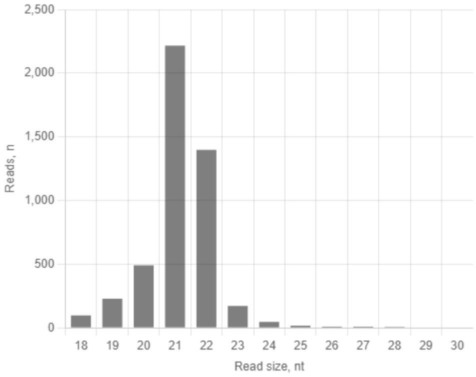

Length distribution of the reads

Dataset	Input reads	Virus	Reference	Mapped reads	Mapped reads, %	RPKM	Forward/Reverse balance	Nonredundant, %	Strain specific	Strain specific, % from total mapped reads	Strain specific, RPKM
1B_1	10000000	SMoV	SMoV_RNA1C	10639	0.106	151.1	1.3	39.4	5248	49.3	74.5

Dataset	Input reads	Virus	Reference	Mapped reads	Mapped reads, %	RPKM	Forward/Reverse balance	Nonredundant, %	Strain specific	Strain specific, % from total mapped reads	Strain specific, RPKM

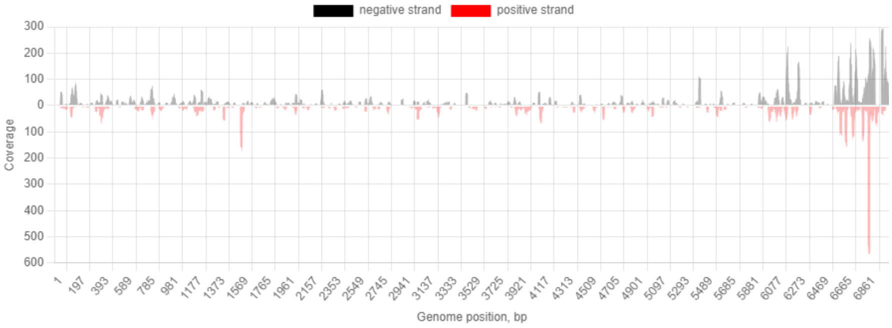

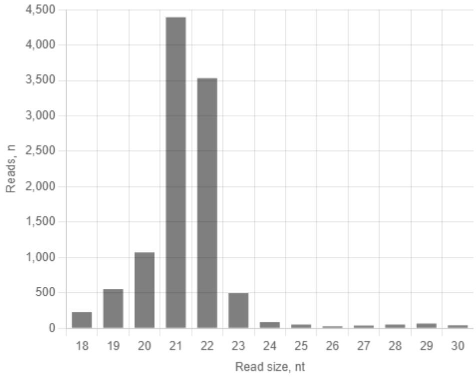

Mapping coverage of all reads matching SMoV_RNA1C (10639reads)

Length distribution of the reads

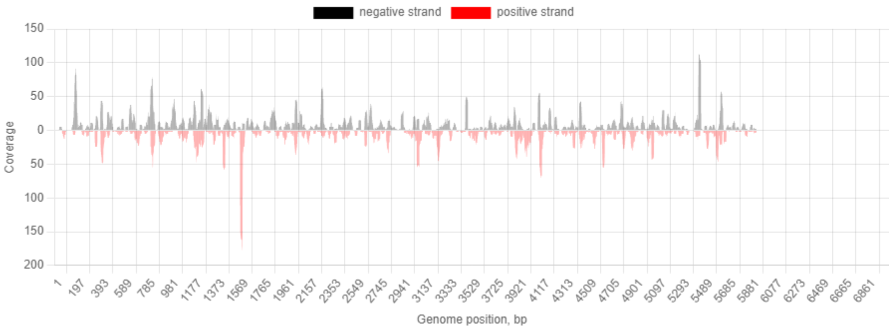

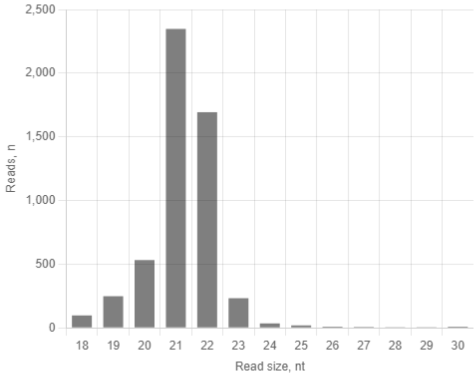

Mapping coverage of SMoV_RNA1C specific reads (5248reads)

Length distribution of the reads

Dataset	Input reads	Virus	Reference	Mapped reads	Mapped reads, %	RPKM	Forward/Reverse balance	Nonredundant, %	Strain specific	Strain specific, % from total mapped reads	Strain specific, RPKM
1B_1	10000000	SMoV	SMoV_RNA2A	7149	0.071	114.1	1.5	43.4	4714	65.9	75.2

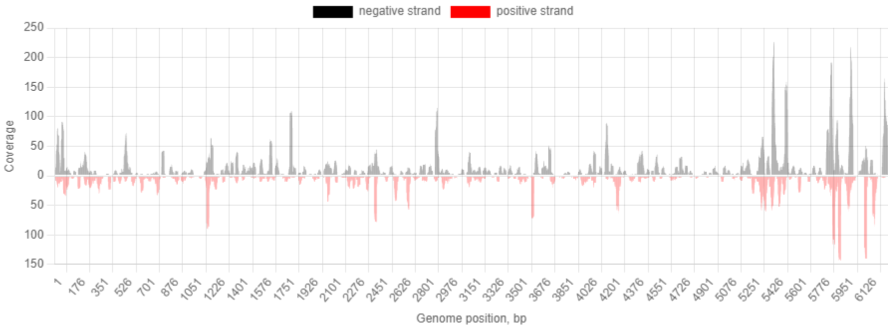

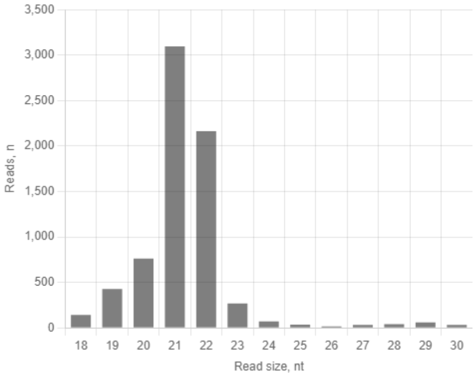

Mapping coverage of all reads matching SMoV_RNA2A (7149reads)

Length distribution of the reads

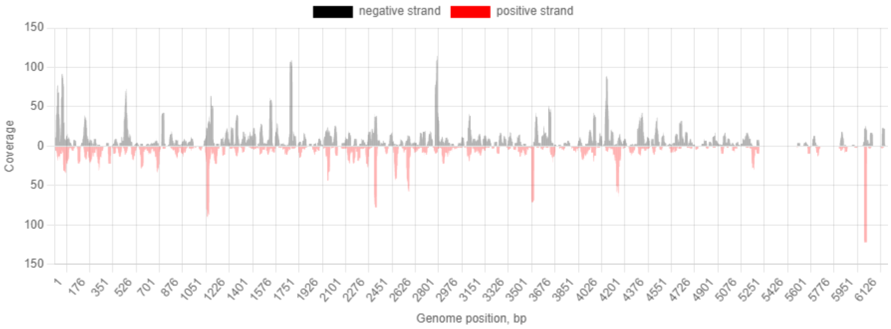

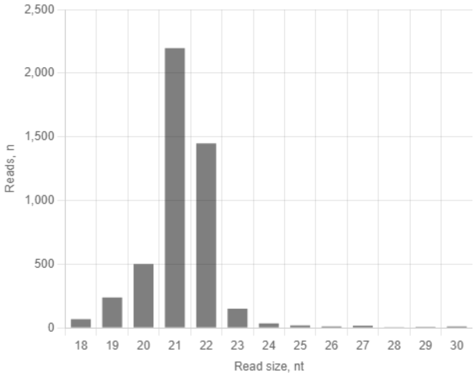

Mapping coverage of SMoV_RNA2A specific reads (4714reads)

Length distribution of the reads

Dataset	Input reads	Virus	Reference	Mapped reads	Mapped reads, %	RPKM	Forward/Reverse balance	Nonredundant, %	Strain specific	Strain specific, % from total mapped reads	Strain specific, RPKM
1B_1	10000000	SMoV	SMoV_RNA2B	6150	0.061	97.3	1.6	43.7	3027	49.2	47.9

Dataset	Input reads	Virus	Reference	Mapped reads	Mapped reads, %	RPKM	Forward/Reverse balance	Nonredundant, %	Strain specific	Strain specific, % from total mapped reads	Strain specific, RPKM

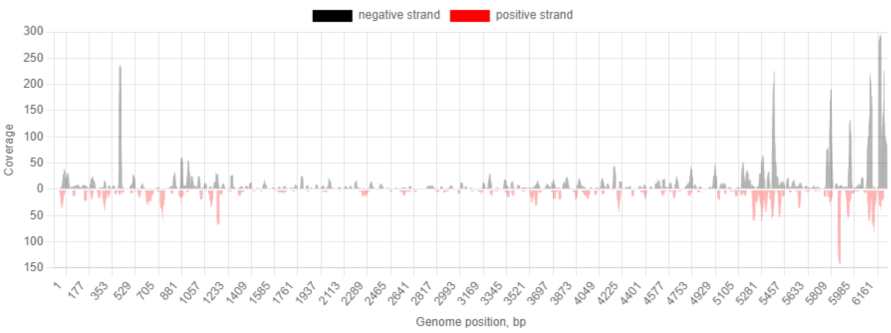

Mapping coverage of all reads matching SMoV_RNA2B (6150reads)

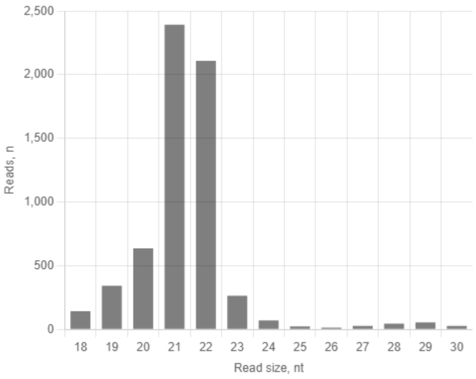

Length distribution of the reads

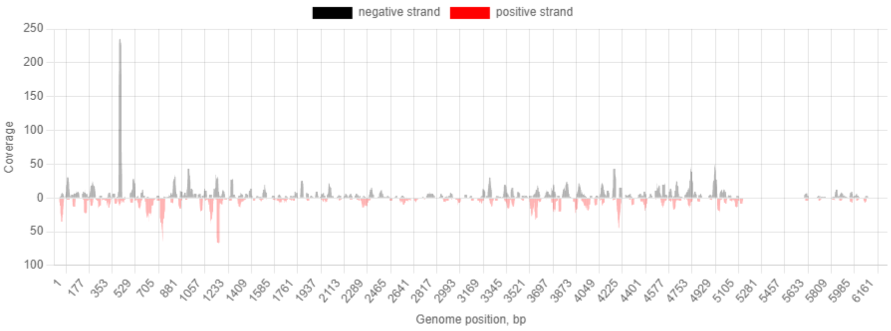

Mapping coverage of SMoV_RNA2B specific reads (3027reads)

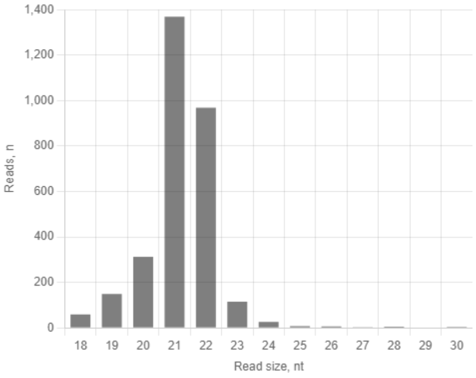

Length distribution of the reads

Dataset	Input reads	Virus	Reference	Mapped reads	Mapped reads, %	RPKM	Forward/Reverse balance	Nonredundant, %	Strain specific	Strain specific, % from total mapped reads	Strain specific, RPKM
1B_1	10000000	SMoV	SMoV_RNA2C	9549	0.095	150.7	1.5	38.4	4485	47.0	70.8

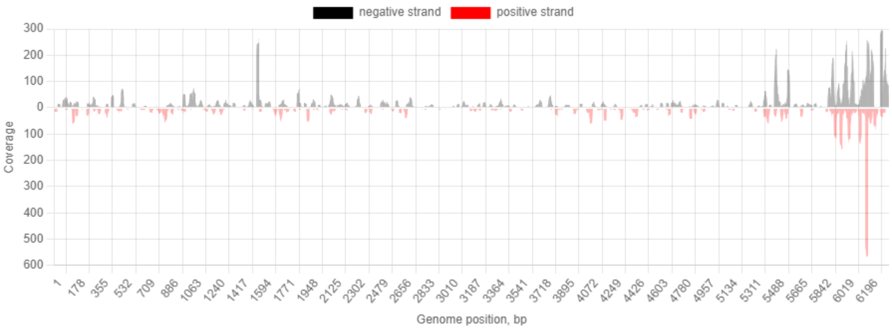

Mapping coverage of all reads matching SMoV_RNA2C (9549reads)

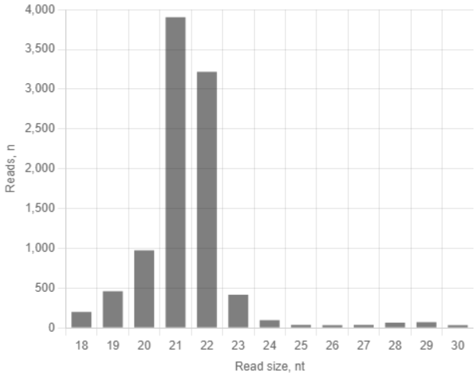

Length distribution of the reads

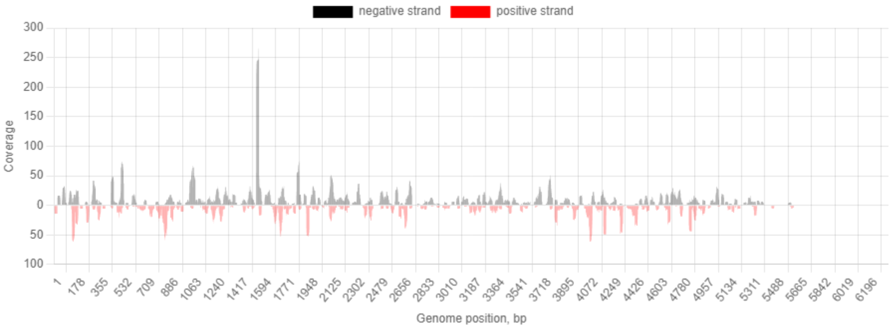

Mapping coverage of SMoV_RNA2C specific reads (4485reads)

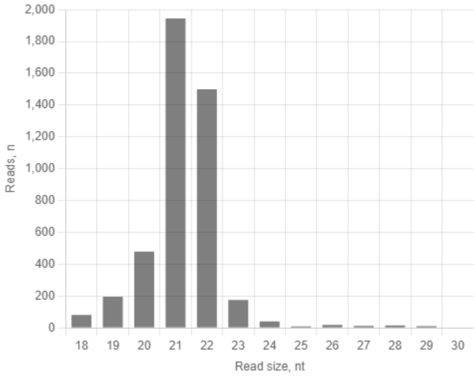

Length distribution of the reads

Dataset	Input reads	Virus	Reference	Mapped reads	Mapped reads, %	RPKM	Forward/Reverse balance	Nonredundant, %	Strain specific	Strain specific, % from total mapped reads	Strain specific, RPKM
1B_1	10000000	StrV1	StrV1_A	2694	0.027	19.0	0.9	65.8	2267	84.1	16.0

Dataset	Input reads	Virus	Reference	Mapped reads	Mapped reads, %	RPKM	Forward/Reverse balance	Nonredundant, %	Strain specific	Strain specific, % from total mapped reads	Strain specific, RPKM

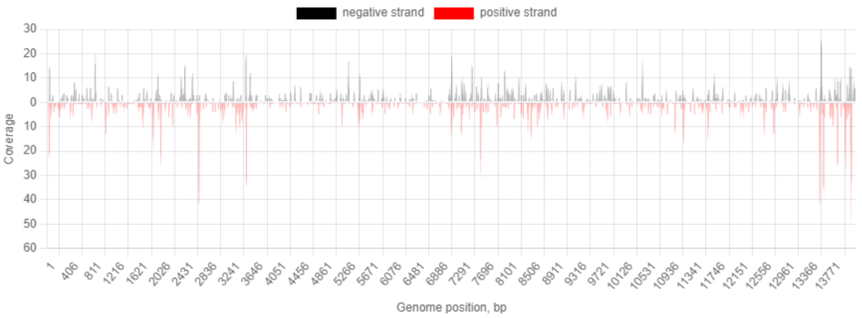

Mapping coverage of all reads matching StrV1_A (2694 reads)

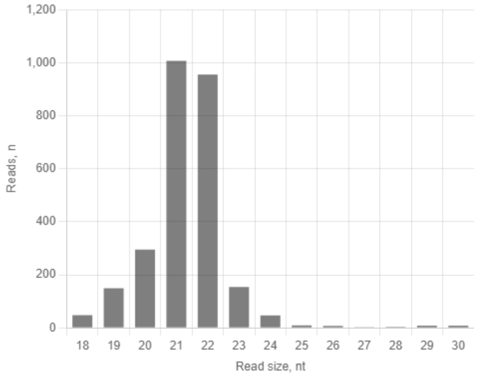

Length distribution of the reads

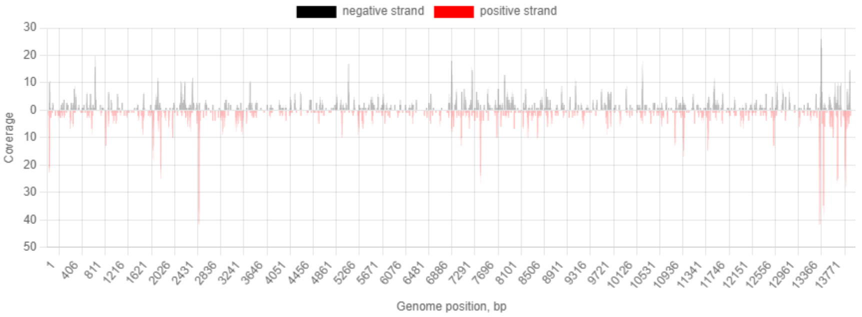

Mapping coverage of StrV1_A specific reads (2267reads)

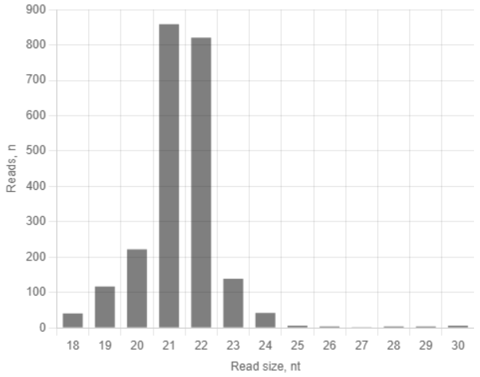

Length distribution of the reads

Dataset	Input reads	Virus	Reference	Mapped reads	Mapped reads, %	RPKM	Forward/Reverse balance	Nonredundant, %	Strain specific	Strain specific, % from total mapped reads	Strain specific, RPKM
1B_1	10000000	StrV1	StrV1_B	5882	0.059	41.9	1.1	43.7	5741	97.6	40.9

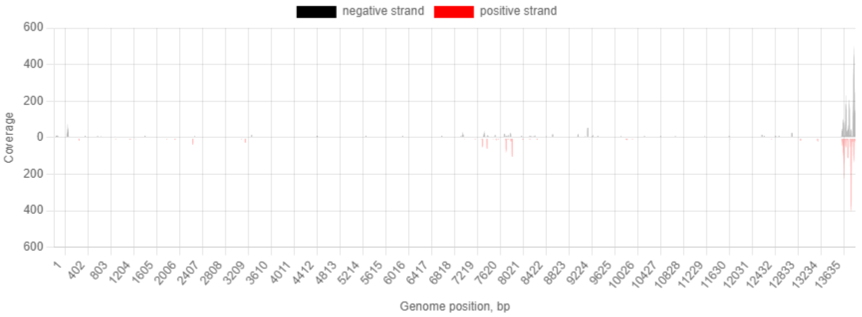

Mapping coverage of all reads matching StrV1_B (5882reads)

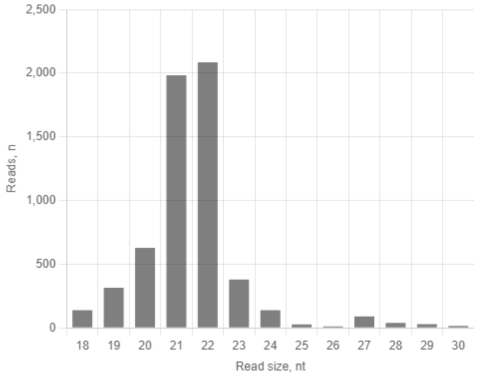

Length distribution of the reads

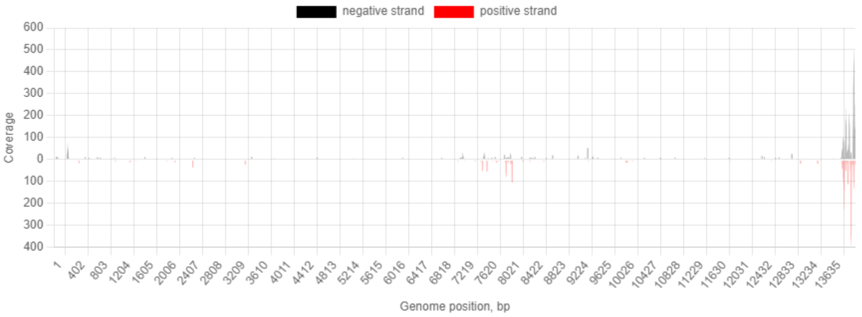

Mapping coverage of StrV1_B specific reads (5741reads)

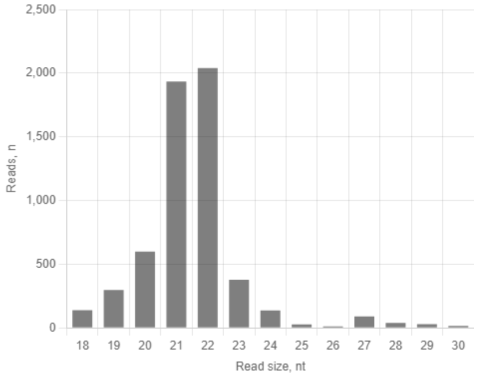

Length distribution of the reads

Dataset	Input reads	Virus	Reference	Mapped reads	Mapped reads, %	RPKM	Forward/Reverse balance	Nonredundant, %	Strain specific	Strain specific, % from total mapped reads	Strain specific, RPKM
1B_1	10000000	StrV1	StrV1_C	1158	0.012	8.1	0.9	68.7	763	65.9	5.4

Dataset	Input reads	Virus	Reference	Mapped reads	Mapped reads, %	RPKM	Forward/Reverse balance	Nonredundant, %	Strain specific	Strain specific, % from total mapped reads	Strain specific, RPKM

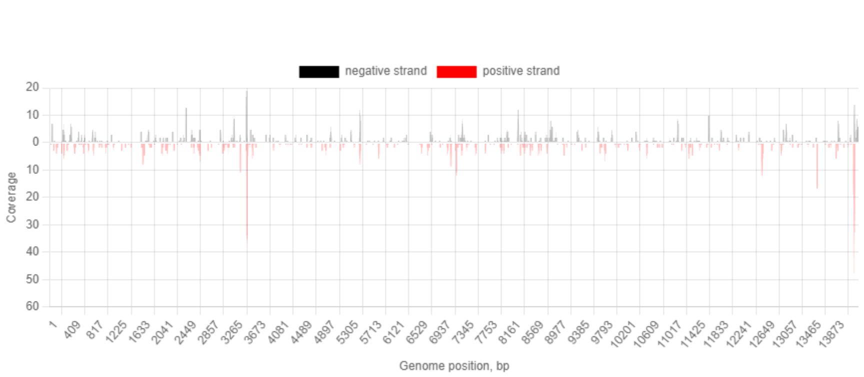

Mapping coverage of all reads matching StrV1_C (1158reads)

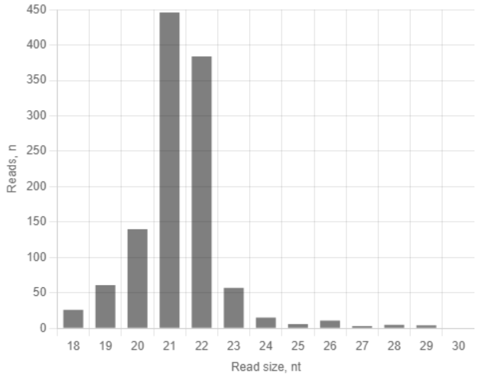

Length distribution of the reads

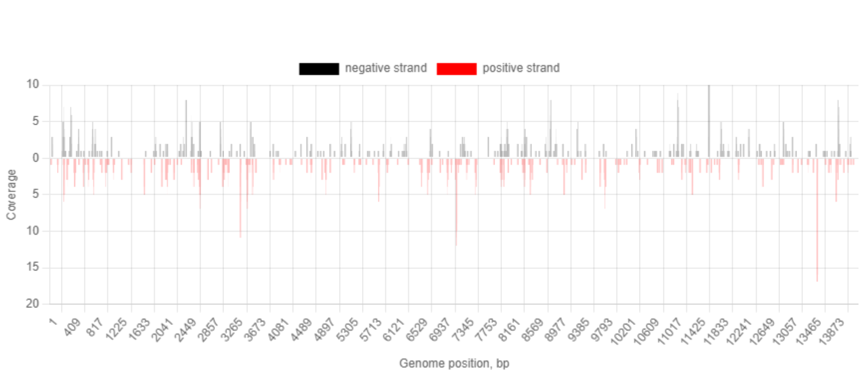

Mapping coverage of StrV1_C specific reads (763 reads)

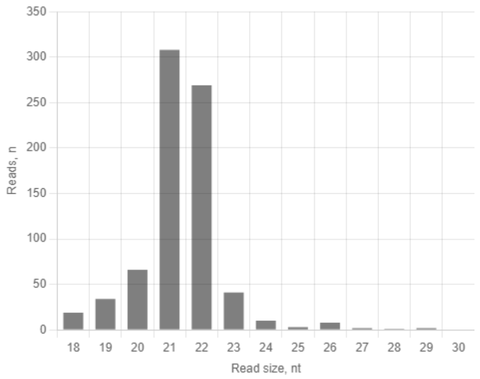

Length distribution of the reads

Mappings of the 1B_2 reads against the viral references.

Dataset	Input reads	Virus	Reference	Mapped reads	Mapped reads, %	RPKM	Forward/Reverse balance	Nonredundant, %	Strain specific	Strain specific, % from total mapped reads	Strain specific, RPKM
1B_2	10000000	SCV	SCV_A	1523	0.015	10.5	0.8	46.7	6504	427.1	9.0

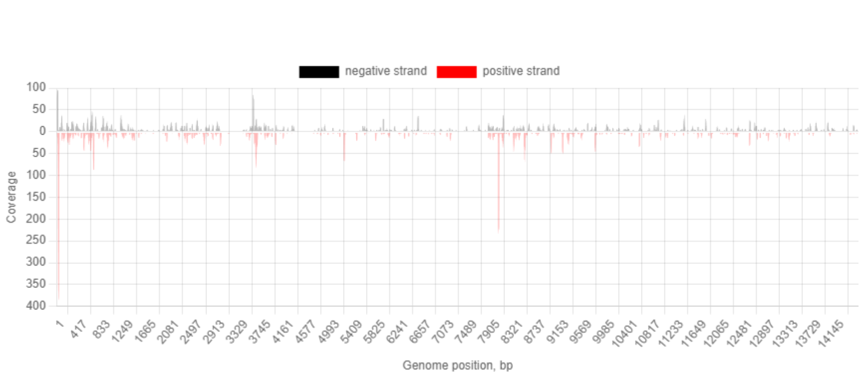

Mapping coverage of all reads matching SCV_A (7814reads)

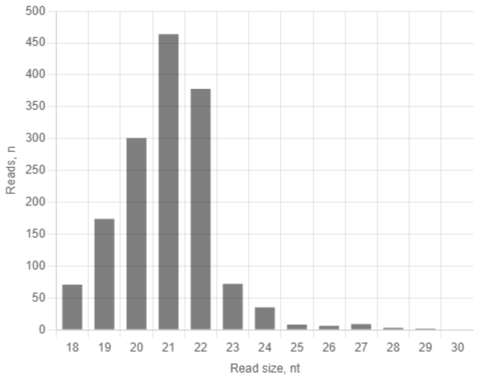

Length distribution of the reads

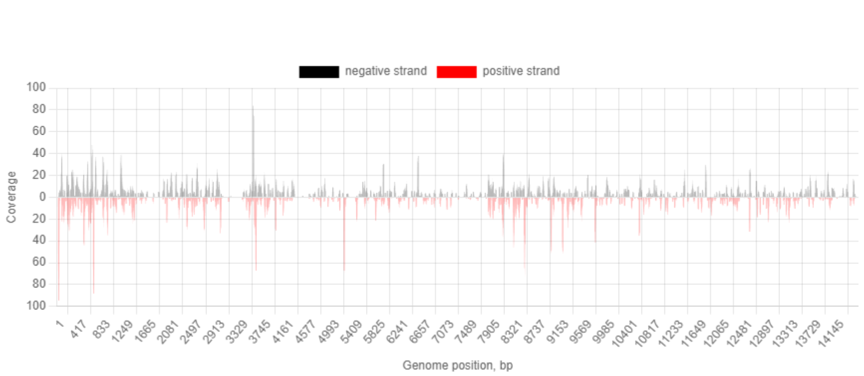

Mapping coverage of SCV_A specific reads (6504reads)

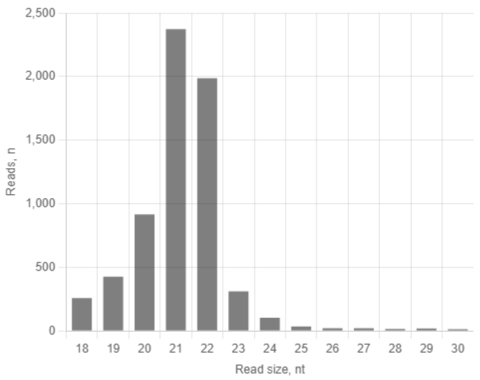

Length distribution of the reads

Dataset	Input reads	Virus	Reference	Mapped reads	Mapped reads, %	RPKM	Forward/Reverse balance	Nonredundant, %	Strain specific	Strain specific, % from total mapped reads	Strain specific, RPKM
1B_2	10000000	SCV	SCV_B	1046	0.010	7.2	0.6	45.4	6824	652.4	5.7

Dataset	Input reads	Virus	Reference	Mapped reads	Mapped reads, %	RPKM	Forward/Reverse balance	Nonredundant, %	Strain specific	Strain specific, % from total mapped reads	Strain specific, RPKM

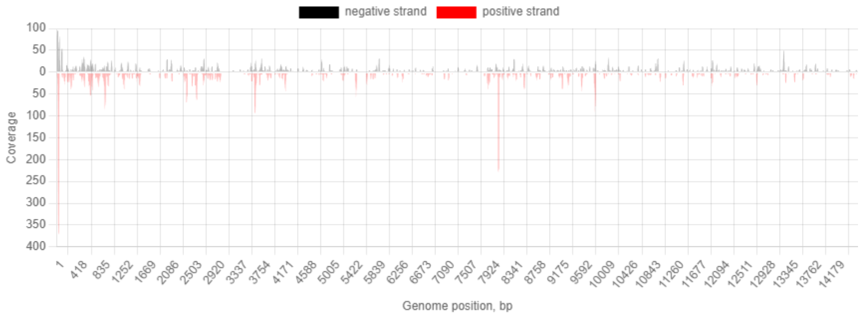

Mapping coverage of all reads matching SCV_B (8134reads)

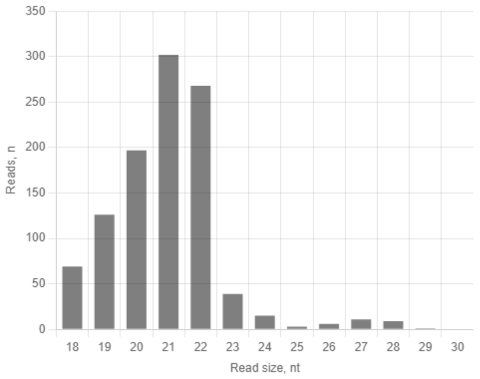

Length distribution of the reads

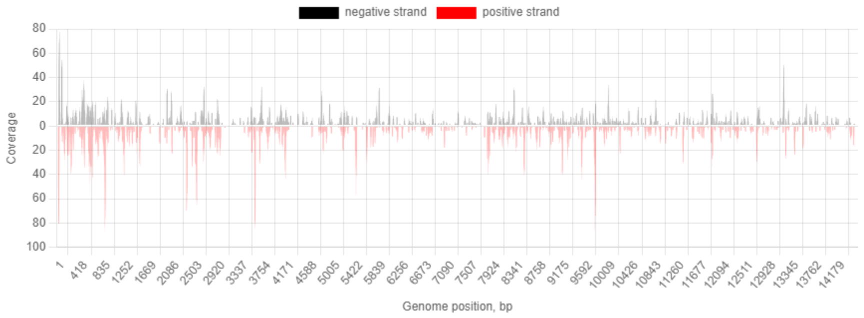

Mapping coverage of SCV_B specific reads (6824reads)

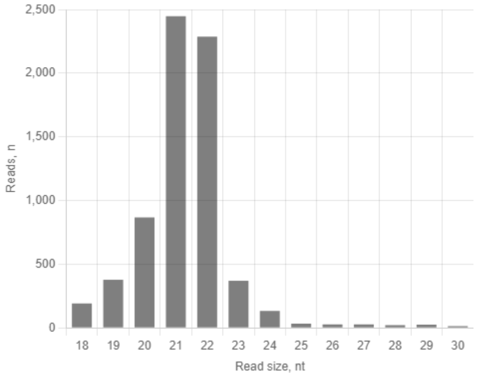

Length distribution of the reads

Dataset	Input reads	Virus	Reference	Mapped reads	Mapped reads, %	RPKM	Forward/Reverse balance	Nonredundant, %	Strain specific	Strain specific, % from total mapped reads	Strain specific, RPKM
1B_2	10000000	SMoV	SMoV_RNA1A	7716	0.077	109.6	1.8	30.5	5469	70.9	55.9

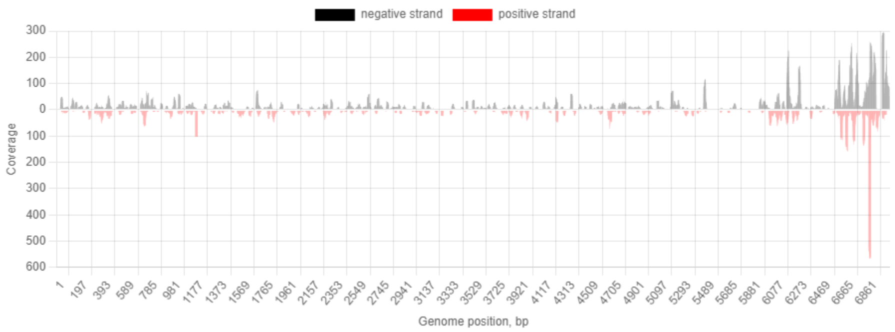

Mapping coverage of all reads matching SMoV_RNA1A (10871reads)

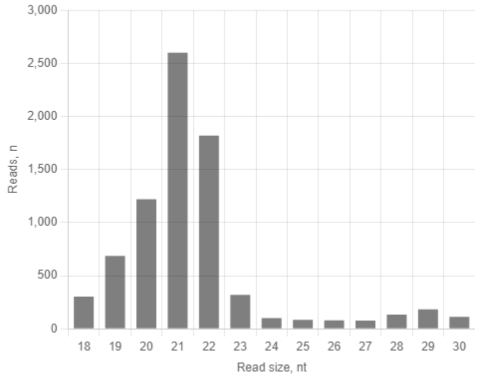

Length distribution of the reads

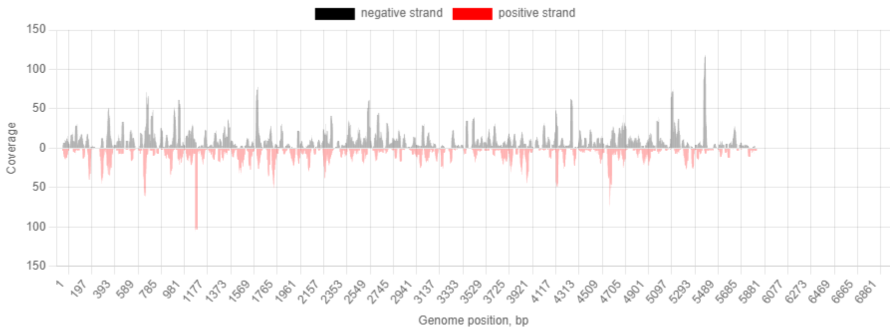

Mapping coverage of SMoV_RNA1A specific reads (5469reads)

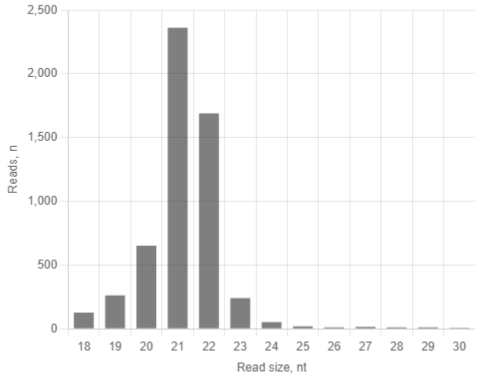

Length distribution of the reads

Dataset	Input reads	Virus	Reference	Mapped reads	Mapped reads, %	RPKM	Forward/Reverse balance	Nonredundant, %	Strain specific	Strain specific, % from total mapped reads	Strain specific, RPKM
1B_2	10000000	SMoV	SMoV_RNA1B	7121	0.071	101.0	1.6	30.9	4704	66.1	47.1

Dataset	Input reads	Virus	Reference	Mapped reads	Mapped reads, %	RPKM	Forward/Reverse balance	Nonredundant, %	Strain specific	Strain specific, % from total mapped reads	Strain specific, RPKM

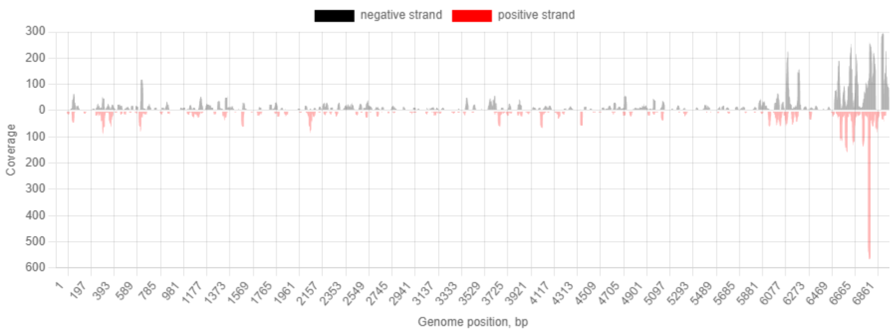

Mapping coverage of all reads matching SMoV_RNA1B (10125reads)

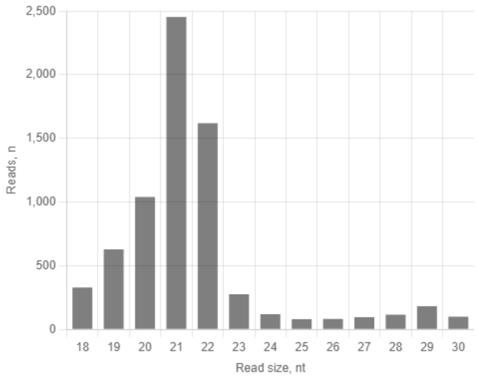

Length distribution of the reads

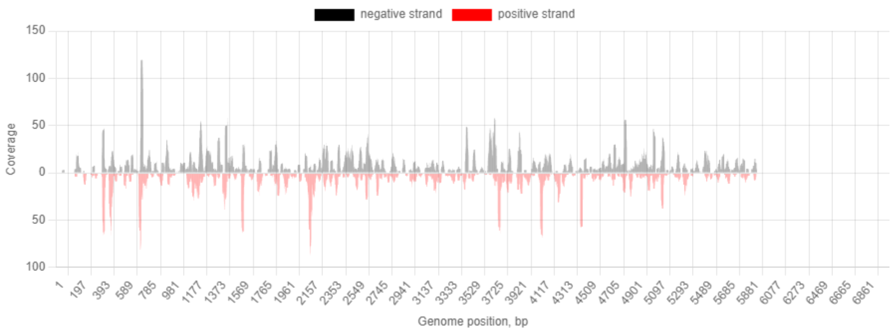

Mapping coverage of SMoV_RNA1B specific reads (4704reads)

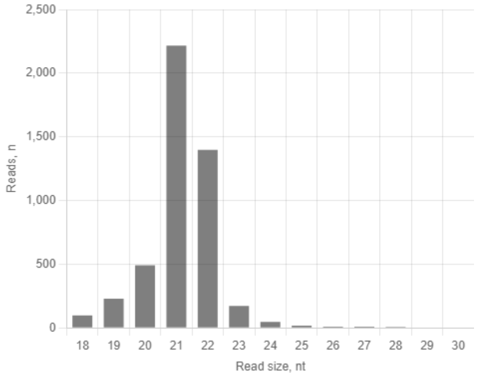

Length distribution of the reads

Dataset	Input reads	Virus	Reference	Mapped reads	Mapped reads, %	RPKM	Forward/Reverse balance	Nonredundant, %	Strain specific	Strain specific, % from total mapped reads	Strain specific, RPKM
1B_2	10000000	SMoV	SMoV_RNA1C	7443	0.074	105.7	1.7	31.3	5248	70.5	51.6

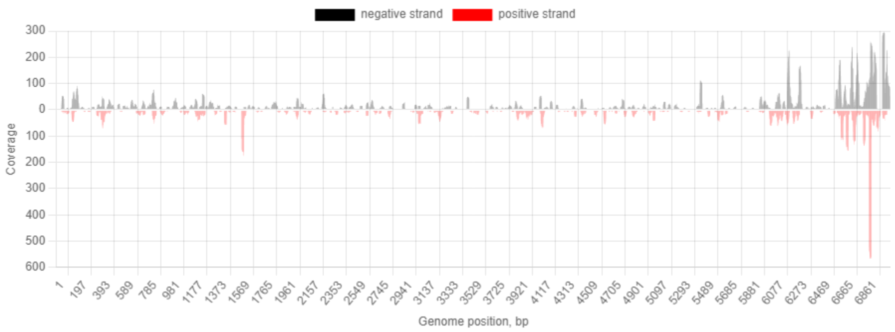

Mapping coverage of all reads matching SMoV_RNA1C (10639reads)

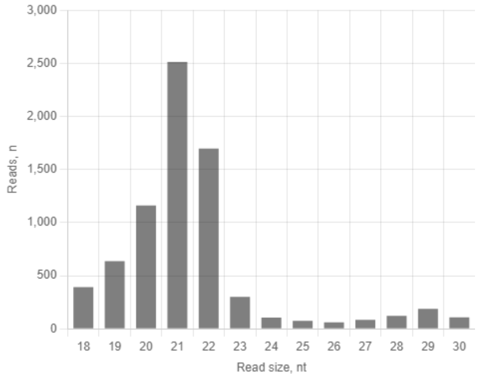

Length distribution of the reads

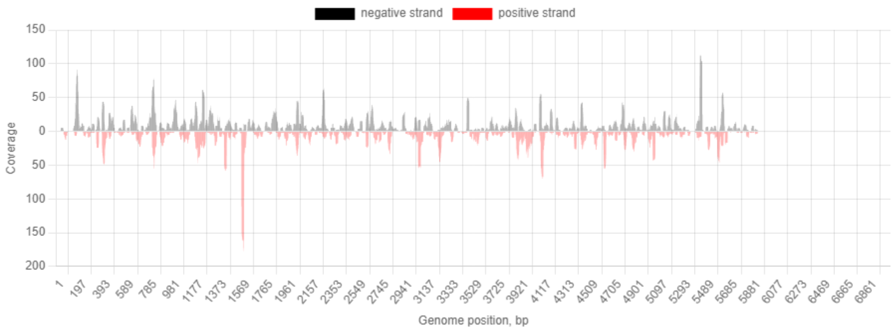

Mapping coverage of SMoV_RNA1C specific reads (5248reads)

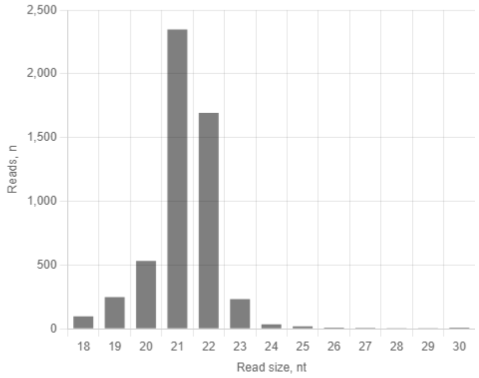

Length distribution of the reads

Dataset	Input reads	Virus	Reference	Mapped reads	Mapped reads, %	RPKM	Forward/Reverse balance	Nonredundant, %	Strain specific	Strain specific, % from total mapped reads	Strain specific, RPKM
1B_2	10000000	SMoV	SMoV_RNA2A	5073	0.051	80.9	1.7	34.5	4714	92.9	52.8

Dataset	Input reads	Virus	Reference	Mapped reads	Mapped reads, %	RPKM	Forward/Reverse balance	Nonredundant, %	Strain specific	Strain specific, % from total mapped reads	Strain specific, RPKM

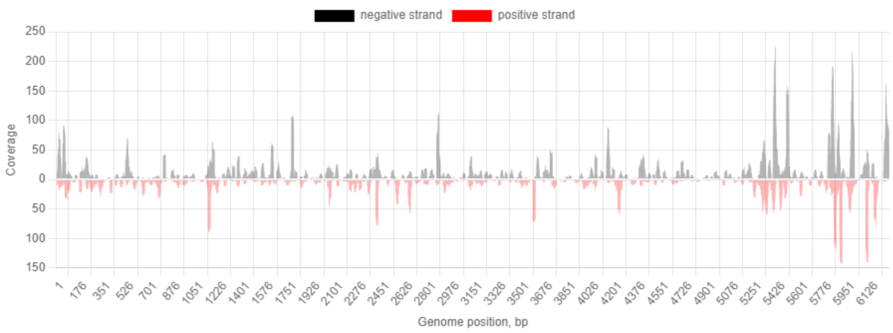

Mapping coverage of all reads matching SMoV_RNA2A (7149reads)

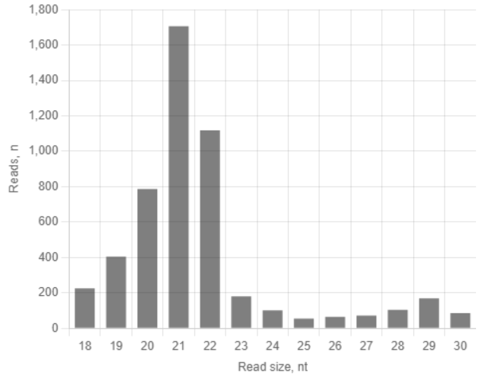

Length distribution of the reads

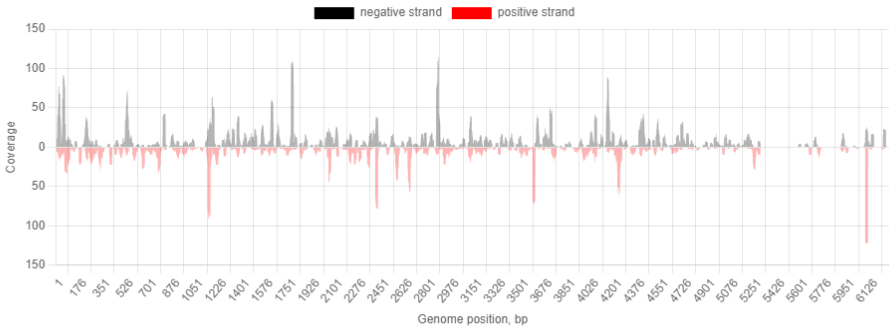

Mapping coverage of SMoV_RNA2A specific reads (4714reads)

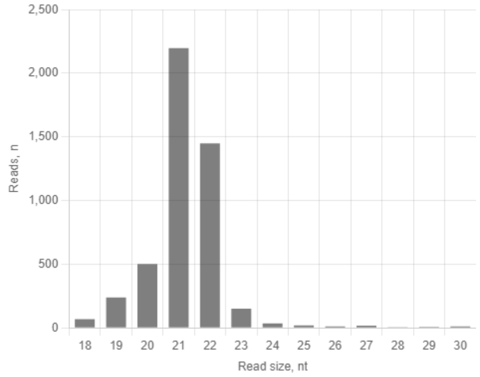

Length distribution of the reads

Dataset	Input reads	Virus	Reference	Mapped reads	Mapped reads, %	RPKM	Forward/Reverse balance	Nonredundant, %	Strain specific	Strain specific, % from total mapped reads	Strain specific, RPKM
1B_2	10000000	SMoV	SMoV_RNA2B	4059	0.041	64.2	2.0	34.1	3027	74.6	29.2

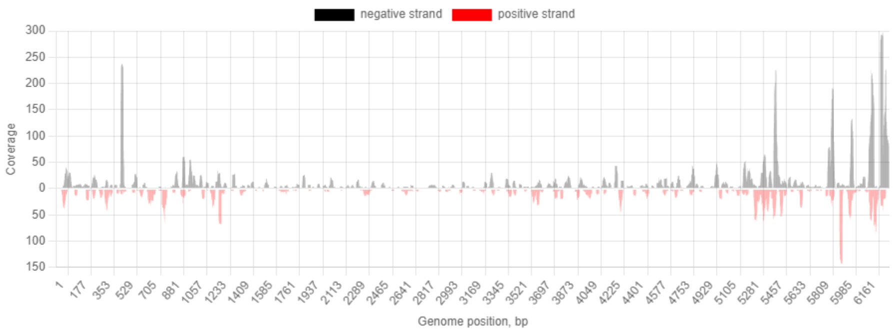

Mapping coverage of all reads matching SMoV_RNA2B (6150reads)

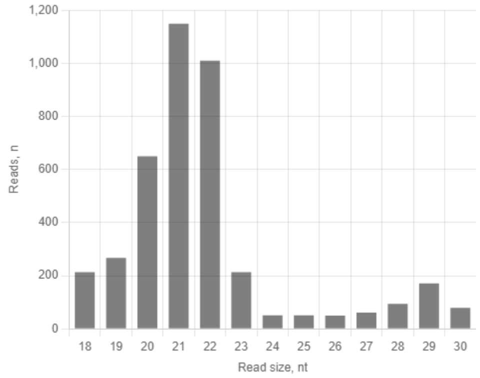

Length distribution of the reads

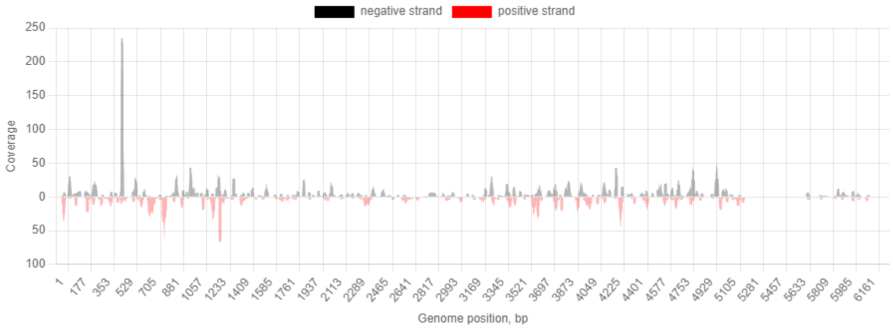

Mapping coverage of SMoV_RNA2B specific reads (3027reads)

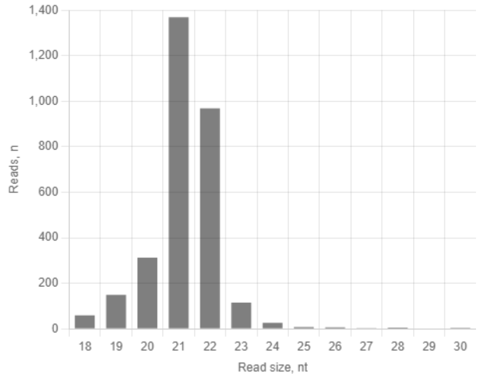

Length distribution of the reads

Dataset	Input reads	Virus	Reference	Mapped reads	Mapped reads, %	RPKM	Forward/Reverse balance	Nonredundant, %	Strain specific	Strain specific, % from total mapped reads	Strain specific, RPKM
1B_2	10000000	SMoV	SMoV_RNA2C	5894	0.059	92.8	1.7	29.4	4485	76.1	35.2

Dataset	Input reads	Virus	Reference	Mapped reads	Mapped reads, %	RPKM	Forward/Reverse balance	Nonredundant, %	Strain specific	Strain specific, % from total mapped reads	Strain specific, RPKM

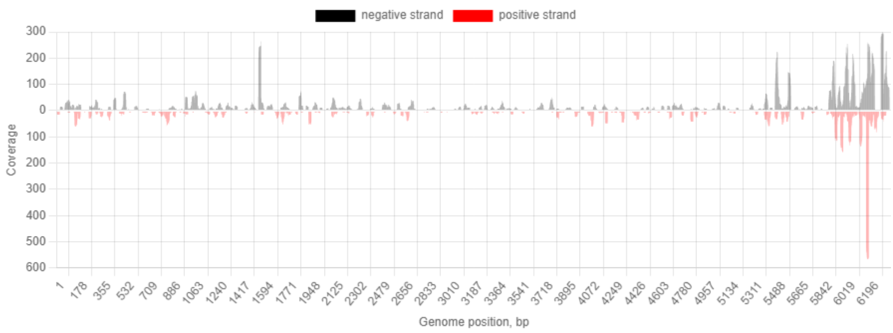

Mapping coverage of all reads matching SMoV_RNA2C (9549reads)

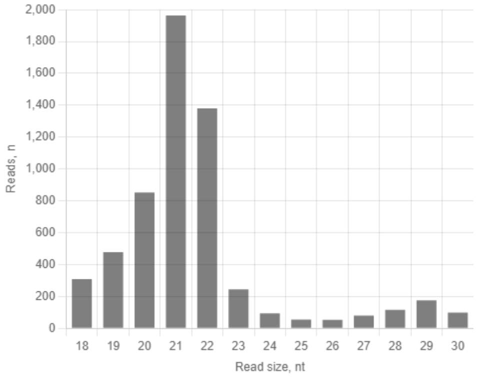

Length distribution of the reads

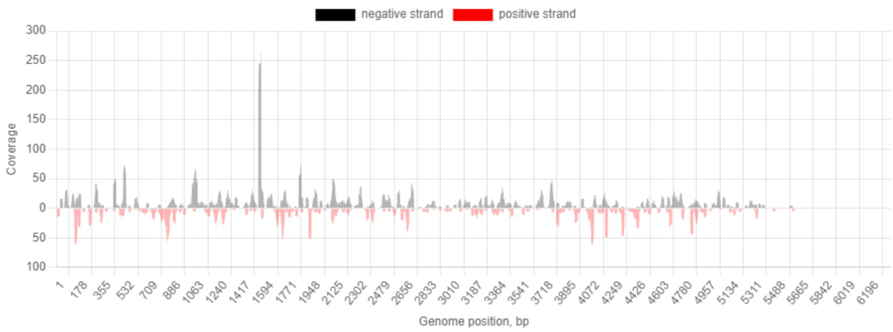

Mapping coverage of SMoV_RNA2C specific reads (4485reads)

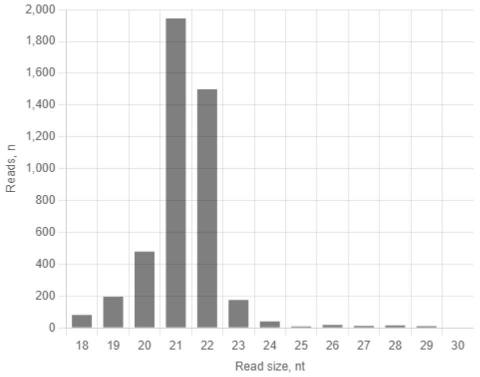

Length distribution of the reads

Dataset	Input reads	Virus	Reference	Mapped reads	Mapped reads, %	RPKM	Forward/Reverse balance	Nonredundant, %	Strain specific	Strain specific, % from total mapped reads	Strain specific, RPKM
1B_2	10000000	StrV1	StrV1_A	1710	0.017	12.1	1.0	41.9	2267	132.6	9.8

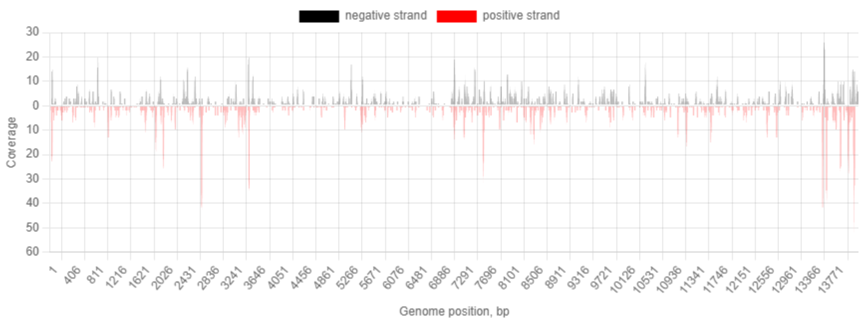

Mapping coverage of all reads matching StrV1_A (2694reads)

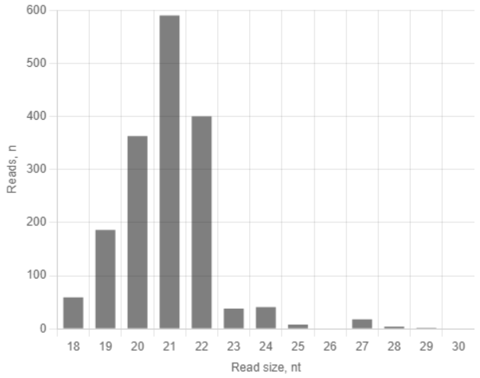

Length distribution of the reads

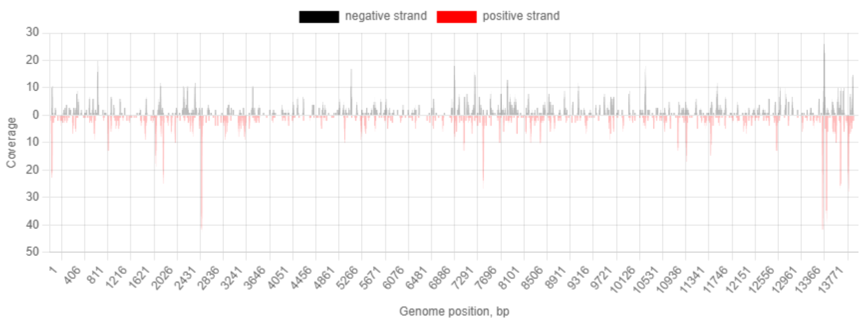

Mapping coverage of StrV1_A specific reads (2267reads)

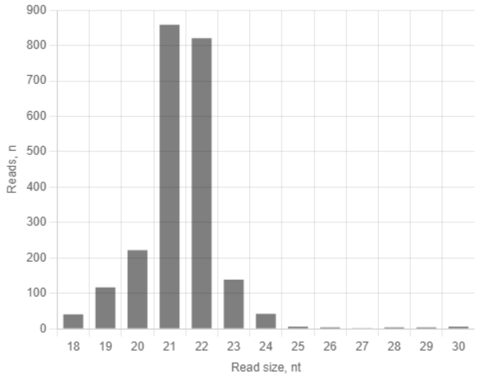

Length distribution of the reads

Dataset	Input reads	Virus	Reference	Mapped reads	Mapped reads, %	RPKM	Forward/Reverse balance	Nonredundant, %	Strain specific	Strain specific, % from total mapped reads	Strain specific, RPKM
1B_2	10000000	StrV1	StrV1_B	4273	0.043	30.5	1.4	30.4	5741	134.4	29.4

Dataset	Input reads	Virus	Reference	Mapped reads	Mapped reads, %	RPKM	Forward/Reverse balance	Nonredundant, %	Strain specific	Strain specific, % from total mapped reads	Strain specific, RPKM

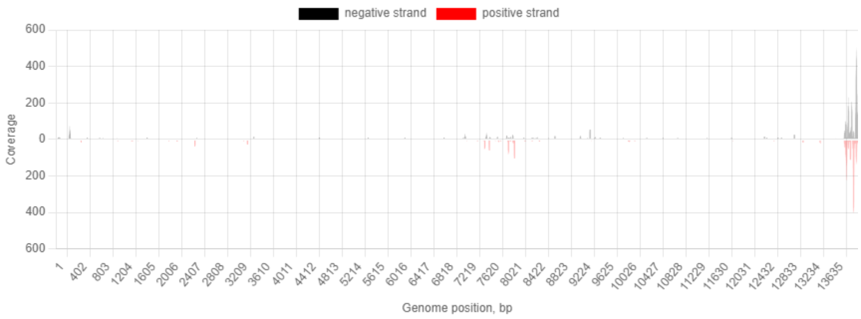

Mapping coverage of all reads matching StrV1_B (5882reads)

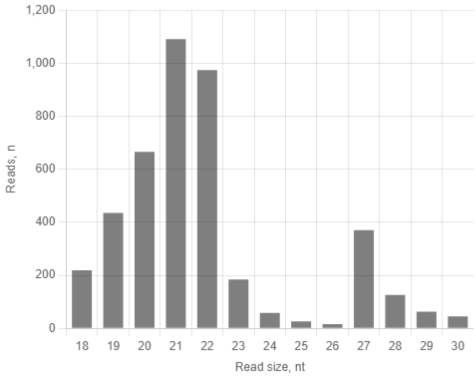

Length distribution of the reads

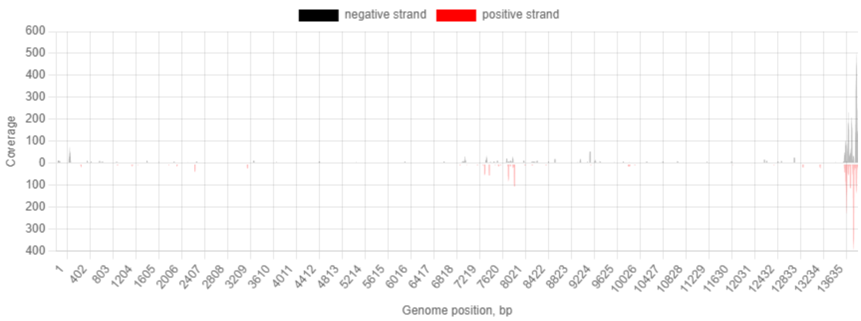

Mapping coverage of StrV1_B specific reads (5741reads)

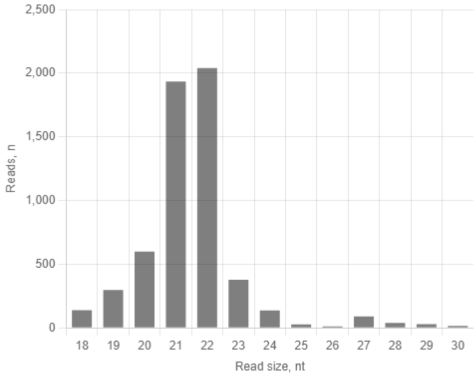

Length distribution of the reads

Dataset	Input reads	Virus	Reference	Mapped reads	Mapped reads, %	RPKM	Forward/Reverse balance	Nonredundant, %	Strain specific	Strain specific, % from total mapped reads	Strain specific, RPKM
1B_2	10000000	StrV1	StrV1_C	1285	0.013	9.0	1.1	46.1	763	59.4	7.1

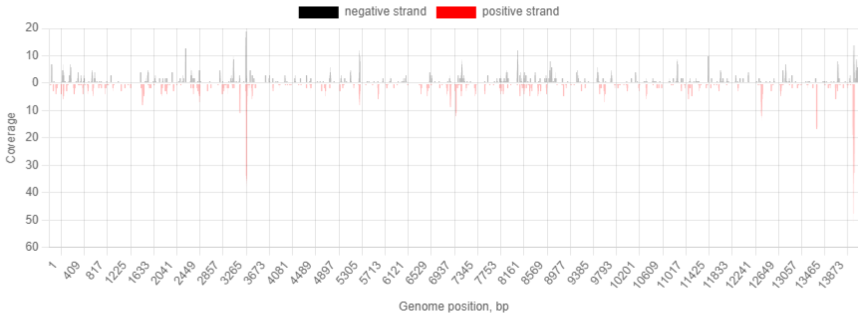

Mapping coverage of all reads matching StrV1_C (1158reads)

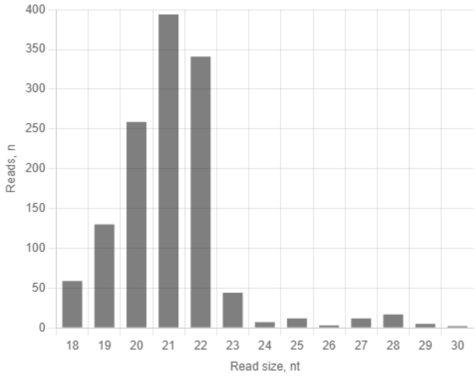

Length distribution of the reads

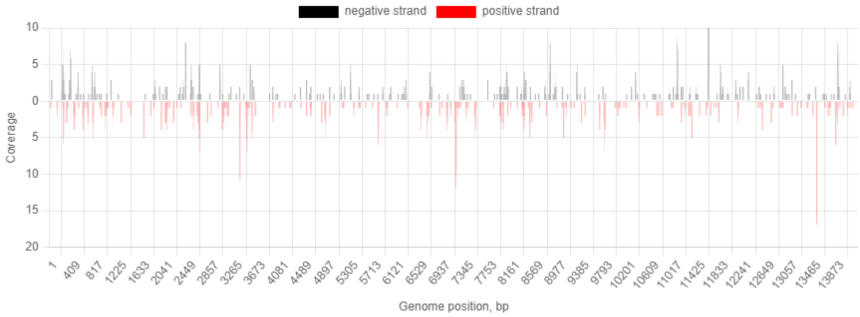

Mapping coverage of StrV1_C specific reads (763 reads)

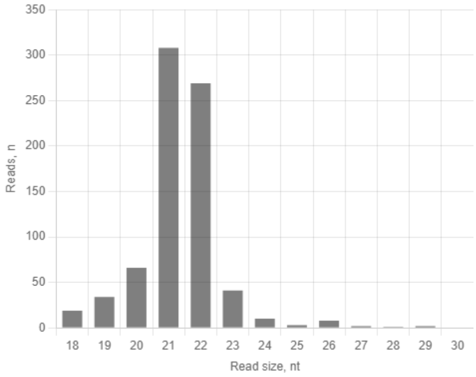

Length distribution of the reads

Mappings of the 1B_3 reads against the viral references.

Dataset	Input reads	Virus	Reference	Mapped reads	Mapped reads, %	RPKM	Forward/Reverse balance	Nonredundant, %	Strain specific	Strain specific, % from total mapped reads	Strain specific, RPKM
1B_3	10000000	SCV	SCV_A	2547	0.025	17.5	0.7	25.2	6504	255.4	15.4

Dataset	Input reads	Virus	Reference	Mapped reads	Mapped reads, %	RPKM	Forward/Reverse balance	Nonredundant, %	Strain specific	Strain specific, % from total mapped reads	Strain specific, RPKM

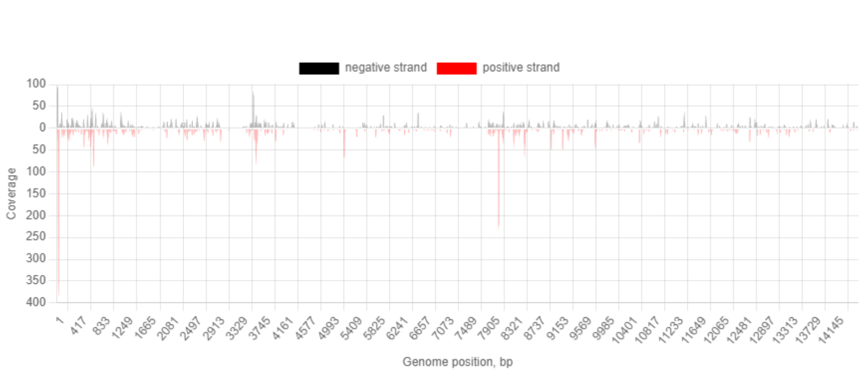

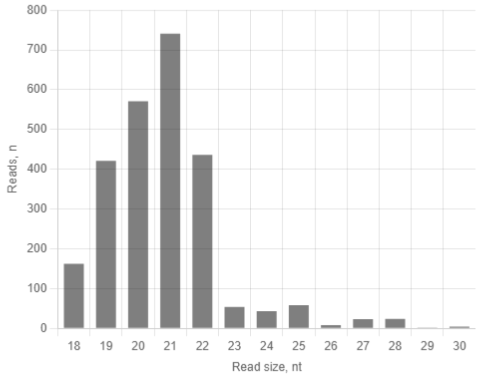

Mapping coverage of all reads matching SCV_A (7814reads)

Length distribution of the reads

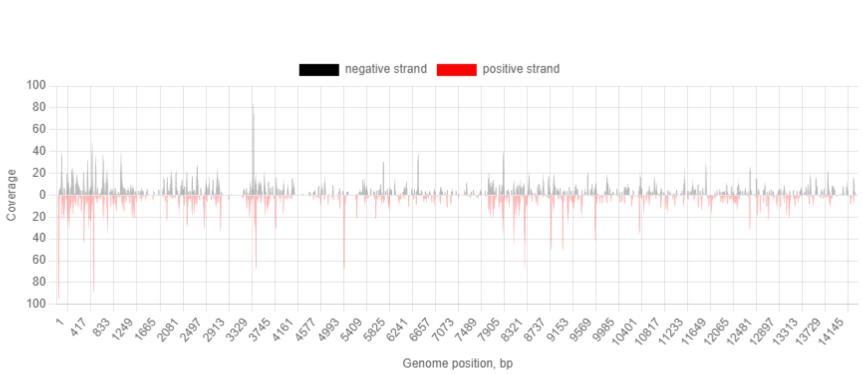

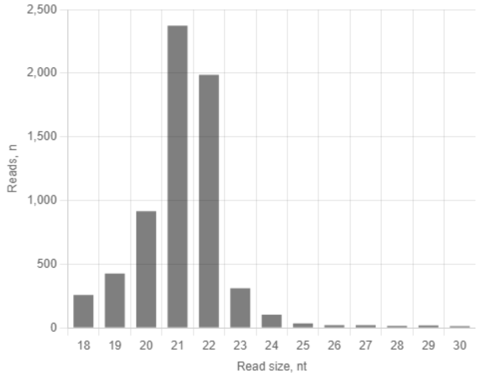

Mapping coverage of SCV_A specific reads (6504reads)

Length distribution of the reads

Dataset	Input reads	Virus	Reference	Mapped reads	Mapped reads, %	RPKM	Forward/Reverse balance	Nonredundant, %	Strain specific	Strain specific, % from total mapped reads	Strain specific, RPKM
1B_3	10000000	SCV	SCV_B	1365	0.014	9.4	0.7	25.9	6824	499.9	7.3

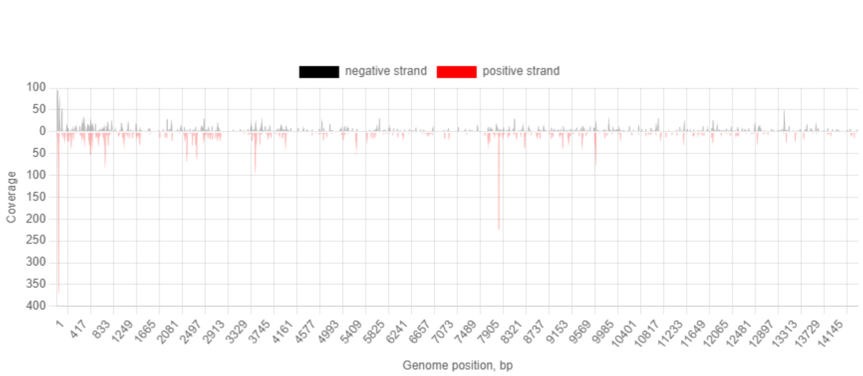

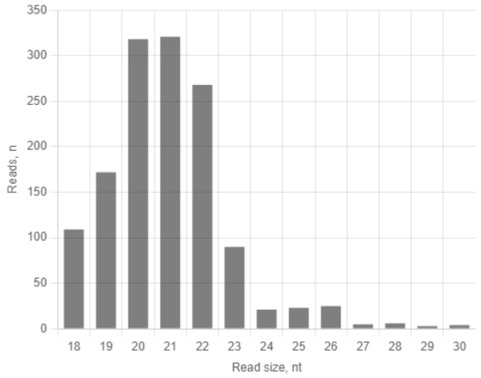

Mapping coverage of all reads matching SCV_B (8134reads)

Length distribution of the reads

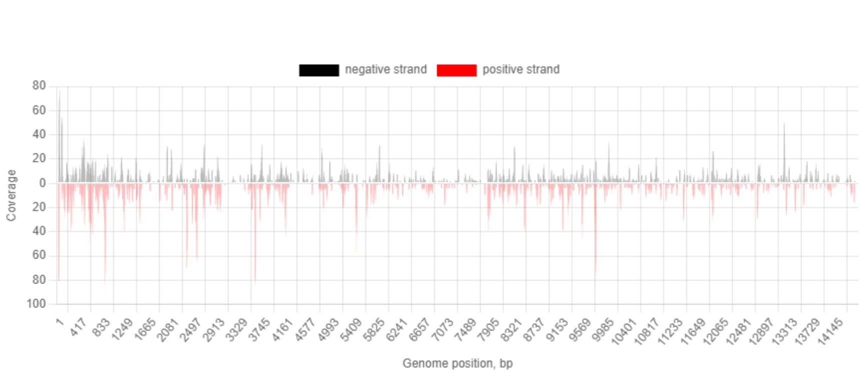

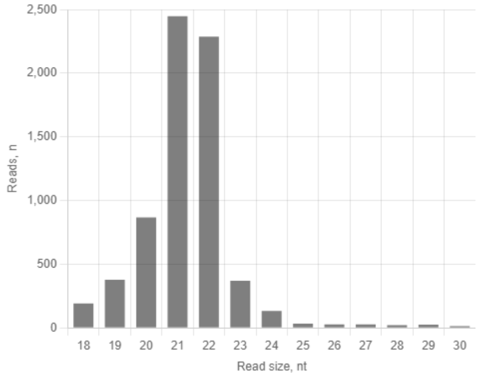

Mapping coverage of SCV_B specific reads (6824reads)

Length distribution of the reads

Dataset	Input reads	Virus	Reference	Mapped reads	Mapped reads, %	RPKM	Forward/Reverse balance	Nonredundant, %	Strain specific	Strain specific, % from total mapped reads	Strain specific, RPKM
1B_3	10000000	SMoV	SMoV_RNA1A	7320	0.073	104.0	2.1	19.1	5469	74.7	46.5

Dataset	Input reads	Virus	Reference	Mapped reads	Mapped reads, %	RPKM	Forward/Reverse balance	Nonredundant, %	Strain specific	Strain specific, % from total mapped reads	Strain specific, RPKM

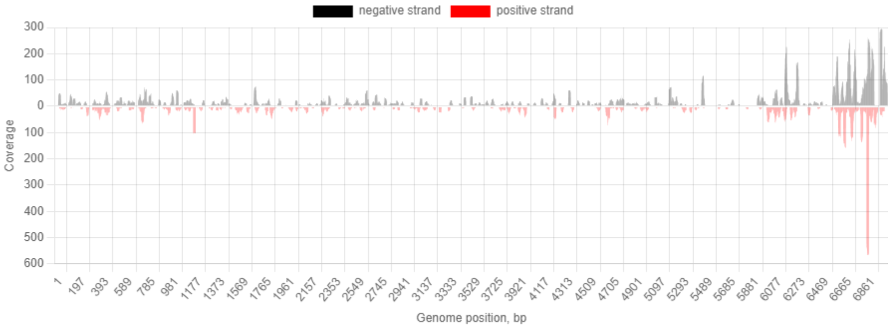

Mapping coverage of all reads matching SMoV_RNA1A (10871reads)

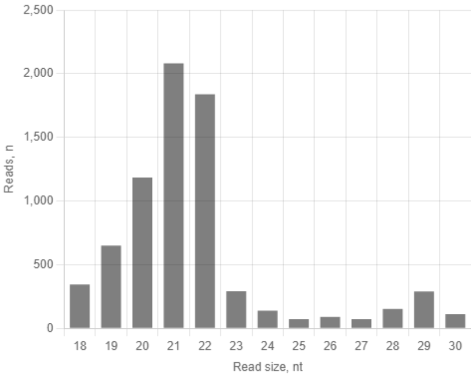

Length distribution of the reads

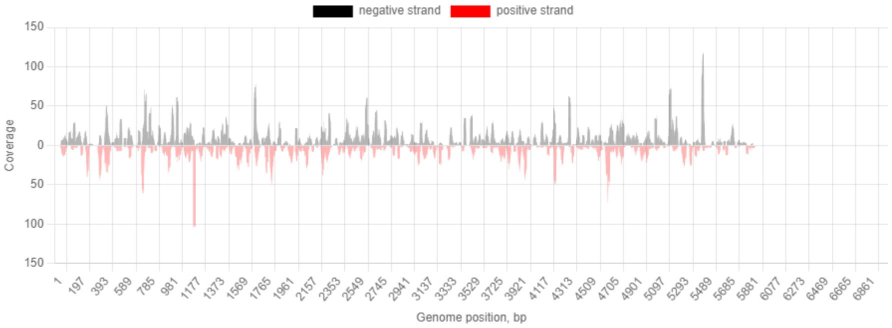

Mapping coverage of SMoV_RNA1A specific reads (5469reads)

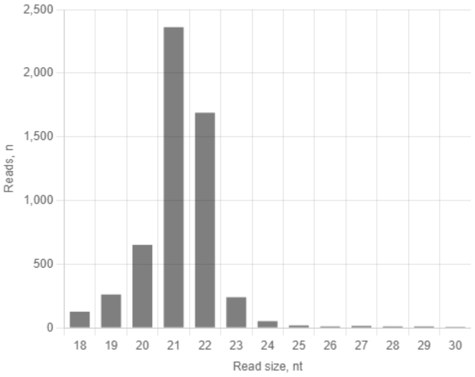

Length distribution of the reads

Dataset	Input reads	Virus	Reference	Mapped reads	Mapped reads, %	RPKM	Forward/Reverse balance	Nonredundant, %	Strain specific	Strain specific, % from total mapped reads	Strain specific, RPKM
1B_3	10000000	SMoV	SMoV_RNA1B	6864	0.069	97.3	1.7	19.7	4704	68.5	39.5

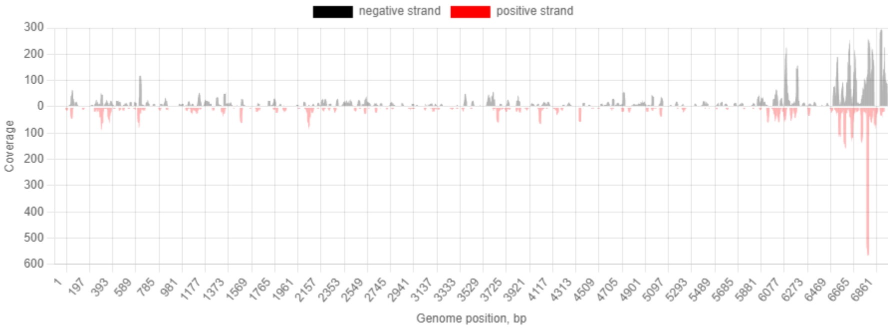

Mapping coverage of all reads matching SMoV_RNA1B (10125reads)

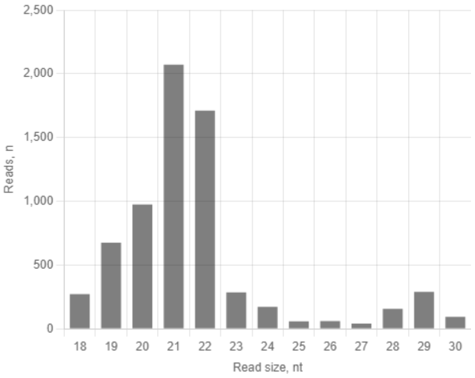

Length distribution of the reads

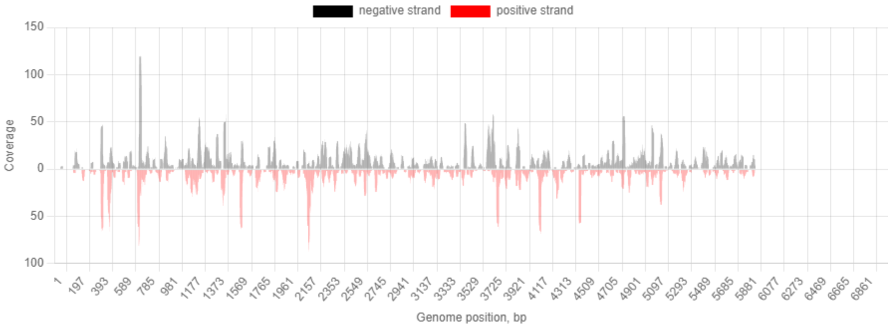

Mapping coverage of SMoV_RNA1B specific reads (4704reads)

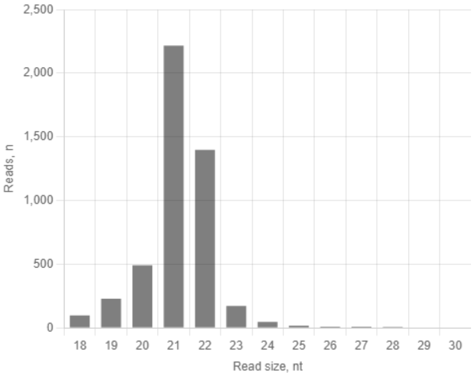

Length distribution of the reads

Dataset	Input reads	Virus	Reference	Mapped reads	Mapped reads, %	RPKM	Forward/Reverse balance	Nonredundant, %	Strain specific	Strain specific, % from total mapped reads	Strain specific, RPKM
1B_3	10000000	SMoV	SMoV_RNA1C	7087	0.071	100.6	1.7	19.1	5248	74.1	42.6

Dataset	Input reads	Virus	Reference	Mapped reads	Mapped reads, %	RPKM	Forward/Reverse balance	Nonredundant, %	Strain specific	Strain specific, % from total mapped reads	Strain specific, RPKM

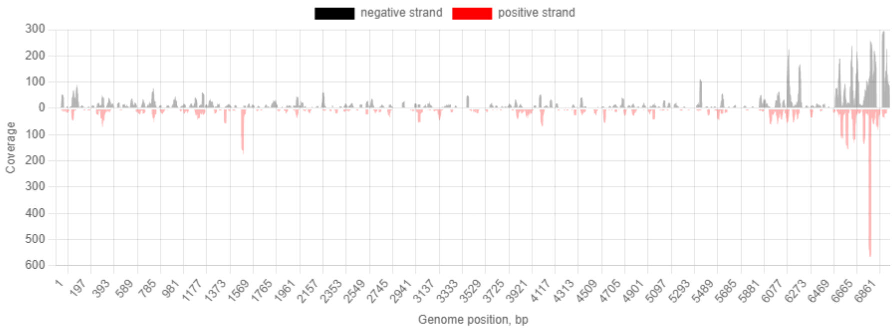

Mapping coverage of all reads matching SMoV_RNA1C (10639reads)

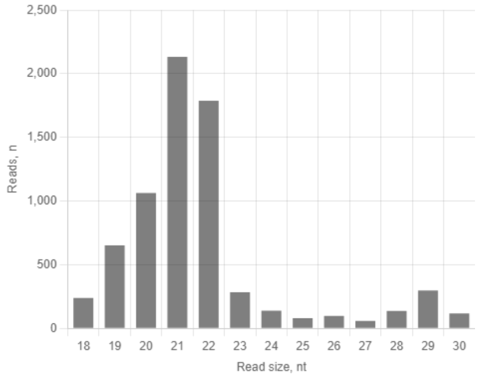

Length distribution of the reads

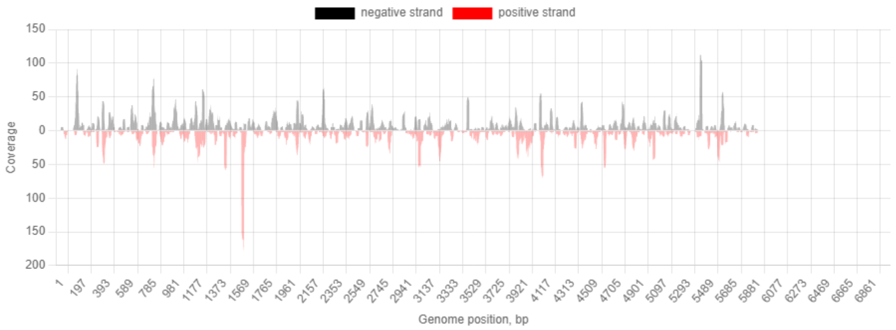

Mapping coverage of SMoV_RNA1C specific reads (5248reads)

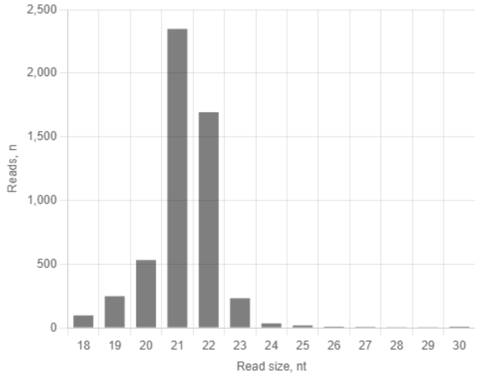

Length distribution of the reads

Dataset	Input reads	Virus	Reference	Mapped reads	Mapped reads, %	RPKM	Forward/Reverse balance	Nonredundant, %	Strain specific	Strain specific, % from total mapped reads	Strain specific, RPKM
1B_3	10000000	SMoV	SMoV_RNA2A	5056	0.051	80.7	1.9	21.3	4714	93.2	50.4

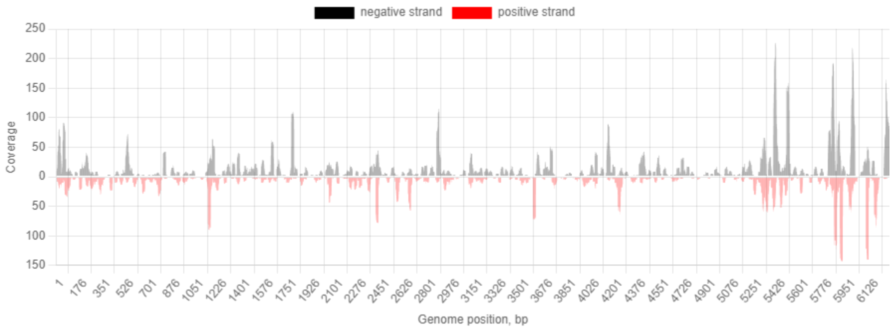

Mapping coverage of all reads matching SMoV_RNA2A (7149reads)

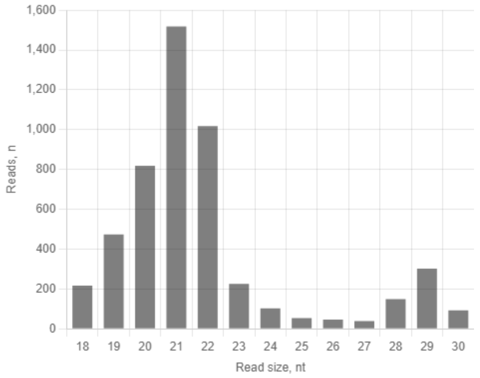

Length distribution of the reads

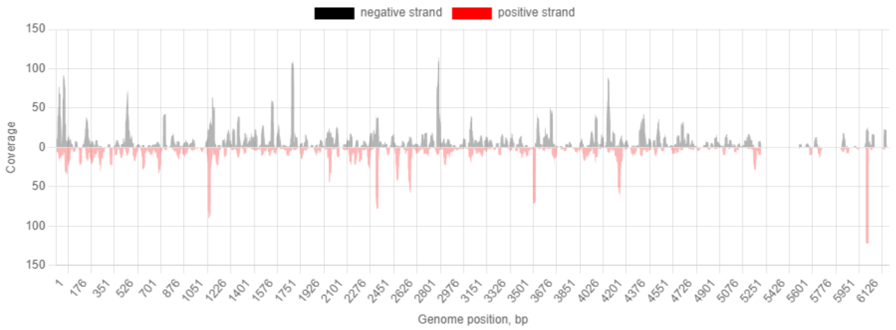

Mapping coverage of SMoV_RNA2A specific reads (4714reads)

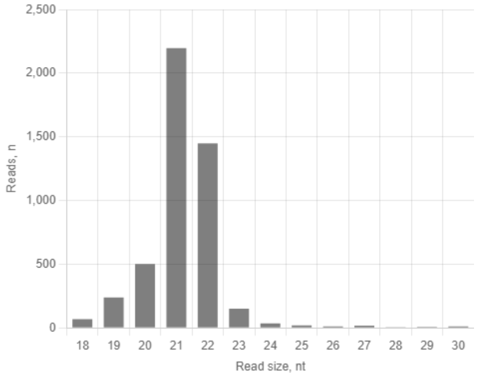

Length distribution of the reads

Dataset	Input reads	Virus	Reference	Mapped reads	Mapped reads, %	RPKM	Forward/Reverse balance	Nonredundant, %	Strain specific	Strain specific, % from total mapped reads	Strain specific, RPKM
1B_3	10000000	SMoV	SMoV_RNA2B	4160	0.042	65.8	2.5	20.4	3027	72.8	25.6

Dataset	Input reads	Virus	Reference	Mapped reads	Mapped reads, %	RPKM	Forward/Reverse balance	Nonredundant, %	Strain specific	Strain specific, % from total mapped reads	Strain specific, RPKM

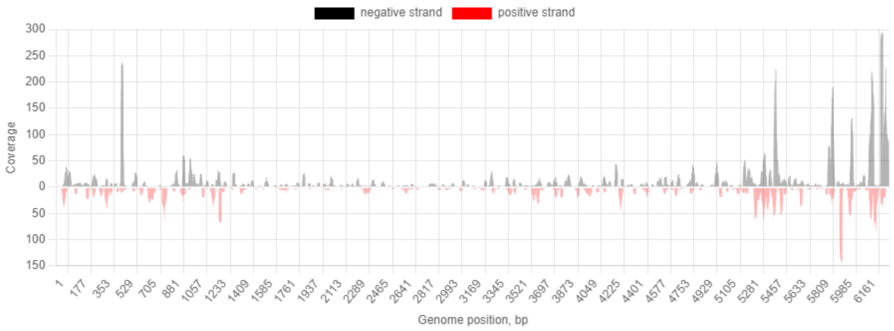

Mapping coverage of all reads matching SMoV_RNA2B (6150reads)

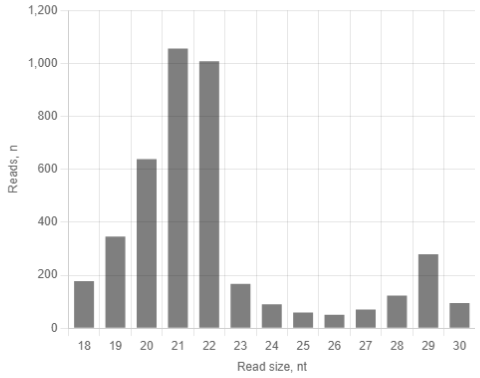

Length distribution of the reads

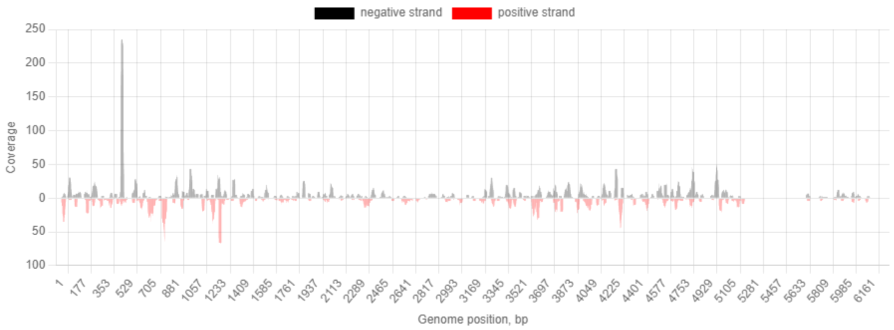

Mapping coverage of SMoV_RNA2B specific reads (3027reads)

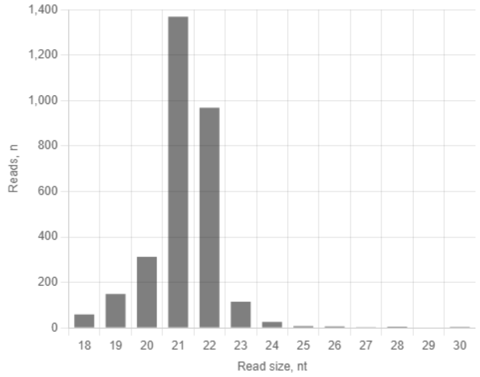

Length distribution of the reads

Dataset	Input reads	Virus	Reference	Mapped reads	Mapped reads, %	RPKM	Forward/Reverse balance	Nonredundant, %	Strain specific	Strain specific, % from total mapped reads	Strain specific, RPKM
1B_3	10000000	SMoV	SMoV_RNA2C	5989	0.060	94.5	1.9	18.9	4485	74.9	33.2

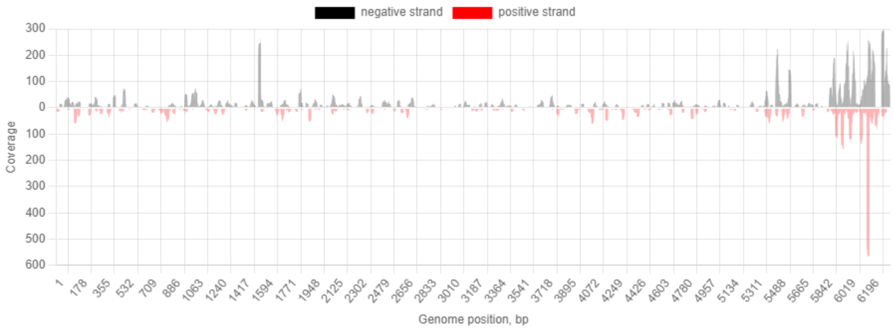

Mapping coverage of all reads matching SMoV_RNA2C (9549reads)

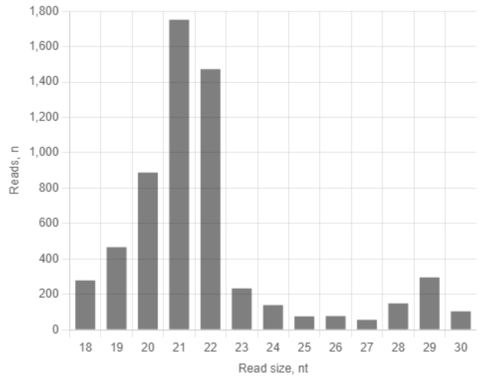

Length distribution of the reads

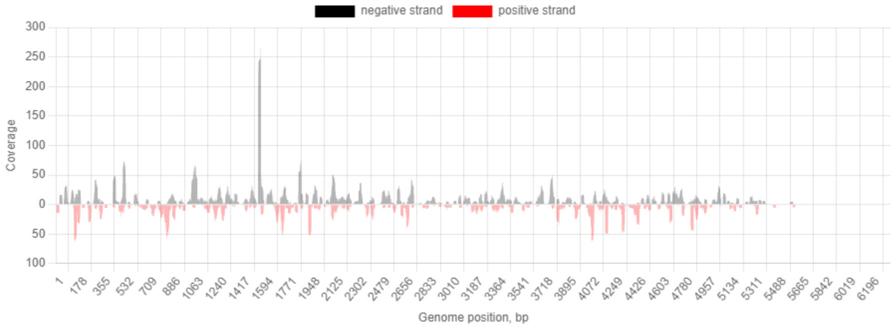

Mapping coverage of SMoV_RNA2C specific reads (4485reads)

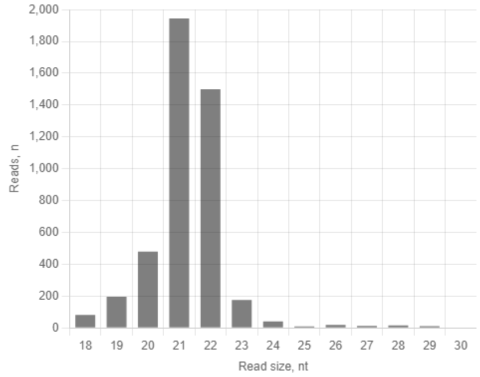

Length distribution of the reads

Dataset	Input reads	Virus	Reference	Mapped reads	Mapped reads, %	RPKM	Forward/Reverse balance	Nonredundant, %	Strain specific	Strain specific, % from total mapped reads	Strain specific, RPKM
1B_3	10000000	StrV1	StrV1_A	1141	0.011	8.1	0.8	30.0	2267	198.7	6.4

Dataset	Input reads	Virus	Reference	Mapped reads	Mapped reads, %	RPKM	Forward/Reverse balance	Nonredundant, %	Strain specific	Strain specific, % from total mapped reads	Strain specific, RPKM

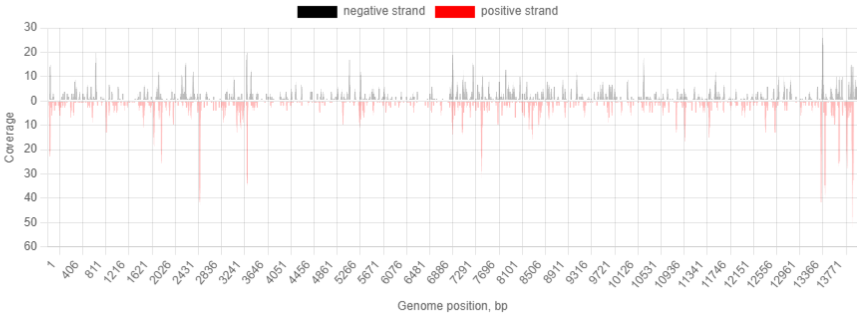

Mapping coverage of all reads matching StrV1_A (2694 reads)

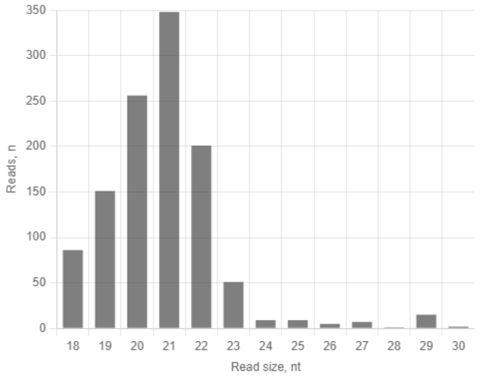

Length distribution of the reads

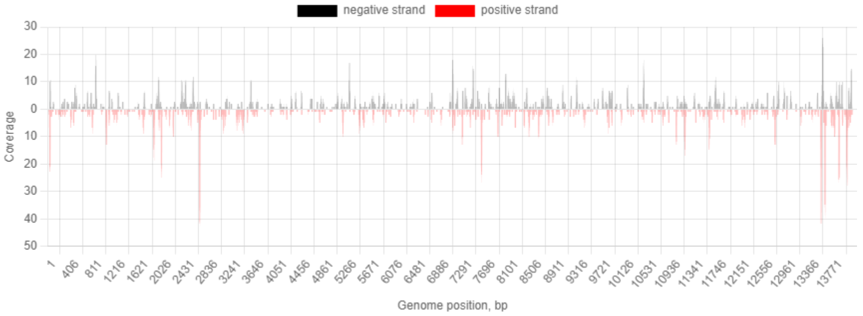

Mapping coverage of StrV1_A specific reads (2267 reads)

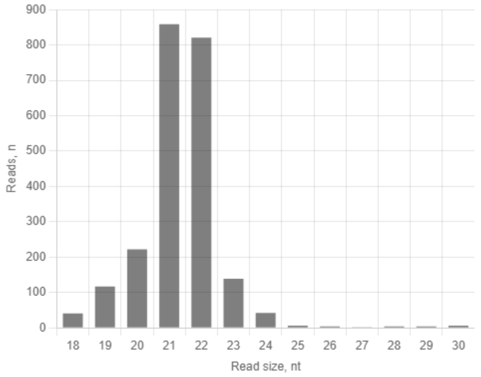

Length distribution of the reads

Dataset	Input reads	Virus	Reference	Mapped reads	Mapped reads, %	RPKM	Forward/Reverse balance	Nonredundant, %	Strain specific	Strain specific, % from total mapped reads	Strain specific, RPKM
1B_3	10000000	StrV1	StrV1_B	4198	0.042	29.9	1.4	17.6	5741	136.8	29.3

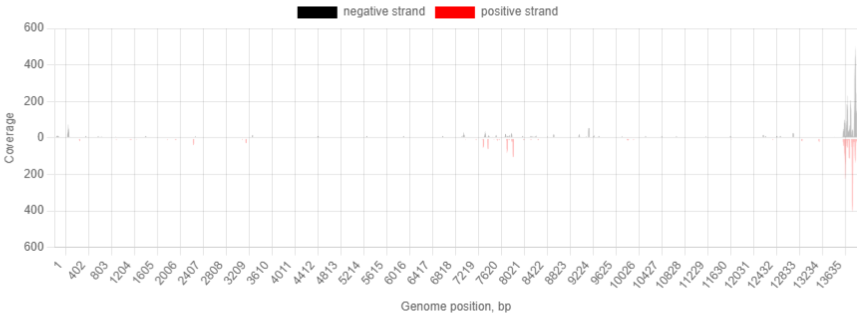

Mapping coverage of all reads matching StrV1_B (5882 reads)

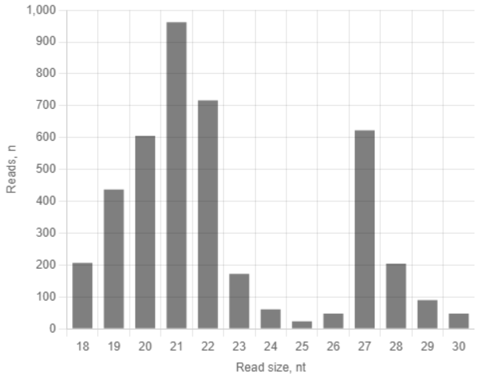

Length distribution of the reads

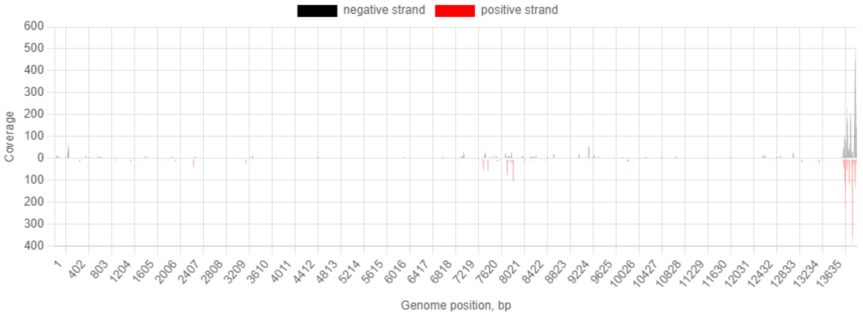

Mapping coverage of StrV1_B specific reads (5741 reads)

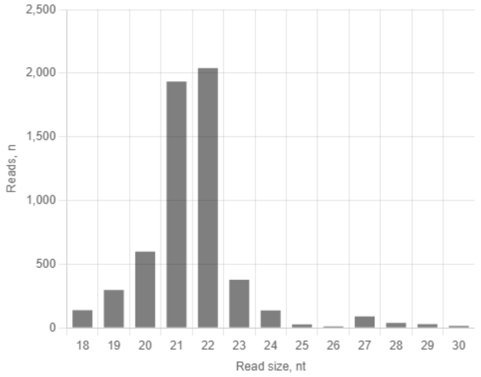

Length distribution of the reads

Dataset	Input reads	Virus	Reference	Mapped reads	Mapped reads, %	RPKM	Forward/Reverse balance	Nonredundant, %	Strain specific	Strain specific, % from total mapped reads	Strain specific, RPKM
1B_3	10000000	StrV1	StrV1_C	664	0.007	4.7	0.7	29.7	763	114.9	3.0

Dataset	Input reads	Virus	Reference	Mapped reads	Mapped reads, %	RPKM	Forward/Reverse balance	Nonredundant, %	Strain specific	Strain specific, % from total mapped reads	Strain specific, RPKM

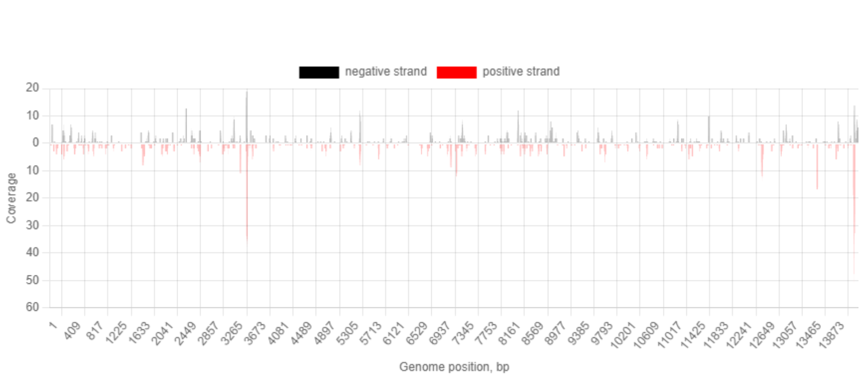

Mapping coverage of all reads matching StrV1_C (1158reads)

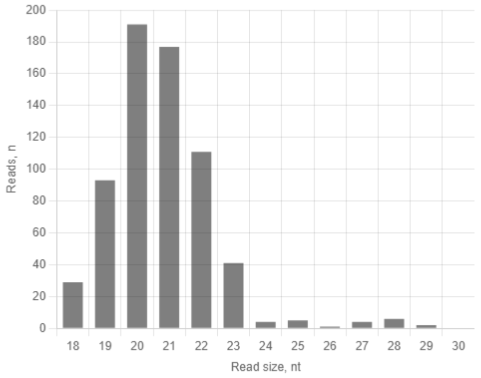

Length distribution of the reads

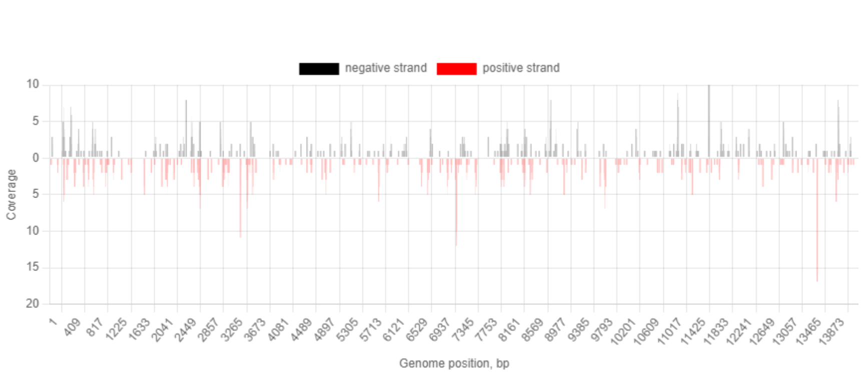

Mapping coverage of StrV1_C specific reads (763reads)

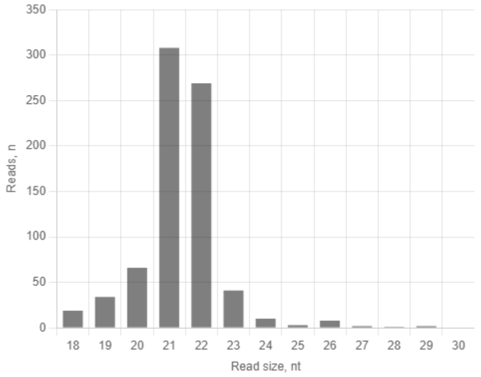

Length distribution of the reads

Mappings of the 1B_4 reads against the viral references.

Dataset	Input reads	Virus	Reference	Mapped reads	Mapped reads, %	RPKM	Forward/Reverse balance	Nonredundant, %	Strain specific	Strain specific, % from total mapped reads	Strain specific, RPKM
1B_4	10000000	SCV	SCV_A	7011	0.070	48.2	0.9	24.0	6504	92.8	42.9

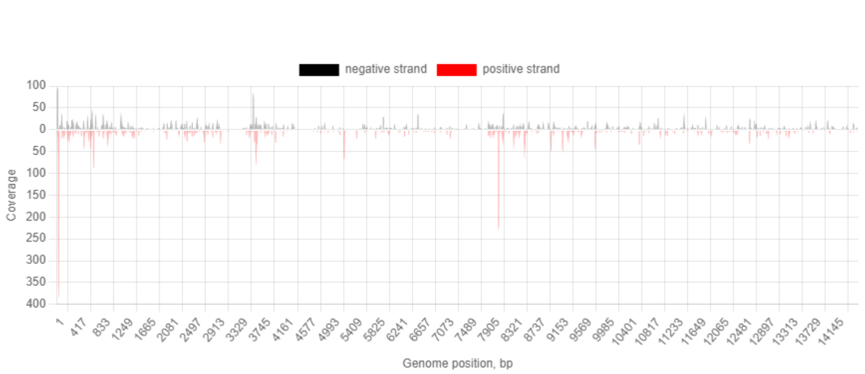

Mapping coverage of all reads matching SCV_A (7814reads)

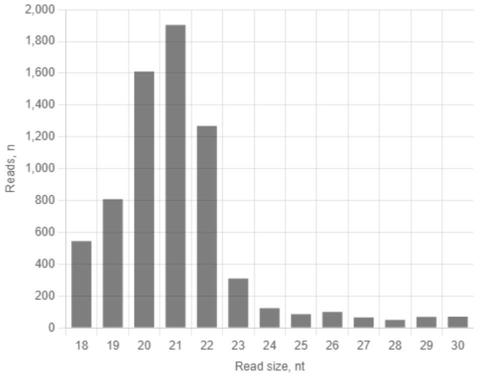

Length distribution of the reads

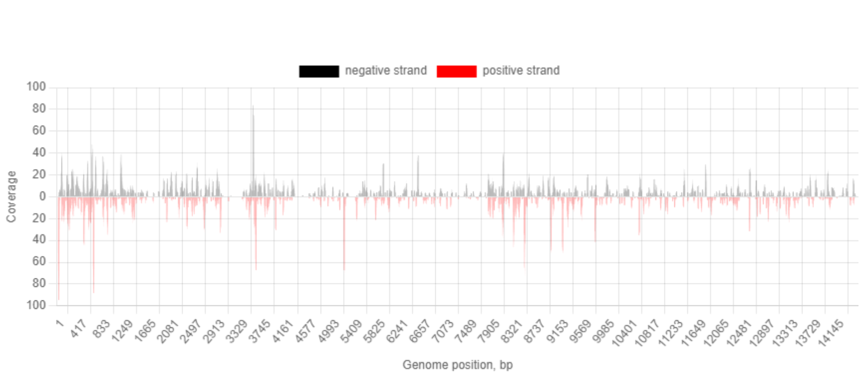

Mapping coverage of SCV_A specific reads (6504reads)

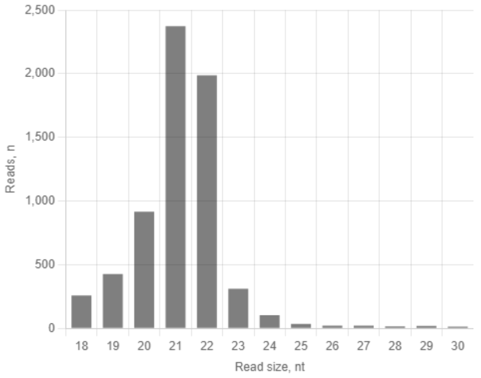

Length distribution of the reads

Dataset	Input reads	Virus	Reference	Mapped reads	Mapped reads, %	RPKM	Forward/Reverse balance	Nonredundant, %	Strain specific	Strain specific, % from total mapped reads	Strain specific, RPKM
1B_4	10000000	SCV	SCV_B	3462	0.035	23.8	0.8	25.8	6824	197.1	18.5

Dataset	Input reads	Virus	Reference	Mapped reads	Mapped reads, %	RPKM	Forward/Reverse balance	Nonredundant, %	Strain specific	Strain specific, % from total mapped reads	Strain specific, RPKM

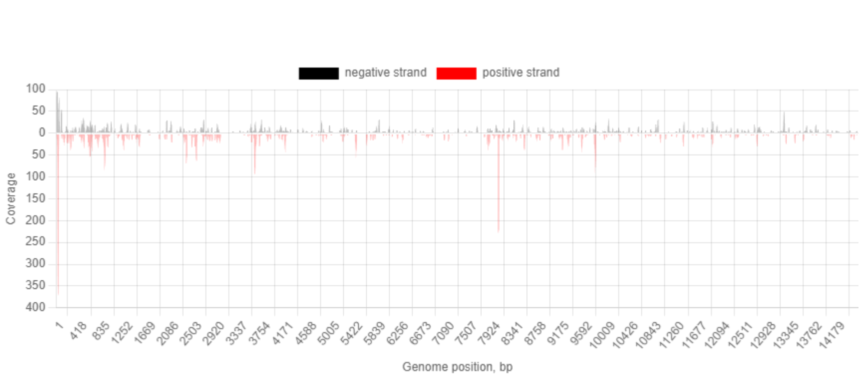

Mapping coverage of all reads matching SCV_B (8134reads)

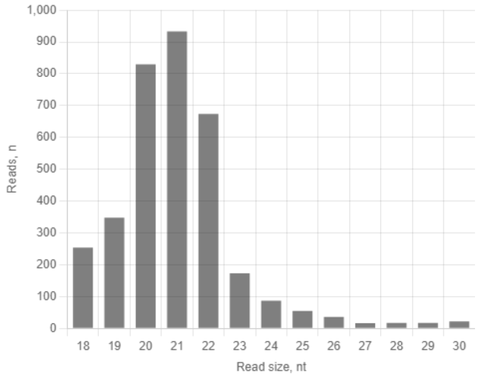

Length distribution of the reads

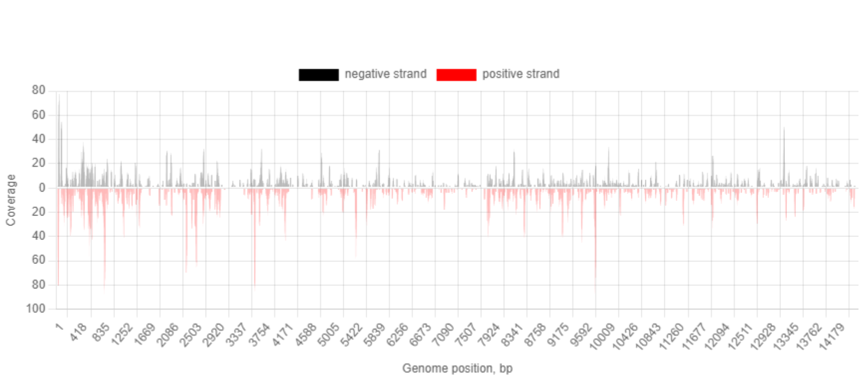

Mapping coverage of SCV_B specific reads (6824reads)

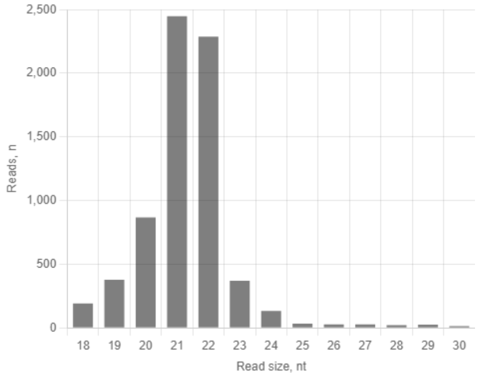

Length distribution of the reads

Dataset	Input reads	Virus	Reference	Mapped reads	Mapped reads, %	RPKM	Forward/Reverse balance	Nonredundant, %	Strain specific	Strain specific, % from total mapped reads	Strain specific, RPKM
1B_4	10000000	SMoV	SMoV_RNA1A	7216	0.072	102.5	1.9	19.3	5469	75.8	48.4

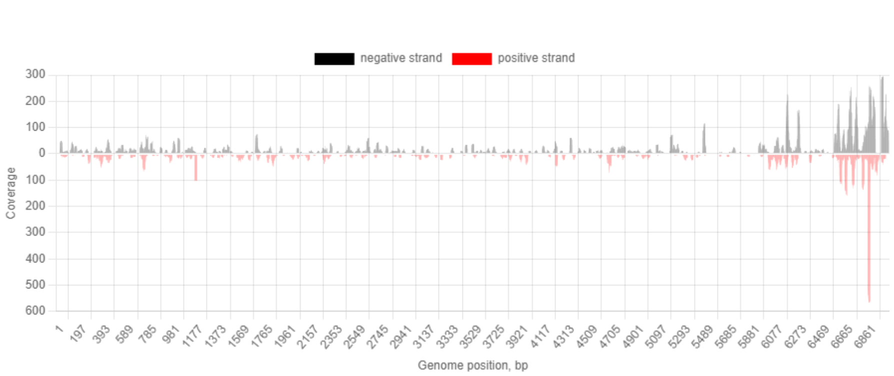

Mapping coverage of all reads matching SMoV_RNA1A (10871reads)

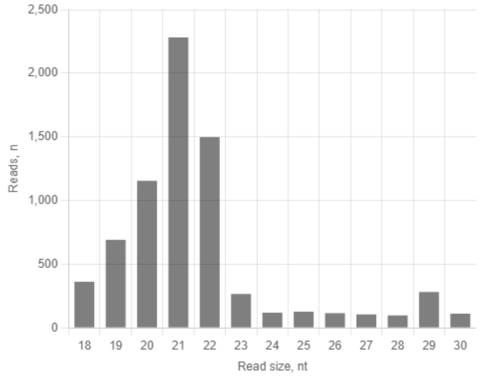

Length distribution of the reads

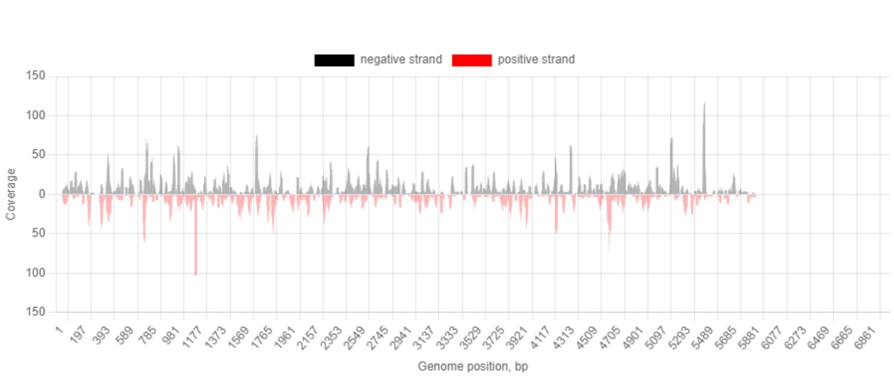

Mapping coverage of SMoV_RNA1A specific reads (5469reads)

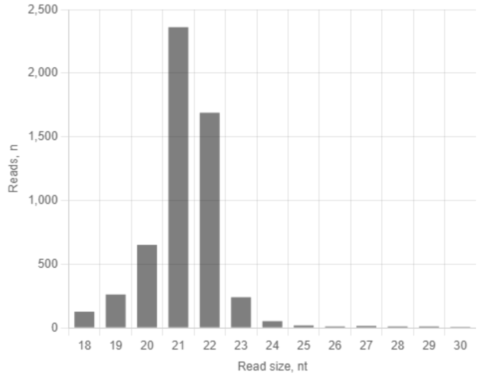

Length distribution of the reads

Dataset	Input reads	Virus	Reference	Mapped reads	Mapped reads, %	RPKM	Forward/Reverse balance	Nonredundant, %	Strain specific	Strain specific, % from total mapped reads	Strain specific, RPKM
1B_4	10000000	SMoV	SMoV_RNA1B	6561	0.066	93.0	1.7	19.2	4704	71.7	38.9

Dataset	Input reads	Virus	Reference	Mapped reads	Mapped reads, %	RPKM	Forward/Reverse balance	Nonredundant, %	Strain specific	Strain specific, % from total mapped reads	Strain specific, RPKM

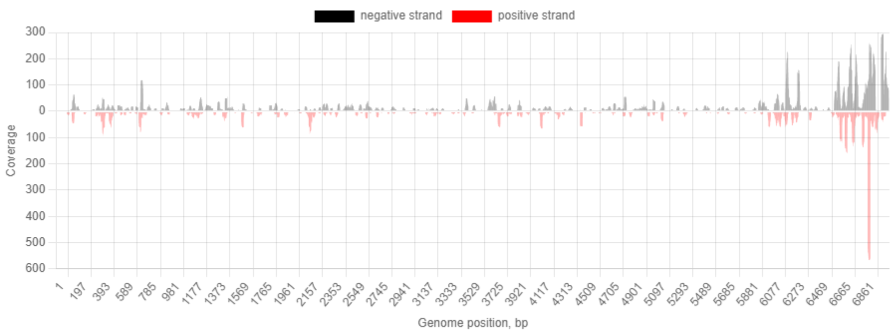

Mapping coverage of all reads matching SMoV_RNA1B (10125reads)

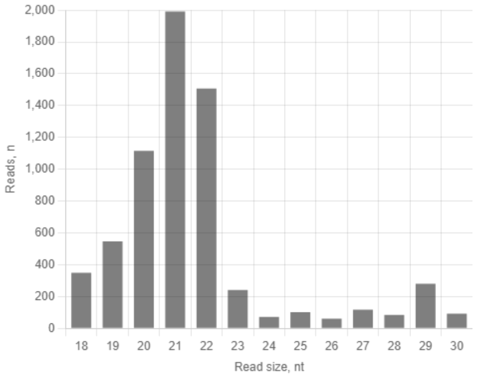

Length distribution of the reads

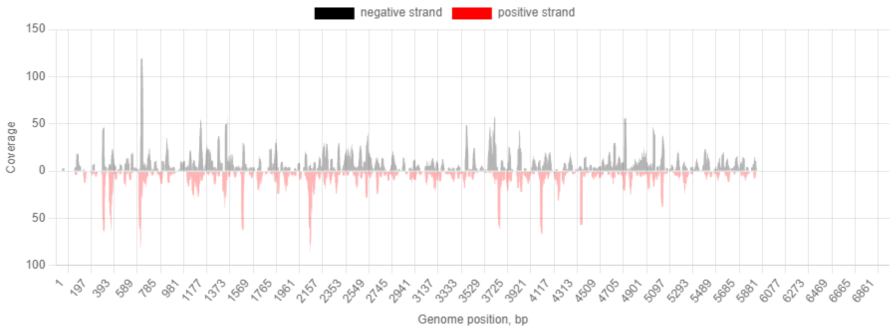

Mapping coverage of SMoV_RNA1B specific reads (4704reads)

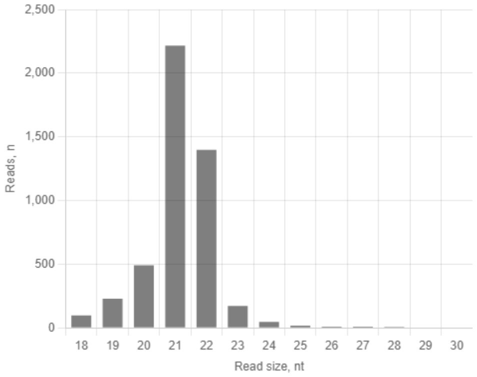

Length distribution of the reads

Dataset	Input reads	Virus	Reference	Mapped reads	Mapped reads, %	RPKM	Forward/Reverse balance	Nonredundant, %	Strain specific	Strain specific, % from total mapped reads	Strain specific, RPKM
1B_4	10000000	SMoV	SMoV_RNA1C	6708	0.067	95.2	1.7	19.9	5248	78.2	39.5

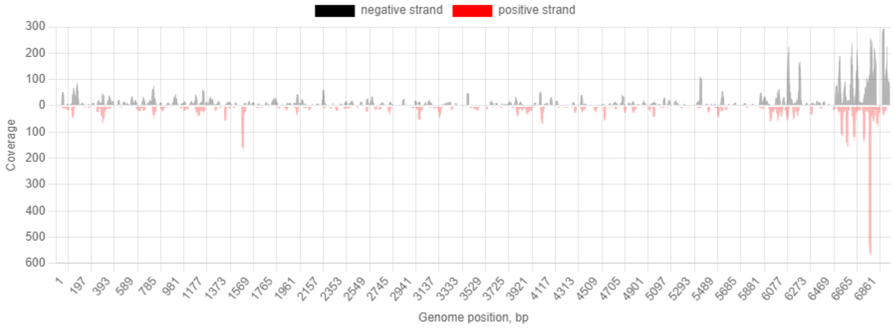

Mapping coverage of all reads matching SMoV_RNA1C (10639reads)

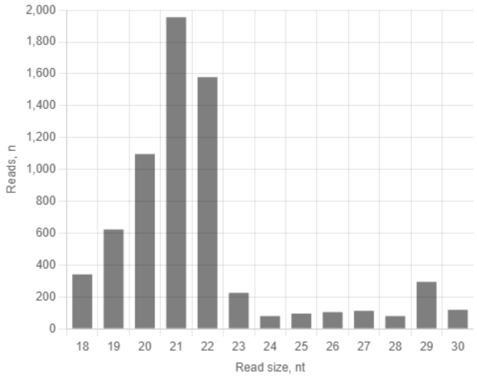

Length distribution of the reads

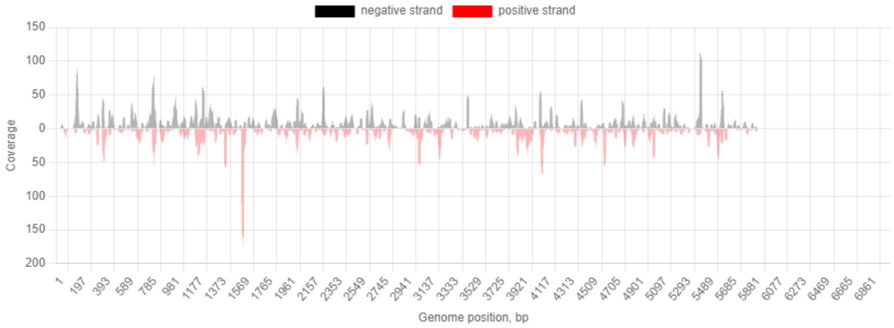

Mapping coverage of SMoV_RNA1C specific reads (5248reads)

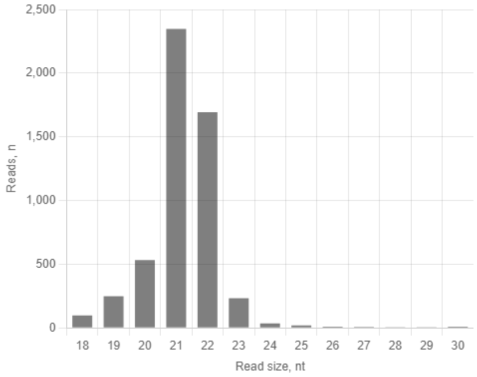

Length distribution of the reads

Dataset	Input reads	Virus	Reference	Mapped reads	Mapped reads, %	RPKM	Forward/Reverse balance	Nonredundant, %	Strain specific	Strain specific, % from total mapped reads	Strain specific, RPKM
1B_4	10000000	SMoV	SMoV_RNA2A	4865	0.049	77.6	1.8	19.8	4714	96.9	45.9

Dataset	Input reads	Virus	Reference	Mapped reads	Mapped reads, %	RPKM	Forward/Reverse balance	Nonredundant, %	Strain specific	Strain specific, % from total mapped reads	Strain specific, RPKM

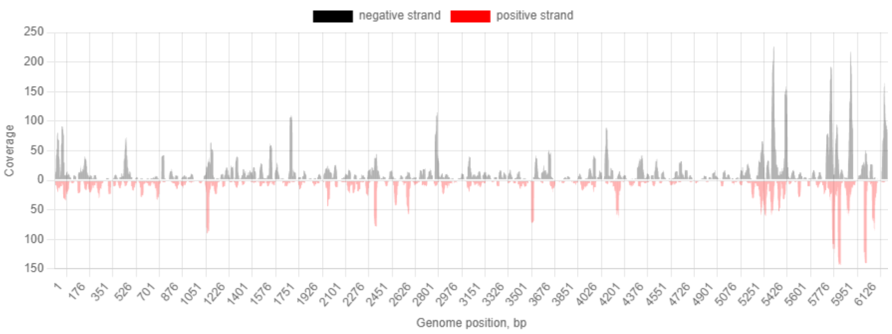

Mapping coverage of all reads matching SMoV_RNA2A (7149reads)

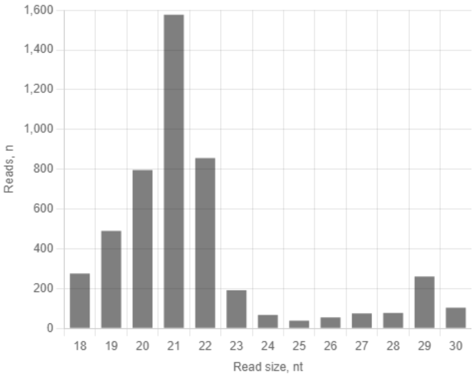

Length distribution of the reads

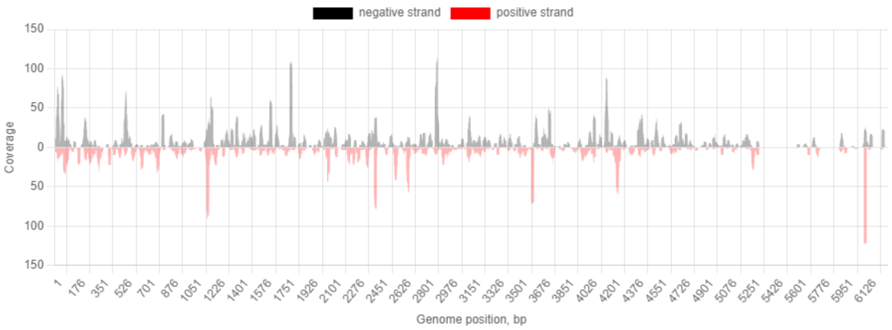

Mapping coverage of SMoV_RNA2A specific reads (4714reads)

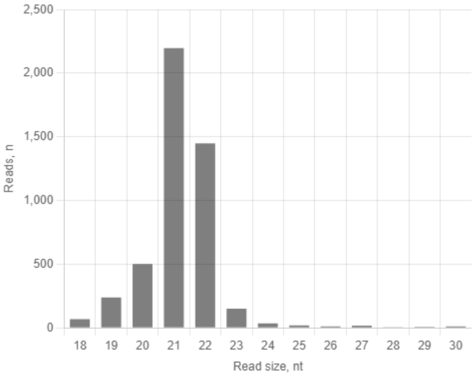

Length distribution of the reads

Dataset	Input reads	Virus	Reference	Mapped reads	Mapped reads, %	RPKM	Forward/Reverse balance	Nonredundant, %	Strain specific	Strain specific, % from total mapped reads	Strain specific, RPKM
1B_4	10000000	SMoV	SMoV_RNA2B	4184	0.042	66.1	2.1	21.3	3027	72.3	29.6

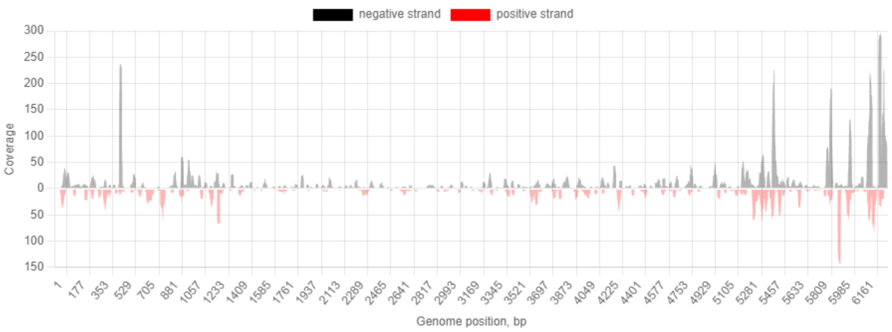

Mapping coverage of all reads matching SMoV_RNA2B (6150reads)

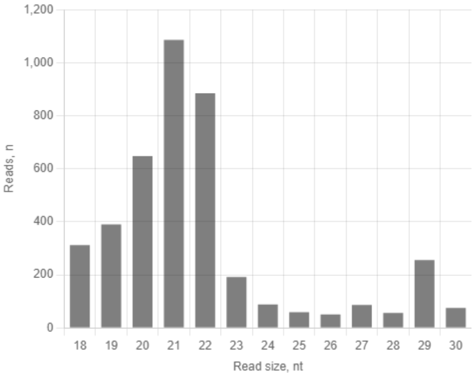

Length distribution of the reads

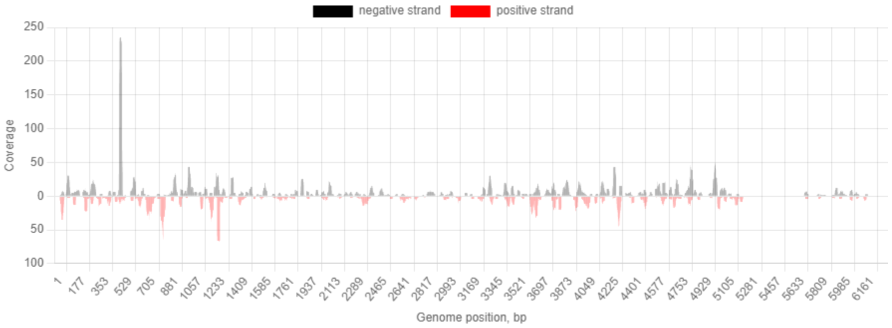

Mapping coverage of SMoV_RNA2B specific reads (3027reads)

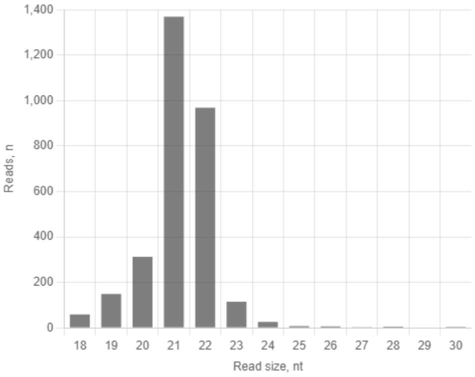

Length distribution of the reads

Dataset	Input reads	Virus	Reference	Mapped reads	Mapped reads, %	RPKM	Forward/Reverse balance	Nonredundant, %	Strain specific	Strain specific, % from total mapped reads	Strain specific, RPKM
1B_4	10000000	SMoV	SMoV_RNA2C	6262	0.063	98.8	1.7	18.4	4485	71.6	40.4

Dataset	Input reads	Virus	Reference	Mapped reads	Mapped reads, %	RPKM	Forward/Reverse balance	Nonredundant, %	Strain specific	Strain specific, % from total mapped reads	Strain specific, RPKM

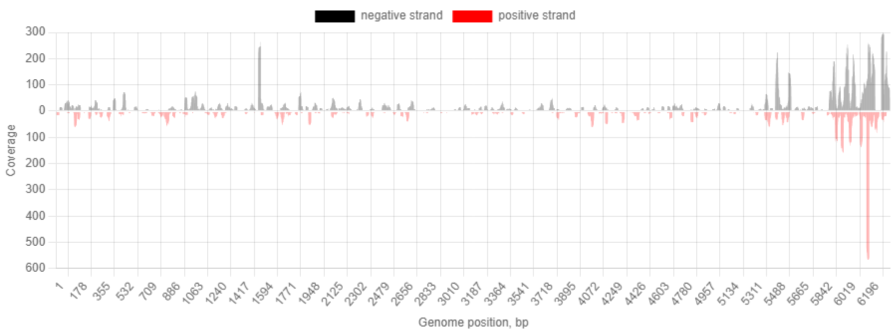

Mapping coverage of all reads matching SMoV_RNA2C (9549reads)

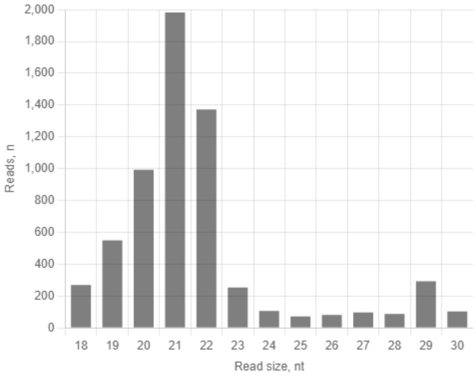

Length distribution of the reads

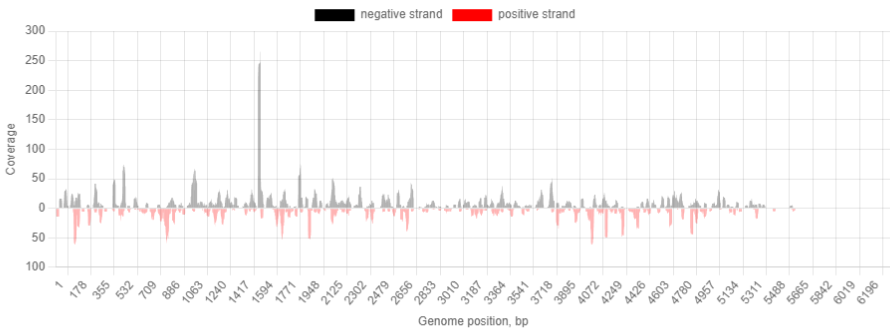

Mapping coverage of SMoV_RNA2C specific reads (4485reads)

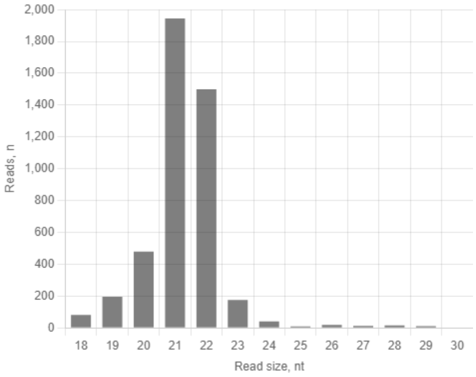

Length distribution of the reads

Dataset	Input reads	Virus	Reference	Mapped reads	Mapped reads, %	RPKM	Forward/Reverse balance	Nonredundant, %	Strain specific	Strain specific, % from total mapped reads	Strain specific, RPKM
1B_4	10000000	StrV1	StrV1_A	1376	0.014	9.7	0.9	25.2	2267	164.8	7.7

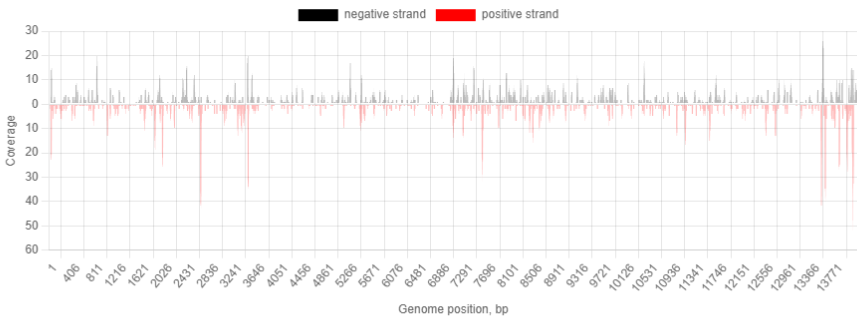

Mapping coverage of all reads matching StrV1_A (2694reads)

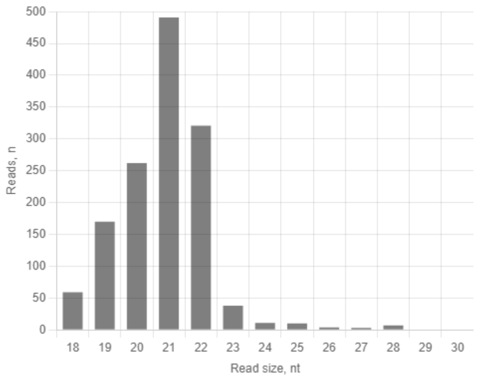

Length distribution of the reads

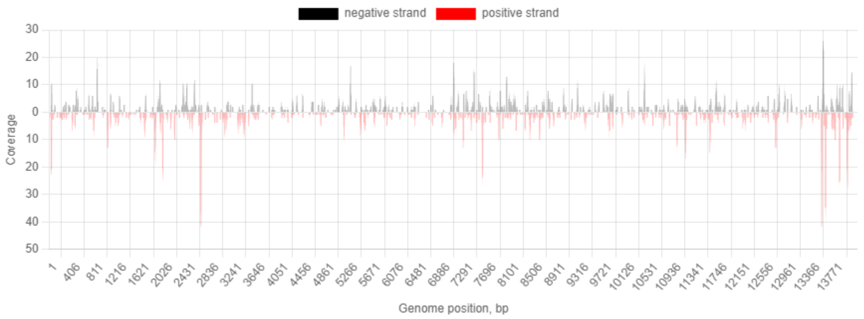

Mapping coverage of StrV1_A specific reads (2267reads)

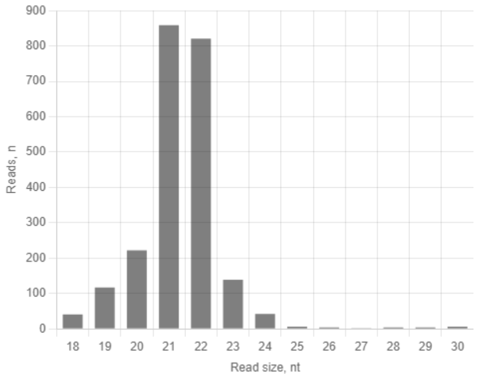

Length distribution of the reads

Dataset	Input reads	Virus	Reference	Mapped reads	Mapped reads, %	RPKM	Forward/Reverse balance	Nonredundant, %	Strain specific	Strain specific, % from total mapped reads	Strain specific, RPKM
1B_4	10000000	StrV1	StrV1_B	3560	0.036	25.4	1.5	21.1	5741	161.3	24.7

Dataset	Input reads	Virus	Reference	Mapped reads	Mapped reads, %	RPKM	Forward/Reverse balance	Nonredundant, %	Strain specific	Strain specific, % from total mapped reads	Strain specific, RPKM

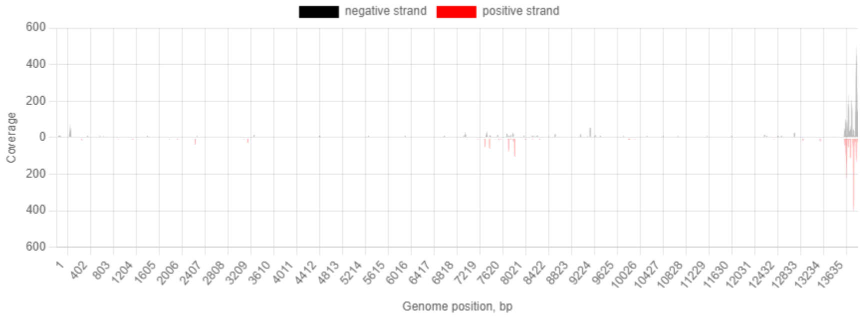

Mapping coverage of all reads matching StrV1_B (5882reads)

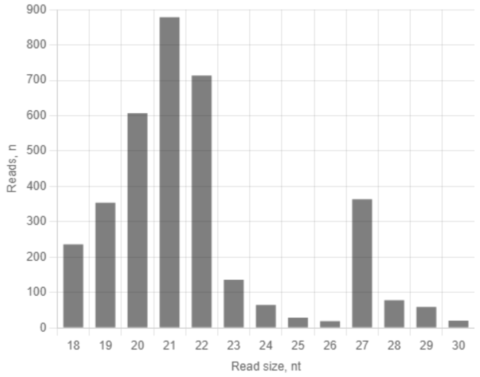

Length distribution of the reads

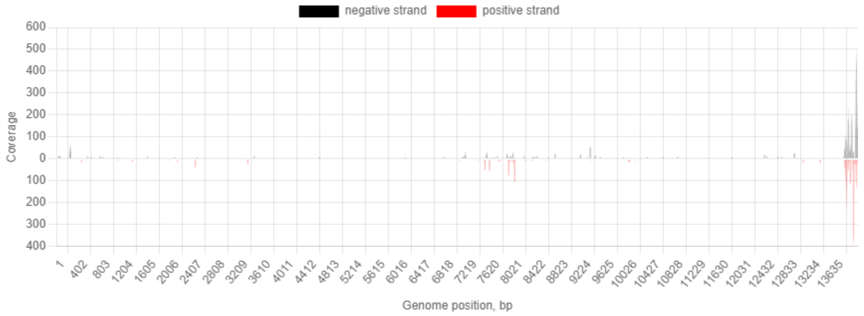

Mapping coverage of StrV1_B specific reads (5741reads)

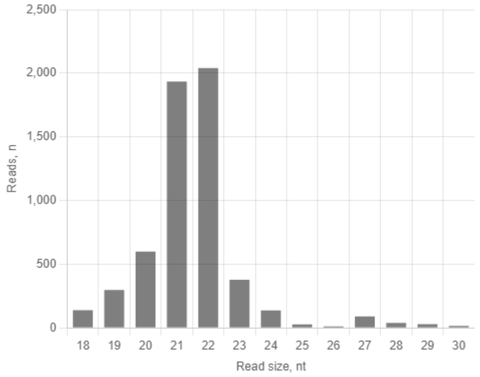

Length distribution of the reads

Dataset	Input reads	Virus	Reference	Mapped reads	Mapped reads, %	RPKM	Forward/Reverse balance	Nonredundant, %	Strain specific	Strain specific, % from total mapped reads	Strain specific, RPKM
1B_4	10000000	StrV1	StrV1_C	1107	0.011	7.8	1.0	26.6	763	68.9	5.8

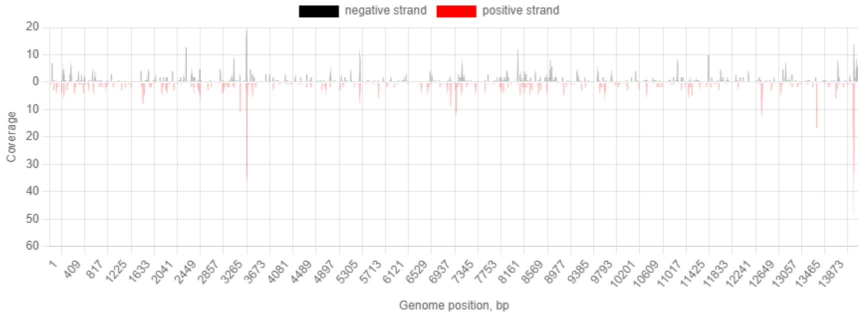

Mapping coverage of all reads matching StrV1_C (1158reads)

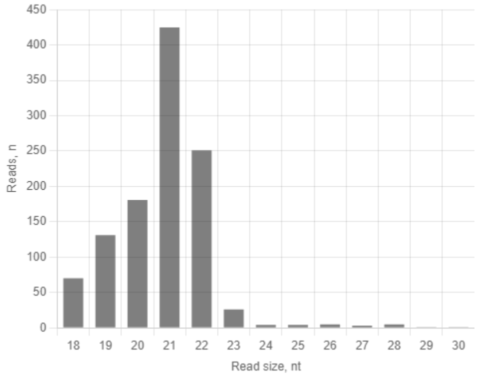

Length distribution of the reads

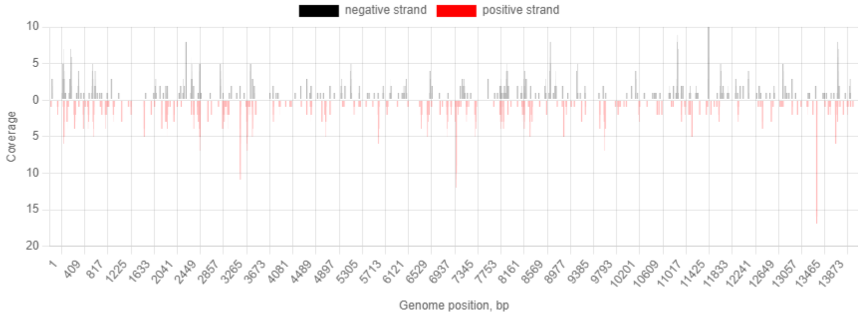

Mapping coverage of StrV1_C specific reads (763 reads)

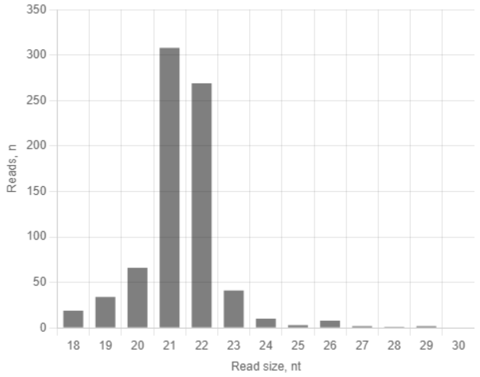

Length distribution of the reads

Mappings of the 2A_2 reads against the viral references.

Dataset	Input reads	Virus	Reference	Mapped reads	Mapped reads, %	RPKM	Forward/Reverse balance	Nonredundant, %	Strain specific	Strain specific, % from total mapped reads	Strain specific, RPKM
2A_2	10000000	SCV	SCV_A	13189	0.132	90.7	0.8	38.4	11245	85.3	77.3

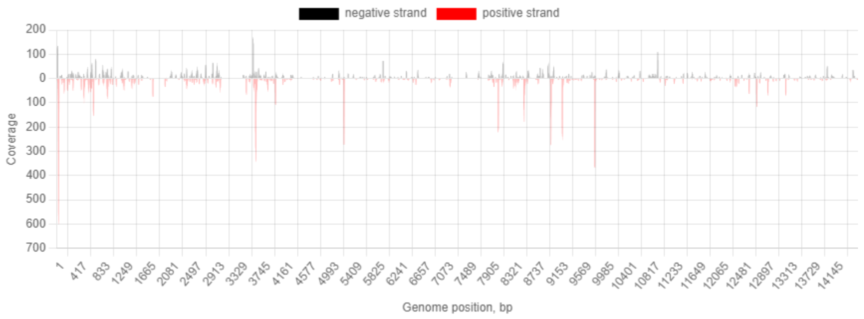

Mapping coverage of **all reads** matching SCV_A (13189 reads)

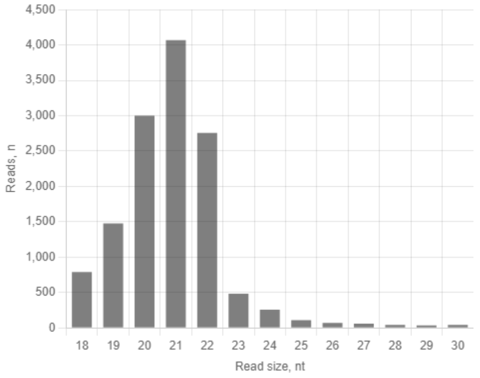

Length distribution of the reads

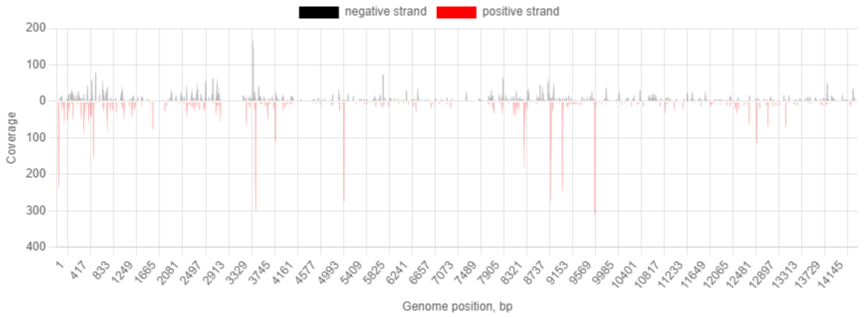

Mapping coverage of SCV_A **specific reads** (11245 reads)

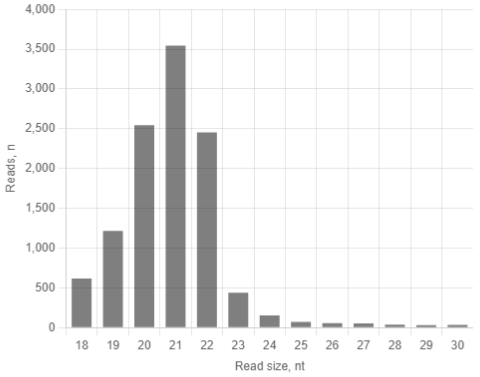

Length distribution of the reads

Dataset	Input reads	Virus	Reference	Mapped reads	Mapped reads, %	RPKM	Forward/Reverse balance	Nonredundant, %	Strain specific	Strain specific, % from total mapped reads	Strain specific, RPKM
2A_2	10000000	SCV	SCV_B	10814	0.108	74.3	0.8	43.3	8870	82.0	60.9

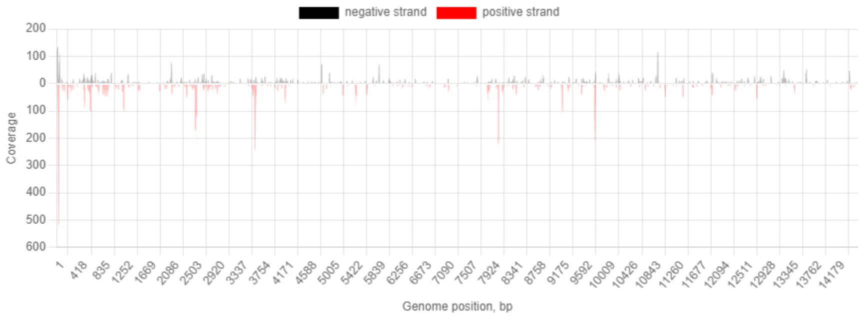

Mapping coverage of **all reads** matching SCV_B (10814 reads)

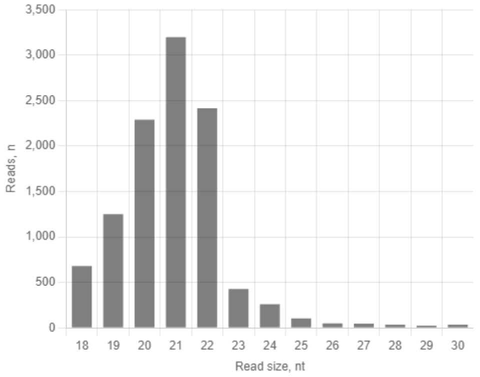

Length distribution of the reads

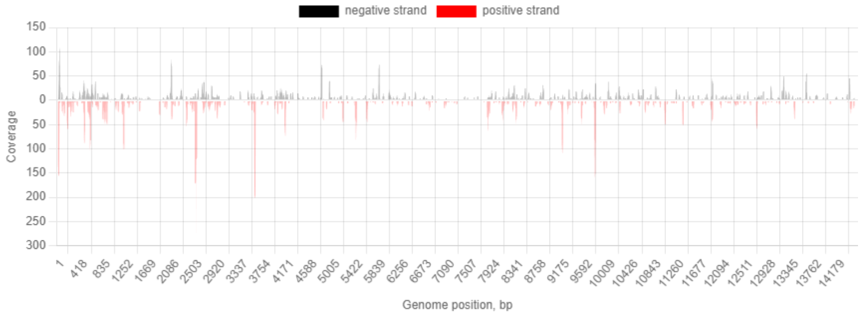

Mapping coverage of SCV_B **specific reads** (8870 reads)

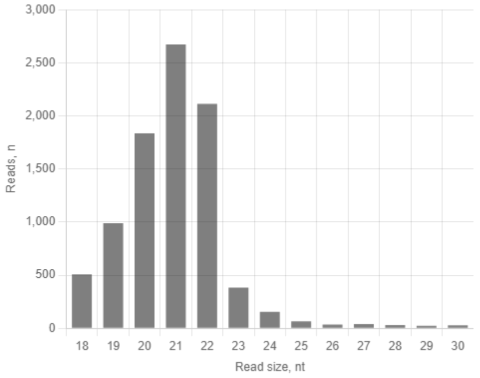

Length distribution of the reads

Dataset	Input reads	Virus	Reference	Mapped reads	Mapped reads, %	RPKM	Forward/Reverse balance	Nonredundant, %	Strain specific	Strain specific, % from total mapped reads	Strain specific, RPKM
2A_2	10000000	SMoV	SMoV_RNA1A	7992	0.080	113.6	1.5	37.2	4257	53.3	60.5

Dataset	Input reads	Virus	Reference	Mapped reads	Mapped reads, %	RPKM	Forward/Reverse balance	Nonredundant, %	Strain specific	Strain specific, % from total mapped reads	Strain specific, RPKM

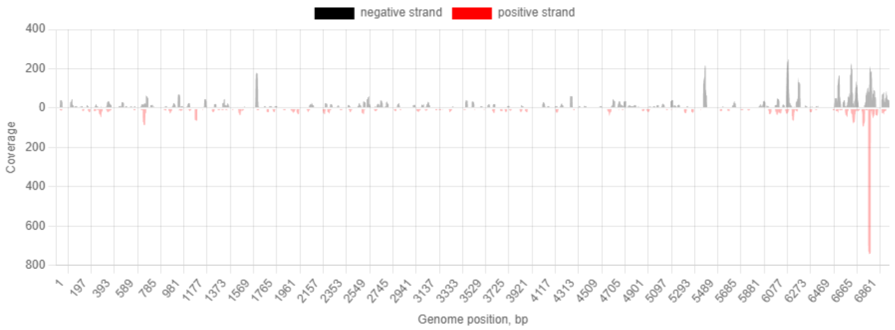

Mapping coverage of all reads matching SMoV_RNA1A (7992 reads)

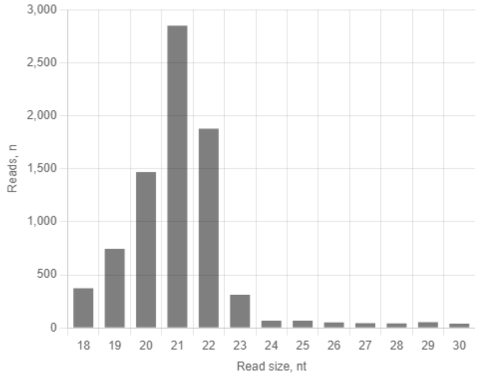

Length distribution of the reads

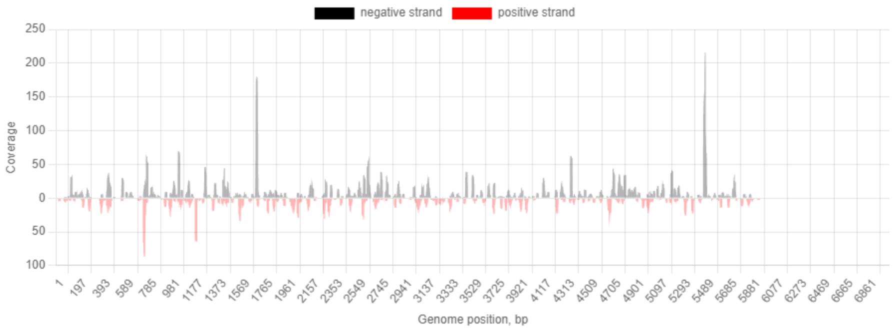

Mapping coverage of SMoV_RNA1A specific reads (4257 reads)

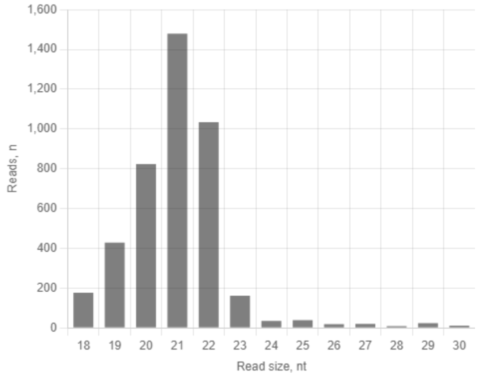

Length distribution of the reads

Dataset	Input reads	Virus	Reference	Mapped reads	Mapped reads, %	RPKM	Forward/Reverse balance	Nonredundant, %	Strain specific	Strain specific, % from total mapped reads	Strain specific, RPKM
2A_2	10000000	SMoV	SMoV_RNA1B	7155	0.072	101.5	1.1	37.9	3443	48.1	48.8

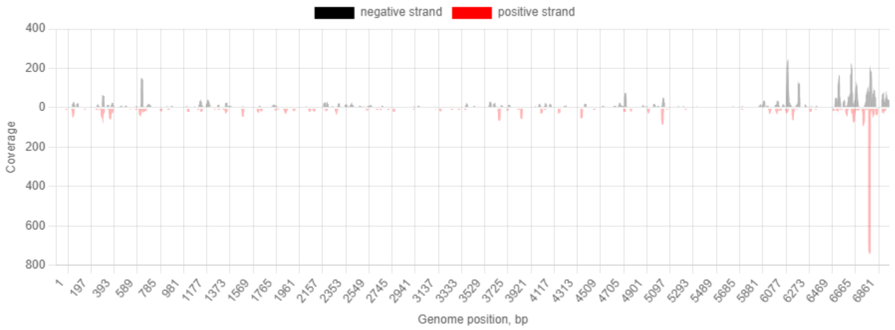

Mapping coverage of all reads matching SMoV_RNA1B (7155 reads)

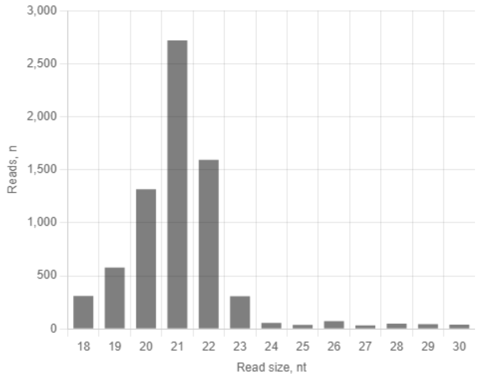

Length distribution of the reads

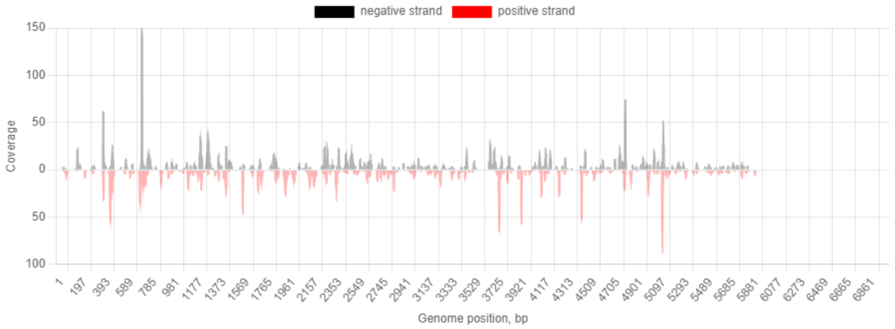

Mapping coverage of SMoV_RNA1B specific reads (3443 reads)

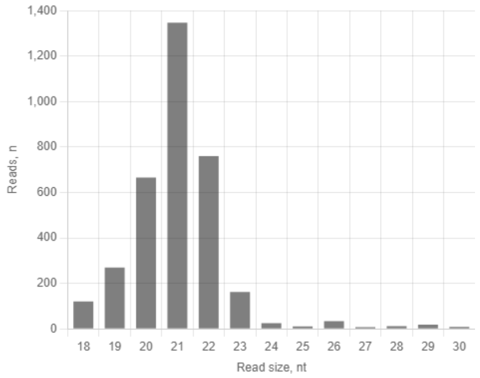

Length distribution of the reads

Dataset	Input reads	Virus	Reference	Mapped reads	Mapped reads, %	RPKM	Forward/Reverse balance	Nonredundant, %	Strain specific	Strain specific, % from total mapped reads	Strain specific, RPKM
2A_2	10000000	SMoV	SMoV_RNA1C	7767	0.078	110.3	1.3	38.7	4015	51.7	57.0

Dataset	Input reads	Virus	Reference	Mapped reads	Mapped reads, %	RPKM	Forward/Reverse balance	Nonredundant, %	Strain specific	Strain specific, % from total mapped reads	Strain specific, RPKM

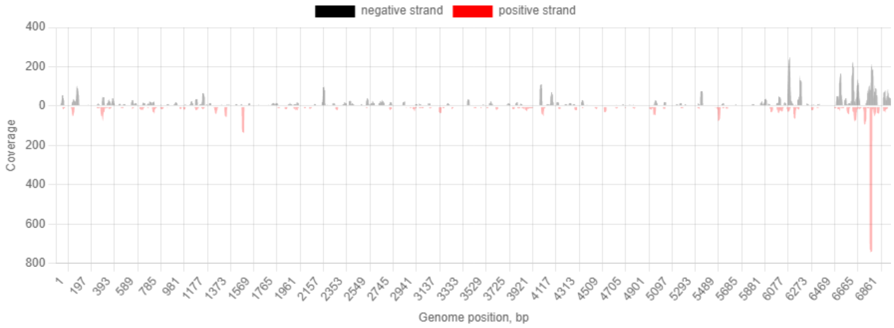

Mapping coverage of all reads matching SMoV_RNA1C (7767 reads)

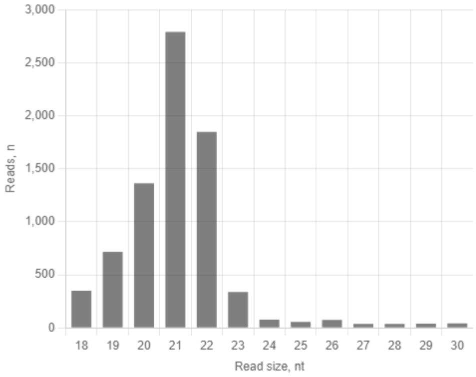

Length distribution of the reads

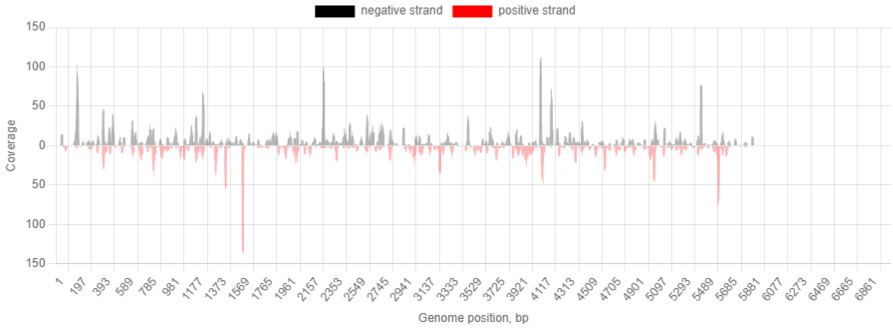

Mapping coverage of SMoV_RNA1C specific reads (4015 reads)

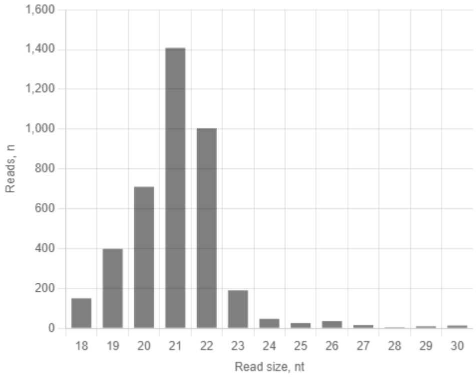

Length distribution of the reads

Dataset	Input reads	Virus	Reference	Mapped reads	Mapped reads, %	RPKM	Forward/Reverse balance	Nonredundant, %	Strain specific	Strain specific, % from total mapped reads	Strain specific, RPKM
2A_2	10000000	SMoV	SMoV_RNA2A	4719	0.047	75.3	1.5	42.2	3172	67.2	50.6

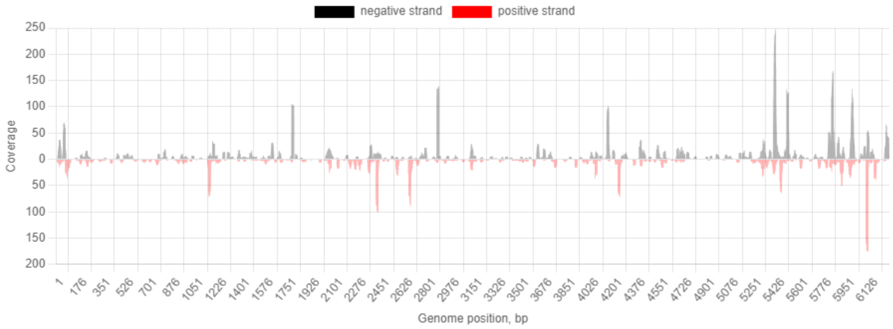

Mapping coverage of all reads matching SMoV_RNA2A (4719 reads)

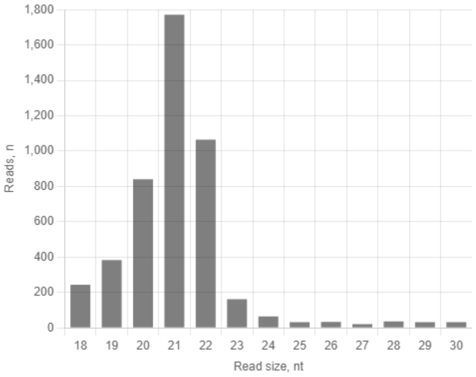

Length distribution of the reads

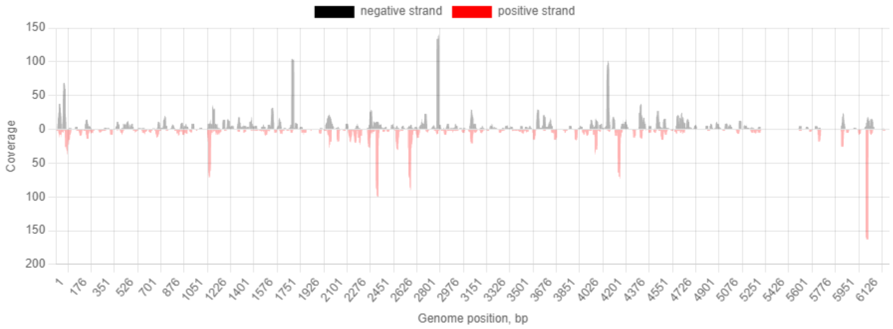

Mapping coverage of SMoV_RNA2A specific reads (3172 reads)

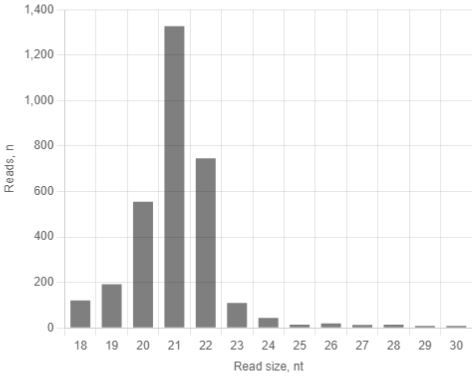

Length distribution of the reads

Dataset	Input reads	Virus	Reference	Mapped reads	Mapped reads, %	RPKM	Forward/Reverse balance	Nonredundant, %	Strain specific	Strain specific, % from total mapped reads	Strain specific, RPKM
2A_2	10000000	SMoV	SMoV_RNA2B	4073	0.041	64.4	1.6	46.4	2198	54.0	34.8

Dataset	Input reads	Virus	Reference	Mapped reads	Mapped reads, %	RPKM	Forward/Reverse balance	Nonredundant, %	Strain specific	Strain specific, % from total mapped reads	Strain specific, RPKM

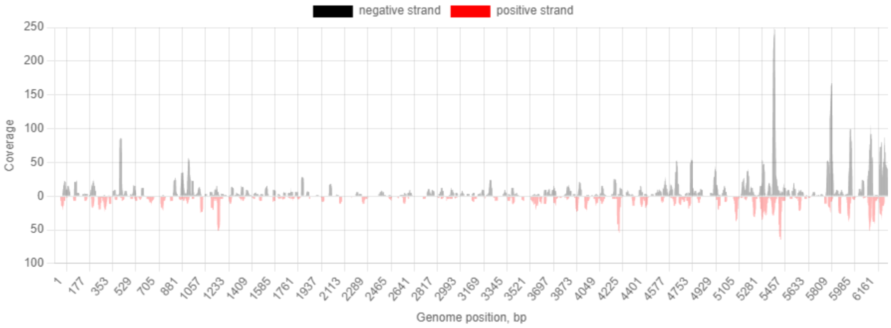

Mapping coverage of all reads matching SMoV_RNA2B (4073 reads)

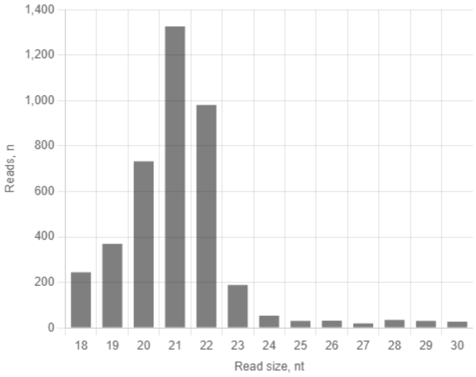

Length distribution of the reads

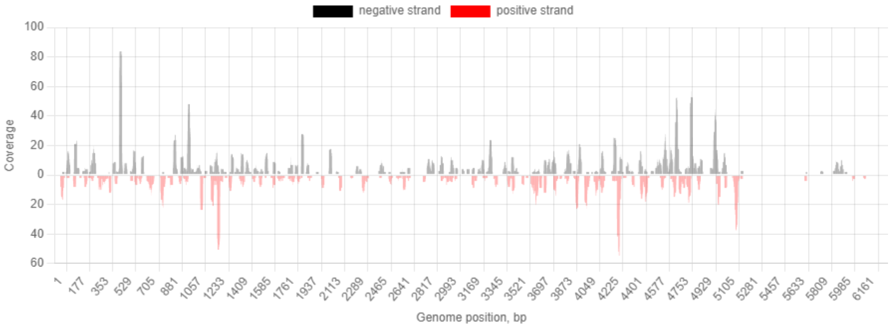

Mapping coverage of SMoV_RNA2B specific reads (2198 reads)

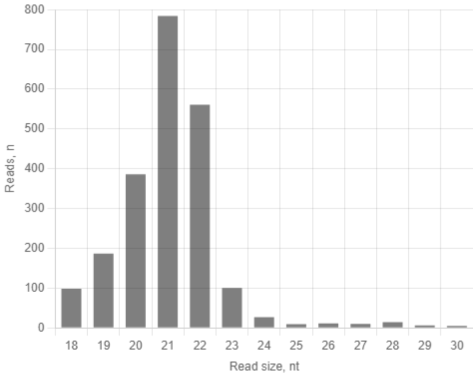

Length distribution of the reads

Dataset	Input reads	Virus	Reference	Mapped reads	Mapped reads, %	RPKM	Forward/Reverse balance	Nonredundant, %	Strain specific	Strain specific, % from total mapped reads	Strain specific, RPKM
2A_2	10000000	SMoV	SMoV_RNA2C	6073	0.061	95.8	1.4	36.7	2588	42.6	40.8

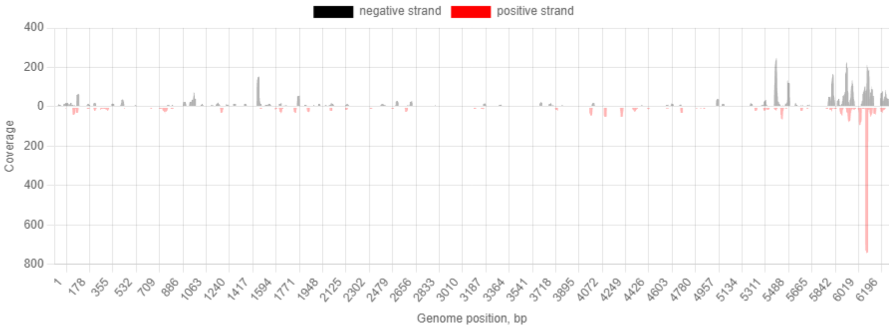

Mapping coverage of all reads matching SMoV_RNA2C (6073 reads)

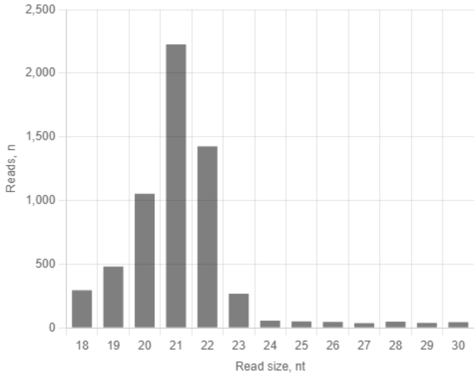

Length distribution of the reads

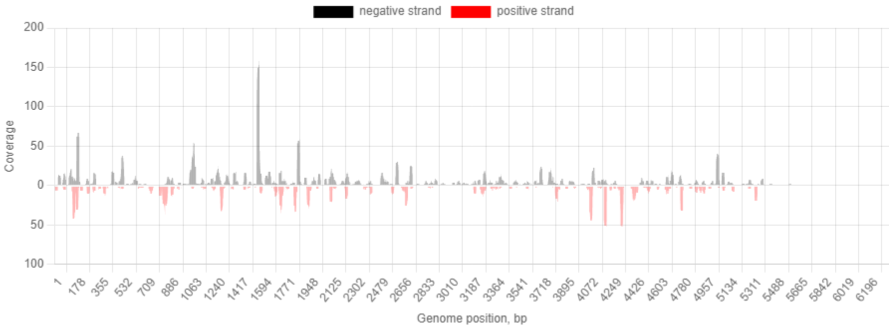

Mapping coverage of SMoV_RNA2C specific reads (2588 reads)

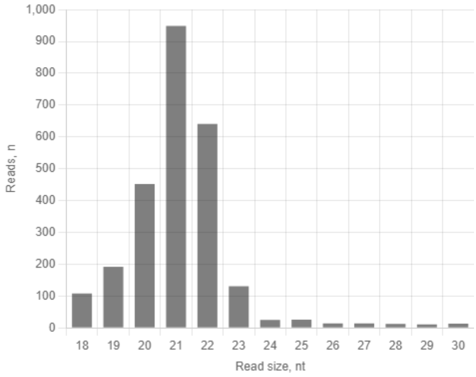

Length distribution of the reads

Dataset	Input reads	Virus	Reference	Mapped reads	Mapped reads, %	RPKM	Forward/Reverse balance	Nonredundant, %	Strain specific	Strain specific, % from total mapped reads	Strain specific, RPKM
2A_2	10000000	StrV1	StrV1_A	7722	0.077	54.5	0.9	43.7	6044	78.3	42.7

Dataset	Input reads	Virus	Reference	Mapped reads	Mapped reads, %	RPKM	Forward/Reverse balance	Nonredundant, %	Strain specific	Strain specific, % from total mapped reads	Strain specific, RPKM

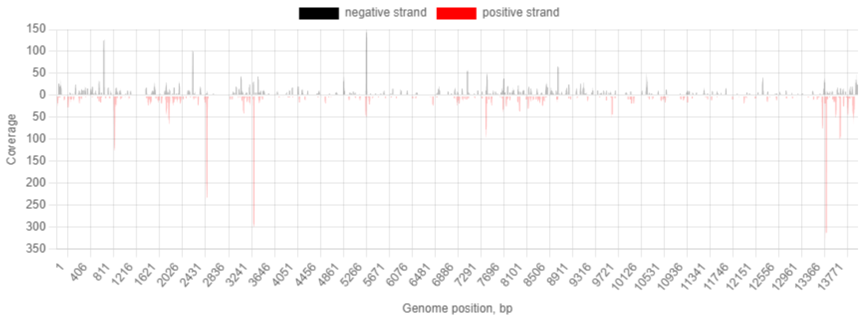

Mapping coverage of all reads matching StrV1_A (7722 reads)

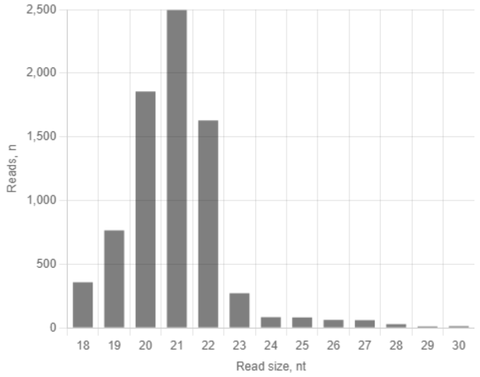

Length distribution of the reads

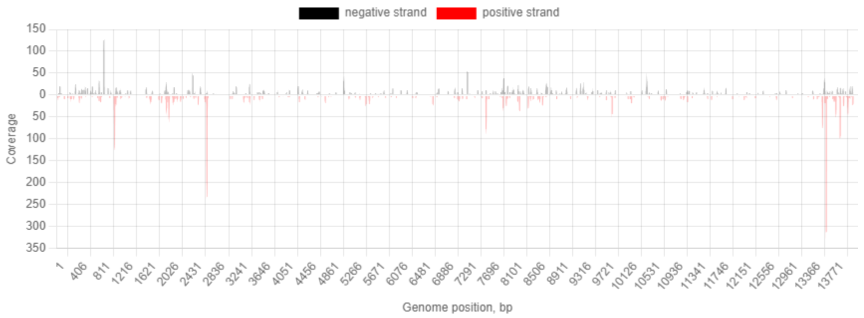

Mapping coverage of StrV1_A specific reads (6044 reads)

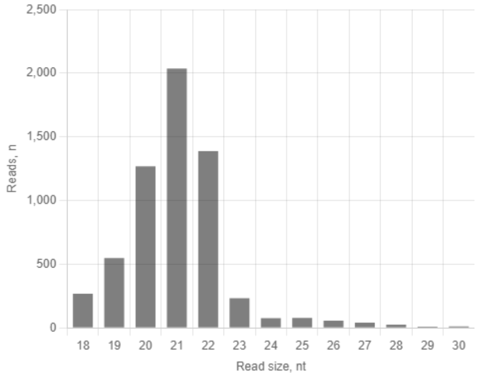

Length distribution of the reads

Dataset	Input reads	Virus	Reference	Mapped reads	Mapped reads, %	RPKM	Forward/Reverse balance	Nonredundant, %	Strain specific	Strain specific, % from total mapped reads	Strain specific, RPKM
2A_2	10000000	StrV1	StrV1_B	12995	0.130	92.6	1.3	34.6	12448	95.8	88.7

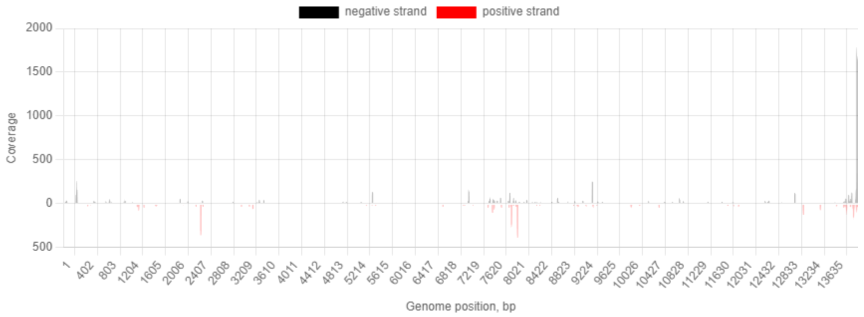

Mapping coverage of all reads matching StrV1_B (12995 reads)

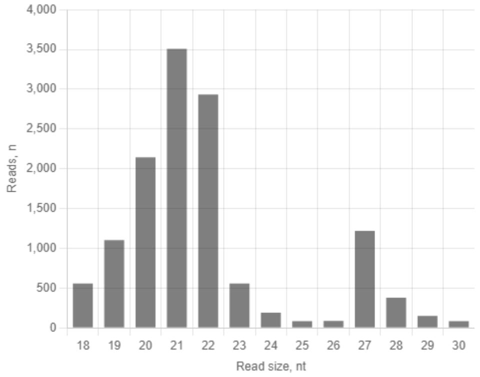

Length distribution of the reads

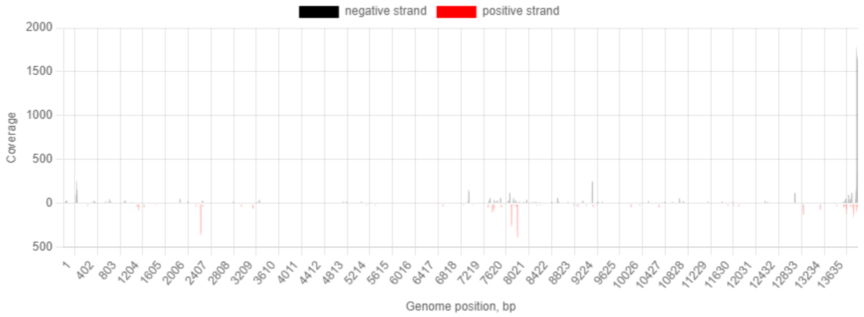

Mapping coverage of StrV1_B specific reads (12448 reads)

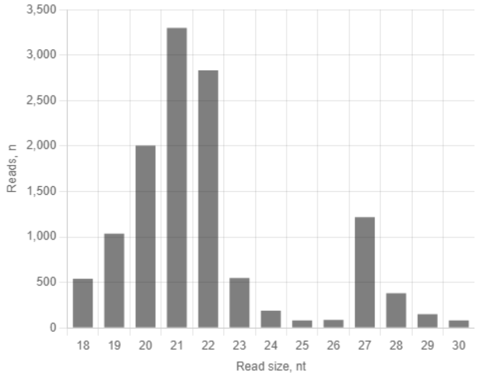

Length distribution of the reads

Dataset	Input reads	Virus	Reference	Mapped reads	Mapped reads, %	RPKM	Forward/Reverse balance	Nonredundant, %	Strain specific	Strain specific, % from total mapped reads	Strain specific, RPKM
2A_2	10000000	StrV1	StrV1_C	5025	0.050	35.3	1.0	47.7	3458	68.8	24.3

Dataset	Input reads	Virus	Reference	Mapped reads	Mapped reads, %	RPKM	Forward/Reverse balance	Nonredundant, %	Strain specific	Strain specific, % from total mapped reads	Strain specific, RPKM

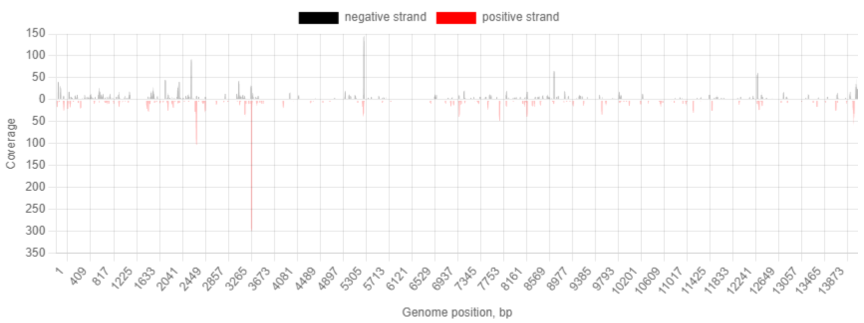

Mapping coverage of all reads matching StrV1_C (5025 reads)

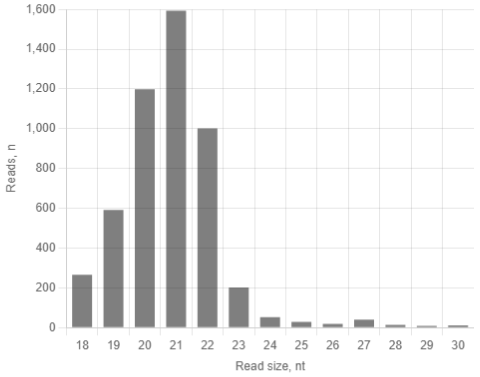

Length distribution of the reads

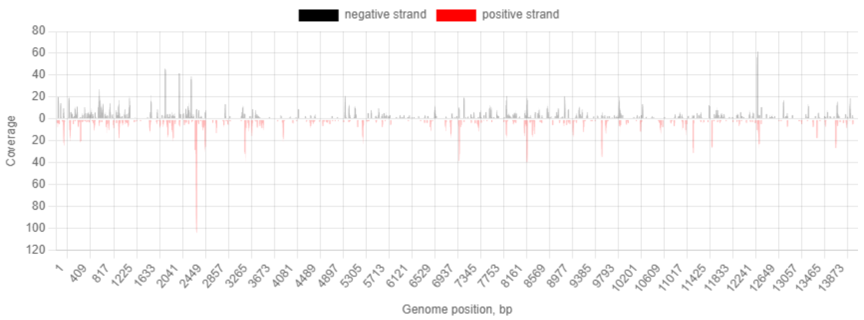

Mapping coverage of StrV1_C specific reads (3458 reads)

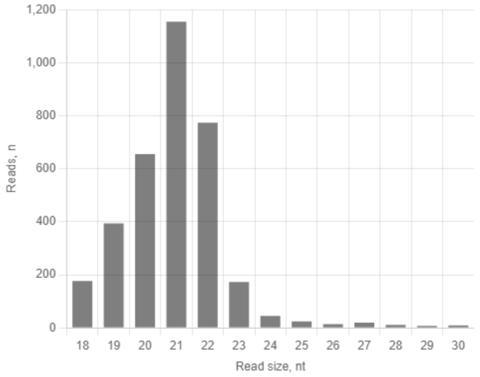

Length distribution of the reads

Mappings of the 2A_3 reads against the viral references.

Dataset	Input reads	Virus	Reference	Mapped reads	Mapped reads, %	RPKM	Forward/Reverse balance	Nonredundant, %	Strain specific	Strain specific, % from total mapped reads	Strain specific, RPKM
2A_3	10000000	SCV	SCV_A	10906	0.109	75.0	0.8	34.3	11245	103.1	62.3

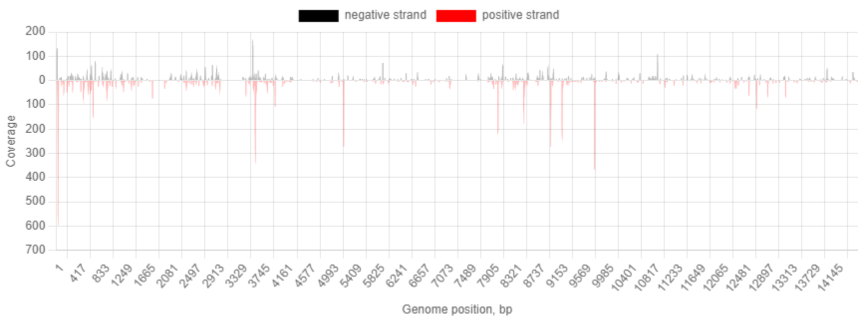

Mapping coverage of all reads matching SCV_A (13189 reads)

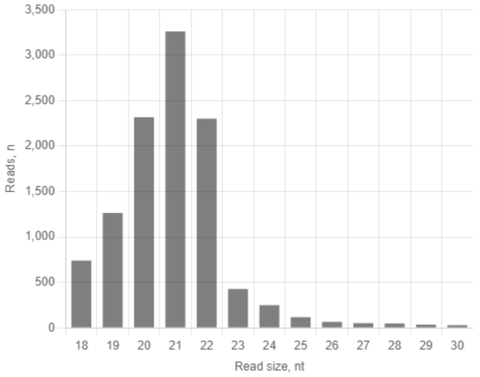

Length distribution of the reads

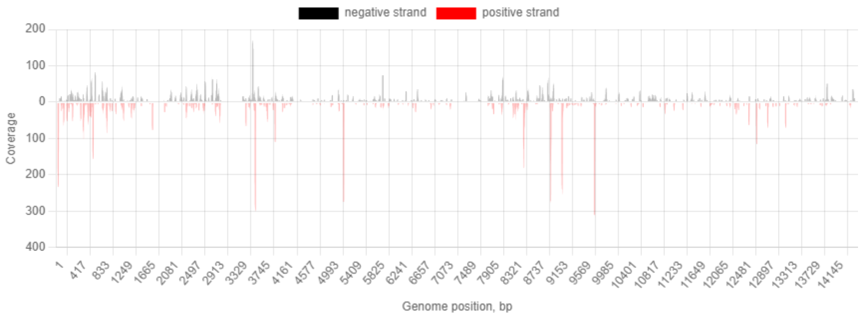

Mapping coverage of SCV_A specific reads (11245 reads)

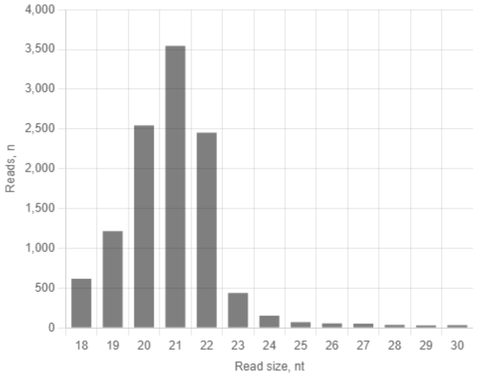

Length distribution of the reads

Dataset	Input reads	Virus	Reference	Mapped reads	Mapped reads, %	RPKM	Forward/Reverse balance	Nonredundant, %	Strain specific	Strain specific, % from total mapped reads	Strain specific, RPKM
2A_3	10000000	SCV	SCV_B	10718	0.107	73.6	0.8	34.9	8870	82.8	61.0

Dataset	Input reads	Virus	Reference	Mapped reads	Mapped reads, %	RPKM	Forward/Reverse balance	Nonredundant, %	Strain specific	Strain specific, % from total mapped reads	Strain specific, RPKM

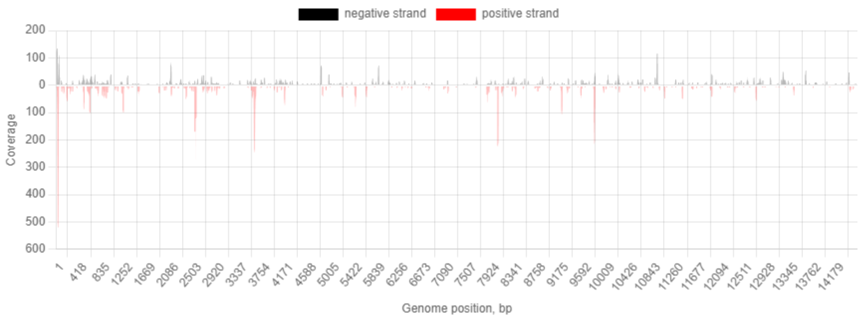

Mapping coverage of all reads matching SCV_B (10814 reads)

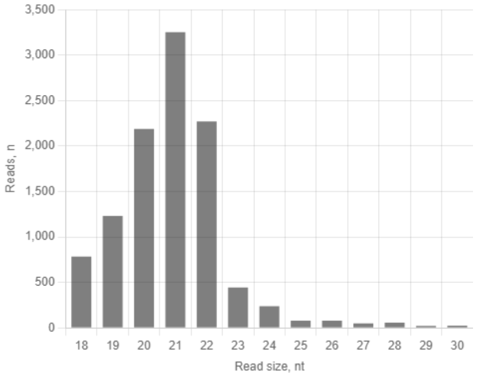

Length distribution of the reads

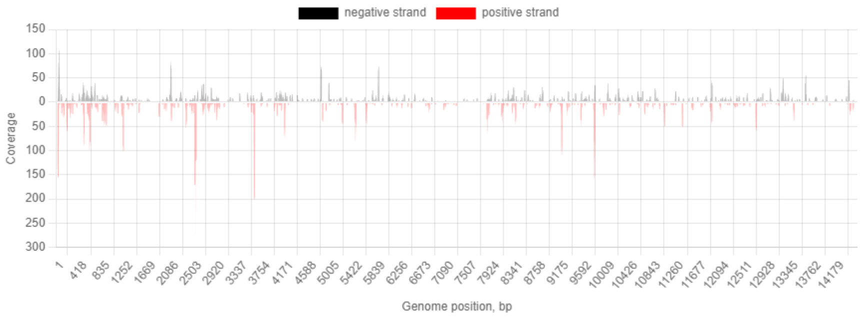

Mapping coverage of SCV_B specific reads (8870 reads)

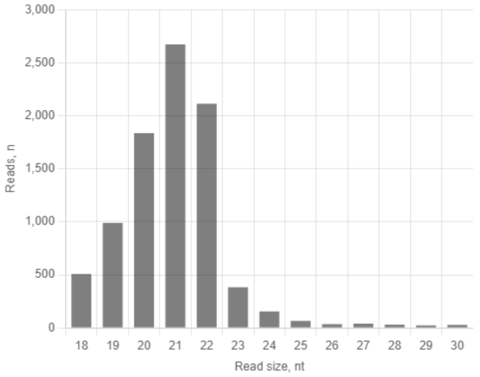

Length distribution of the reads

Dataset	Input reads	Virus	Reference	Mapped reads	Mapped reads, %	RPKM	Forward/Reverse balance	Nonredundant, %	Strain specific	Strain specific, % from total mapped reads	Strain specific, RPKM
2A_3	10000000	SMoV	SMoV_RNA1A	7497	0.075	106.6	1.6	31.9	4257	56.8	57.1

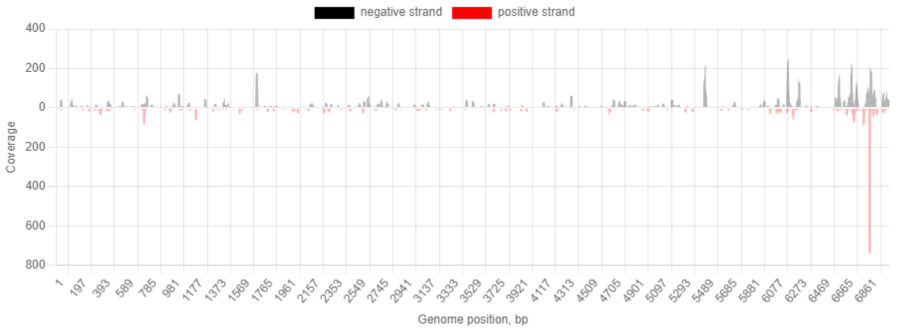

Mapping coverage of all reads matching SMoV_RNA1A (7992 reads)

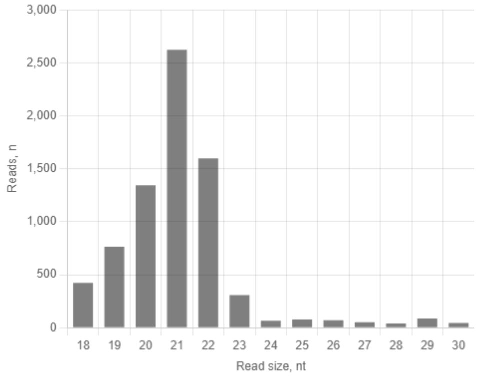

Length distribution of the reads

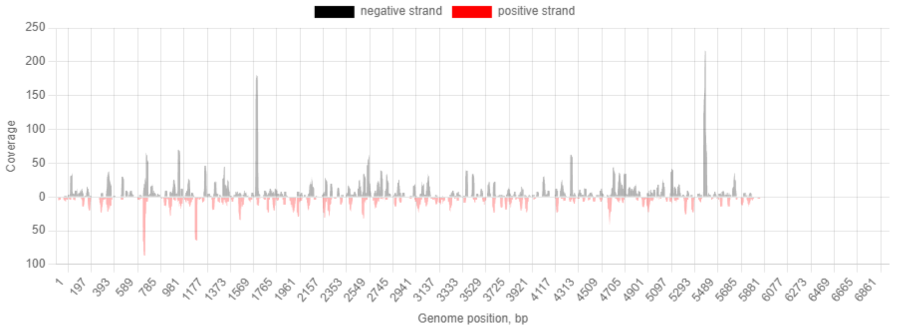

Mapping coverage of SMoV_RNA1A specific reads (4257 reads)

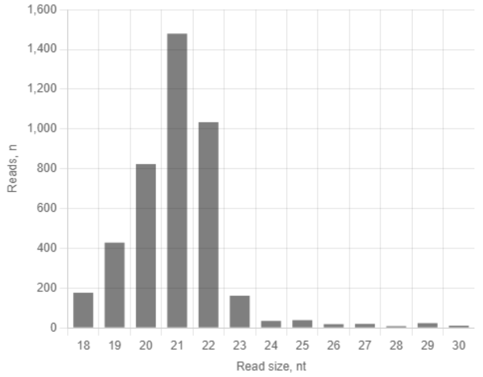

Length distribution of the reads

Dataset	Input reads	Virus	Reference	Mapped reads	Mapped reads, %	RPKM	Forward/Reverse balance	Nonredundant, %	Strain specific	Strain specific, % from total mapped reads	Strain specific, RPKM
2A_3	10000000	SMoV	SMoV_RNA1B	6494	0.065	92.1	1.3	32.6	3443	53.0	44.0

Dataset	Input reads	Virus	Reference	Mapped reads	Mapped reads, %	RPKM	Forward/Reverse balance	Nonredundant, %	Strain specific	Strain specific, % from total mapped reads	Strain specific, RPKM

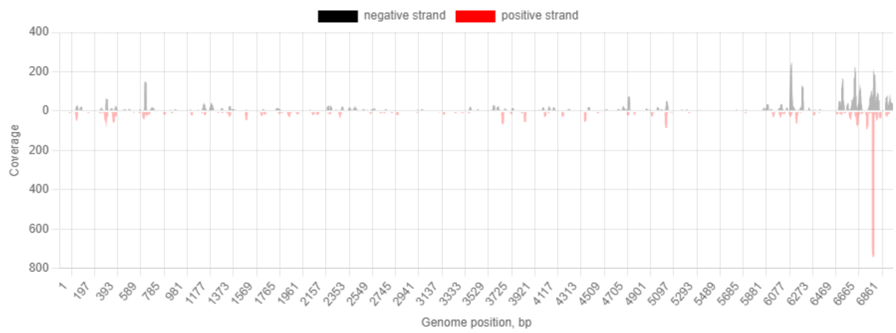

Mapping coverage of **all reads** matching SMoV_RNA1B (7155 reads)

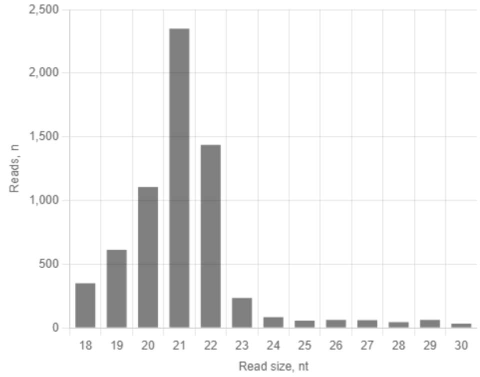

Length distribution of the reads

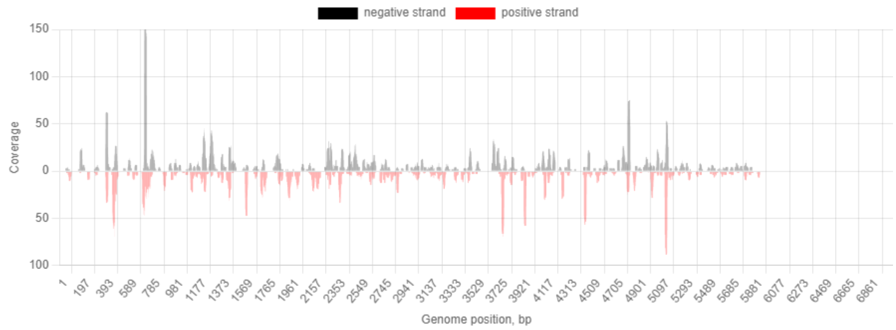

Mapping coverage of SMoV_RNA1B **specific reads** (3443 reads)

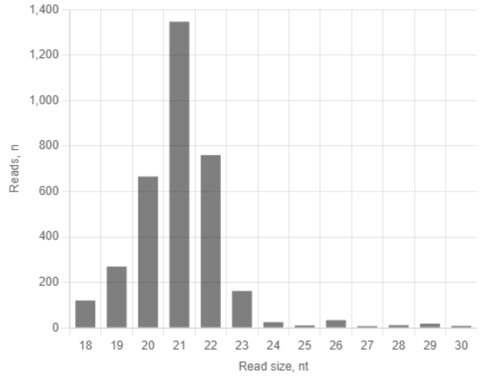

Length distribution of the reads

Dataset	Input reads	Virus	Reference	Mapped reads	Mapped reads, %	RPKM	Forward/Reverse balance	Nonredundant, %	Strain specific	Strain specific, % from total mapped reads	Strain specific, RPKM
2A_3	10000000	SMoV	SMoV_RNA1C	7238	0.072	102.8	1.4	33.0	4015	55.5	53.6

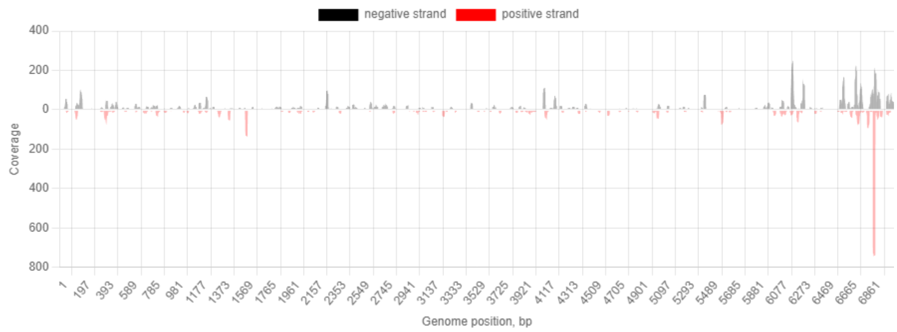

Mapping coverage of **all reads** matching SMoV_RNA1C (7767 reads)

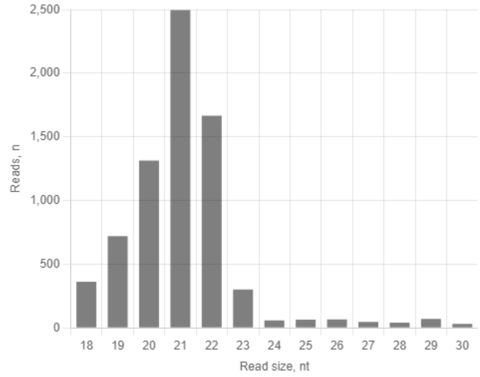

Length distribution of the reads

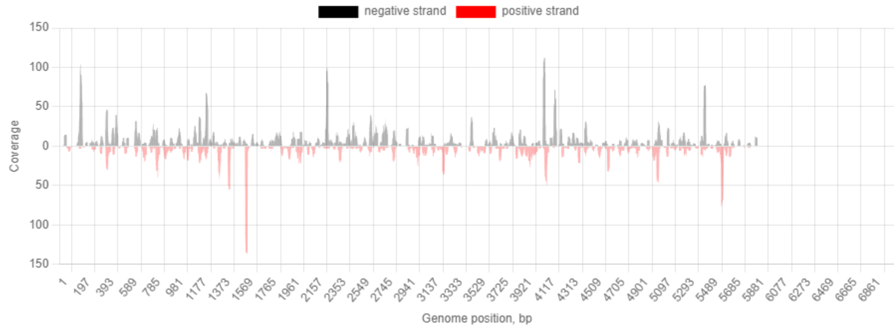

Mapping coverage of SMoV_RNA1C **specific reads** (4015 reads)

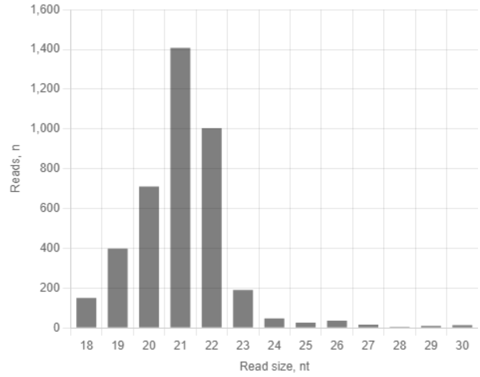

Length distribution of the reads

Dataset	Input reads	Virus	Reference	Mapped reads	Mapped reads, %	RPKM	Forward/Reverse balance	Nonredundant, %	Strain specific	Strain specific, % from total mapped reads	Strain specific, RPKM
2A_3	10000000	SMoV	SMoV_RNA2A	4309	0.043	68.8	1.7	35.0	3172	73.6	42.7

Dataset	Input reads	Virus	Reference	Mapped reads	Mapped reads, %	RPKM	Forward/Reverse balance	Nonredundant, %	Strain specific	Strain specific, % from total mapped reads	Strain specific, RPKM

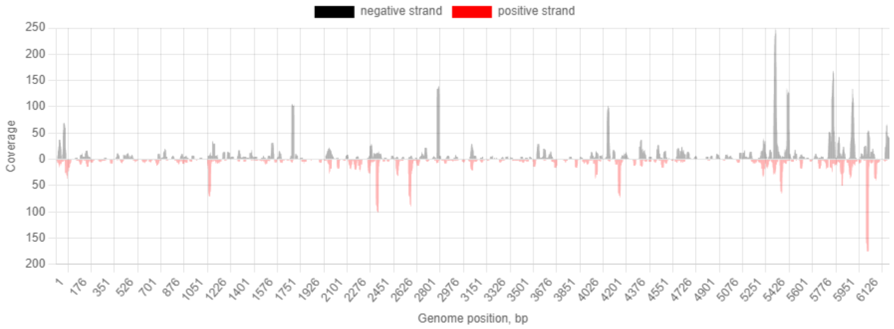

Mapping coverage of **all reads** matching SMoV_RNA2A (4719 reads)

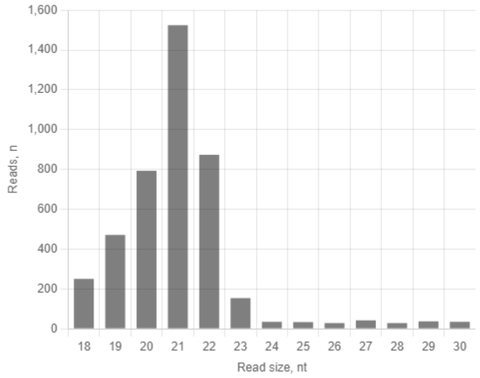

Length distribution of the reads

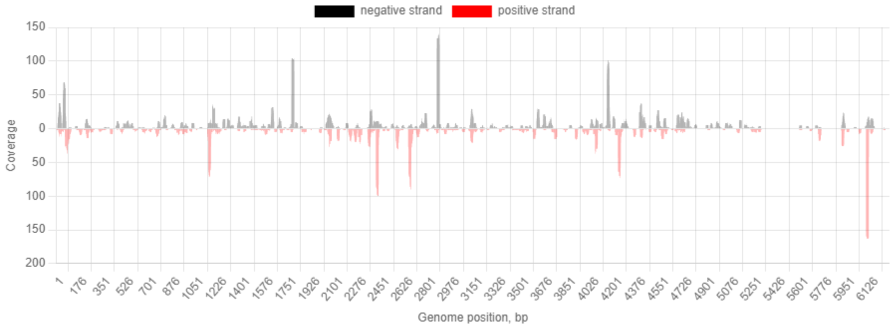

Mapping coverage of SMoV_RNA2A **specific reads** (3172 reads)

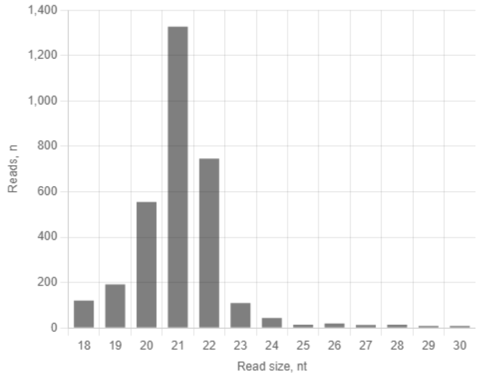

Length distribution of the reads

Dataset	Input reads	Virus	Reference	Mapped reads	Mapped reads, %	RPKM	Forward/Reverse balance	Nonredundant, %	Strain specific	Strain specific, % from total mapped reads	Strain specific, RPKM
2A_3	10000000	SMoV	SMoV_RNA2B	4178	0.042	66.1	1.6	36.2	2198	52.6	35.7

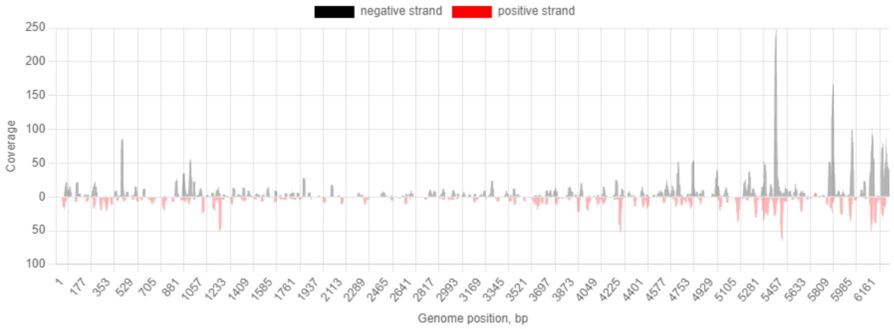

Mapping coverage of **all reads** matching SMoV_RNA2B (4073 reads)

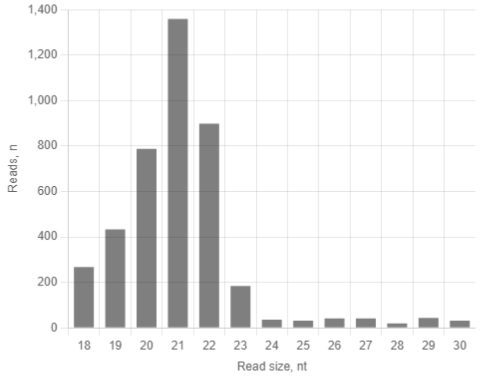

Length distribution of the reads

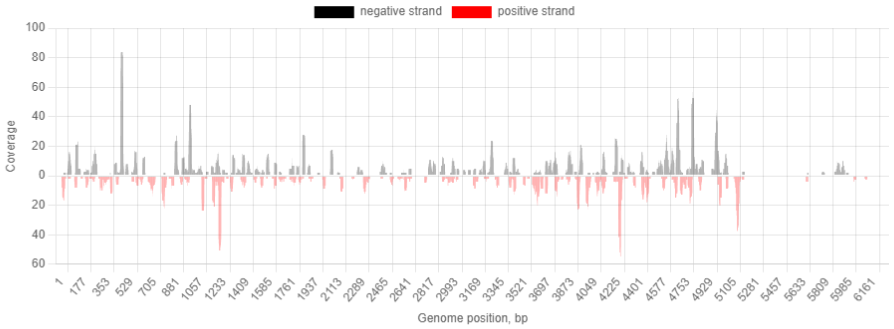

Mapping coverage of SMoV_RNA2B **specific reads** (2198 reads)

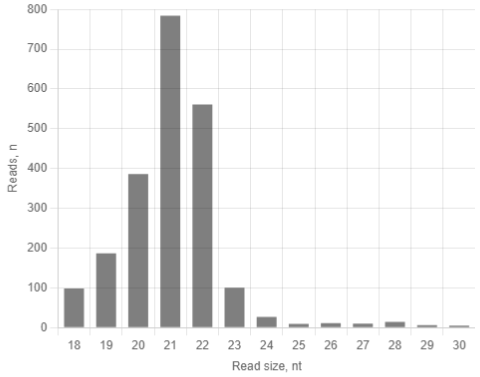

Length distribution of the reads

Dataset	Input reads	Virus	Reference	Mapped reads	Mapped reads, %	RPKM	Forward/Reverse balance	Nonredundant, %	Strain specific	Strain specific, % from total mapped reads	Strain specific, RPKM
2A_3	10000000	SMoV	SMoV_RNA2C	5810	0.058	91.7	1.4	31.3	2588	44.5	41.3

Dataset	Input reads	Virus	Reference	Mapped reads	Mapped reads, %	RPKM	Forward/Reverse balance	Nonredundant, %	Strain specific	Strain specific, % from total mapped reads	Strain specific, RPKM

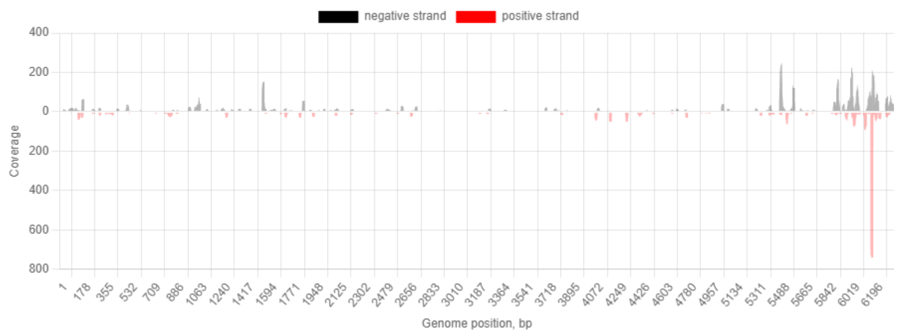

Mapping coverage of all reads matching SMoV_RNA2C (6073 reads)

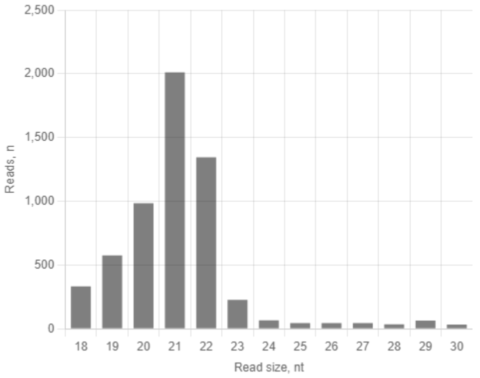

Length distribution of the reads

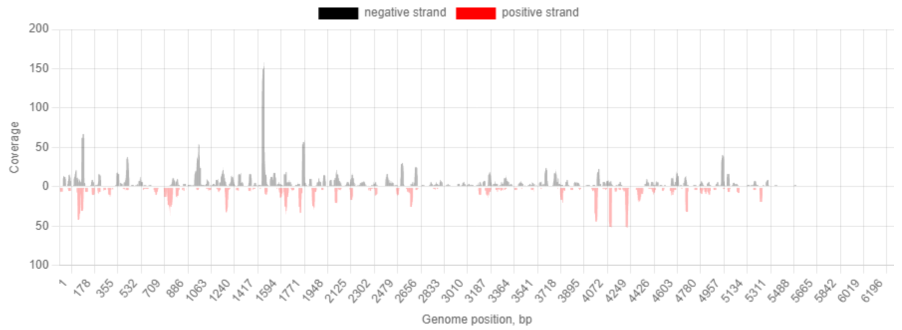

Mapping coverage of SMoV_RNA2C specific reads (2588 reads)

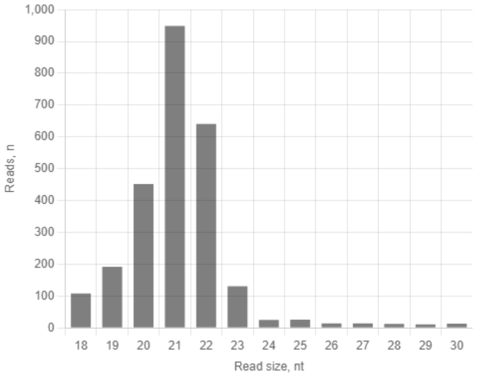

Length distribution of the reads

Dataset	Input reads	Virus	Reference	Mapped reads	Mapped reads, %	RPKM	Forward/Reverse balance	Nonredundant, %	Strain specific	Strain specific, % from total mapped reads	Strain specific, RPKM
2A_3	10000000	StrV1	StrV1_A	7704	0.077	54.4	0.9	35.7	6044	78.5	43.7

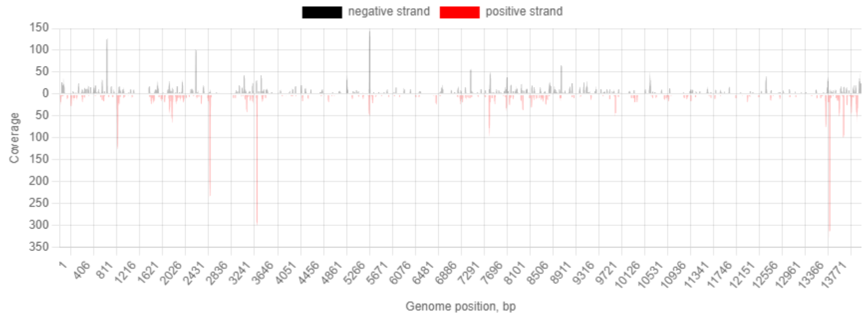

Mapping coverage of all reads matching StrV1_A (7722 reads)

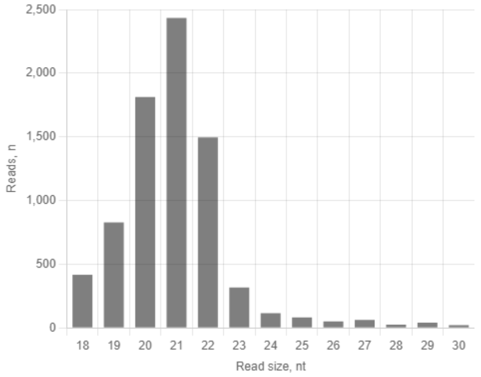

Length distribution of the reads

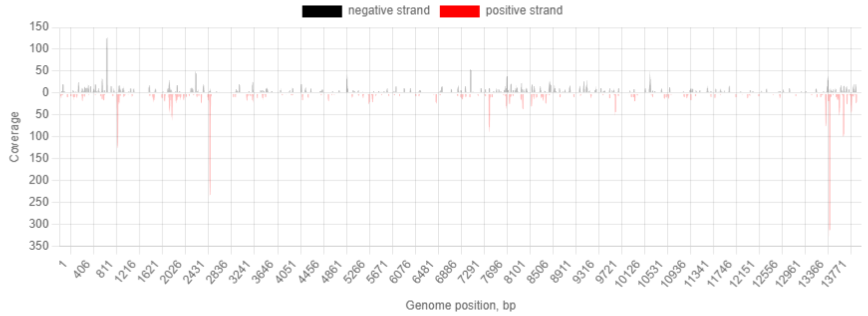

Mapping coverage of StrV1_A specific reads (6044 reads)

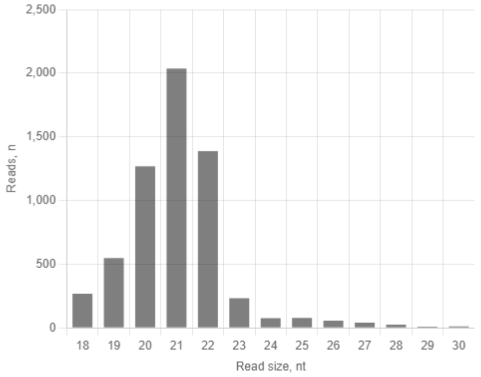

Length distribution of the reads

Dataset	Input reads	Virus	Reference	Mapped reads	Mapped reads, %	RPKM	Forward/Reverse balance	Nonredundant, %	Strain specific	Strain specific, % from total mapped reads	Strain specific, RPKM
2A_3	10000000	StrV1	StrV1_B	16466	0.165	117.4	1.4	26.9	12448	75.6	112.9

Dataset	Input reads	Virus	Reference	Mapped reads	Mapped reads, %	RPKM	Forward/Reverse balance	Nonredundant, %	Strain specific	Strain specific, % from total mapped reads	Strain specific, RPKM

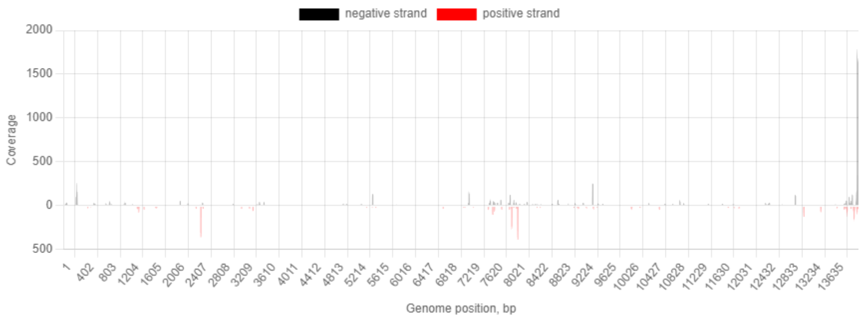

Mapping coverage of all reads matching StrV1_B (12995 reads)

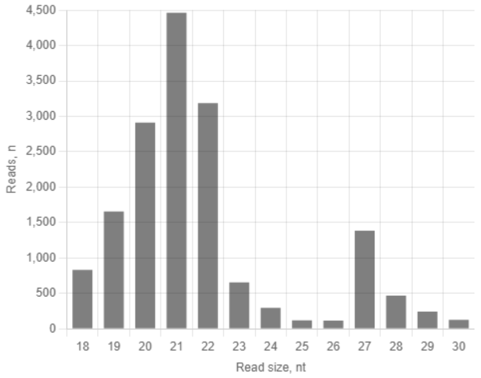

Length distribution of the reads

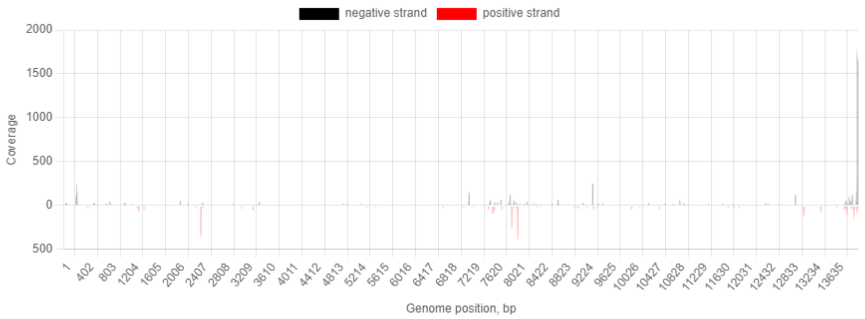

Mapping coverage of StrV1_B specific reads (12448 reads)

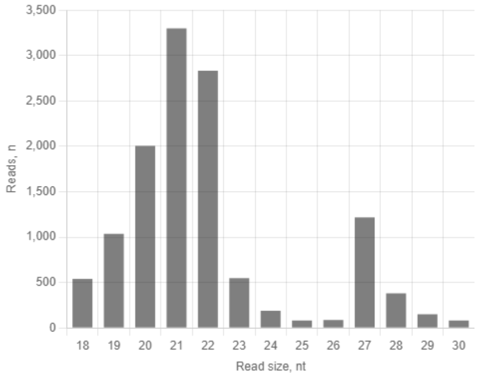

Length distribution of the reads

Dataset	Input reads	Virus	Reference	Mapped reads	Mapped reads, %	RPKM	Forward/Reverse balance	Nonredundant, %	Strain specific	Strain specific, % from total mapped reads	Strain specific, RPKM
2A_3	10000000	StrV1	StrV1_C	4232	0.042	29.7	1.1	38.4	3458	81.7	20.1

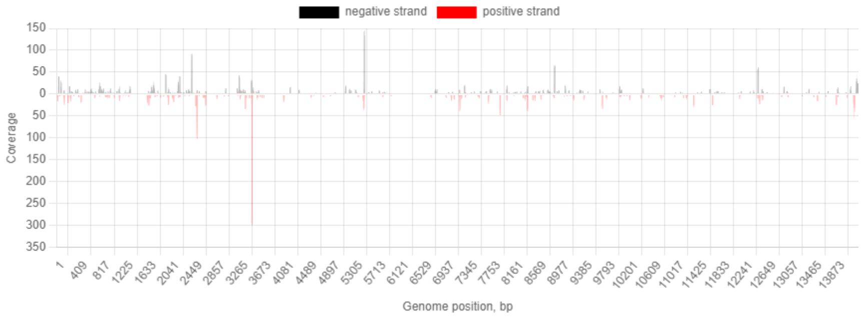

Mapping coverage of all reads matching StrV1_C (5025 reads)

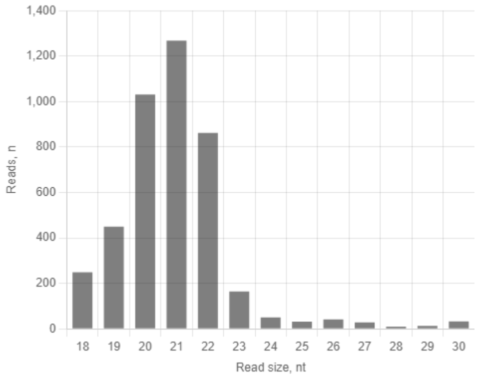

Length distribution of the reads

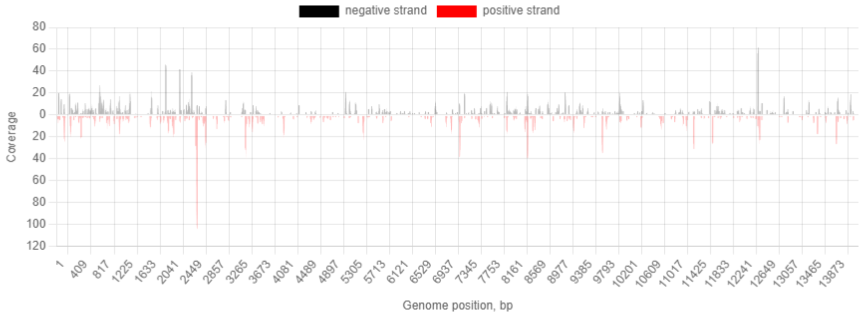

Mapping coverage of StrV1_C specific reads (3458 reads)

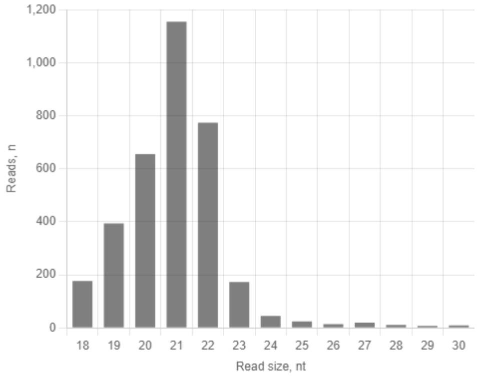

Length distribution of the reads

Mappings of the 2A_4 reads against the viral references.

Dataset	Input reads	Virus	Reference	Mapped reads	Mapped reads, %	RPKM	Forward/Reverse balance	Nonredundant, %	Strain specific	Strain specific, % from total mapped reads	Strain specific, RPKM
2A_4	10000000	SCV	SCV_A	5649	0.056	38.8	0.8	32.4	11245	199.1	33.1

Dataset	Input reads	Virus	Reference	Mapped reads	Mapped reads, %	RPKM	Forward/Reverse balance	Nonredundant, %	Strain specific	Strain specific, % from total mapped reads	Strain specific, RPKM

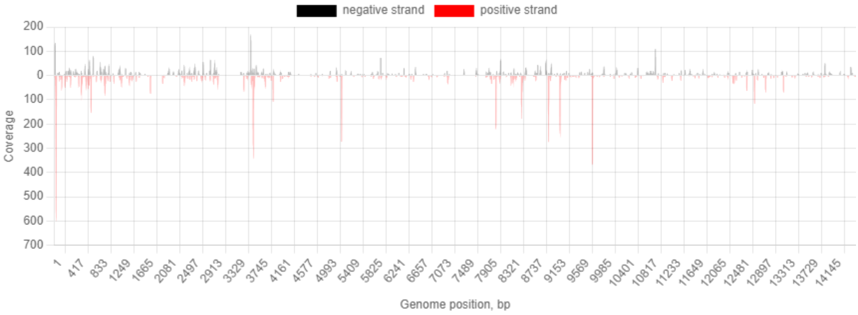

Mapping coverage of all reads matching SCV_A (13189 reads)

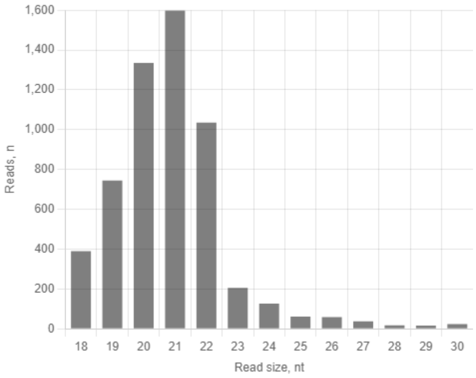

Length distribution of the reads

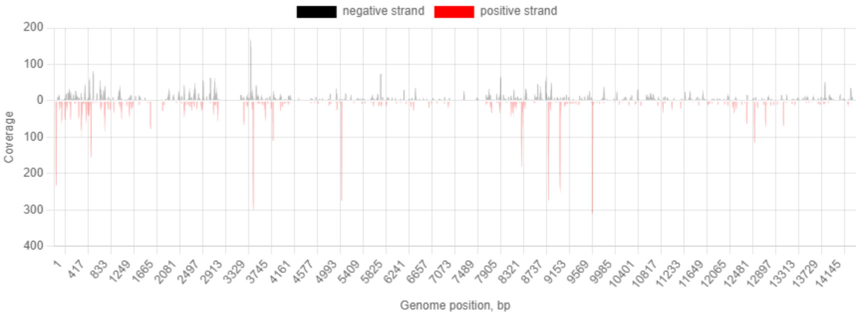

Mapping coverage of SCV_A specific reads (11245 reads)

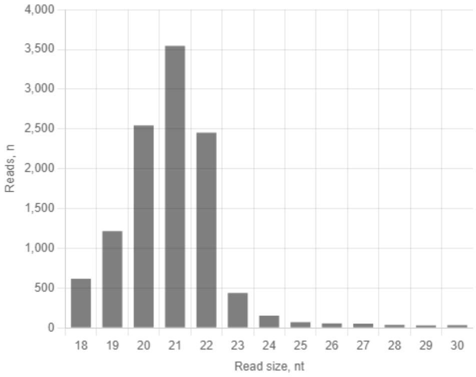

Length distribution of the reads

Dataset	Input reads	Virus	Reference	Mapped reads	Mapped reads, %	RPKM	Forward/Reverse balance	Nonredundant, %	Strain specific	Strain specific, % from total mapped reads	Strain specific, RPKM
2A_4	10000000	SCV	SCV_B	4337	0.043	29.8	0.8	35.0	8870	204.5	24.1

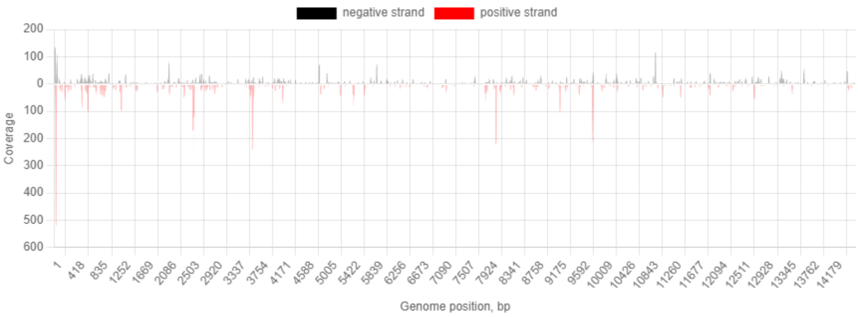

Mapping coverage of all reads matching SCV_B (10814 reads)

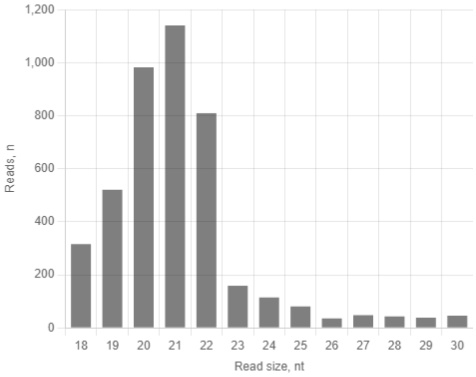

Length distribution of the reads

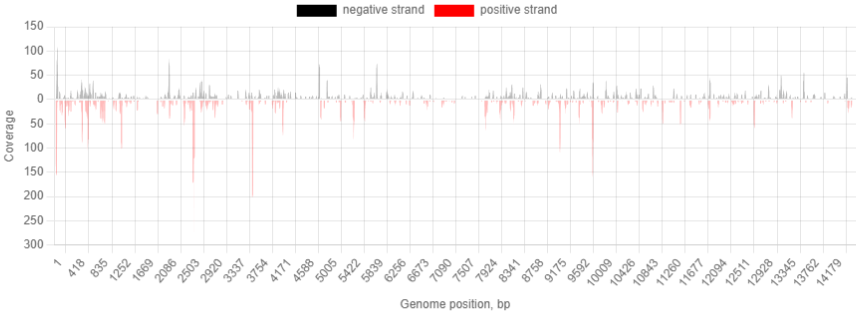

Mapping coverage of SCV_B specific reads (8870 reads)

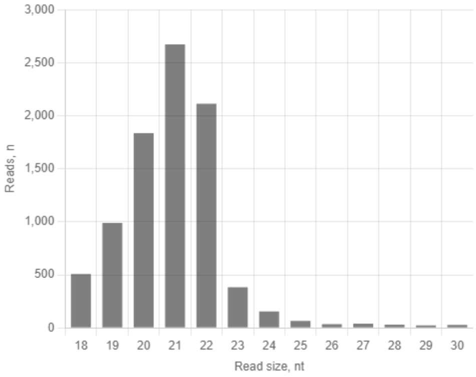

Length distribution of the reads

Dataset	Input reads	Virus	Reference	Mapped reads	Mapped reads, %	RPKM	Forward/Reverse balance	Nonredundant, %	Strain specific	Strain specific, % from total mapped reads	Strain specific, RPKM
2A_4	10000000	SMoV	SMoV_RNA1A	6062	0.061	86.2	1.5	28.3	4257	70.2	47.5

Dataset	Input reads	Virus	Reference	Mapped reads	Mapped reads, %	RPKM	Forward/Reverse balance	Nonredundant, %	Strain specific	Strain specific, % from total mapped reads	Strain specific, RPKM

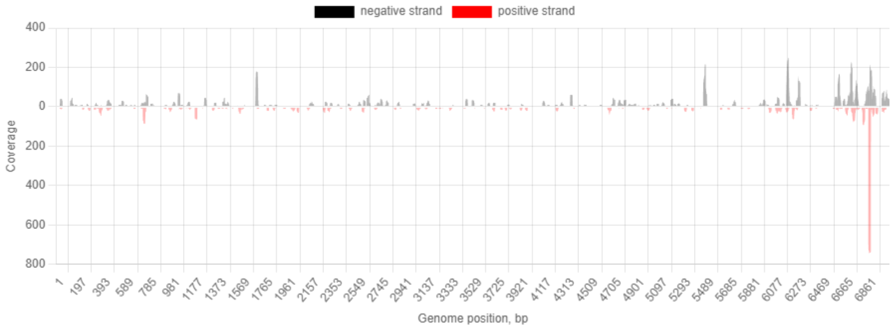

Mapping coverage of all reads matching SMoV_RNA1A (7992 reads)

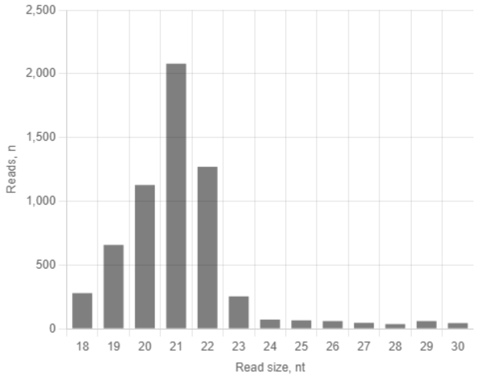

Length distribution of the reads

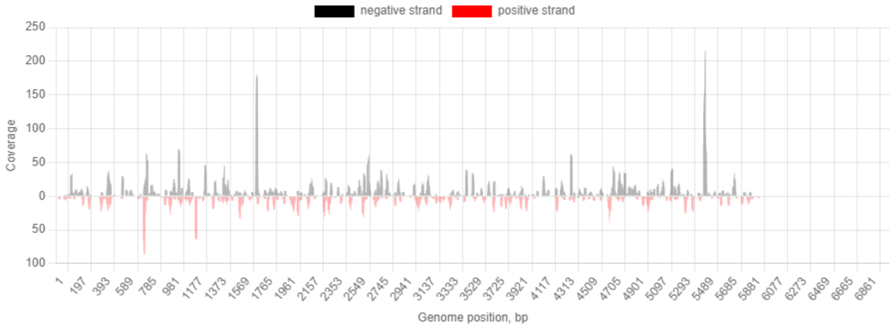

Mapping coverage of SMoV_RNA1A specific reads (4257 reads)

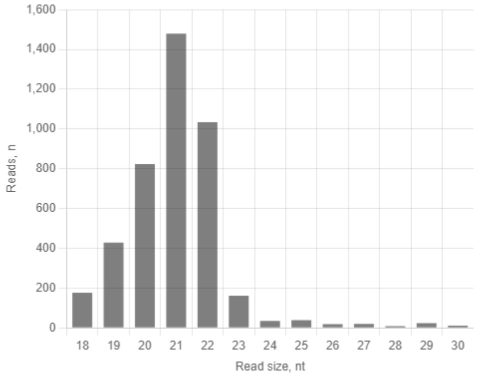

Length distribution of the reads

Dataset	Input reads	Virus	Reference	Mapped reads	Mapped reads, %	RPKM	Forward/Reverse balance	Nonredundant, %	Strain specific	Strain specific, % from total mapped reads	Strain specific, RPKM
2A_4	10000000	SMoV	SMoV_RNA1B	5372	0.054	76.2	1.2	29.5	3443	64.1	38.0

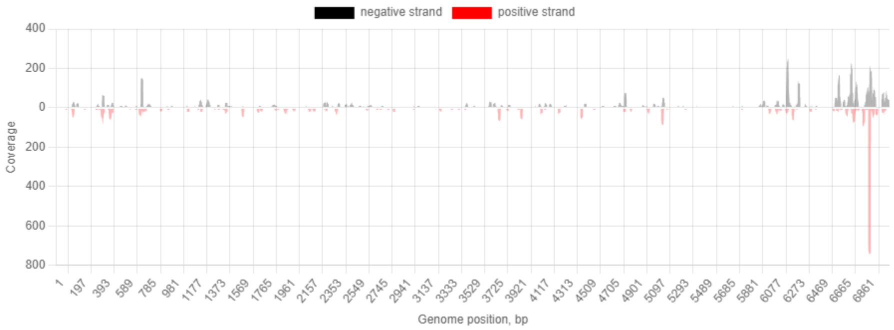

Mapping coverage of all reads matching SMoV_RNA1B (7155 reads)

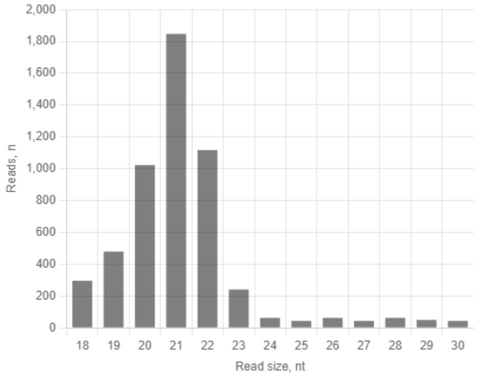

Length distribution of the reads

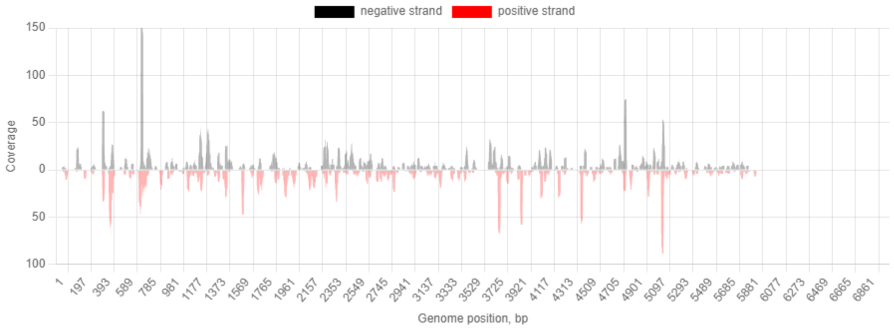

Mapping coverage of SMoV_RNA1B specific reads (3443 reads)

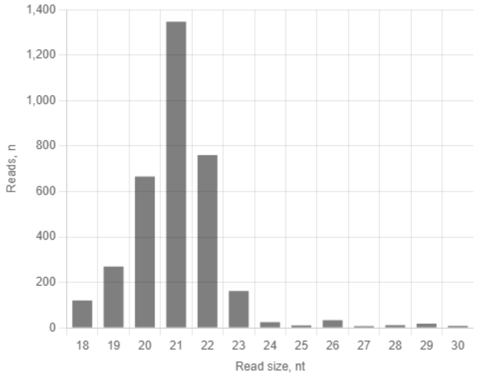

Length distribution of the reads

Dataset	Input reads	Virus	Reference	Mapped reads	Mapped reads, %	RPKM	Forward/Reverse balance	Nonredundant, %	Strain specific	Strain specific, % from total mapped reads	Strain specific, RPKM
2A_4	10000000	SMoV	SMoV_RNA1C	5813	0.058	82.6	1.3	29.1	4015	69.1	43.7

Dataset	Input reads	Virus	Reference	Mapped reads	Mapped reads, %	RPKM	Forward/Reverse balance	Nonredundant, %	Strain specific	Strain specific, % from total mapped reads	Strain specific, RPKM

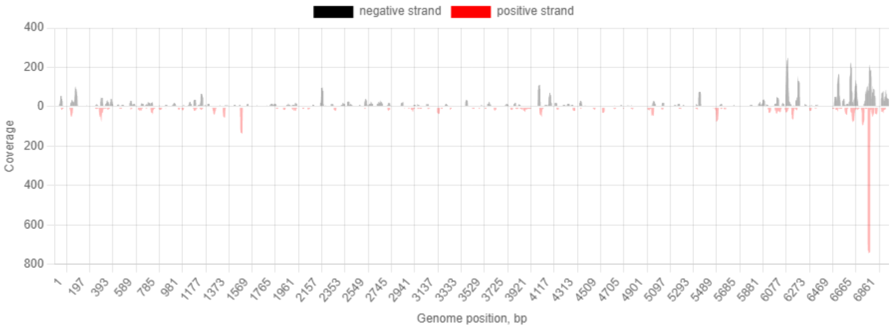

Mapping coverage of **all reads** matching SMoV_RNA1C (7767 reads)

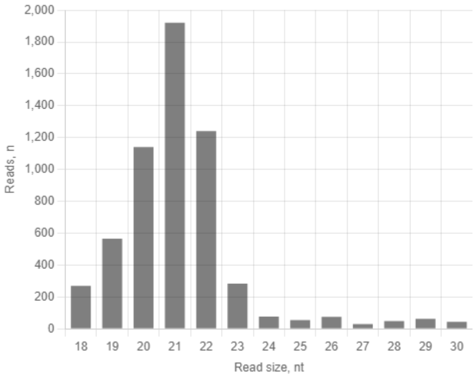

Length distribution of the reads

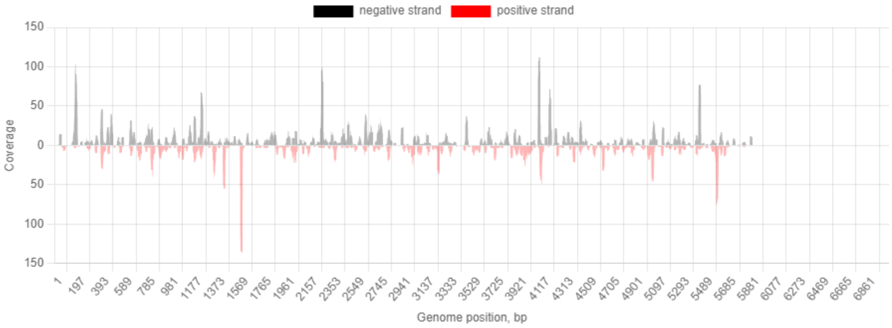

Mapping coverage of SMoV_RNA1C **specific reads** (4015 reads)

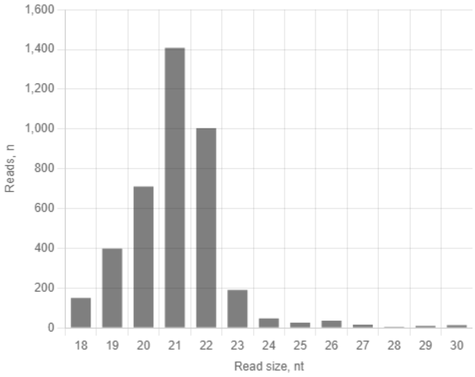

Length distribution of the reads

Dataset	Input reads	Virus	Reference	Mapped reads	Mapped reads, %	RPKM	Forward/Reverse balance	Nonredundant, %	Strain specific	Strain specific, % from total mapped reads	Strain specific, RPKM
2A_4	10000000	SMoV	SMoV_RNA2A	3808	0.038	60.7	1.7	30.6	3172	83.3	42.2

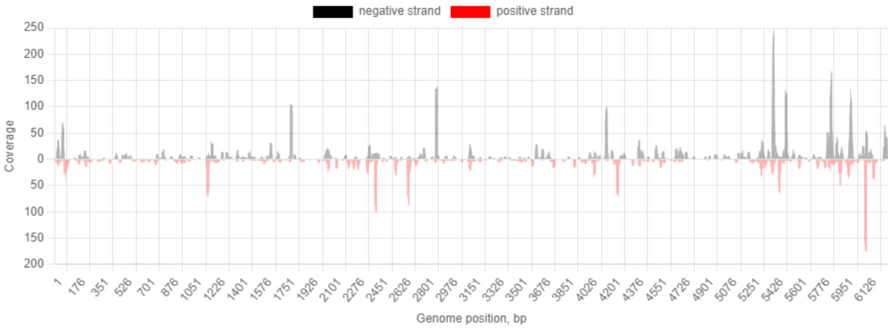

Mapping coverage of **all reads** matching SMoV_RNA2A (4719 reads)

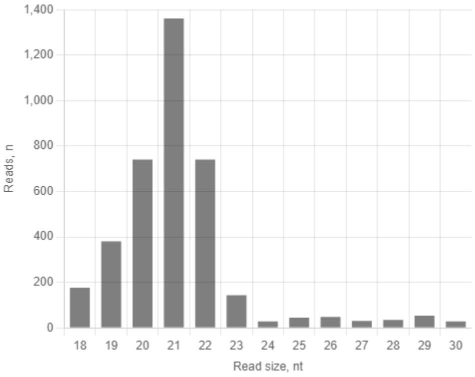

Length distribution of the reads

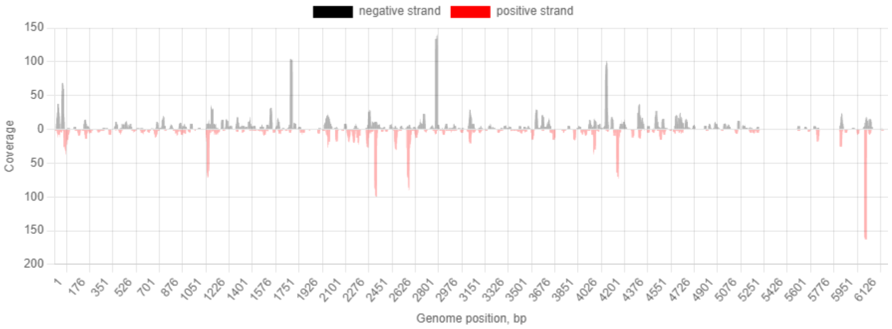

Mapping coverage of SMoV_RNA2A **specific reads** (3172 reads)

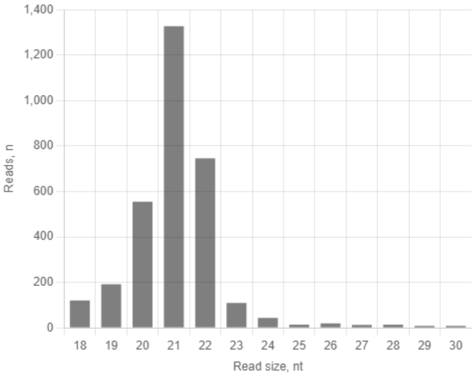

Length distribution of the reads

Dataset	Input reads	Virus	Reference	Mapped reads	Mapped reads, %	RPKM	Forward/Reverse balance	Nonredundant, %	Strain specific	Strain specific, % from total mapped reads	Strain specific, RPKM
2A_4	10000000	SMoV	SMoV_RNA2B	3090	0.031	48.9	1.7	33.5	2198	71.1	26.2

Dataset	Input reads	Virus	Reference	Mapped reads	Mapped reads, %	RPKM	Forward/Reverse balance	Nonredundant, %	Strain specific	Strain specific, % from total mapped reads	Strain specific, RPKM

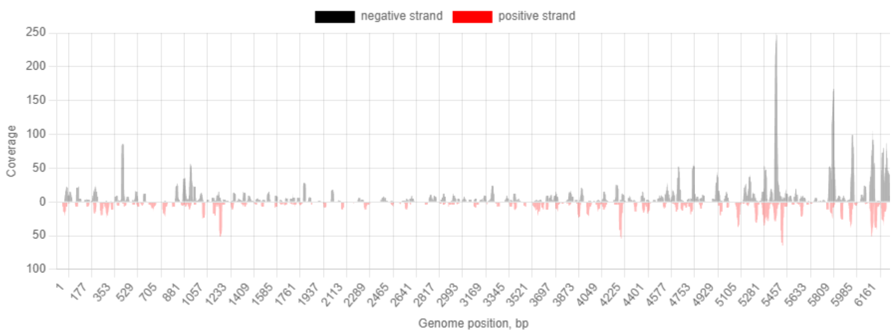

Mapping coverage of **all reads** matching SMoV_RNA2B (4073 reads)

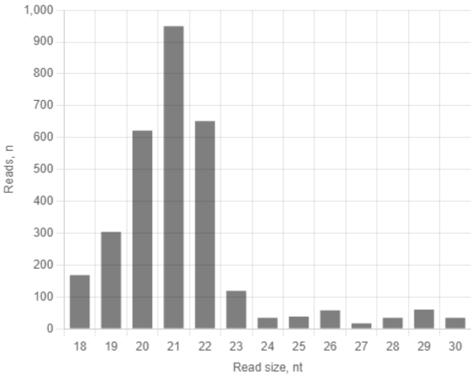

Length distribution of the reads

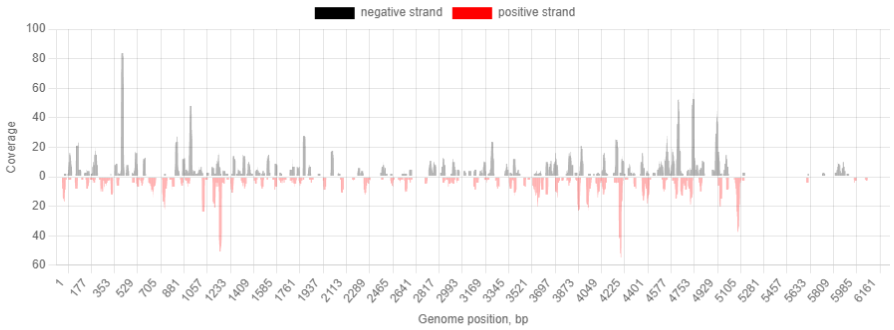

Mapping coverage of SMoV_RNA2B **specific reads** (2198 reads)

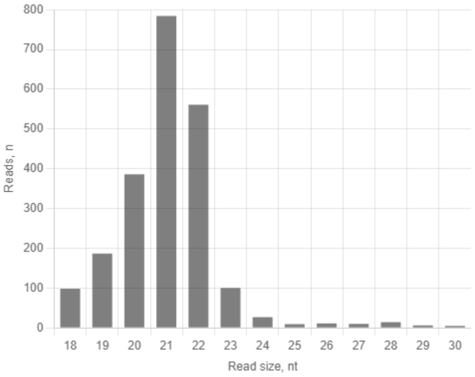

Length distribution of the reads

Dataset	Input reads	Virus	Reference	Mapped reads	Mapped reads, %	RPKM	Forward/Reverse balance	Nonredundant, %	Strain specific	Strain specific, % from total mapped reads	Strain specific, RPKM
2A_4	10000000	SMoV	SMoV_RNA2C	4626	0.046	73.0	1.4	28.0	2588	55.9	33.7

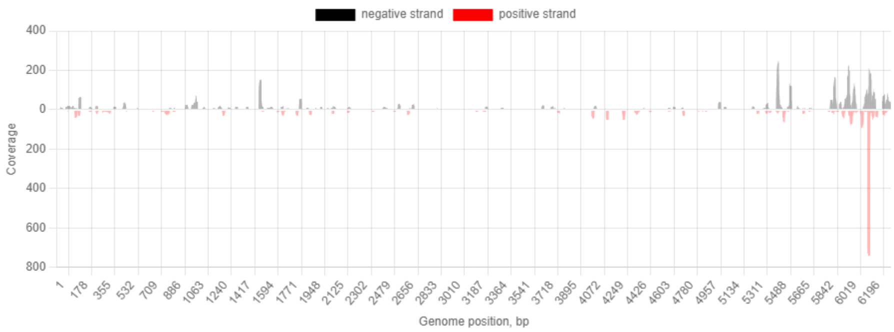

Mapping coverage of **all reads** matching SMoV_RNA2C (6073 reads)

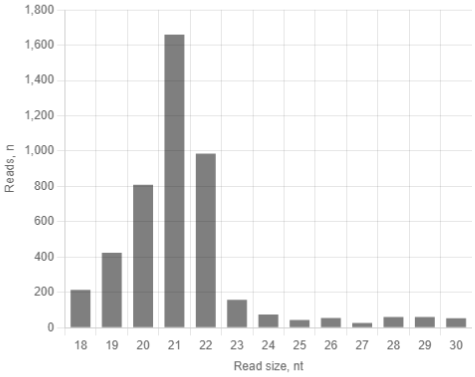

Length distribution of the reads

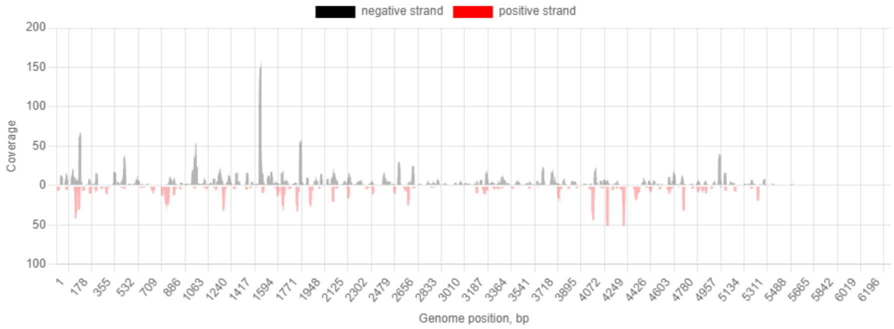

Mapping coverage of SMoV_RNA2C **specific reads** (2588 reads)

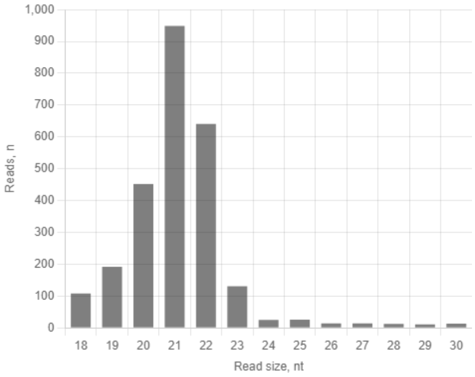

Length distribution of the reads

Dataset	Input reads	Virus	Reference	Mapped reads	Mapped reads, %	RPKM	Forward/Reverse balance	Nonredundant, %	Strain specific	Strain specific, % from total mapped reads	Strain specific, RPKM
2A_4	10000000	StrV1	StrV1_A	3009	0.030	21.2	0.8	35.1	6044	200.9	17.4

Dataset	Input reads	Virus	Reference	Mapped reads	Mapped reads, %	RPKM	Forward/Reverse balance	Nonredundant, %	Strain specific	Strain specific, % from total mapped reads	Strain specific, RPKM

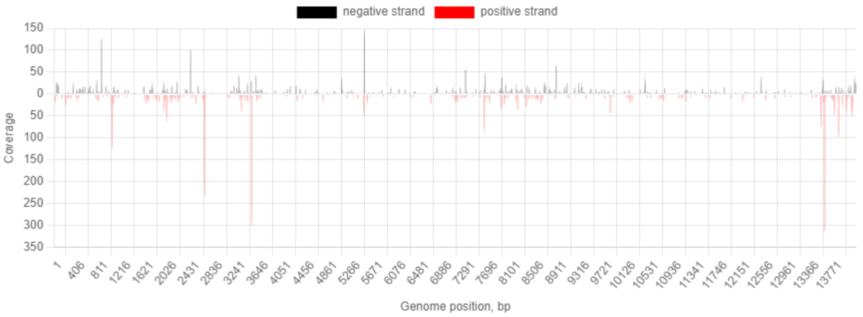

Mapping coverage of all reads matching StrV1_A (7722 reads)

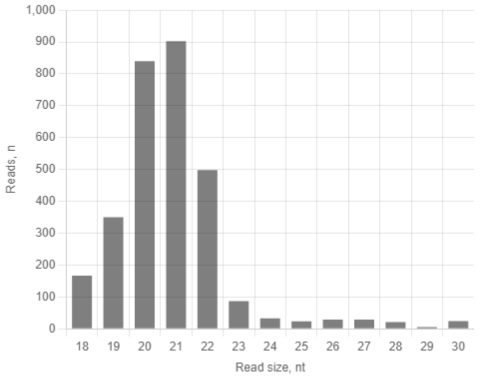

Length distribution of the reads

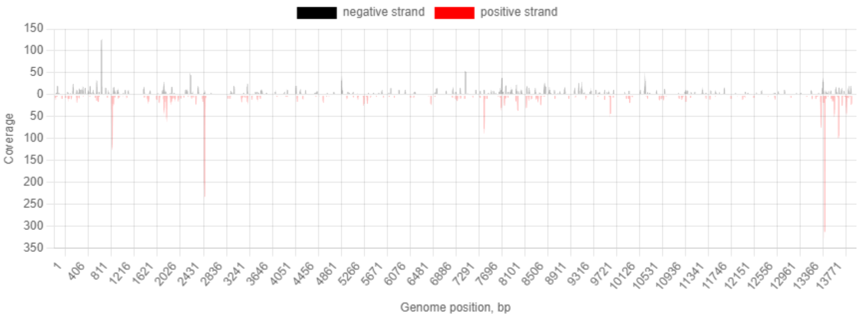

Mapping coverage of StrV1_A specific reads (6044 reads)

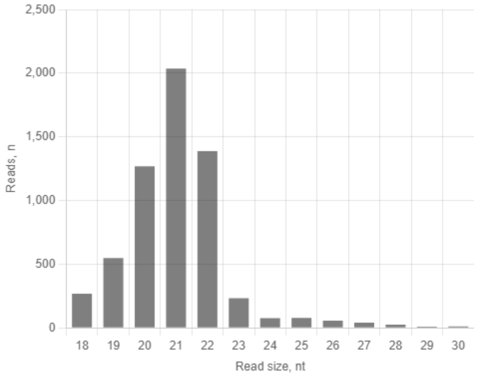

Length distribution of the reads

Dataset	Input reads	Virus	Reference	Mapped reads	Mapped reads, %	RPKM	Forward/Reverse balance	Nonredundant, %	Strain specific	Strain specific, % from total mapped reads	Strain specific, RPKM
2A_4	10000000	StrV1	StrV1_B	8341	0.083	59.5	1.6	25.7	12448	149.2	58.2

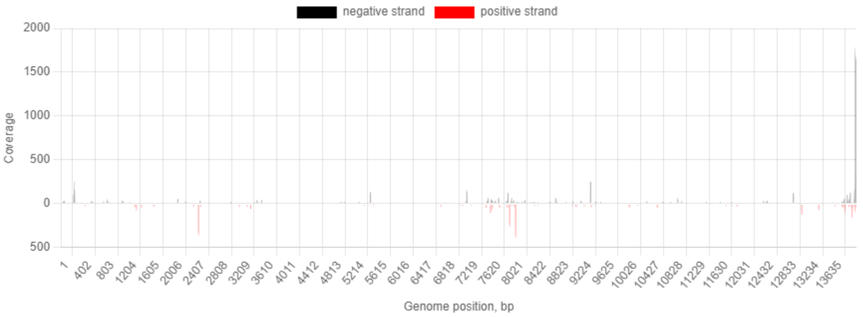

Mapping coverage of all reads matching StrV1_B (12995 reads)

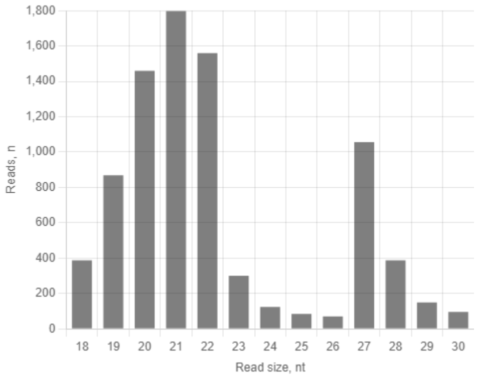

Length distribution of the reads

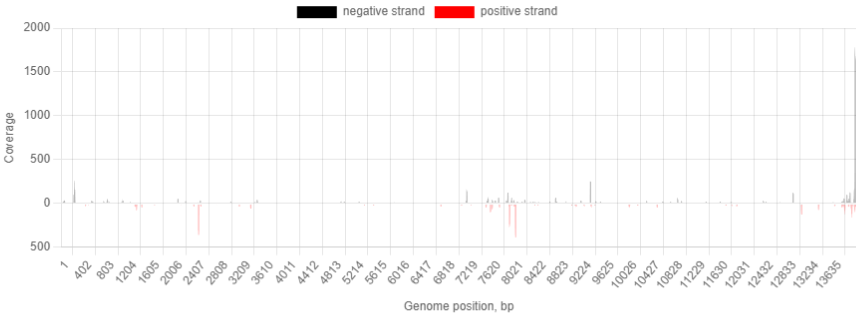

Mapping coverage of StrV1_B specific reads (12448 reads)

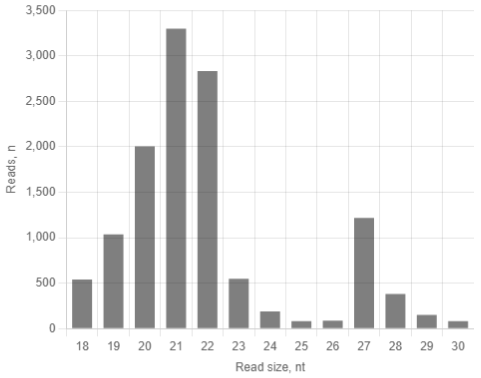

Length distribution of the reads

Dataset	Input reads	Virus	Reference	Mapped reads	Mapped reads, %	RPKM	Forward/Reverse balance	Nonredundant, %	Strain specific	Strain specific, % from total mapped reads	Strain specific, RPKM
2A_4	10000000	StrV1	StrV1_C	1428	0.014	10.0	1.0	36.4	3458	242.2	6.4

Dataset	Input reads	Virus	Reference	Mapped reads	Mapped reads, %	RPKM	Forward/Reverse balance	Nonredundant, %	Strain specific	Strain specific, % from total mapped reads	Strain specific, RPKM

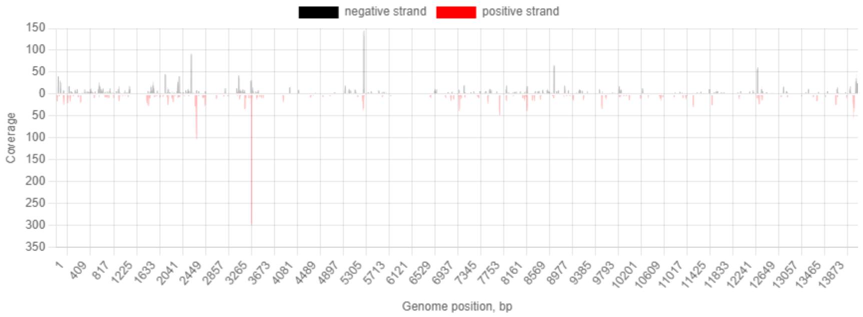

Mapping coverage of all reads matching StrV1_C (5025 reads)

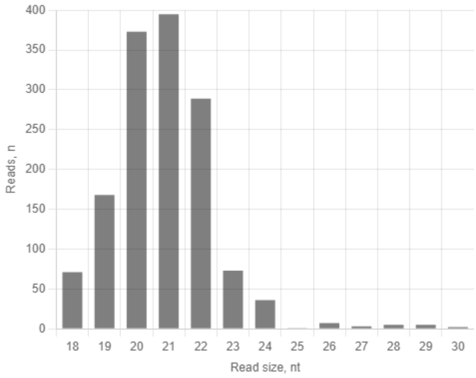

Length distribution of the reads

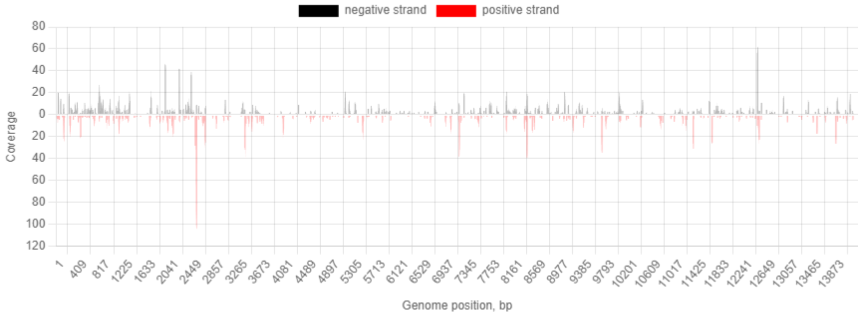

Mapping coverage of StrV1_C specific reads (3458 reads)

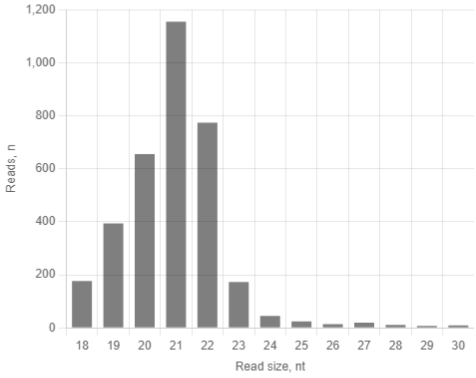

Length distribution of the reads

Mappings of the 3K_1 reads against the viral references.

Dataset	Input reads	Virus	Reference	Mapped reads	Mapped reads, %	RPKM	Forward/Reverse balance	Nonredundant, %	Strain specific	Strain specific, % from total mapped reads	Strain specific, RPKM
3K_1	10000000	SCV	SCV_A	9737	0.097	66.9	0.8	56.6	8443	86.7	58.0

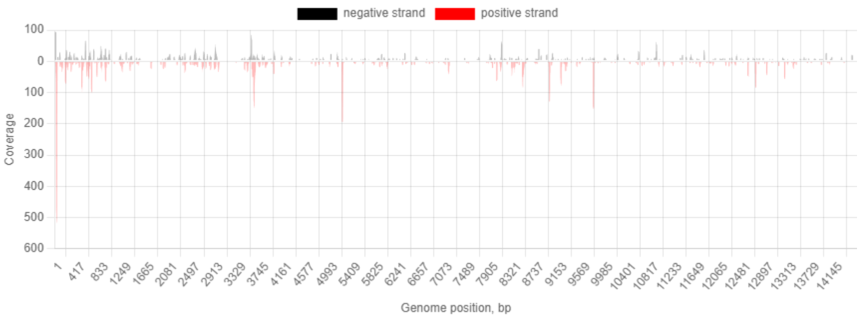

Mapping coverage of all reads matching SCV_A (9737 reads)

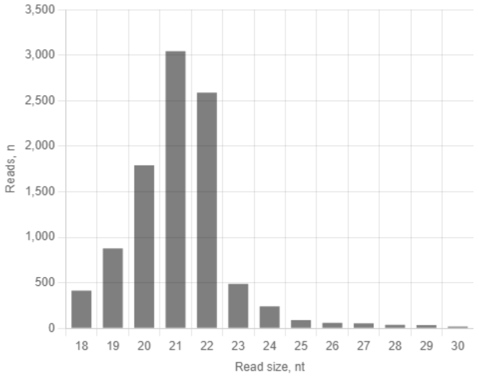

Length distribution of the reads

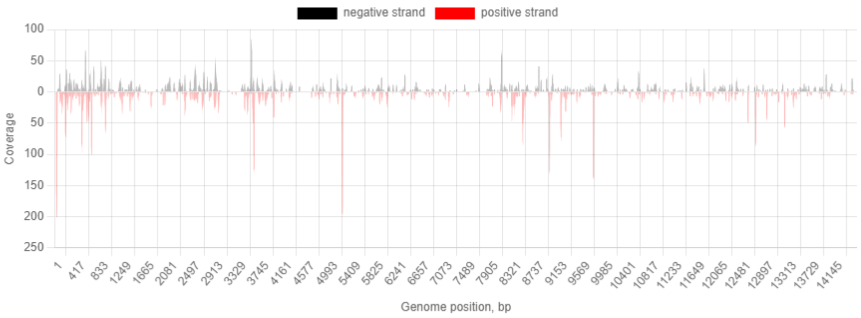

Mapping coverage of SCV_A specific reads (8443 reads)

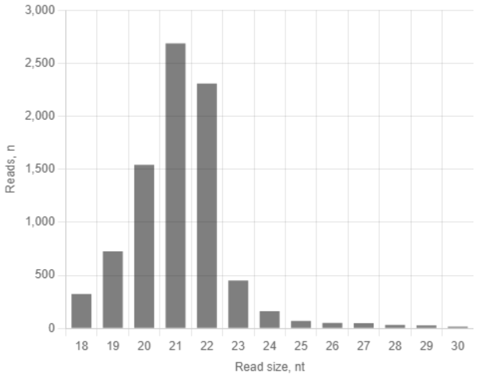

Length distribution of the reads

Dataset	Input reads	Virus	Reference	Mapped reads	Mapped reads, %	RPKM	Forward/Reverse balance	Nonredundant, %	Strain specific	Strain specific, % from total mapped reads	Strain specific, RPKM
3K_1	10000000	SCV	SCV_B	6588	0.066	45.2	0.7	60.1	5294	80.4	36.4

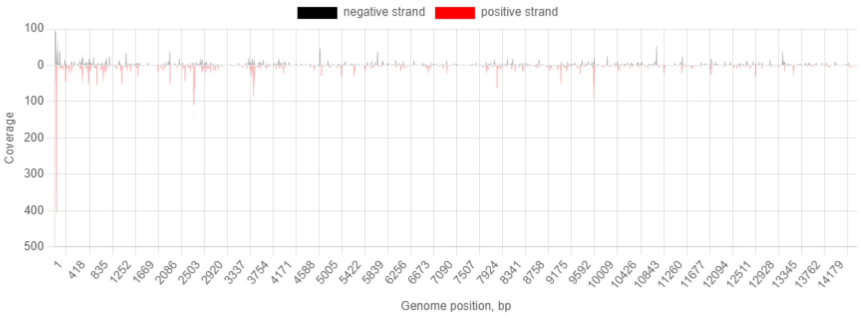

Mapping coverage of all reads matching SCV_B (6588 reads)

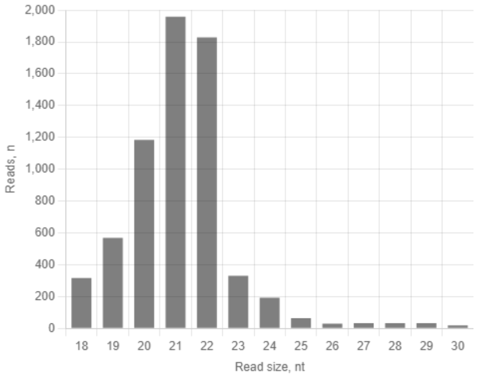

Length distribution of the reads

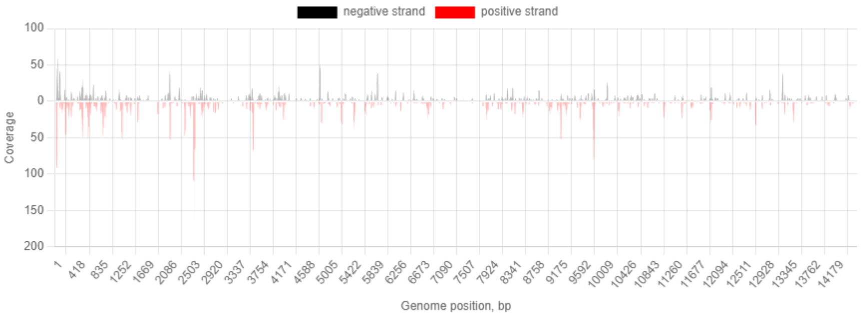

Mapping coverage of SCV_B specific reads (5294 reads)

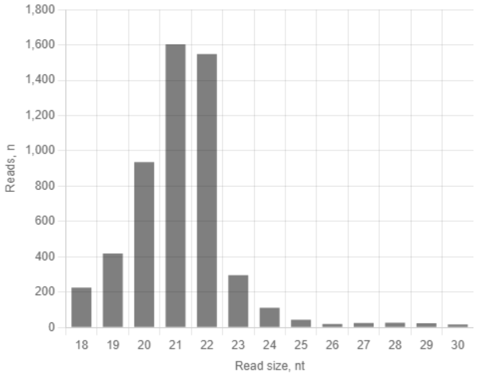

Length distribution of the reads

Dataset	Input reads	Virus	Reference	Mapped reads	Mapped reads, %	RPKM	Forward/Reverse balance	Nonredundant, %	Strain specific	Strain specific, % from total mapped reads	Strain specific, RPKM
3K_1	10000000	SMoV	SMoV_RNA1A	8455	0.085	120.2	1.5	50.1	4731	56.0	67.2

Dataset	Input reads	Virus	Reference	Mapped reads	Mapped reads, %	RPKM	Forward/Reverse balance	Nonredundant, %	Strain specific	Strain specific, % from total mapped reads	Strain specific, RPKM

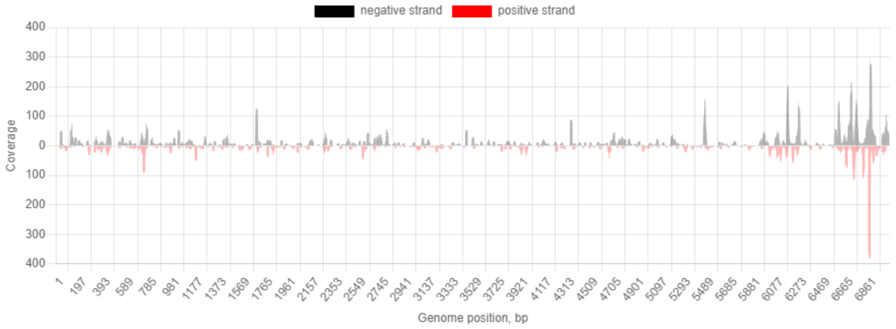

Mapping coverage of all reads matching SMoV_RNA1A (8455 reads)

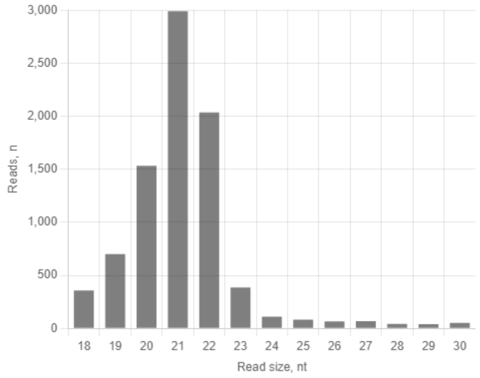

Length distribution of the reads

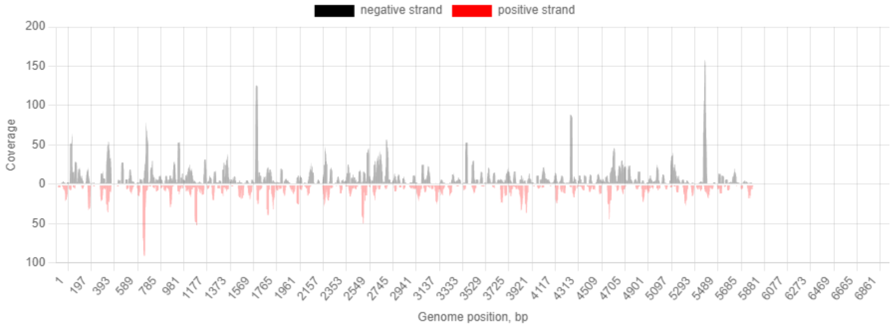

Mapping coverage of SMoV_RNA1A specific reads (4731 reads)

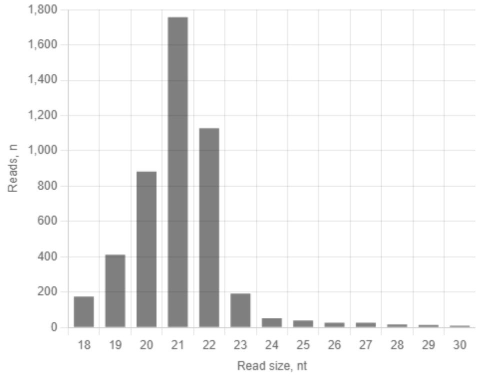

Length distribution of the reads

Dataset	Input reads	Virus	Reference	Mapped reads	Mapped reads, %	RPKM	Forward/Reverse balance	Nonredundant, %	Strain specific	Strain specific, % from total mapped reads	Strain specific, RPKM
3K_1	10000000	SMoV	SMoV_RNA1B	7511	0.075	106.5	1.3	49.5	3850	51.3	54.6

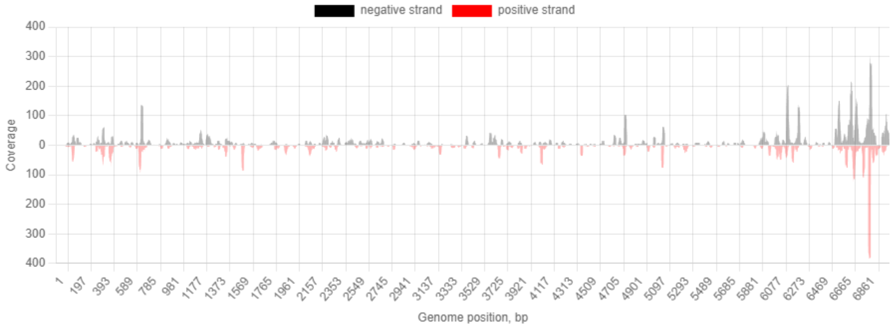

Mapping coverage of all reads matching SMoV_RNA1B (7511 reads)

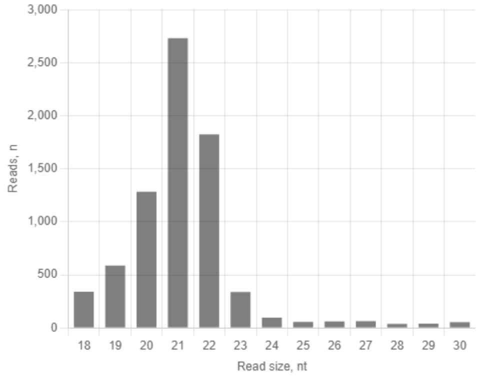

Length distribution of the reads

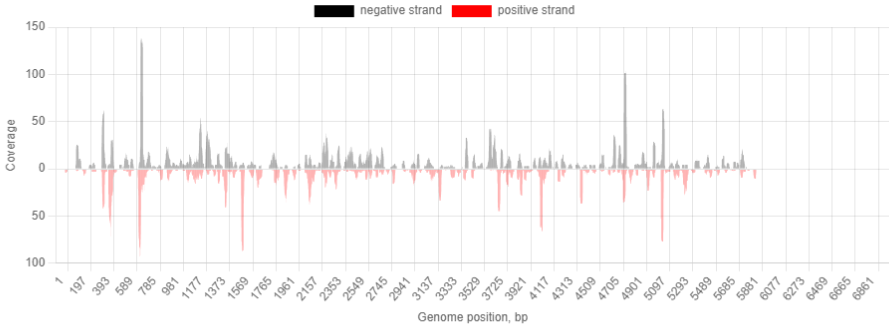

Mapping coverage of SMoV_RNA1B specific reads (3850 reads)

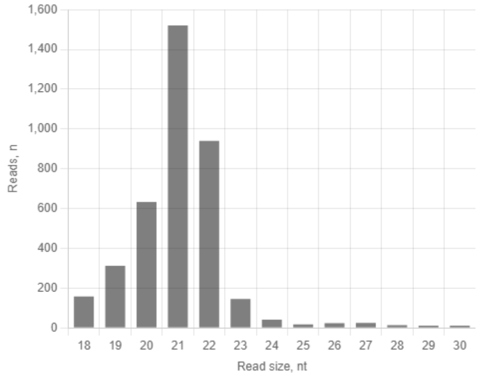

Length distribution of the reads

Dataset	Input reads	Virus	Reference	Mapped reads	Mapped reads, %	RPKM	Forward/Reverse balance	Nonredundant, %	Strain specific	Strain specific, % from total mapped reads	Strain specific, RPKM
3K_1	10000000	SMoV	SMoV_RNA1C	8226	0.082	116.8	1.4	49.2	4522	55.0	64.2

Dataset	Input reads	Virus	Reference	Mapped reads	Mapped reads, %	RPKM	Forward/Reverse balance	Nonredundant, %	Strain specific	Strain specific, % from total mapped reads	Strain specific, RPKM

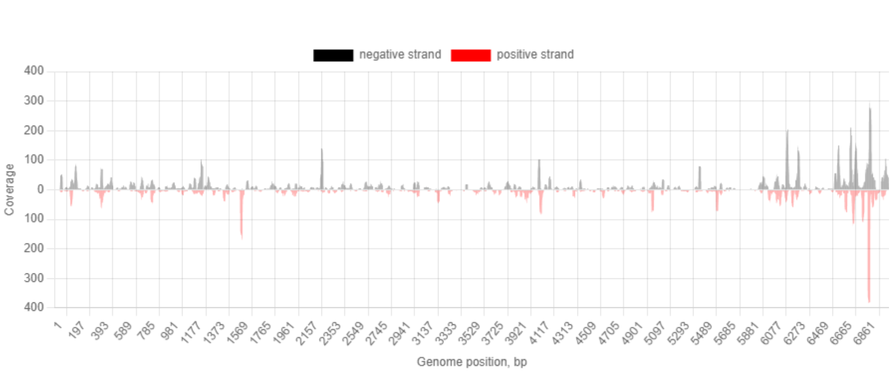

Mapping coverage of all reads matching SMoV_RNA1C (8226 reads)

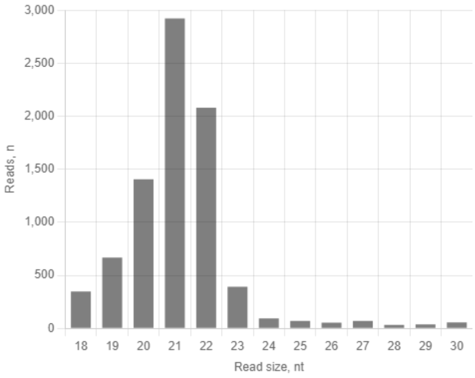

Length distribution of the reads

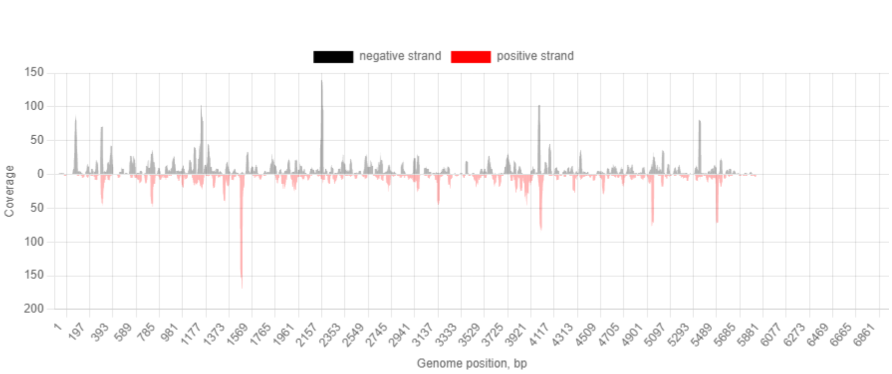

Mapping coverage of SMoV_RNA1C specific reads (4522 reads)

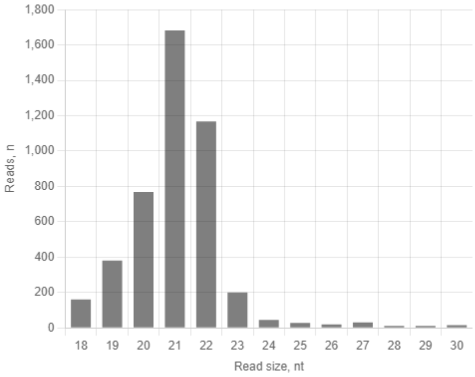

Length distribution of the reads

Dataset	Input reads	Virus	Reference	Mapped reads	Mapped reads, %	RPKM	Forward/Reverse balance	Nonredundant, %	Strain specific	Strain specific, % from total mapped reads	Strain specific, RPKM
3K_1	10000000	SMoV	SMoV_RNA2A	5328	0.053	84.9	1.4	54.6	3543	66.5	56.5

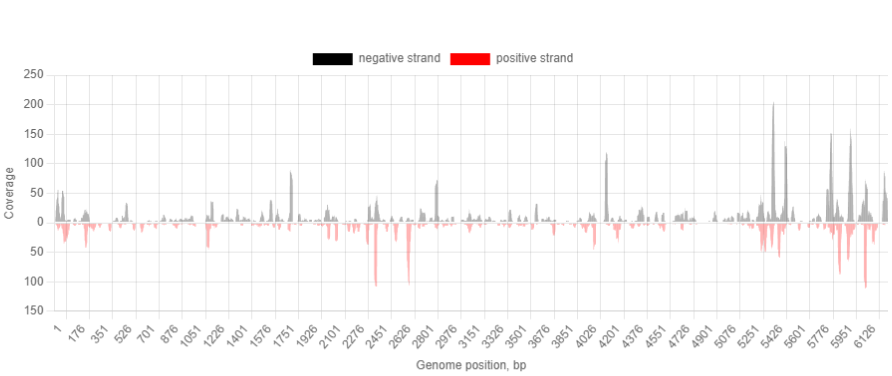

Mapping coverage of all reads matching SMoV_RNA2A (5328 reads)

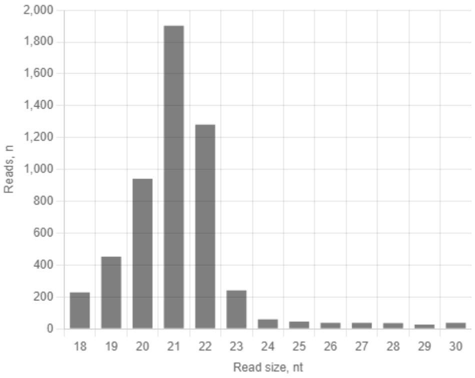

Length distribution of the reads

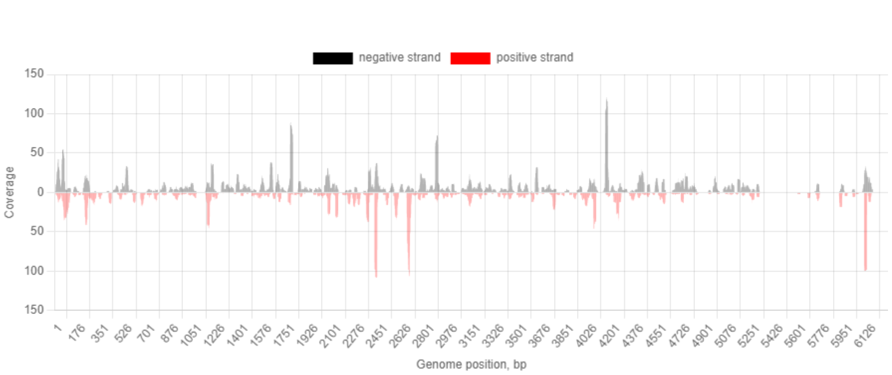

Mapping coverage of SMoV_RNA2A specific reads (3543 reads)

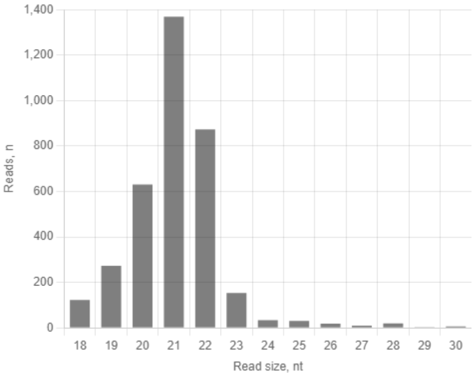

Length distribution of the reads

Dataset	Input reads	Virus	Reference	Mapped reads	Mapped reads, %	RPKM	Forward/Reverse balance	Nonredundant, %	Strain specific	Strain specific, % from total mapped reads	Strain specific, RPKM
3K_1	10000000	SMoV	SMoV_RNA2B	4406	0.044	69.7	1.4	58.6	2383	54.1	37.7

Dataset	Input reads	Virus	Reference	Mapped reads	Mapped reads, %	RPKM	Forward/Reverse balance	Nonredundant, %	Strain specific	Strain specific, % from total mapped reads	Strain specific, RPKM

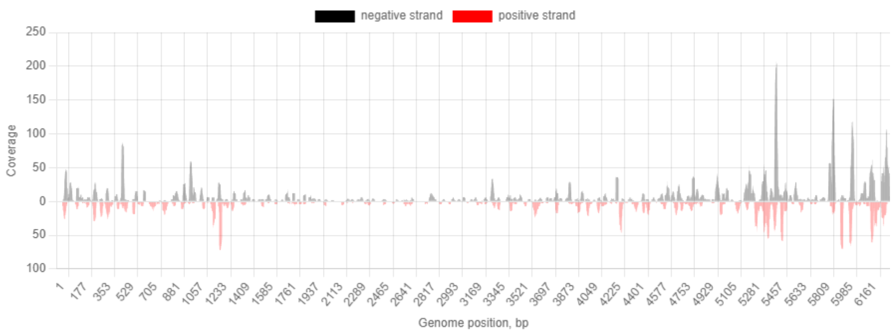

Mapping coverage of all reads matching SMoV_RNA2B (4406 reads)

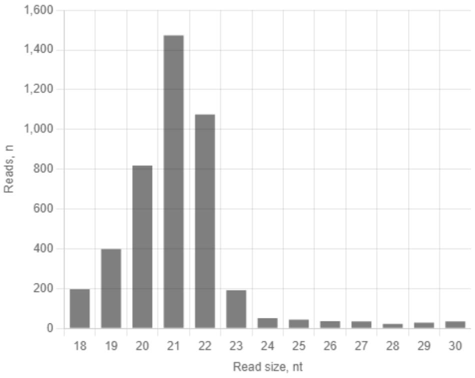

Length distribution of the reads

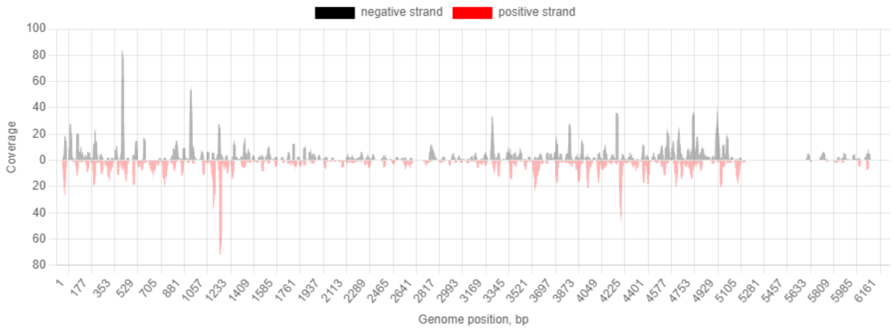

Mapping coverage of SMoV_RNA2B specific reads (2383 reads)

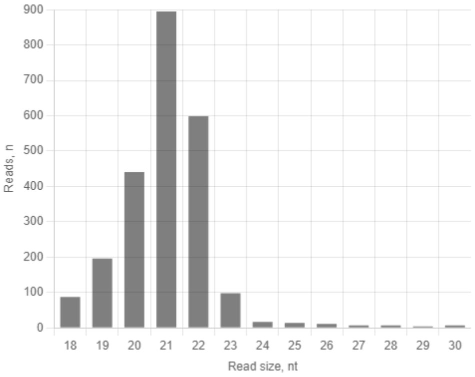

Length distribution of the reads

Dataset	Input reads	Virus	Reference	Mapped reads	Mapped reads, %	RPKM	Forward/Reverse balance	Nonredundant, %	Strain specific	Strain specific, % from total mapped reads	Strain specific, RPKM
3K_1	10000000	SMoV	SMoV_RNA2C	6056	0.061	95.6	1.3	48.5	2702	44.6	42.6

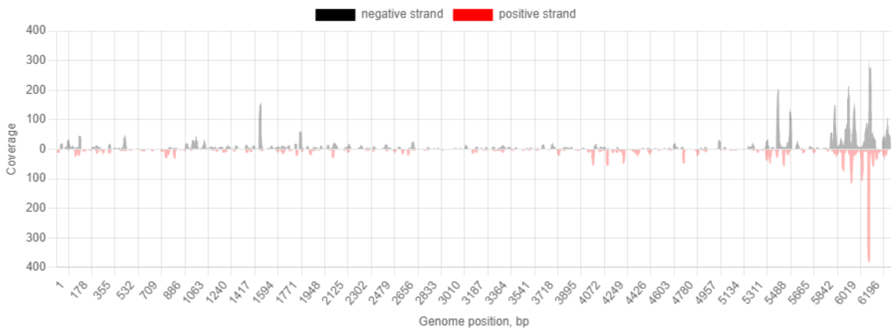

Mapping coverage of all reads matching SMoV_RNA2C (6056 reads)

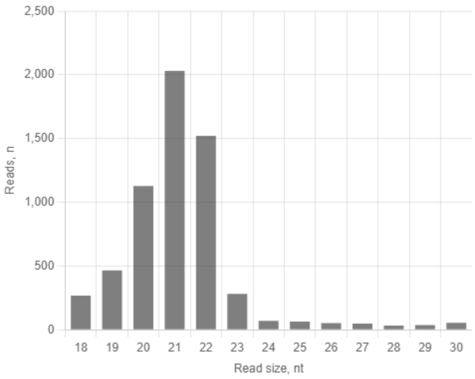

Length distribution of the reads

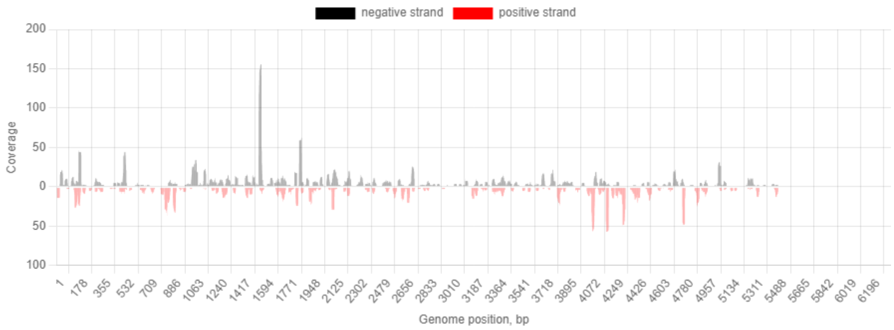

Mapping coverage of SMoV_RNA2C specific reads (2702 reads)

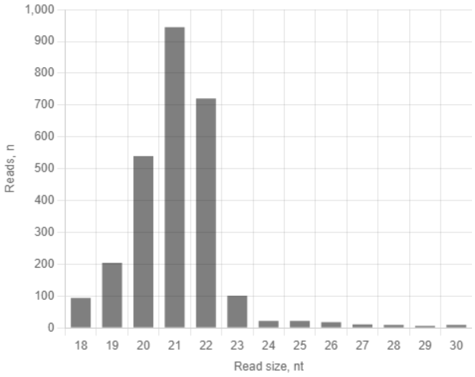

Length distribution of the reads

Dataset	Input reads	Virus	Reference	Mapped reads	Mapped reads, %	RPKM	Forward/Reverse balance	Nonredundant, %	Strain specific	Strain specific, % from total mapped reads	Strain specific, RPKM
3K_1	10000000	StrV1	StrV1_A	2859	0.029	20.2	0.9	67.8	2149	75.2	15.2

Dataset	Input reads	Virus	Reference	Mapped reads	Mapped reads, %	RPKM	Forward/Reverse balance	Nonredundant, %	Strain specific	Strain specific, % from total mapped reads	Strain specific, RPKM

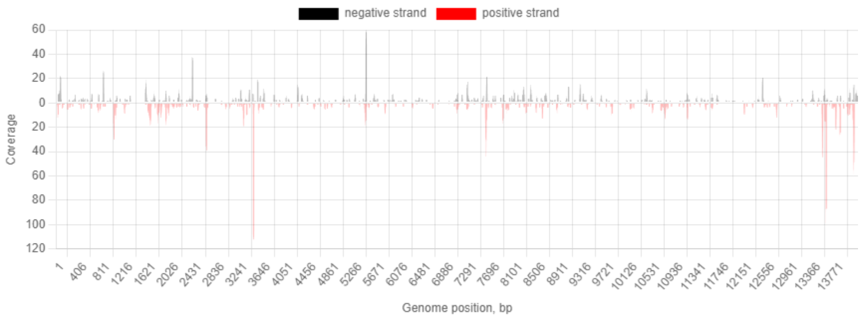

Mapping coverage of all reads matching StrV1_A (2859 reads)

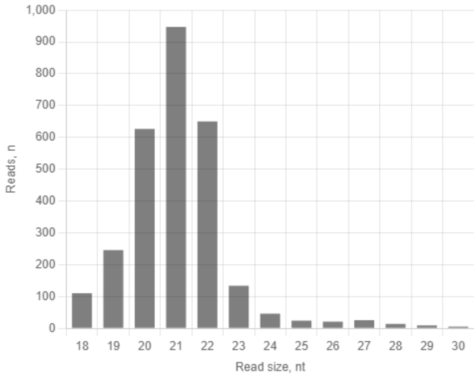

Length distribution of the reads

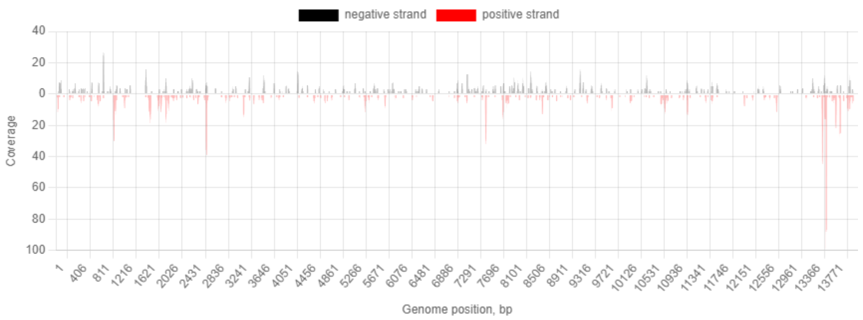

Mapping coverage of StrV1_A specific reads (2149 reads)

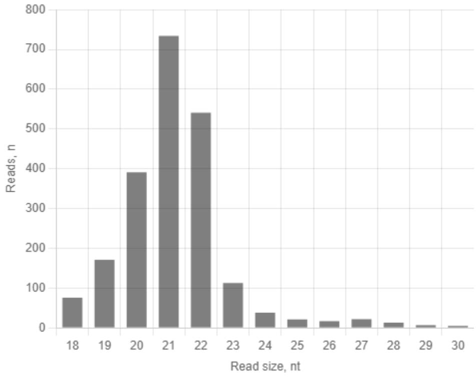

Length distribution of the reads

Dataset	Input reads	Virus	Reference	Mapped reads	Mapped reads, %	RPKM	Forward/Reverse balance	Nonredundant, %	Strain specific	Strain specific, % from total mapped reads	Strain specific, RPKM
3K_1	10000000	StrV1	StrV1_B	7267	0.073	51.8	1.3	40.1	6971	95.9	49.7

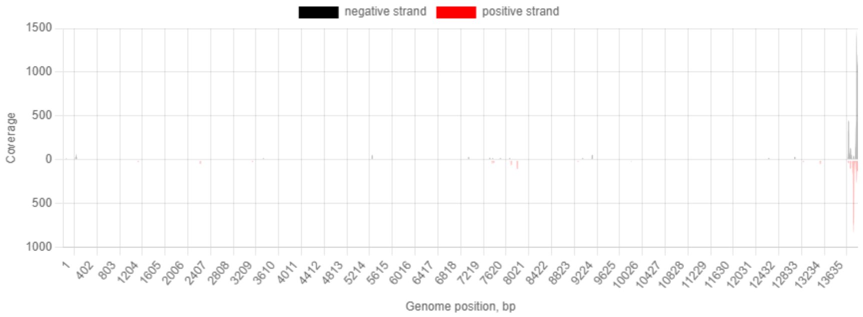

Mapping coverage of all reads matching StrV1_B (7267 reads)

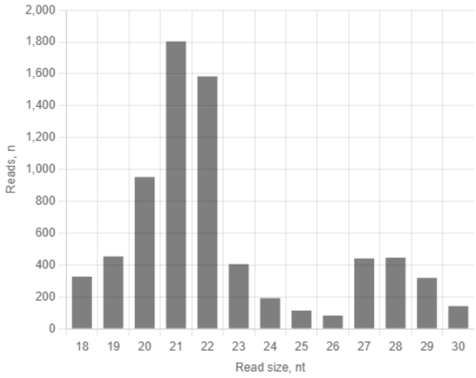

Length distribution of the reads

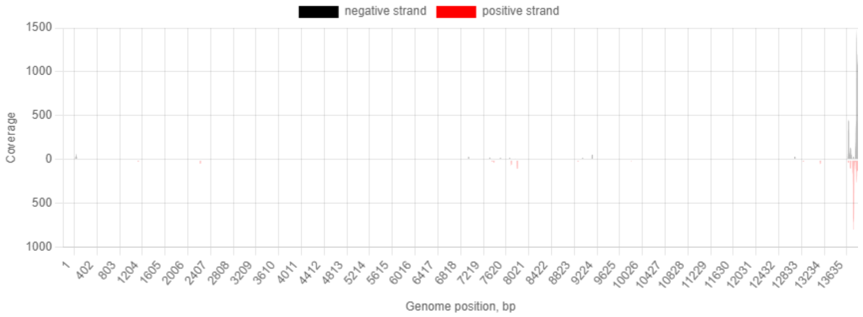

Mapping coverage of StrV1_B specific reads (6971 reads)

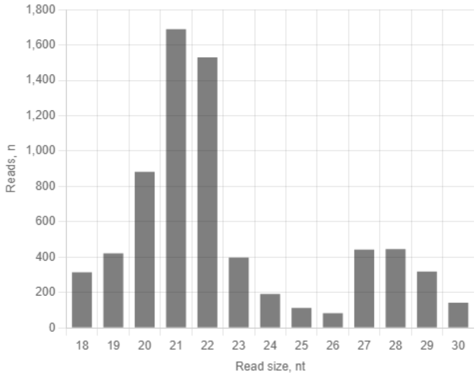

Length distribution of the reads

Dataset	Input reads	Virus	Reference	Mapped reads	Mapped reads, %	RPKM	Forward/Reverse balance	Nonredundant, %	Strain specific	Strain specific, % from total mapped reads	Strain specific, RPKM
3K_1	10000000	StrV1	StrV1_C	2365	0.024	16.6	1.0	70.7	1721	72.8	12.1

Dataset	Input reads	Virus	Reference	Mapped reads	Mapped reads, %	RPKM	Forward/Reverse balance	Nonredundant, %	Strain specific	Strain specific, % from total mapped reads	Strain specific, RPKM

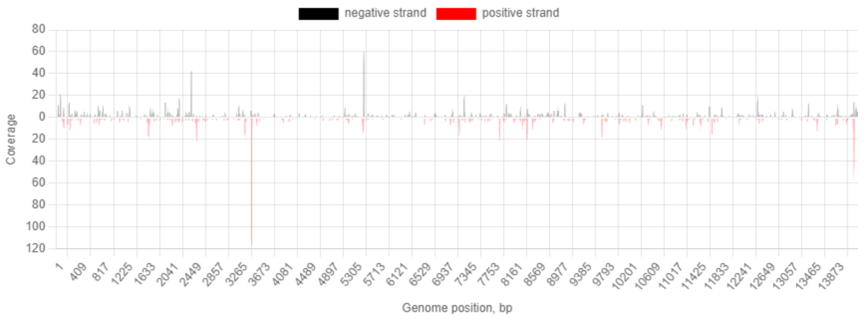

Mapping coverage of all reads matching StrV1_C (2365 reads)

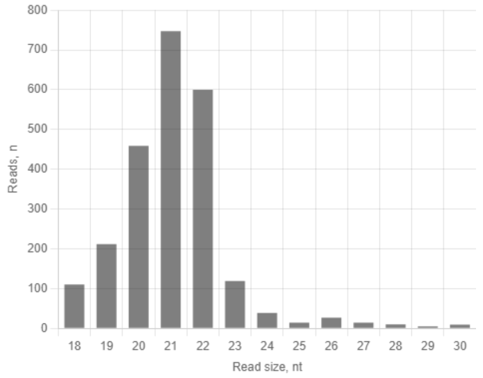

Length distribution of the reads

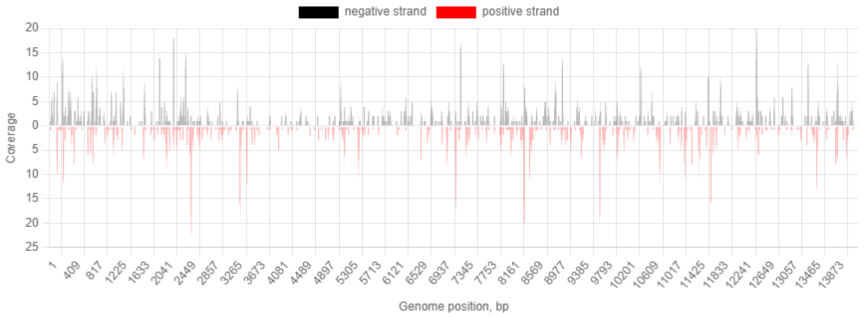

Mapping coverage of StrV1_C specific reads (1721 reads)

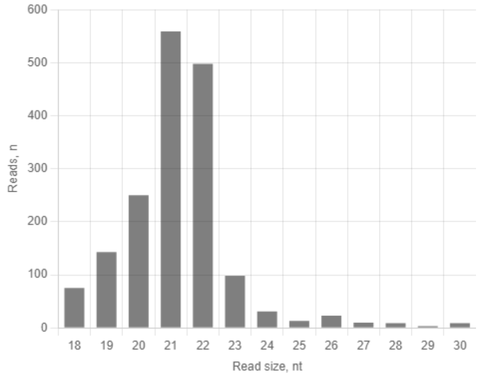

Length distribution of the reads

Mappings of the 3K_2 reads against the viral references.

Dataset	Input reads	Virus	Reference	Mapped reads	Mapped reads, %	RPKM	Forward/Reverse balance	Nonredundant, %	Strain specific	Strain specific, % from total mapped reads	Strain specific, RPKM
3K_2	10000000	SCV	SCV_A	11218	0.112	77.1	0.9	58.0	8443	75.3	66.5

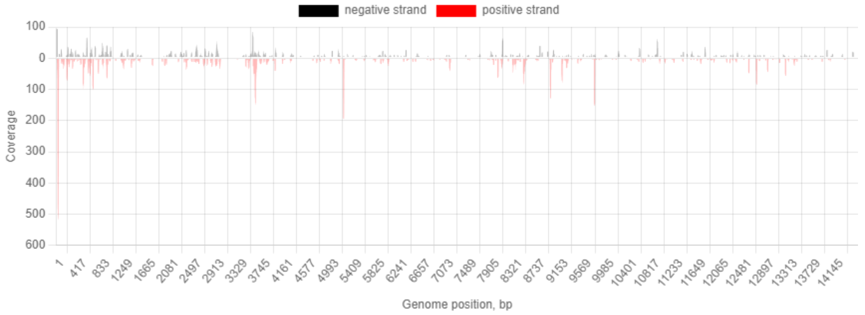

Mapping coverage of all reads matching SCV_A (9737 reads)

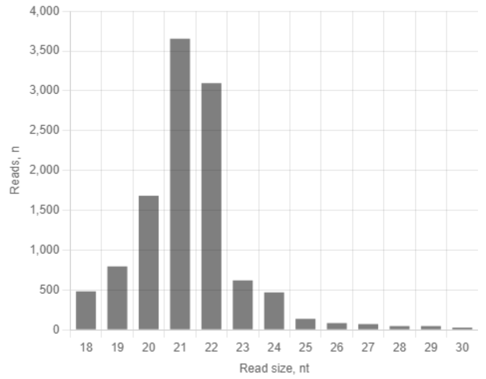

Length distribution of the reads

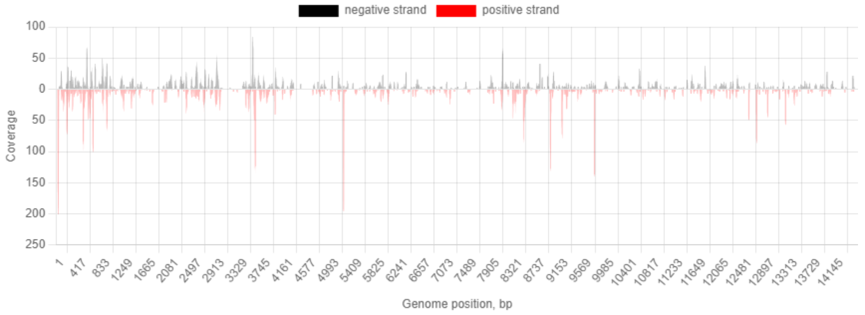

Mapping coverage of SCV_A specific reads (8443 reads)

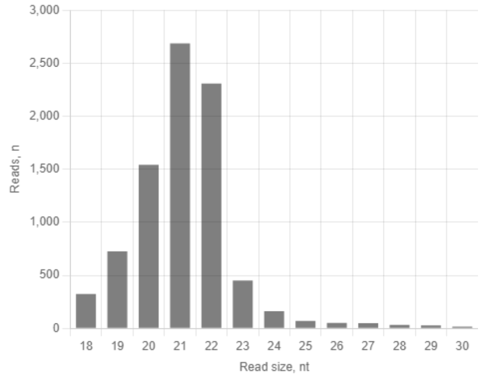

Length distribution of the reads

Dataset	Input reads	Virus	Reference	Mapped reads	Mapped reads, %	RPKM	Forward/Reverse balance	Nonredundant, %	Strain specific	Strain specific, % from total mapped reads	Strain specific, RPKM
3K_2	10000000	SCV	SCV_B	7197	0.072	49.4	0.8	61.9	5294	73.6	38.8

Dataset	Input reads	Virus	Reference	Mapped reads	Mapped reads, %	RPKM	Forward/Reverse balance	Nonredundant, %	Strain specific	Strain specific, % from total mapped reads	Strain specific, RPKM

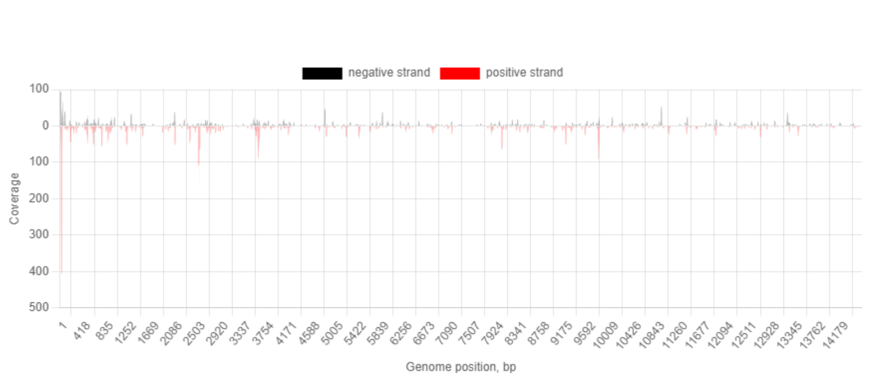

Mapping coverage of all reads matching SCV_B (6588 reads)

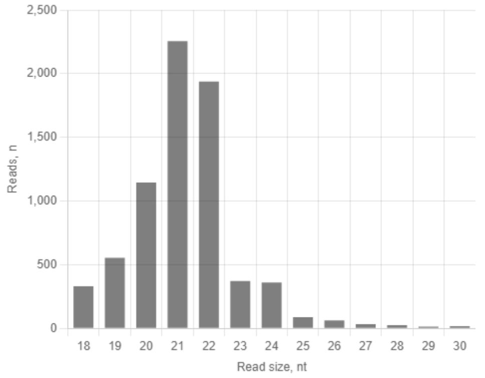

Length distribution of the reads

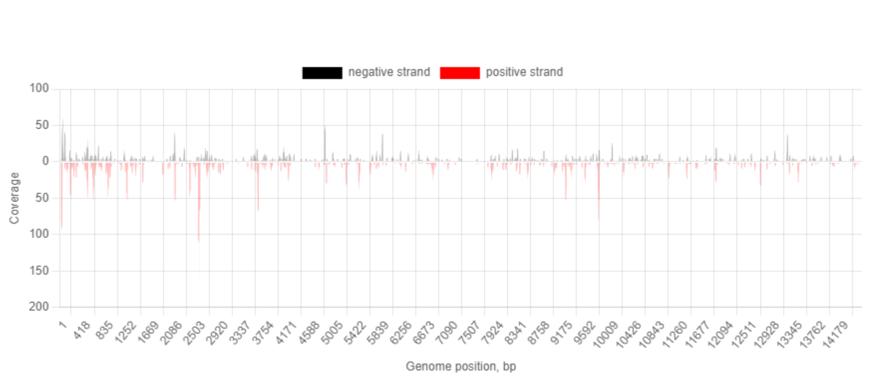

Mapping coverage of SCV_B specific reads (5294 reads)

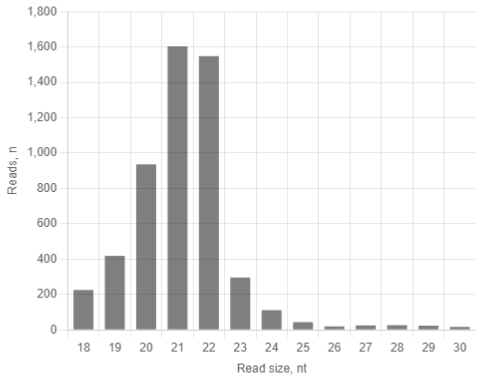

Length distribution of the reads

Dataset	Input reads	Virus	Reference	Mapped reads	Mapped reads, %	RPKM	Forward/Reverse balance	Nonredundant, %	Strain specific	Strain specific, % from total mapped reads	Strain specific, RPKM
3K_2	10000000	SMoV	SMoV_RNA1A	9058	0.091	128.8	1.3	51.9	4731	52.2	73.8

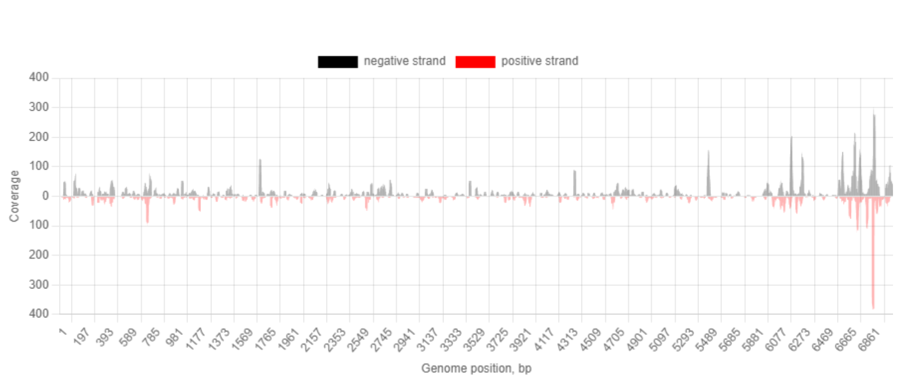

Mapping coverage of all reads matching SMoV_RNA1A (8455 reads)

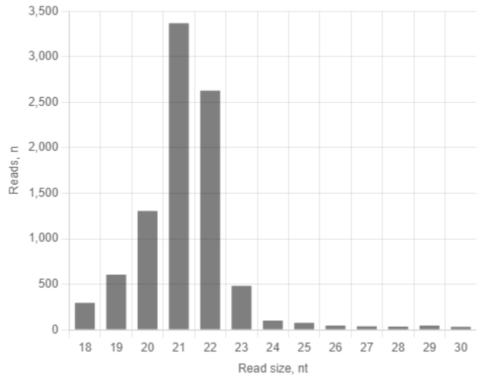

Length distribution of the reads

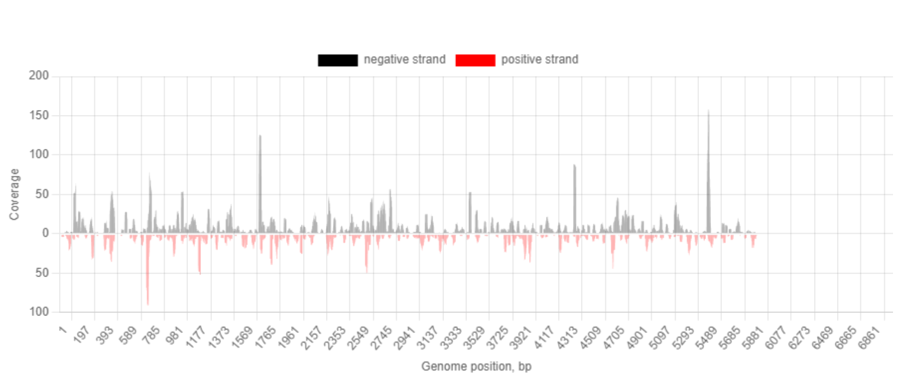

Mapping coverage of SMoV_RNA1A specific reads (4731 reads)

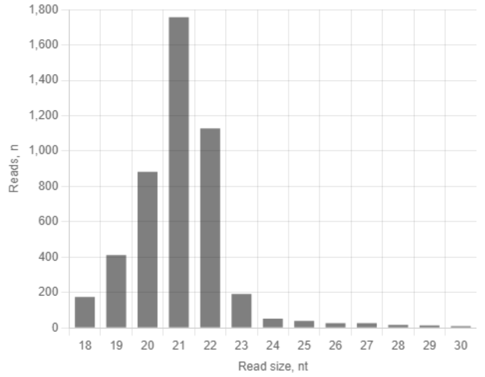

Length distribution of the reads

Dataset	Input reads	Virus	Reference	Mapped reads	Mapped reads, %	RPKM	Forward/Reverse balance	Nonredundant, %	Strain specific	Strain specific, % from total mapped reads	Strain specific, RPKM
3K_2	10000000	SMoV	SMoV_RNA1B	7667	0.077	108.8	1.2	51.9	3850	50.2	55.3

Dataset	Input reads	Virus	Reference	Mapped reads	Mapped reads, %	RPKM	Forward/Reverse balance	Nonredundant, %	Strain specific	Strain specific, % from total mapped reads	Strain specific, RPKM

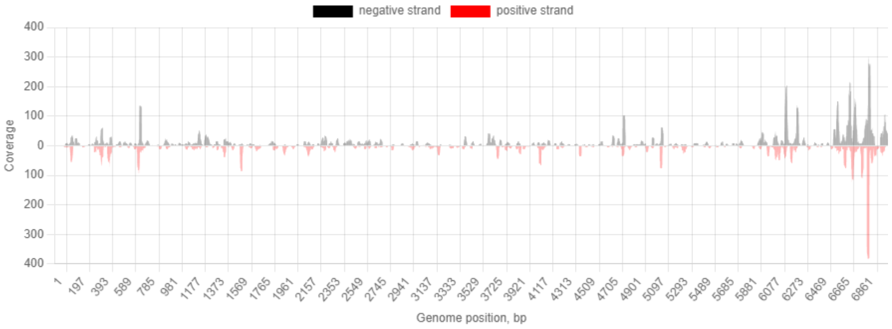

Mapping coverage of all reads matching SMoV_RNA1B (7511 reads)

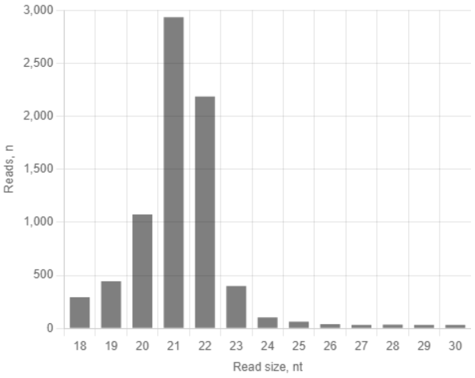

Length distribution of the reads

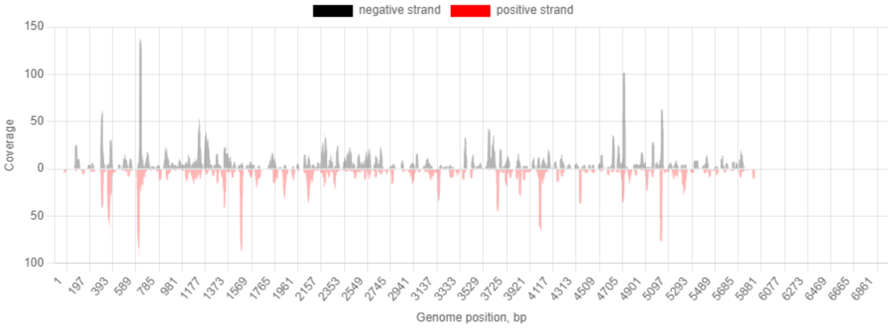

Mapping coverage of SMoV_RNA1B specific reads (3850 reads)

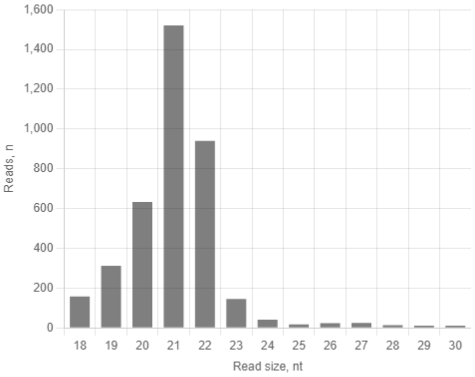

Length distribution of the reads

Dataset	Input reads	Virus	Reference	Mapped reads	Mapped reads, %	RPKM	Forward/Reverse balance	Nonredundant, %	Strain specific	Strain specific, % from total mapped reads	Strain specific, RPKM
3K_2	10000000	SMoV	SMoV_RNA1C	8292	0.083	117.8	1.2	52.5	4522	54.5	62.9

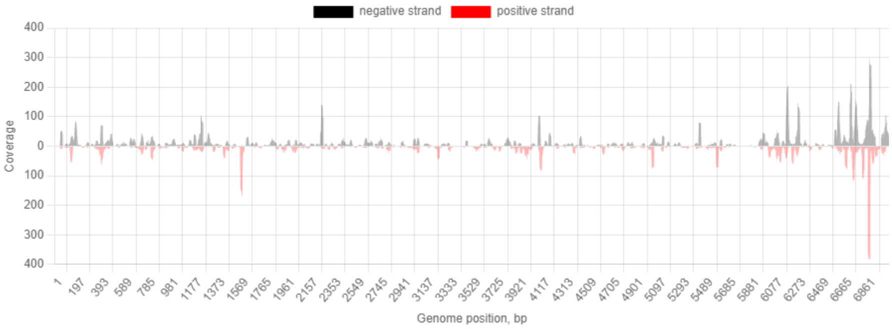

Mapping coverage of all reads matching SMoV_RNA1C (8226 reads)

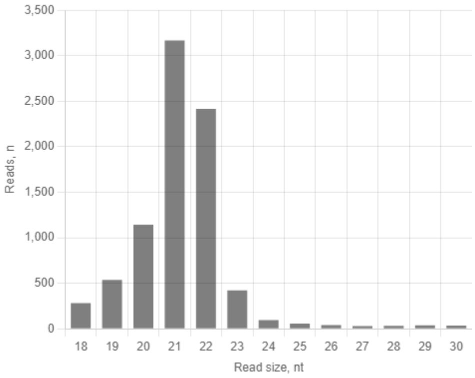

Length distribution of the reads

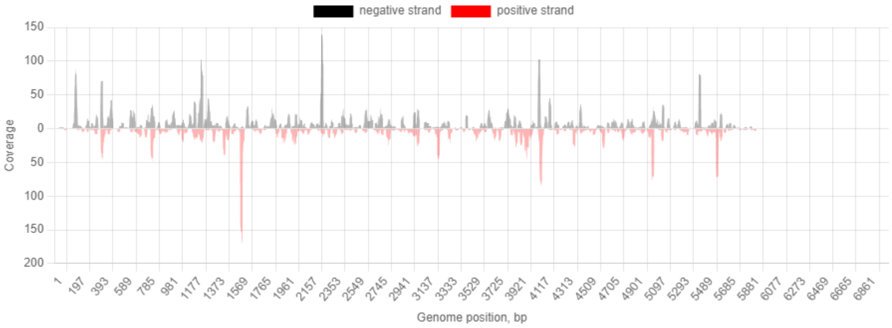

Mapping coverage of SMoV_RNA1C specific reads (4522 reads)

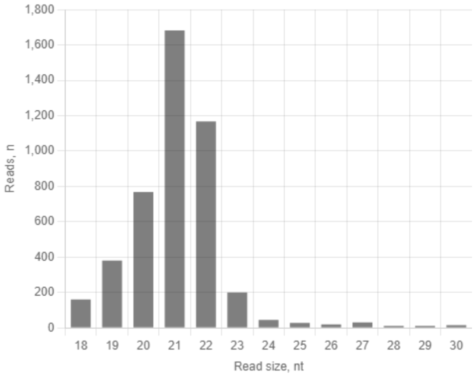

Length distribution of the reads

Dataset	Input reads	Virus	Reference	Mapped reads	Mapped reads, %	RPKM	Forward/Reverse balance	Nonredundant, %	Strain specific	Strain specific, % from total mapped reads	Strain specific, RPKM
3K_2	10000000	SMoV	SMoV_RNA2A	5438	0.054	86.8	1.4	56.8	3543	65.2	58.7

Dataset	Input reads	Virus	Reference	Mapped reads	Mapped reads, %	RPKM	Forward/Reverse balance	Nonredundant, %	Strain specific	Strain specific, % from total mapped reads	Strain specific, RPKM

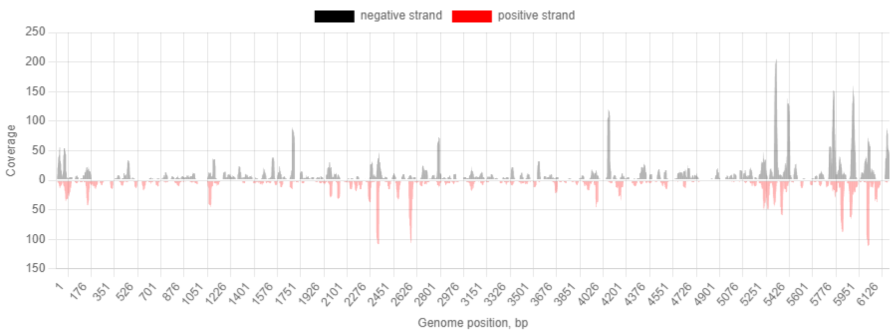

Mapping coverage of all reads matching SMoV_RNA2A (5328 reads)

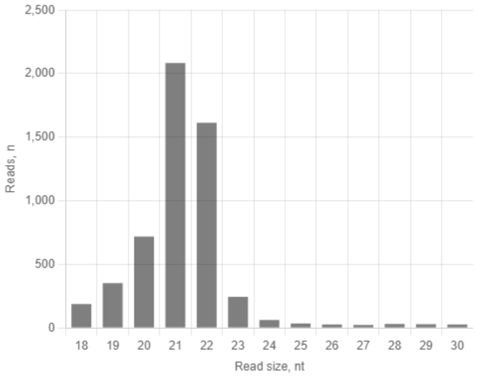

Length distribution of the reads

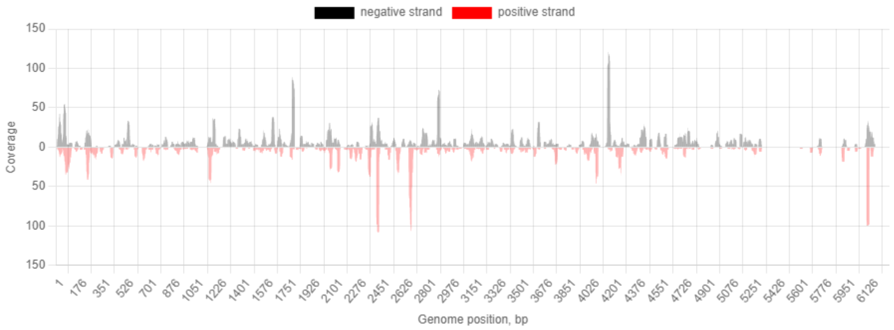

Mapping coverage of SMoV_RNA2A specific reads (3543 reads)

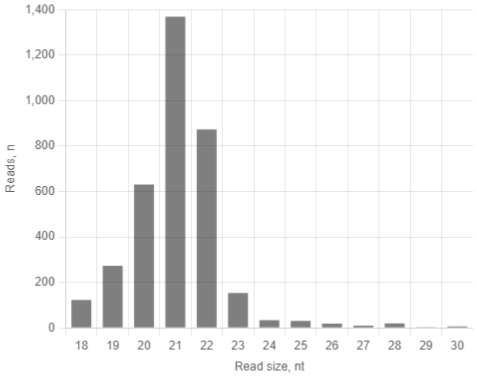

Length distribution of the reads

Dataset	Input reads	Virus	Reference	Mapped reads	Mapped reads, %	RPKM	Forward/Reverse balance	Nonredundant, %	Strain specific	Strain specific, % from total mapped reads	Strain specific, RPKM
3K_2	10000000	SMoV	SMoV_RNA2B	4696	0.047	74.3	1.4	60.7	2383	50.7	41.6

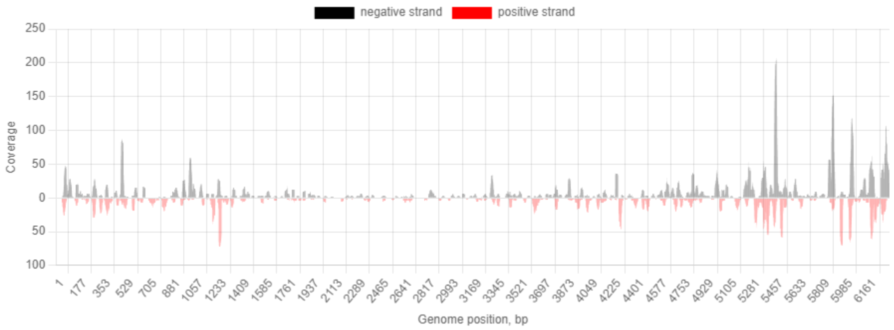

Mapping coverage of all reads matching SMoV_RNA2B (4406 reads)

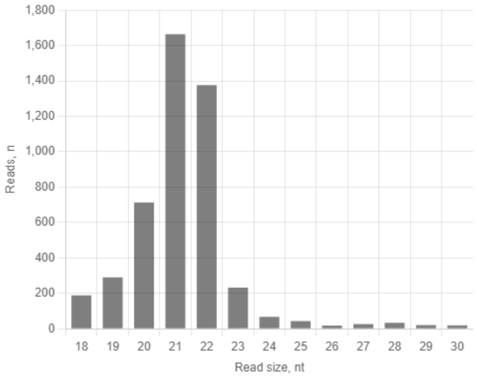

Length distribution of the reads

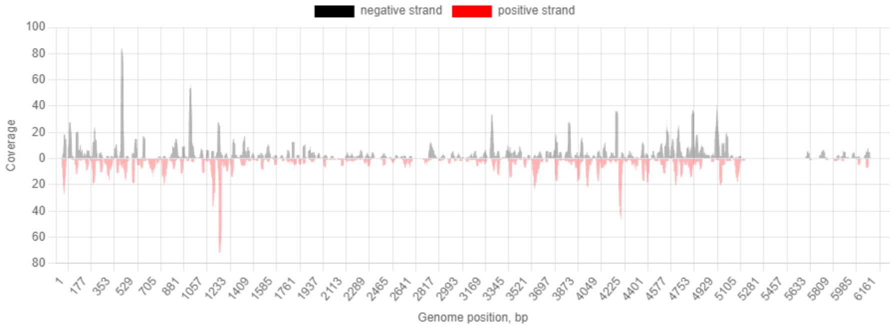

Mapping coverage of SMoV_RNA2B specific reads (2383 reads)

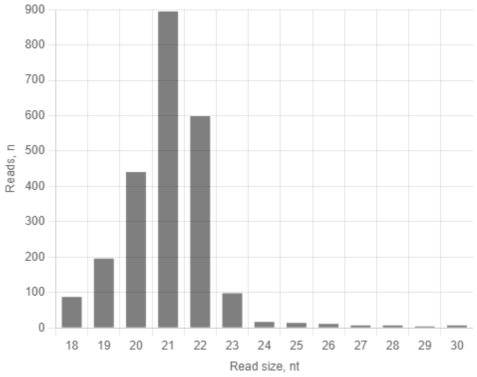

Length distribution of the reads

Dataset	Input reads	Virus	Reference	Mapped reads	Mapped reads, %	RPKM	Forward/Reverse balance	Nonredundant, %	Strain specific	Strain specific, % from total mapped reads	Strain specific, RPKM
3K_2	10000000	SMoV	SMoV_RNA2C	6121	0.061	96.6	1.2	49.8	2702	44.1	42.5

Dataset	Input reads	Virus	Reference	Mapped reads	Mapped reads, %	RPKM	Forward/Reverse balance	Nonredundant, %	Strain specific	Strain specific, % from total mapped reads	Strain specific, RPKM

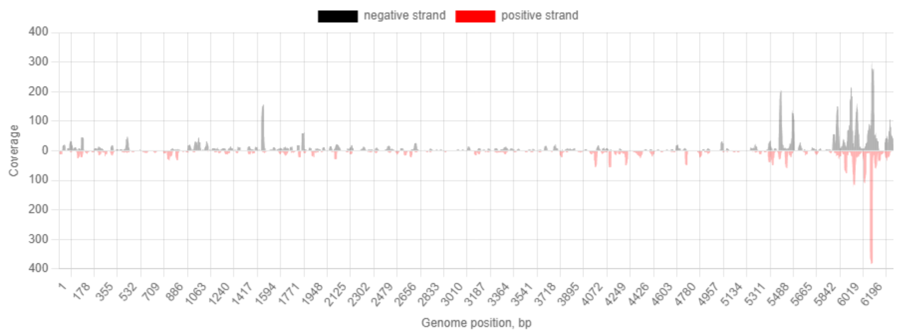

Mapping coverage of **all reads** matching SMoV_RNA2C (6056 reads)

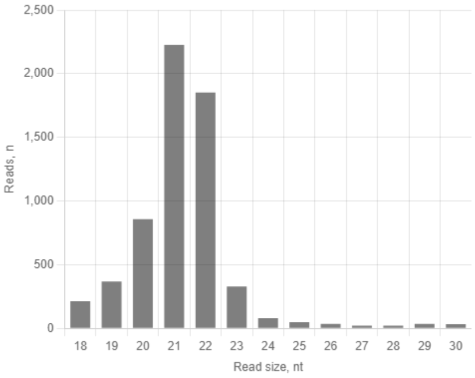

Length distribution of the reads

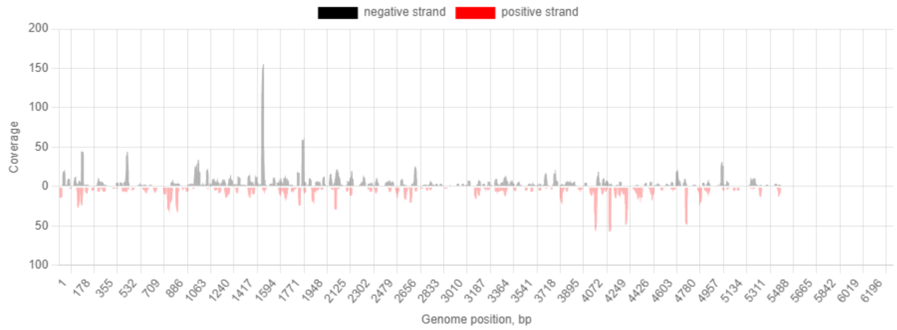

Mapping coverage of SMoV_RNA2C **specific reads** (2702 reads)

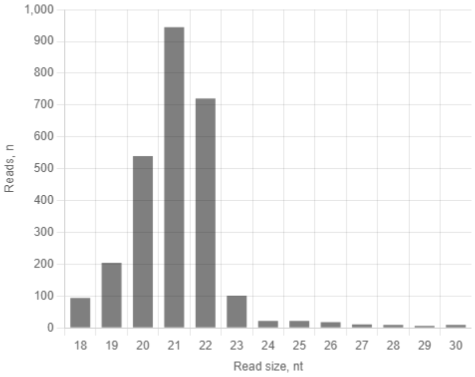

Length distribution of the reads

Dataset	Input reads	Virus	Reference	Mapped reads	Mapped reads, %	RPKM	Forward/Reverse balance	Nonredundant, %	Strain specific	Strain specific, % from total mapped reads	Strain specific, RPKM
3K_2	10000000	StrV1	StrV1_A	3940	0.039	27.8	1.0	70.2	2149	54.5	22.1

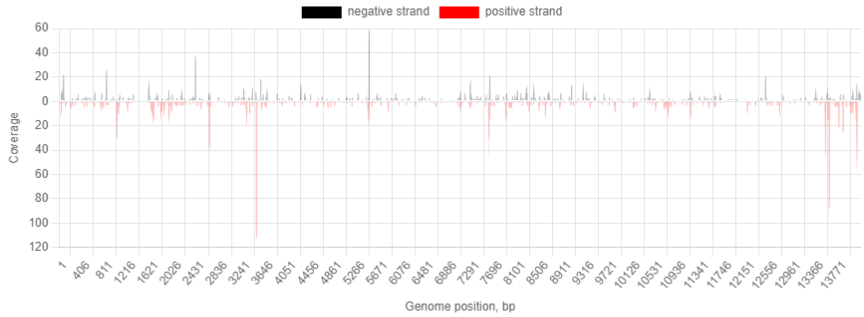

Mapping coverage of **all reads** matching StrV1_A (2859 reads)

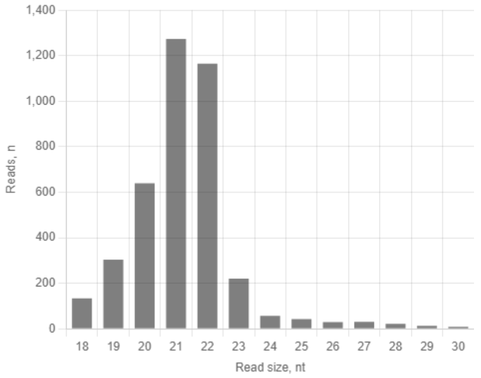

Length distribution of the reads

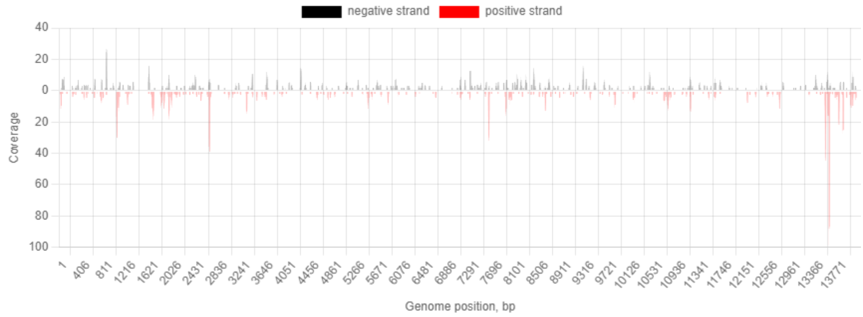

Mapping coverage of StrV1_A **specific reads** (2149 reads)

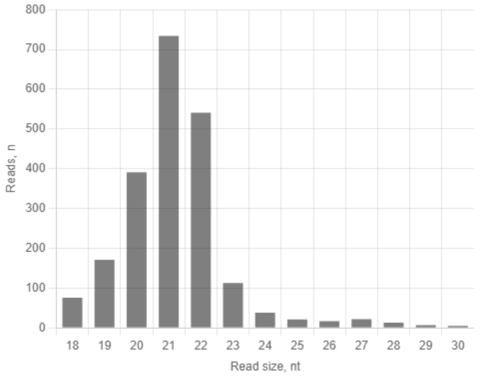

Length distribution of the reads

Dataset	Input reads	Virus	Reference	Mapped reads	Mapped reads, %	RPKM	Forward/Reverse balance	Nonredundant, %	Strain specific	Strain specific, % from total mapped reads	Strain specific, RPKM
3K_2	10000000	StrV1	StrV1_B	11149	0.111	79.5	1.3	42.2	6971	62.5	76.6

Dataset	Input reads	Virus	Reference	Mapped reads	Mapped reads, %	RPKM	Forward/Reverse balance	Nonredundant, %	Strain specific	Strain specific, % from total mapped reads	Strain specific, RPKM

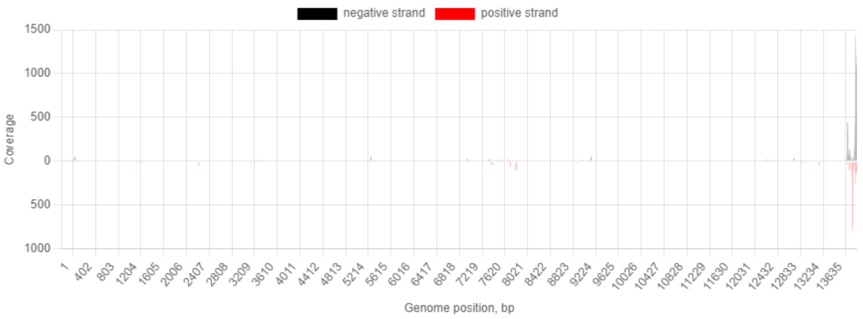

Mapping coverage of **all reads** matching StrV1_B (7267 reads)

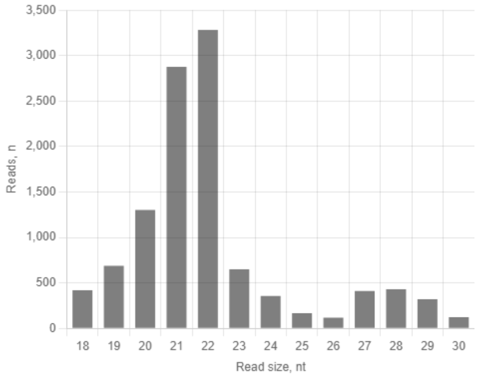

Length distribution of the reads

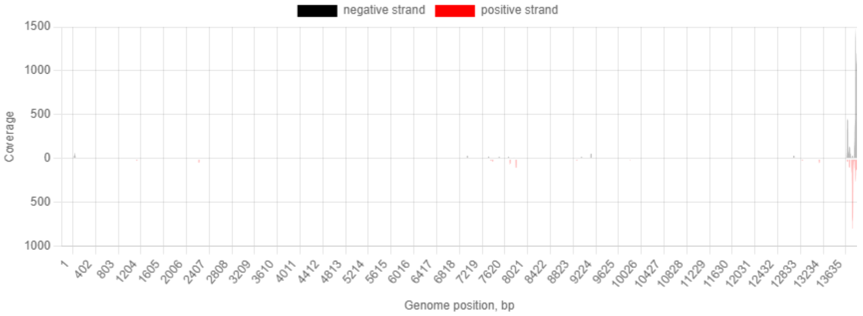

Mapping coverage of StrV1_B **specific reads** (6971 reads)

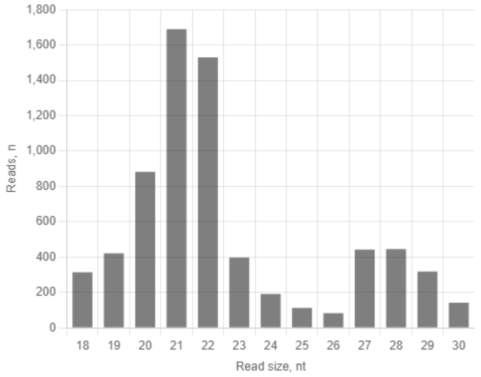

Length distribution of the reads

Dataset	Input reads	Virus	Reference	Mapped reads	Mapped reads, %	RPKM	Forward/Reverse balance	Nonredundant, %	Strain specific	Strain specific, % from total mapped reads	Strain specific, RPKM
3K_2	10000000	StrV1	StrV1_C	2759	0.028	19.4	1.1	72.4	1721	62.4	13.8

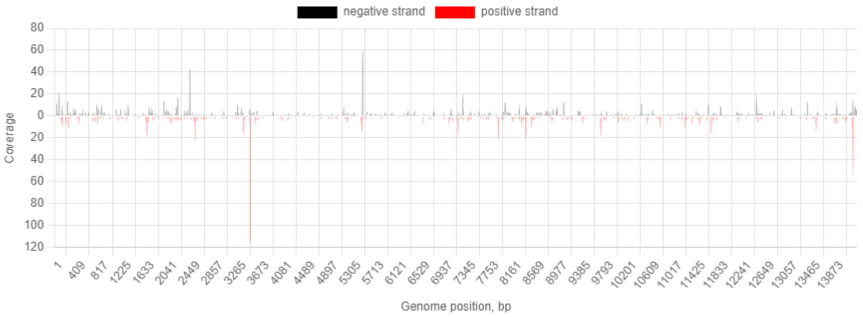

Mapping coverage of **all reads** matching StrV1_C (2365 reads)

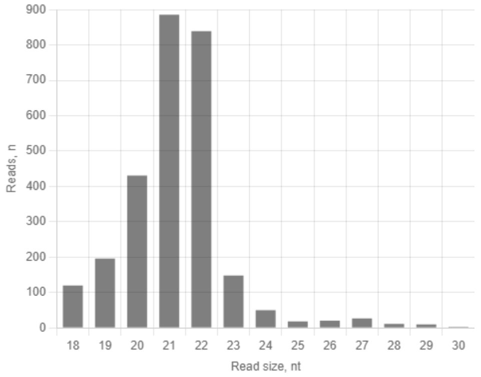

Length distribution of the reads

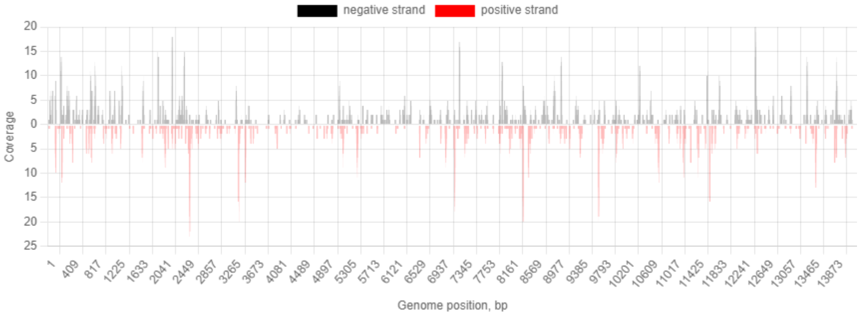

Mapping coverage of StrV1_C **specific reads** (1721 reads)

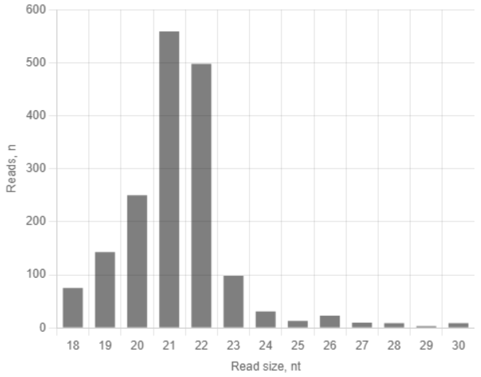

Length distribution of the reads

Mappings of the 3K_3 reads against the viral references.

Dataset	Input reads	Virus	Reference	Mapped reads	Mapped reads, %	RPKM	Forward/Reverse balance	Nonredundant, %	Strain specific	Strain specific, % from total mapped reads	Strain specific, RPKM
3K_3	10000000	SCV	SCV_A	13890	0.139	95.5	1.0	47.3	8443	60.8	84.7

Dataset	Input reads	Virus	Reference	Mapped reads	Mapped reads, %	RPKM	Forward/Reverse balance	Nonredundant, %	Strain specific	Strain specific, % from total mapped reads	Strain specific, RPKM

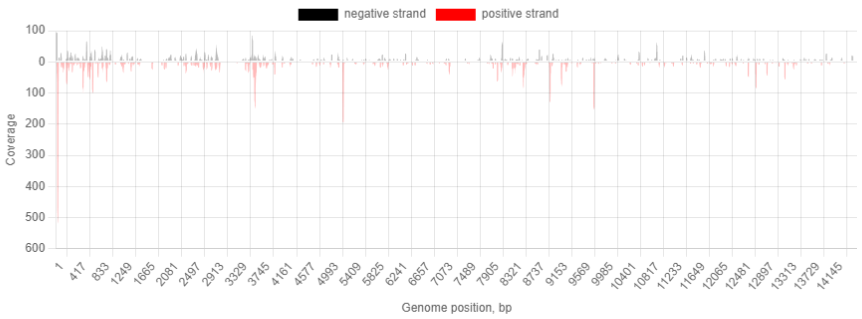

Mapping coverage of all reads matching SCV_A (9737 reads)

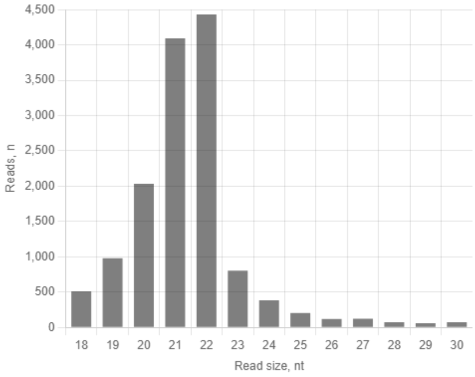

Length distribution of the reads

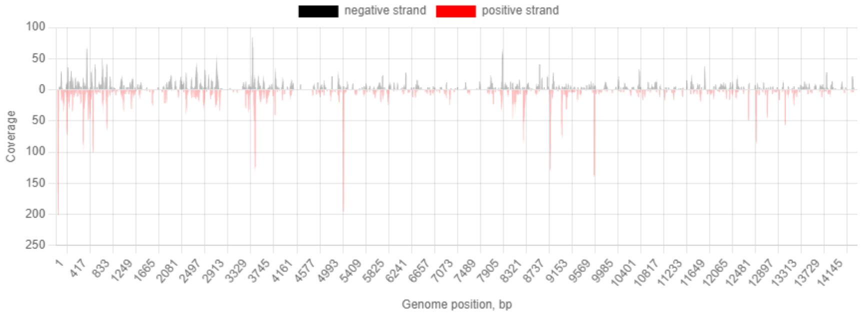

Mapping coverage of SCV_A specific reads (8443 reads)

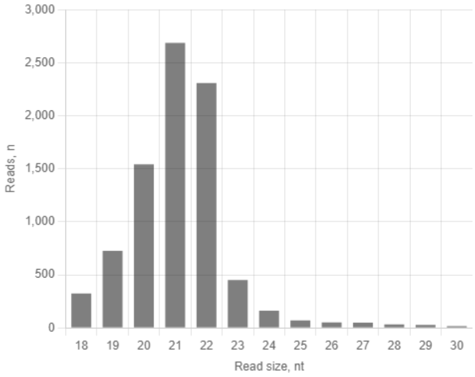

Length distribution of the reads

Dataset	Input reads	Virus	Reference	Mapped reads	Mapped reads, %	RPKM	Forward/Reverse balance	Nonredundant, %	Strain specific	Strain specific, % from total mapped reads	Strain specific, RPKM
3K_3	10000000	SCV	SCV_B	6567	0.066	45.1	1.0	52.9	5294	80.6	34.3

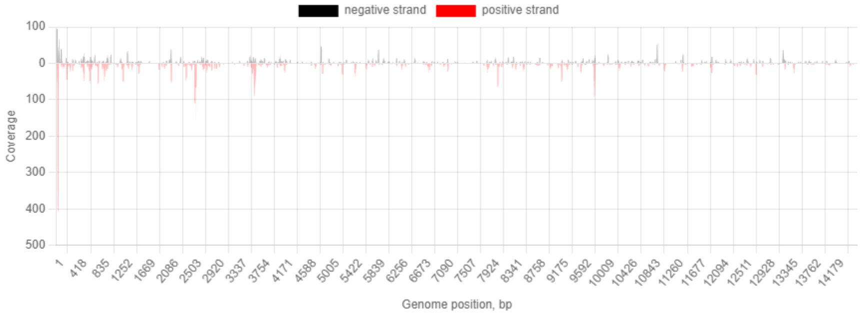

Mapping coverage of all reads matching SCV_B (6588 reads)

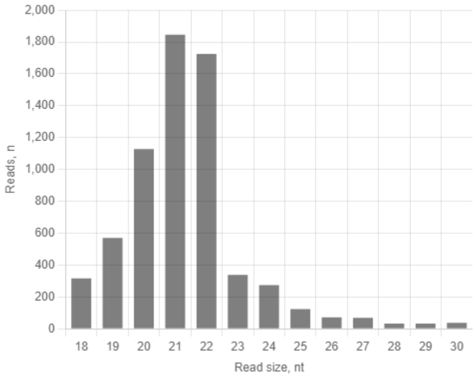

Length distribution of the reads

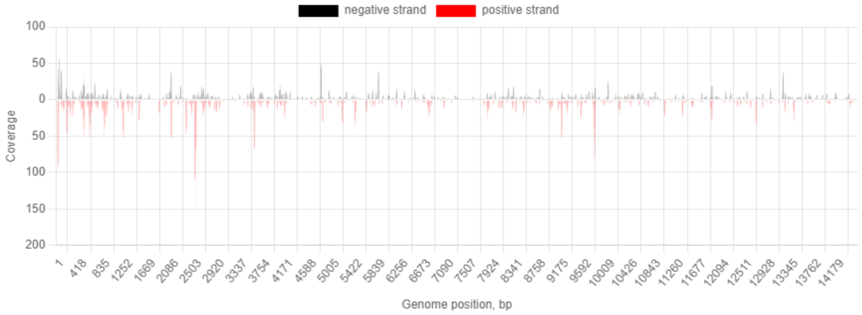

Mapping coverage of SCV_B specific reads (5294 reads)

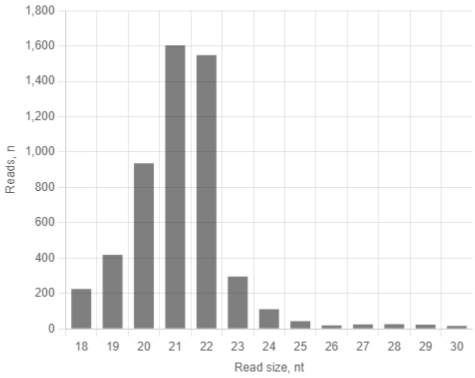

Length distribution of the reads

Dataset	Input reads	Virus	Reference	Mapped reads	Mapped reads, %	RPKM	Forward/Reverse balance	Nonredundant, %	Strain specific	Strain specific, % from total mapped reads	Strain specific, RPKM
3K_3	10000000	SMoV	SMoV_RNA1A	5642	0.056	80.1	1.7	48.8	4731	83.9	43.3

Dataset	Input reads	Virus	Reference	Mapped reads	Mapped reads, %	RPKM	Forward/Reverse balance	Nonredundant, %	Strain specific	Strain specific, % from total mapped reads	Strain specific, RPKM

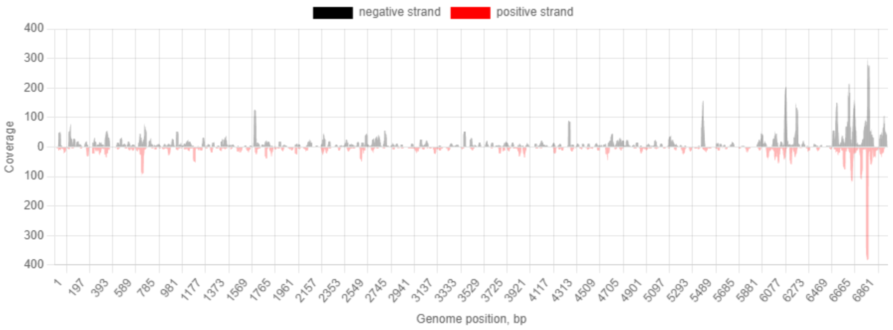

Mapping coverage of all reads matching SMoV_RNA1A (8455 reads)

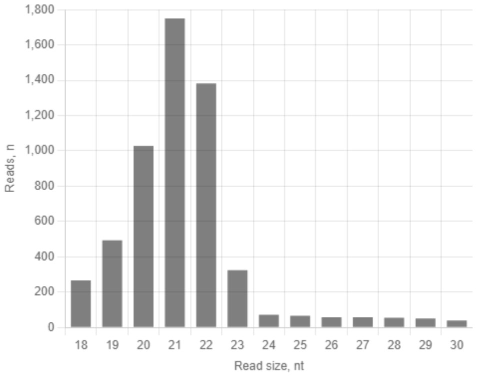

Length distribution of the reads

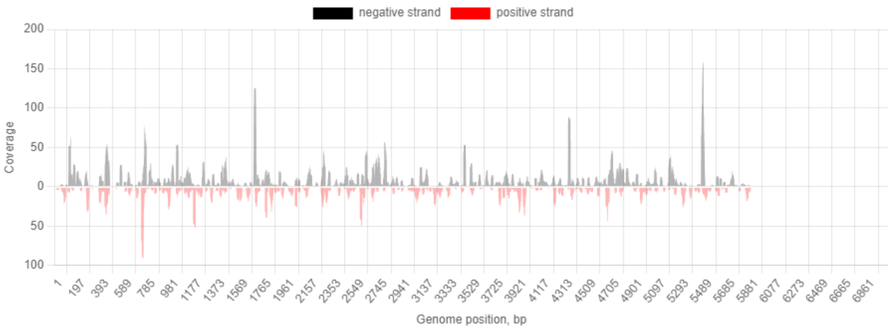

Mapping coverage of SMoV_RNA1A specific reads (4731 reads)

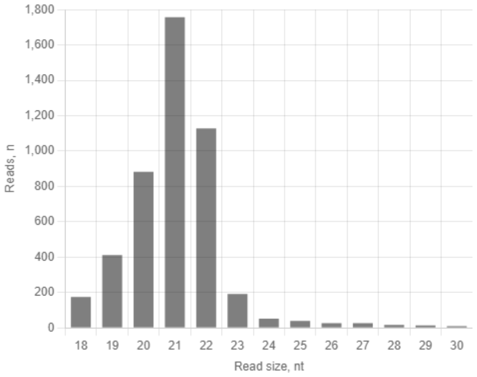

Length distribution of the reads

Dataset	Input reads	Virus	Reference	Mapped reads	Mapped reads, %	RPKM	Forward/Reverse balance	Nonredundant, %	Strain specific	Strain specific, % from total mapped reads	Strain specific, RPKM
3K_3	10000000	SMoV	SMoV_RNA1B	5082	0.051	72.1	1.3	47.1	3850	75.8	36.4

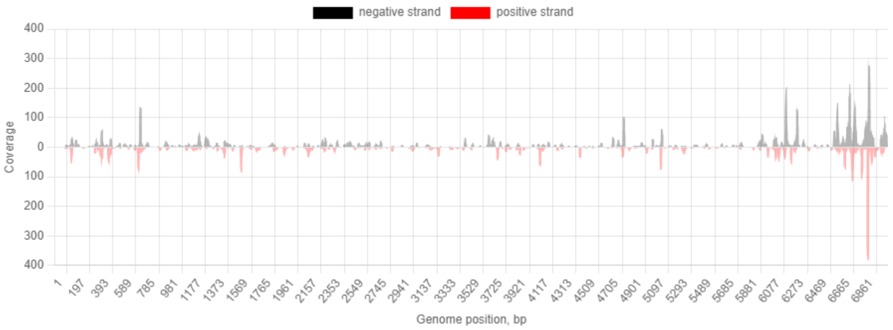

Mapping coverage of all reads matching SMoV_RNA1B (7511 reads)

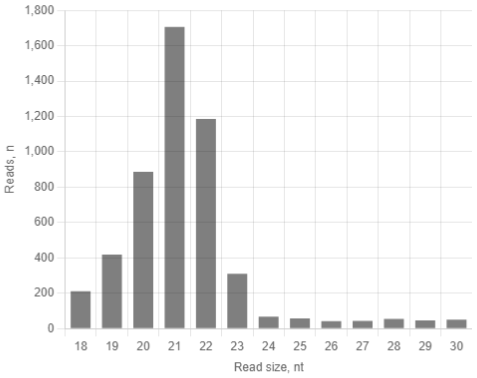

Length distribution of the reads

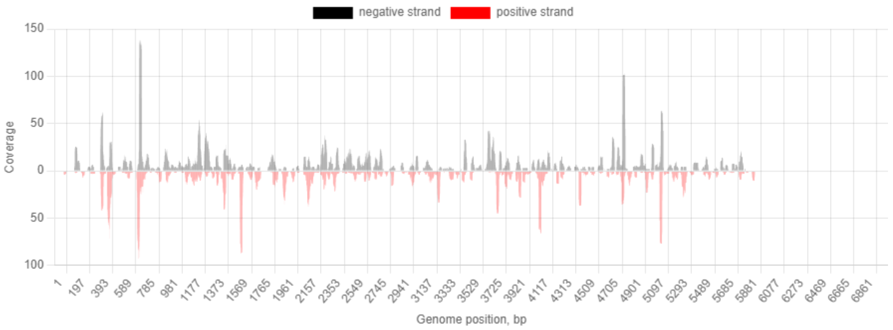

Mapping coverage of SMoV_RNA1B specific reads (3850 reads)

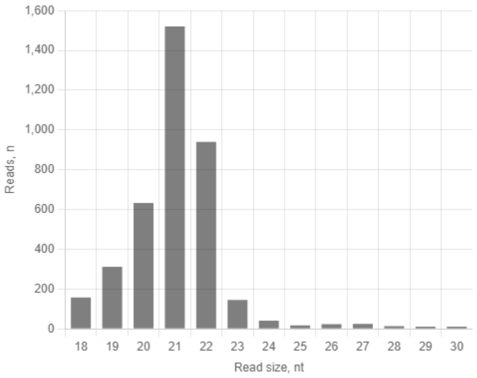

Length distribution of the reads

Dataset	Input reads	Virus	Reference	Mapped reads	Mapped reads, %	RPKM	Forward/Reverse balance	Nonredundant, %	Strain specific	Strain specific, % from total mapped reads	Strain specific, RPKM
3K_3	10000000	SMoV	SMoV_RNA1C	5308	0.053	75.4	1.3	48.4	4522	85.2	38.7

Dataset	Input reads	Virus	Reference	Mapped reads	Mapped reads, %	RPKM	Forward/Reverse balance	Nonredundant, %	Strain specific	Strain specific, % from total mapped reads	Strain specific, RPKM

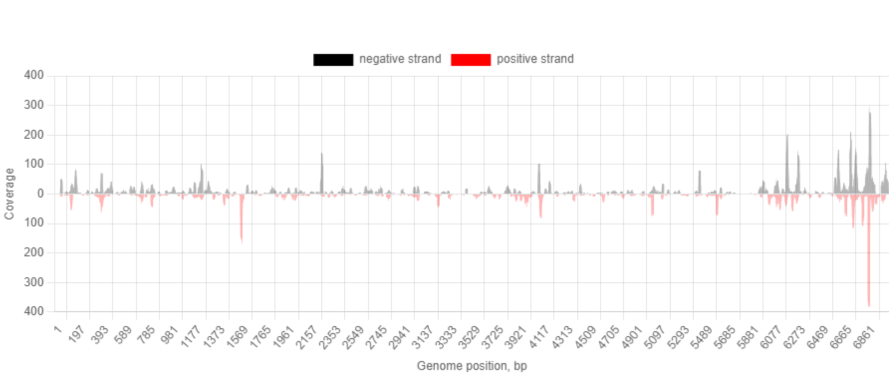

Mapping coverage of all reads matching SMoV_RNA1C (8226 reads)

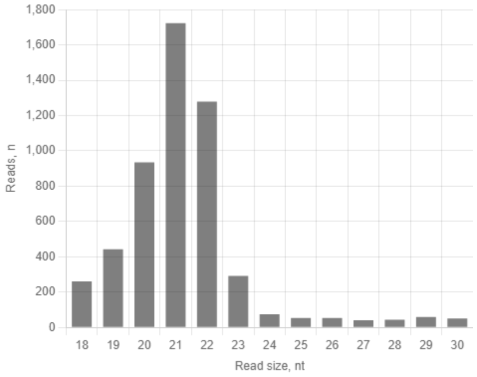

Length distribution of the reads

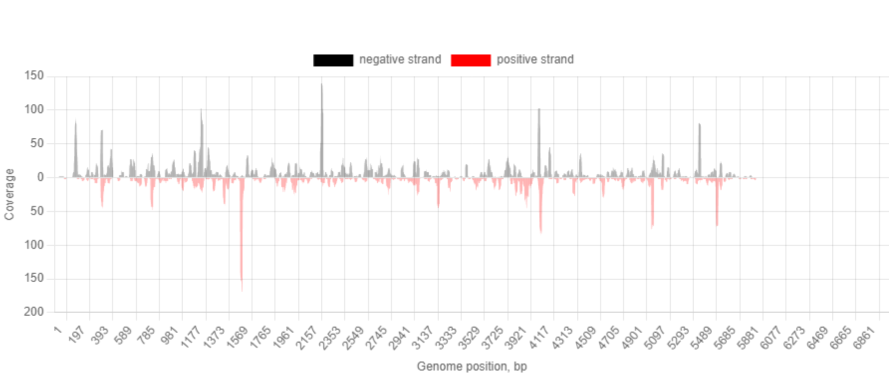

Mapping coverage of SMoV_RNA1C specific reads (4522 reads)

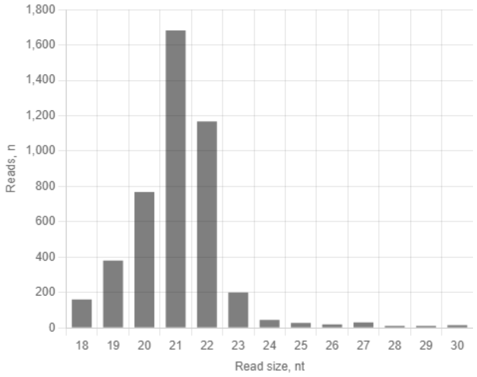

Length distribution of the reads

Dataset	Input reads	Virus	Reference	Mapped reads	Mapped reads, %	RPKM	Forward/Reverse balance	Nonredundant, %	Strain specific	Strain specific, % from total mapped reads	Strain specific, RPKM
3K_3	10000000	SMoV	SMoV_RNA2A	3527	0.035	56.2	1.6	52.3	3543	100.5	34.7

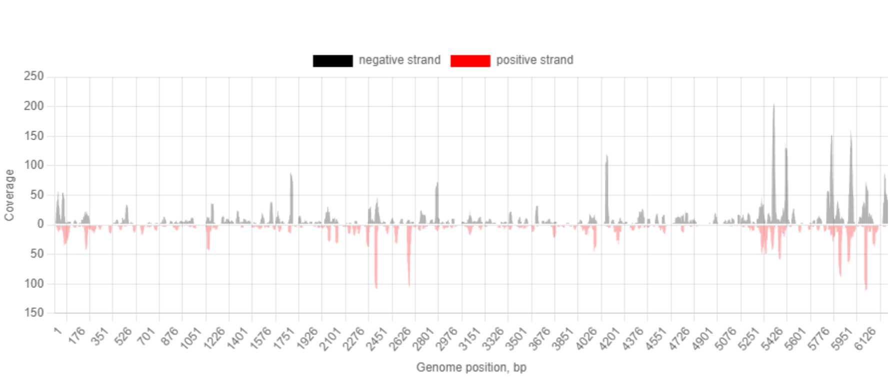

Mapping coverage of all reads matching SMoV_RNA2A (5328 reads)

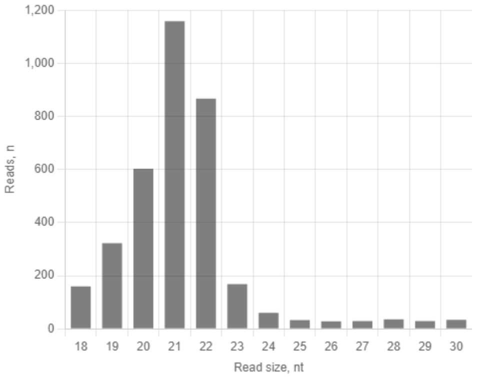

Length distribution of the reads

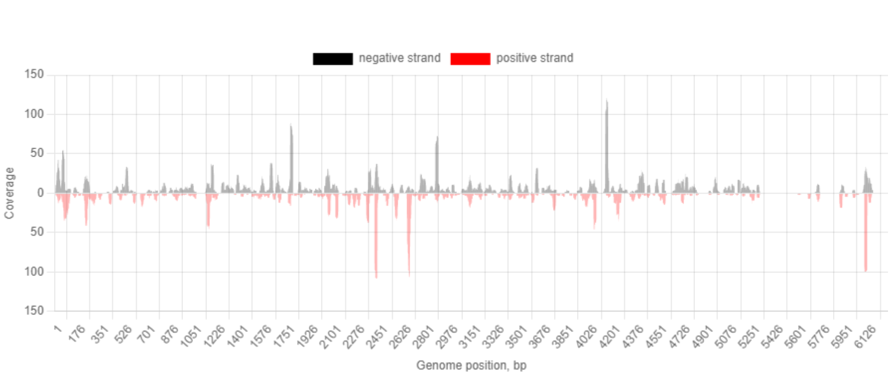

Mapping coverage of SMoV_RNA2A specific reads (3543 reads)

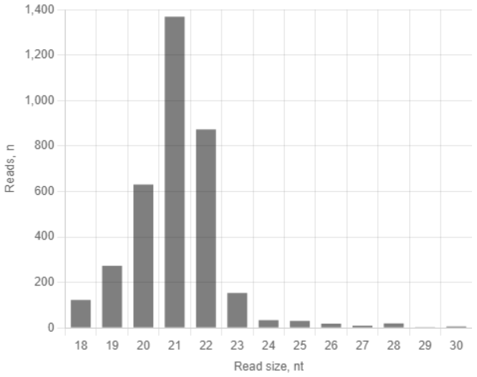

Length distribution of the reads

Dataset	Input reads	Virus	Reference	Mapped reads	Mapped reads, %	RPKM	Forward/Reverse balance	Nonredundant, %	Strain specific	Strain specific, % from total mapped reads	Strain specific, RPKM
3K_3	10000000	SMoV	SMoV_RNA2B	3053	0.031	48.3	1.6	52.5	2383	78.1	24.4

Dataset	Input reads	Virus	Reference	Mapped reads	Mapped reads, %	RPKM	Forward/Reverse balance	Nonredundant, %	Strain specific	Strain specific, % from total mapped reads	Strain specific, RPKM

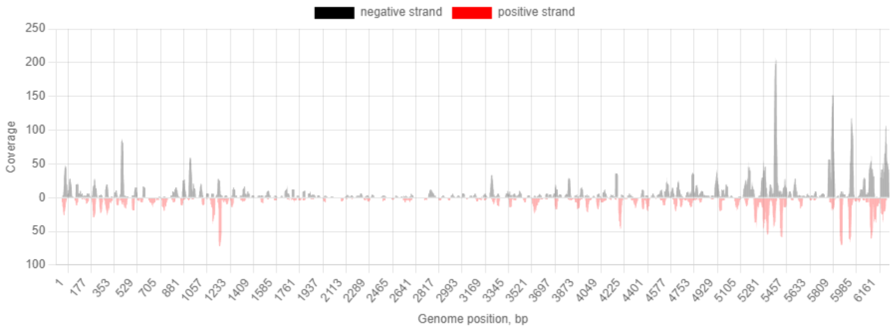

Mapping coverage of all reads matching SMoV_RNA2B (4406 reads)

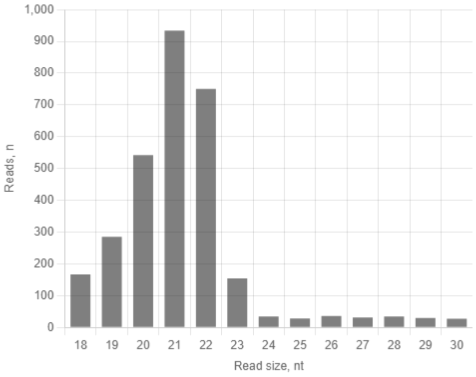

Length distribution of the reads

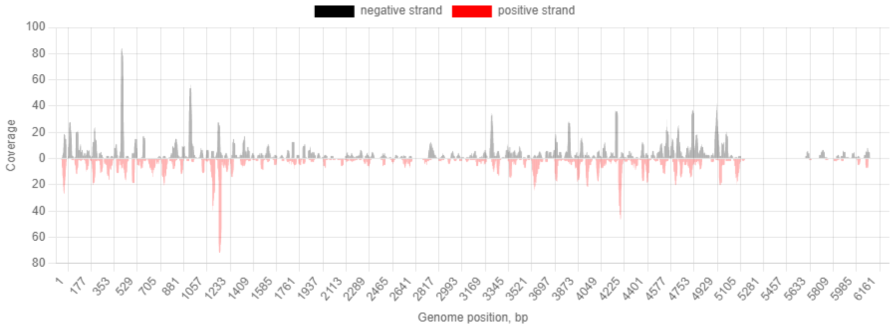

Mapping coverage of SMoV_RNA2B specific reads (2383 reads)

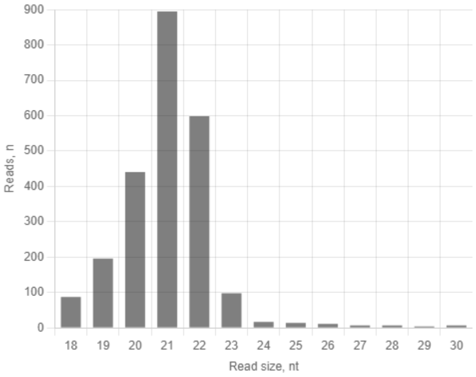

Length distribution of the reads

Dataset	Input reads	Virus	Reference	Mapped reads	Mapped reads, %	RPKM	Forward/Reverse balance	Nonredundant, %	Strain specific	Strain specific, % from total mapped reads	Strain specific, RPKM
3K_3	10000000	SMoV	SMoV_RNA2C	4212	0.042	66.4	1.5	47.3	2702	64.2	30.3

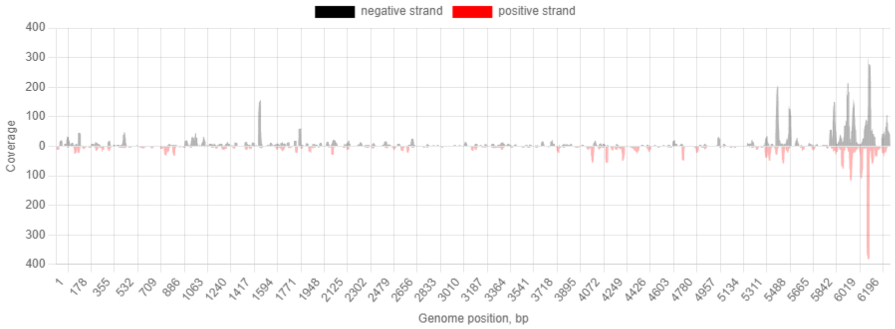

Mapping coverage of all reads matching SMoV_RNA2C (6056 reads)

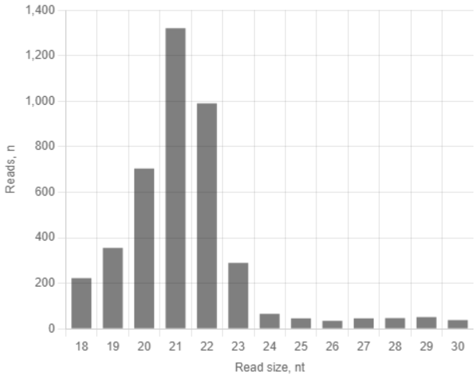

Length distribution of the reads

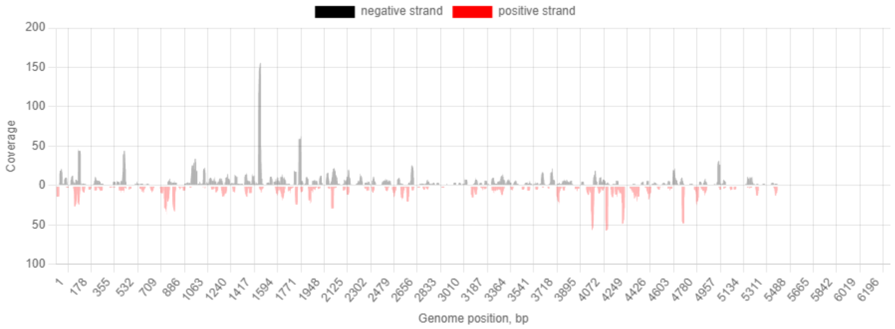

Mapping coverage of SMoV_RNA2C specific reads (2702 reads)

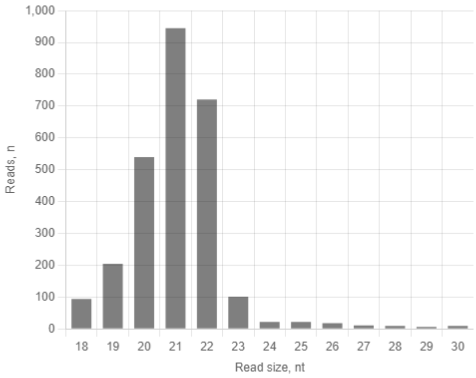

Length distribution of the reads

Dataset	Input reads	Virus	Reference	Mapped reads	Mapped reads, %	RPKM	Forward/Reverse balance	Nonredundant, %	Strain specific	Strain specific, % from total mapped reads	Strain specific, RPKM
3K_3	10000000	StrV1	StrV1_A	2437	0.024	17.2	1.0	61.3	2149	88.2	13.2

Dataset	Input reads	Virus	Reference	Mapped reads	Mapped reads, %	RPKM	Forward/Reverse balance	Nonredundant, %	Strain specific	Strain specific, % from total mapped reads	Strain specific, RPKM

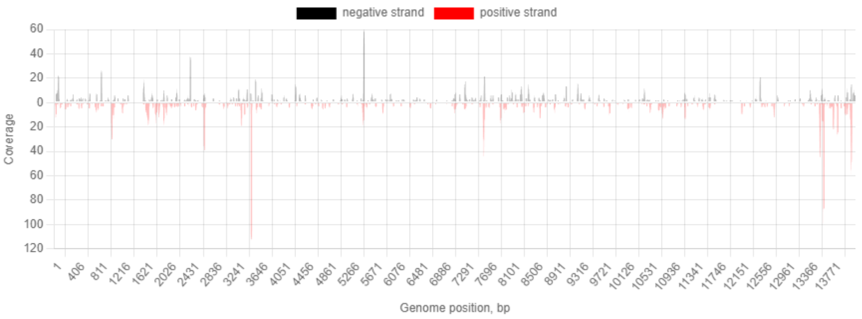

Mapping coverage of all reads matching StrV1_A (2859 reads)

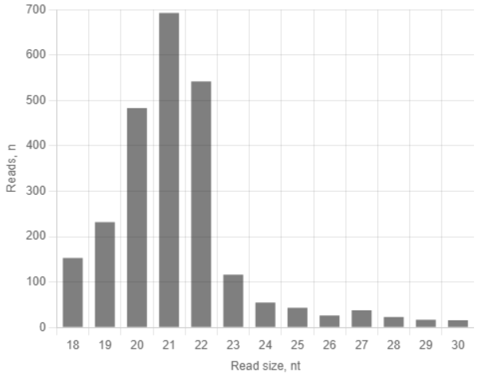

Length distribution of the reads

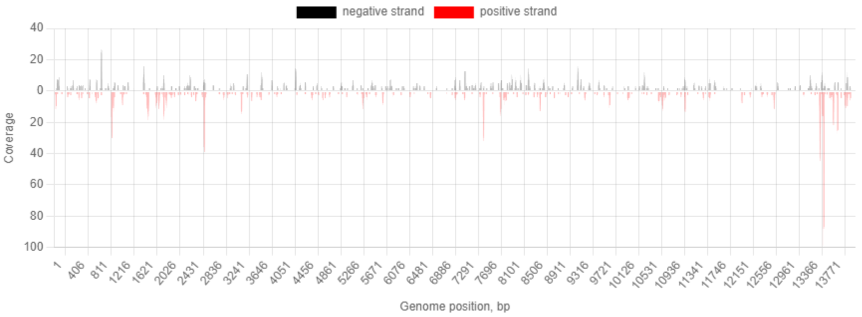

Mapping coverage of StrV1_A specific reads (2149 reads)

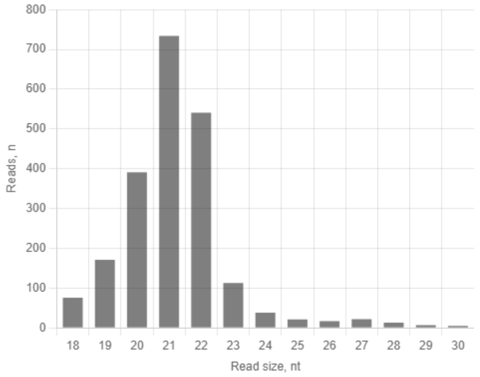

Length distribution of the reads

Dataset	Input reads	Virus	Reference	Mapped reads	Mapped reads, %	RPKM	Forward/Reverse balance	Nonredundant, %	Strain specific	Strain specific, % from total mapped reads	Strain specific, RPKM
3K_3	10000000	StrV1	StrV1_B	6422	0.064	45.8	1.6	40.3	6971	108.5	44.0

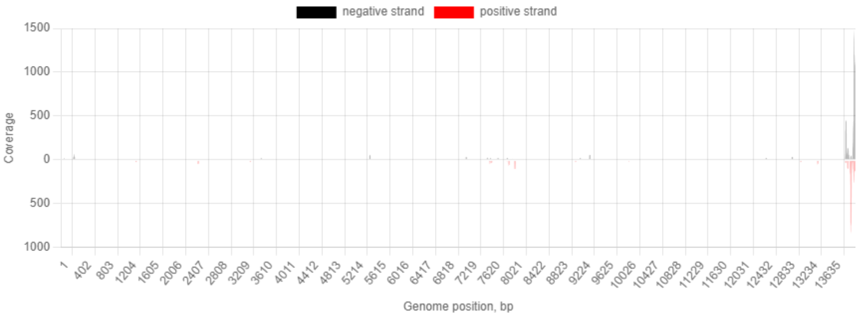

Mapping coverage of all reads matching StrV1_B (7267 reads)

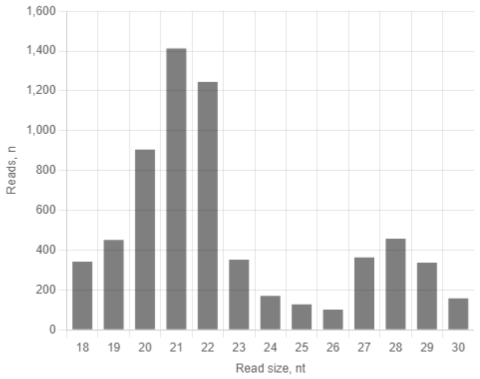

Length distribution of the reads

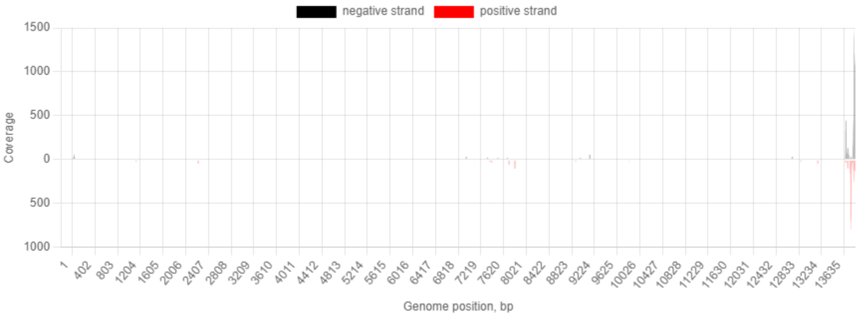

Mapping coverage of StrV1_B specific reads (6971 reads)

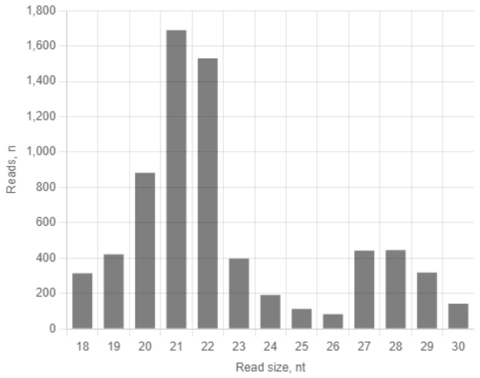

Length distribution of the reads

Dataset	Input reads	Virus	Reference	Mapped reads	Mapped reads, %	RPKM	Forward/Reverse balance	Nonredundant, %	Strain specific	Strain specific, % from total mapped reads	Strain specific, RPKM
3K_3	10000000	StrV1	StrV1_C	2004	0.020	14.1	1.1	61.6	1721	85.9	9.9

Dataset	Input reads	Virus	Reference	Mapped reads	Mapped reads, %	RPKM	Forward/Reverse balance	Nonredundant, %	Strain specific	Strain specific, % from total mapped reads	Strain specific, RPKM

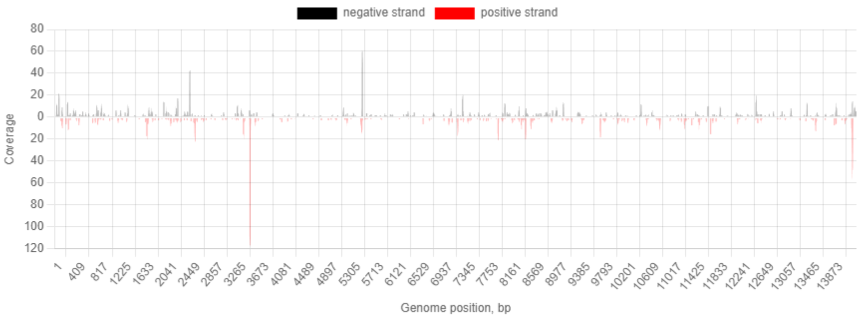

Mapping coverage of all reads matching StrV1_C (2365 reads)

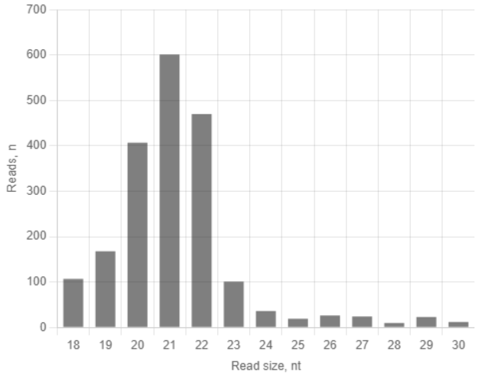

Length distribution of the reads

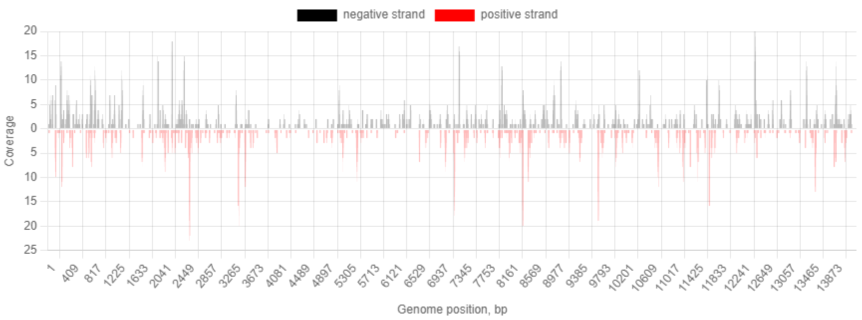

Mapping coverage of StrV1_C specific reads (1721 reads)

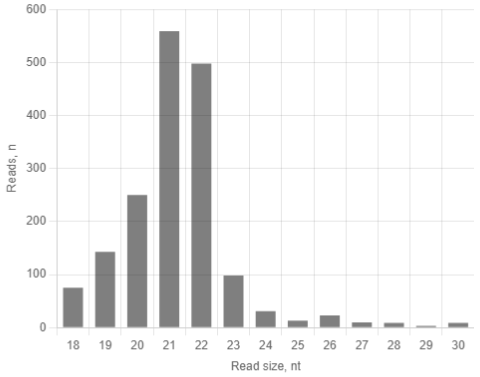

Length distribution of the reads

Mappings of the 3K_4 reads against the viral references.

Dataset	Input reads	Virus	Reference	Mapped reads	Mapped reads, %	RPKM	Forward/Reverse balance	Nonredundant, %	Strain specific	Strain specific, % from total mapped reads	Strain specific, RPKM
3K_4	10000000	SCV	SCV_A	16741	0.167	115.1	0.9	52.8	8443	50.4	100.2

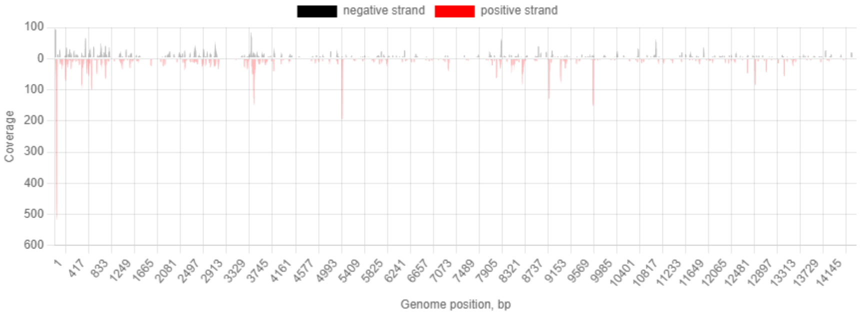

Mapping coverage of all reads matching SCV_A (9737 reads)

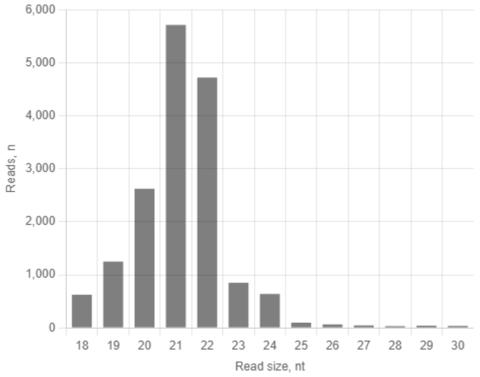

Length distribution of the reads

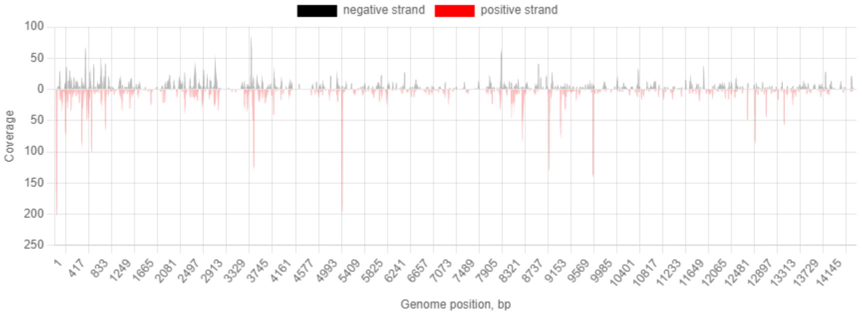

Mapping coverage of SCV_A specific reads (8443 reads)

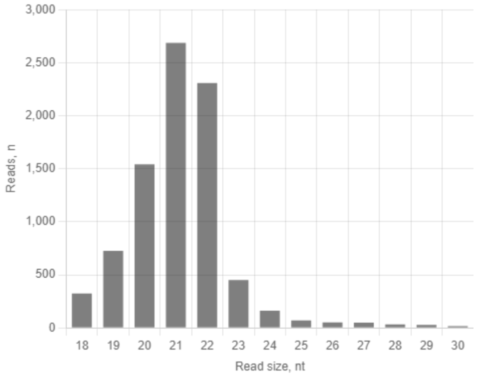

Length distribution of the reads

Dataset	Input reads	Virus	Reference	Mapped reads	Mapped reads, %	RPKM	Forward/Reverse balance	Nonredundant, %	Strain specific	Strain specific, % from total mapped reads	Strain specific, RPKM
3K_4	10000000	SCV	SCV_B	11607	0.116	79.7	0.8	56.4	5294	45.6	64.9

Dataset	Input reads	Virus	Reference	Mapped reads	Mapped reads, %	RPKM	Forward/Reverse balance	Nonredundant, %	Strain specific	Strain specific, % from total mapped reads	Strain specific, RPKM

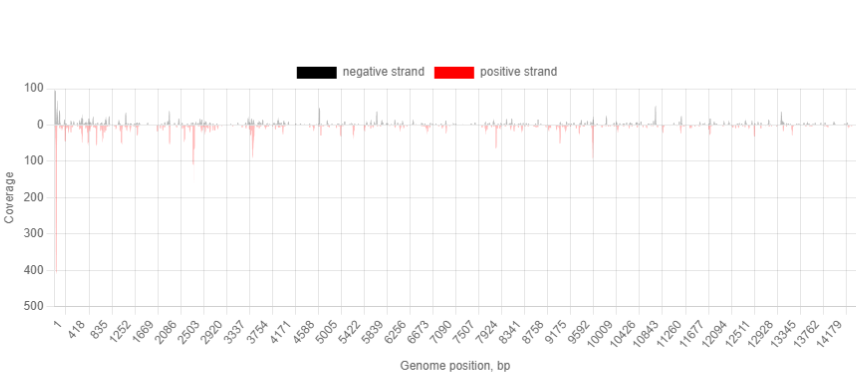

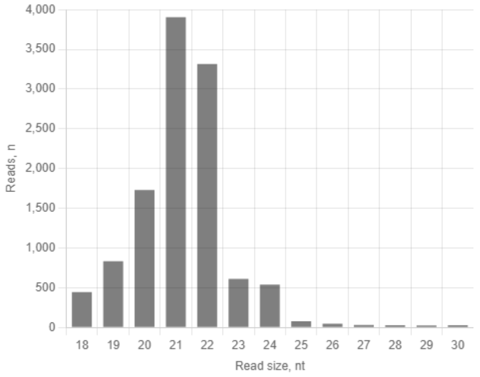

Mapping coverage of all reads matching SCV_B (6588 reads)

Length distribution of the reads

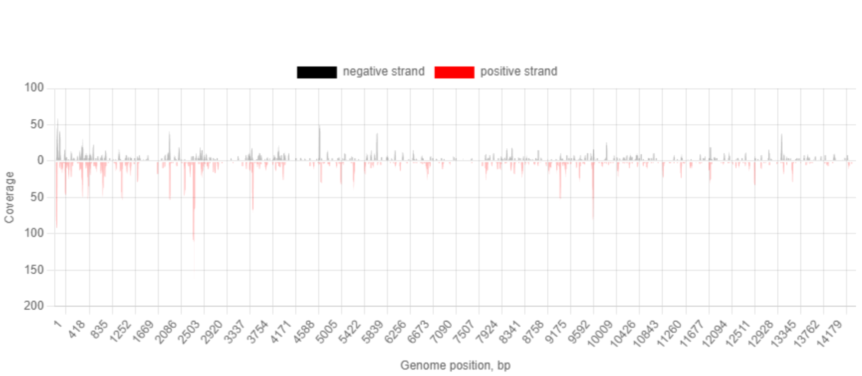

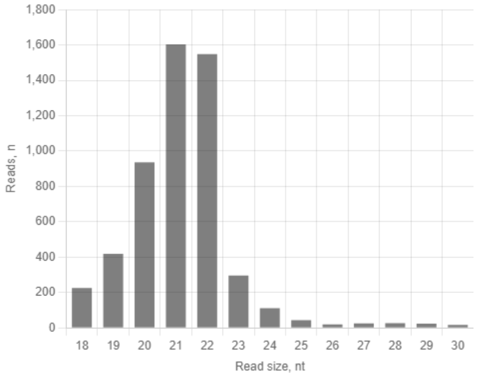

Mapping coverage of SCV_B specific reads (5294 reads)

Length distribution of the reads

Dataset	Input reads	Virus	Reference	Mapped reads	Mapped reads, %	RPKM	Forward/Reverse balance	Nonredundant, %	Strain specific	Strain specific, % from total mapped reads	Strain specific, RPKM
3K_4	10000000	SMoV	SMoV_RNA1A	9272	0.093	131.8	1.3	51.5	4731	51.0	72.5

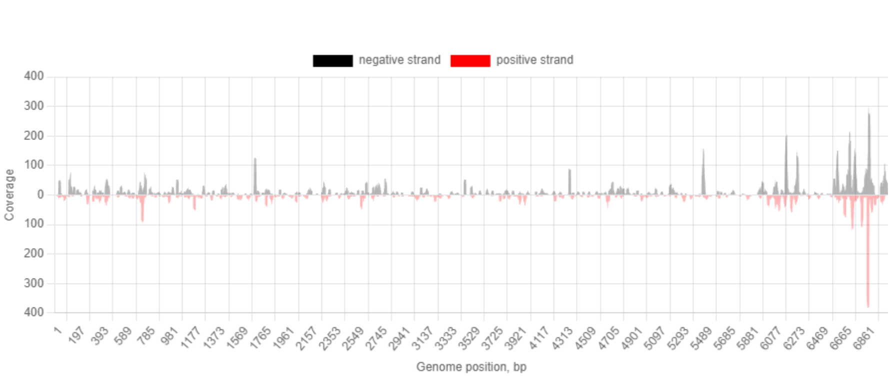

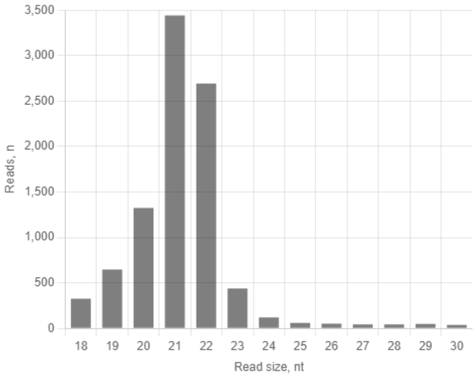

Mapping coverage of all reads matching SMoV_RNA1A (8455 reads)

Length distribution of the reads

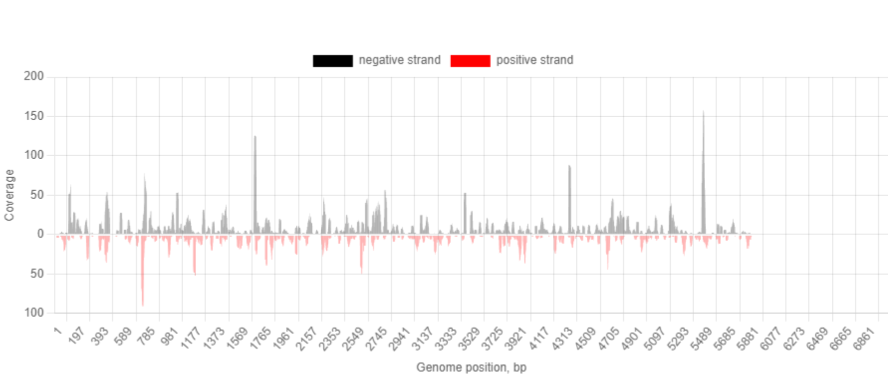

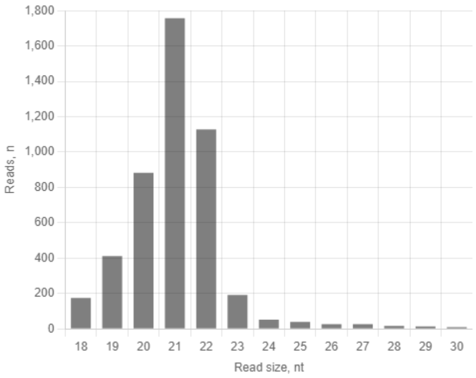

Mapping coverage of SMoV_RNA1A specific reads (4731 reads)

Length distribution of the reads

Dataset	Input reads	Virus	Reference	Mapped reads	Mapped reads, %	RPKM	Forward/Reverse balance	Nonredundant, %	Strain specific	Strain specific, % from total mapped reads	Strain specific, RPKM
3K_4	10000000	SMoV	SMoV_RNA1B	8175	0.082	115.9	1.2	50.3	3850	47.1	58.0

Dataset	Input reads	Virus	Reference	Mapped reads	Mapped reads, %	RPKM	Forward/Reverse balance	Nonredundant, %	Strain specific	Strain specific, % from total mapped reads	Strain specific, RPKM

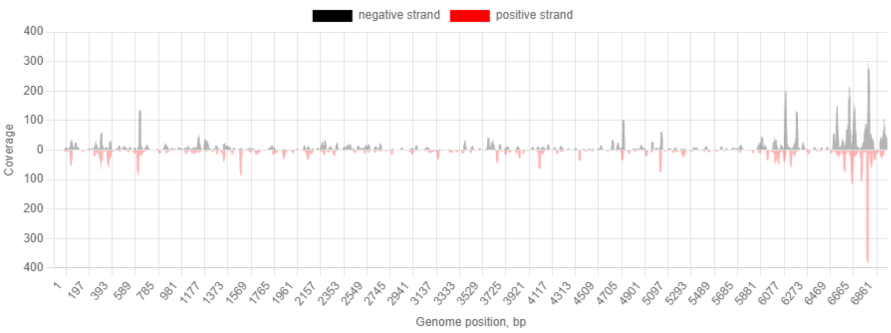

Mapping coverage of **all reads** matching SMoV_RNA1B (7511 reads)

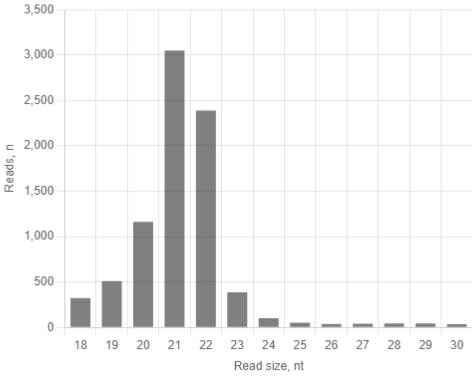

Length distribution of the reads

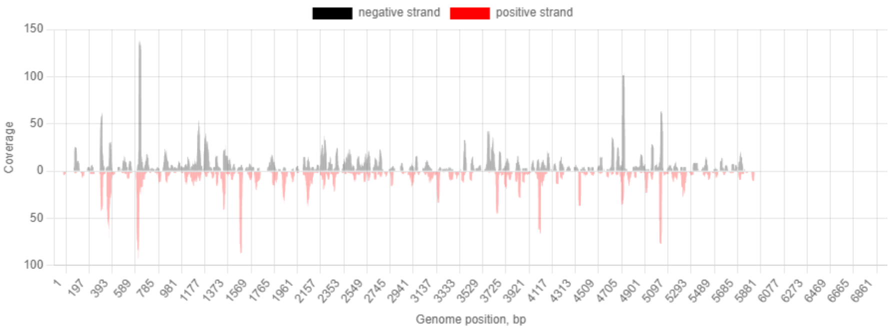

Mapping coverage of SMoV_RNA1B **specific reads** (3850 reads)

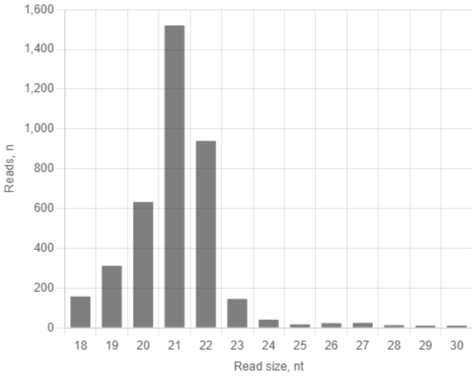

Length distribution of the reads

Dataset	Input reads	Virus	Reference	Mapped reads	Mapped reads, %	RPKM	Forward/Reverse balance	Nonredundant, %	Strain specific	Strain specific, % from total mapped reads	Strain specific, RPKM
3K_4	10000000	SMoV	SMoV_RNA1C	8838	0.088	125.5	1.2	51.1	4522	51.2	66.5

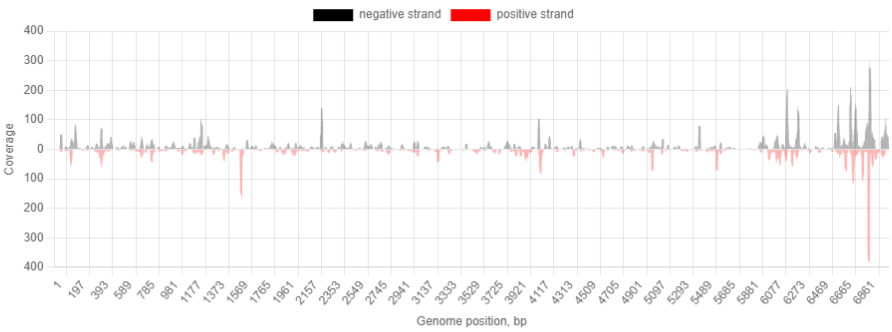

Mapping coverage of **all reads** matching SMoV_RNA1C (8226 reads)

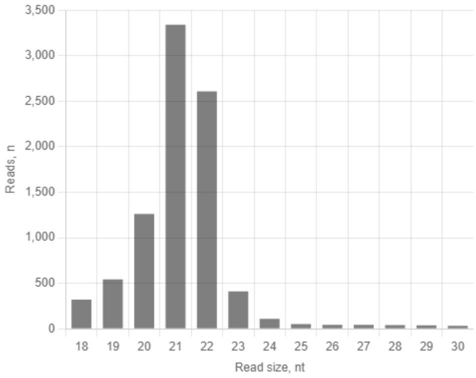

Length distribution of the reads

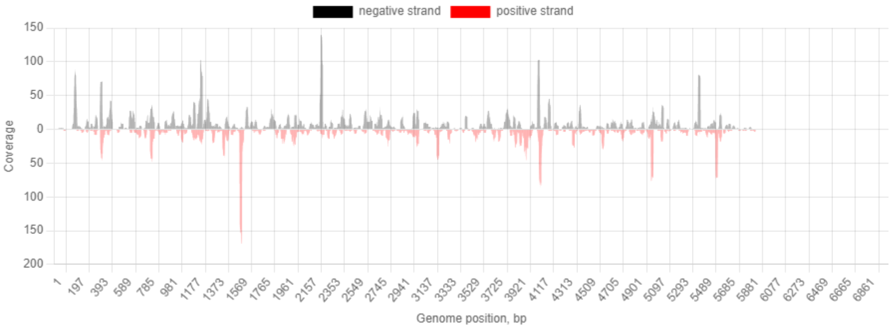

Mapping coverage of SMoV_RNA1C **specific reads** (4522 reads)

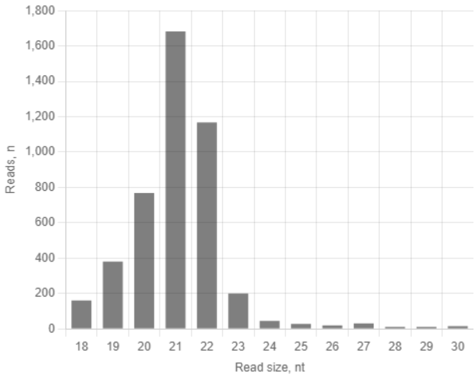

Length distribution of the reads

Dataset	Input reads	Virus	Reference	Mapped reads	Mapped reads, %	RPKM	Forward/Reverse balance	Nonredundant, %	Strain specific	Strain specific, % from total mapped reads	Strain specific, RPKM
3K_4	10000000	SMoV	SMoV_RNA2A	5487	0.055	87.6	1.4	56.9	3543	64.6	56.5

Dataset	Input reads	Virus	Reference	Mapped reads	Mapped reads, %	RPKM	Forward/Reverse balance	Nonredundant, %	Strain specific	Strain specific, % from total mapped reads	Strain specific, RPKM

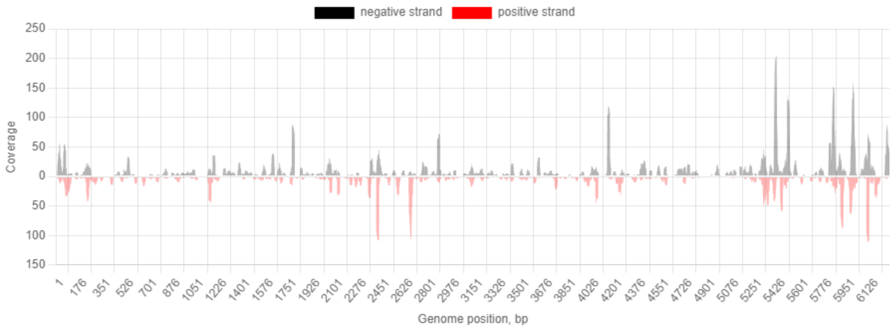

Mapping coverage of all reads matching SMoV_RNA2A (5328 reads)

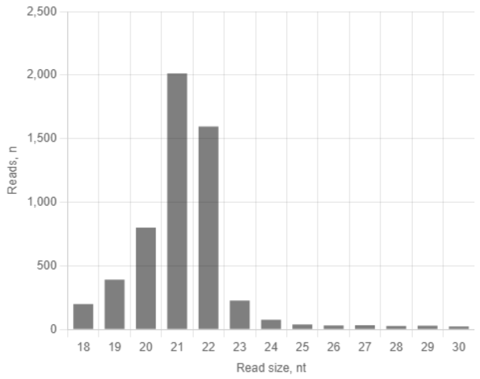

Length distribution of the reads

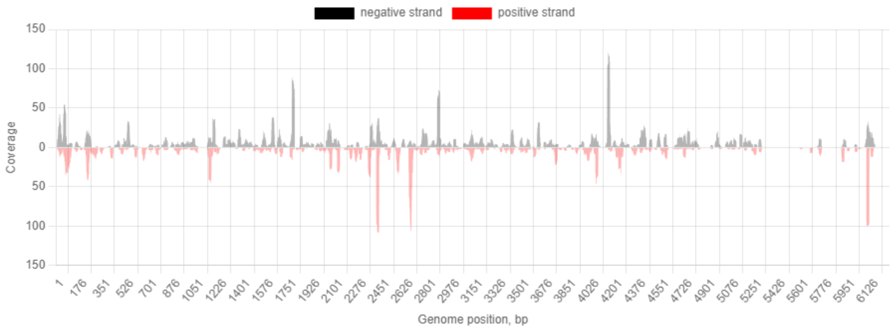

Mapping coverage of SMoV_RNA2A specific reads (3543 reads)

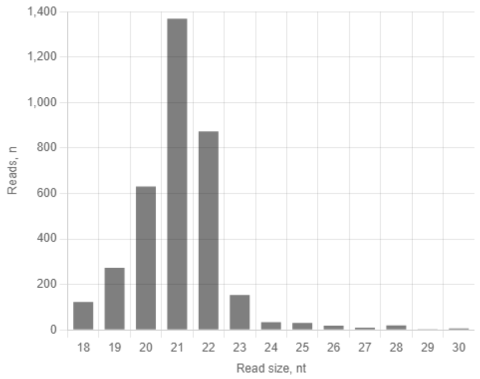

Length distribution of the reads

Dataset	Input reads	Virus	Reference	Mapped reads	Mapped reads, %	RPKM	Forward/Reverse balance	Nonredundant, %	Strain specific	Strain specific, % from total mapped reads	Strain specific, RPKM
3K_4	10000000	SMoV	SMoV_RNA2B	4859	0.049	76.8	1.4	59.8	2383	49.0	42.3

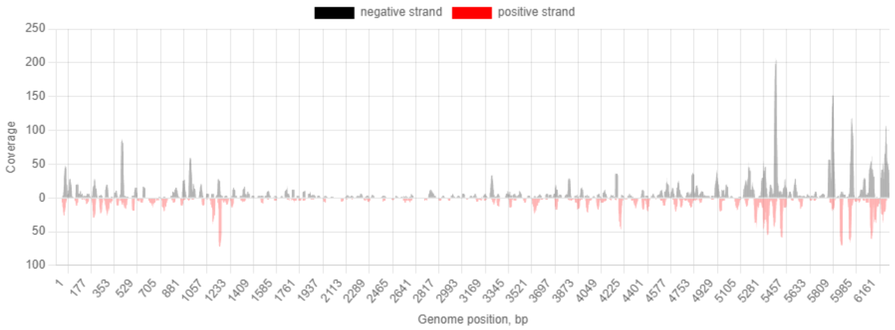

Mapping coverage of all reads matching SMoV_RNA2B (4406 reads)

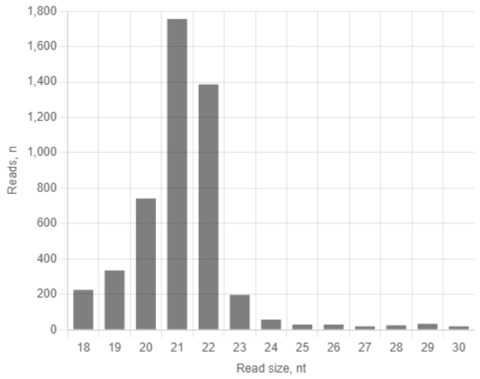

Length distribution of the reads

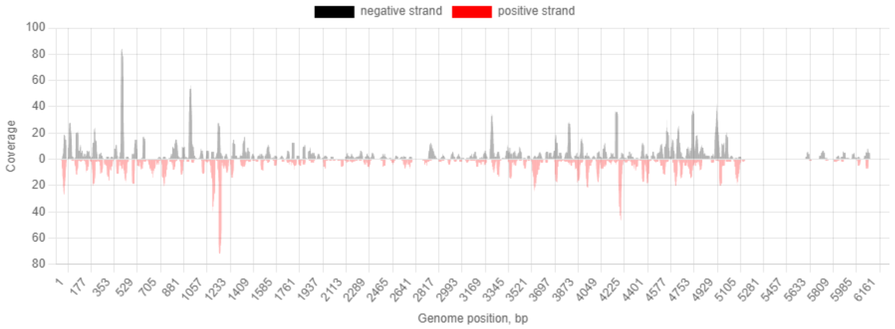

Mapping coverage of SMoV_RNA2B specific reads (2383 reads)

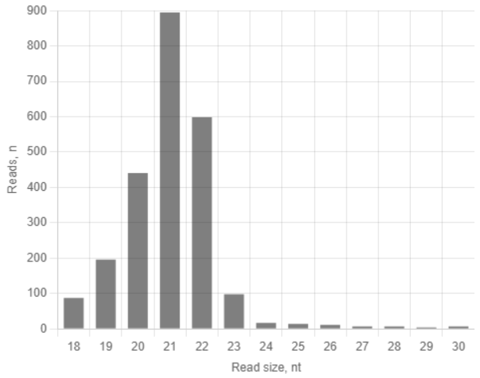

Length distribution of the reads

Dataset	Input reads	Virus	Reference	Mapped reads	Mapped reads, %	RPKM	Forward/Reverse balance	Nonredundant, %	Strain specific	Strain specific, % from total mapped reads	Strain specific, RPKM
3K_4	10000000	SMoV	SMoV_RNA2C	6716	0.067	105.9	1.2	49.5	2702	40.2	46.8

Dataset	Input reads	Virus	Reference	Mapped reads	Mapped reads, %	RPKM	Forward/Reverse balance	Nonredundant, %	Strain specific	Strain specific, % from total mapped reads	Strain specific, RPKM

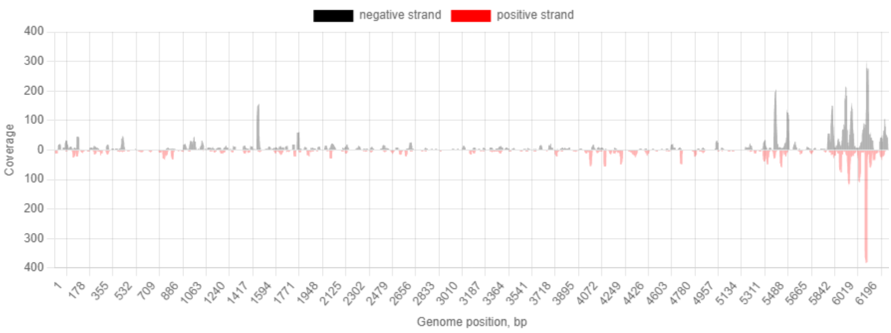

Mapping coverage of all reads matching SMoV_RNA2C (6056 reads)

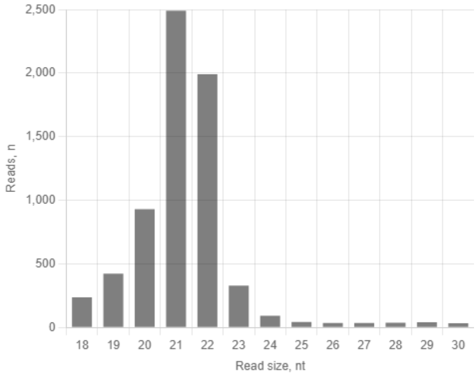

Length distribution of the reads

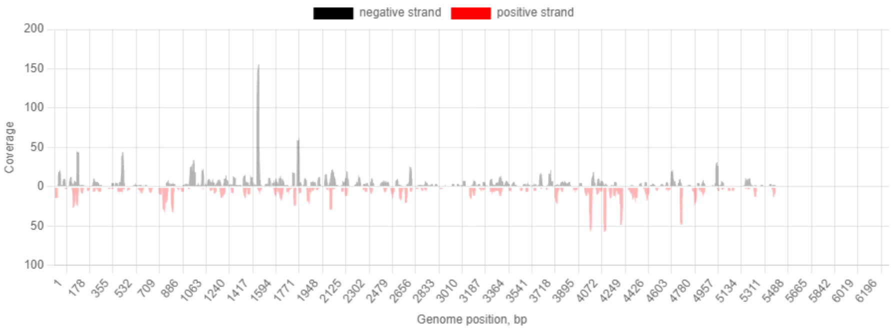

Mapping coverage of SMoV_RNA2C specific reads (2702 reads)

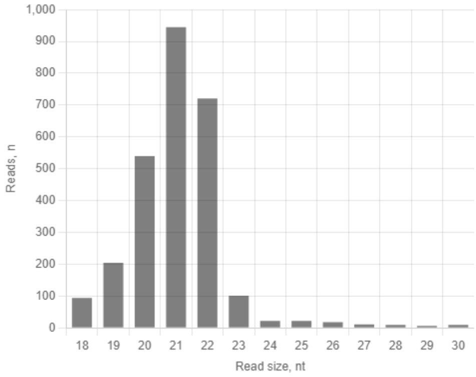

Length distribution of the reads

Dataset	Input reads	Virus	Reference	Mapped reads	Mapped reads, %	RPKM	Forward/Reverse balance	Nonredundant, %	Strain specific	Strain specific, % from total mapped reads	Strain specific, RPKM
3K_4	10000000	StrV1	StrV1_A	3980	0.040	28.1	1.0	71.8	2149	54.0	22.9

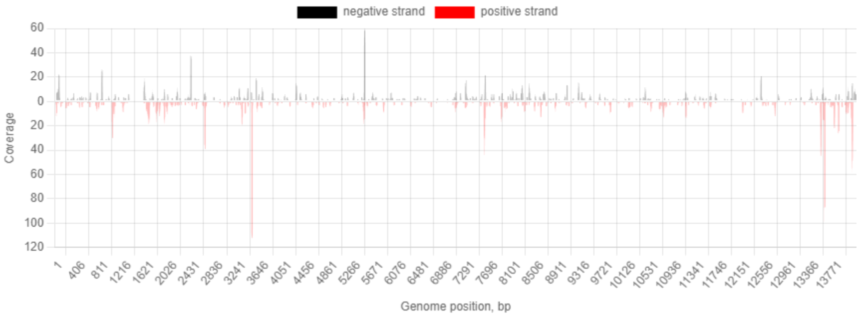

Mapping coverage of all reads matching StrV1_A (2859 reads)

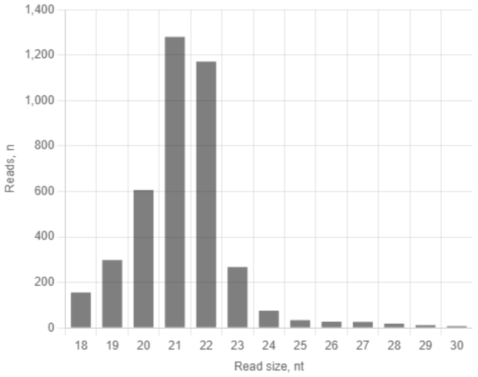

Length distribution of the reads

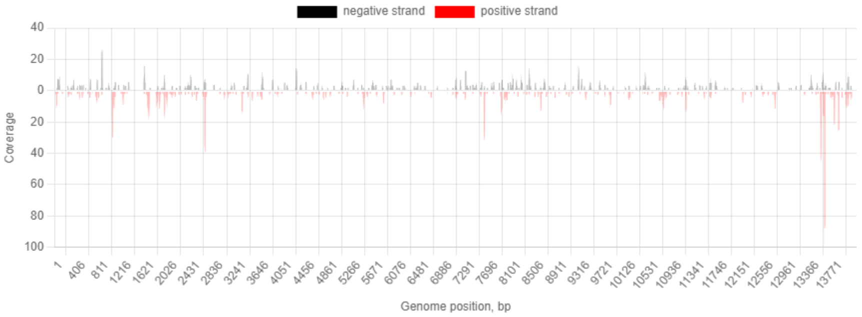

Mapping coverage of StrV1_A specific reads (2149 reads)

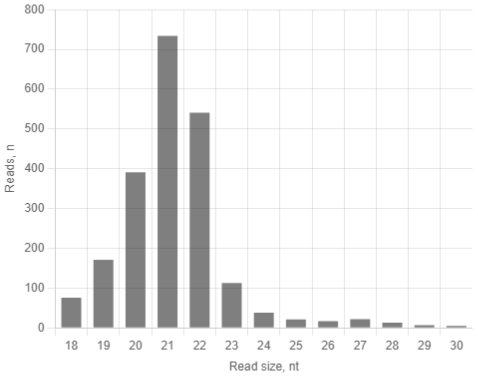

Length distribution of the reads

Dataset	Input reads	Virus	Reference	Mapped reads	Mapped reads, %	RPKM	Forward/Reverse balance	Nonredundant, %	Strain specific	Strain specific, % from total mapped reads	Strain specific, RPKM
3K_4	10000000	StrV1	StrV1_B	8719	0.087	62.1	1.4	42.7	6971	80.0	59.8

Dataset	Input reads	Virus	Reference	Mapped reads	Mapped reads, %	RPKM	Forward/Reverse balance	Nonredundant, %	Strain specific	Strain specific, % from total mapped reads	Strain specific, RPKM

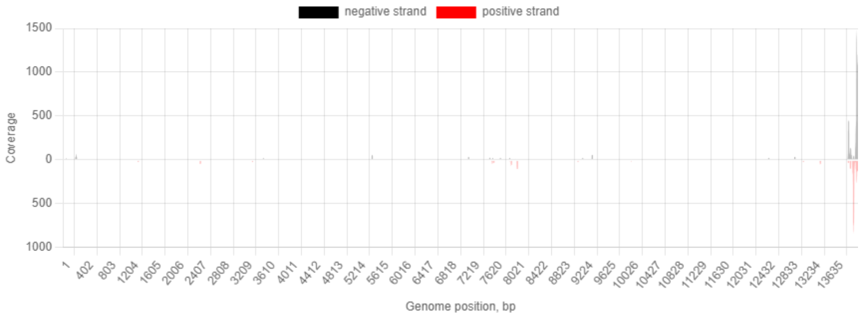

Mapping coverage of all reads matching StrV1_B (7267 reads)

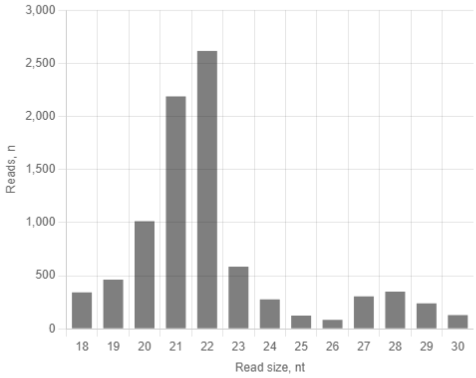

Length distribution of the reads

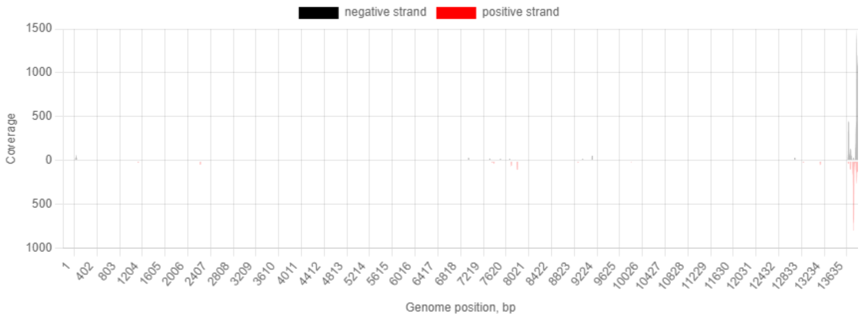

Mapping coverage of StrV1_B specific reads (6971 reads)

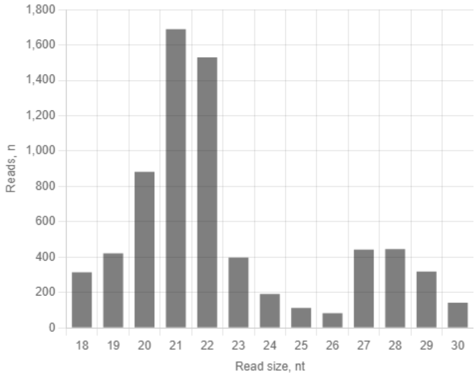

Length distribution of the reads

Dataset	Input reads	Virus	Reference	Mapped reads	Mapped reads, %	RPKM	Forward/Reverse balance	Nonredundant, %	Strain specific	Strain specific, % from total mapped reads	Strain specific, RPKM
3K_4	10000000	StrV1	StrV1_C	2729	0.027	19.1	1.1	75.2	1721	63.1	14.4

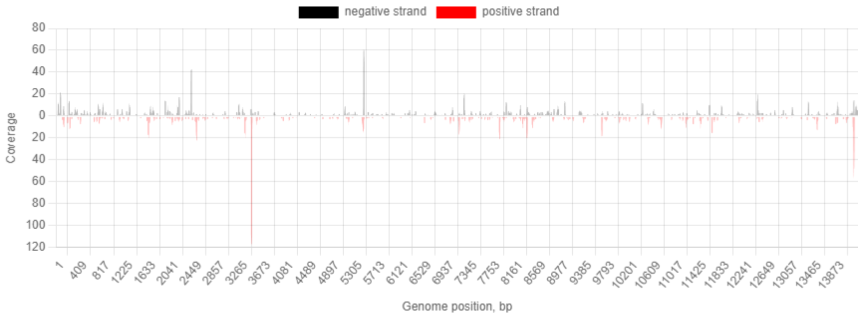

Mapping coverage of all reads matching StrV1_C (2365 reads)

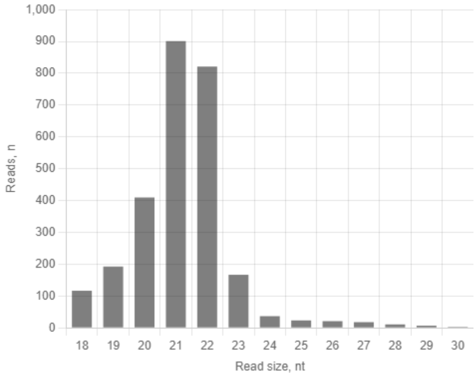

Length distribution of the reads

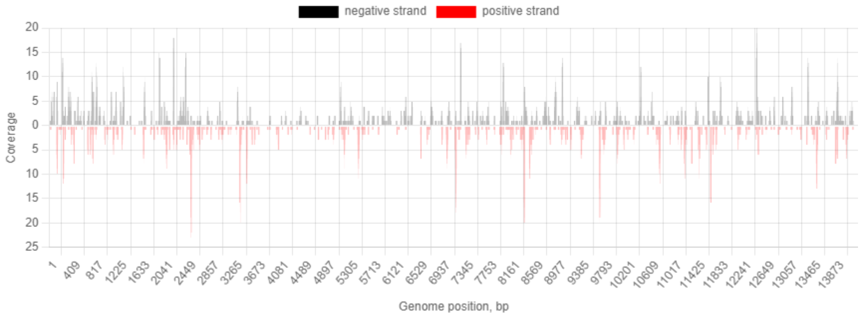

Mapping coverage of StrV1_C specific reads (1721 reads)

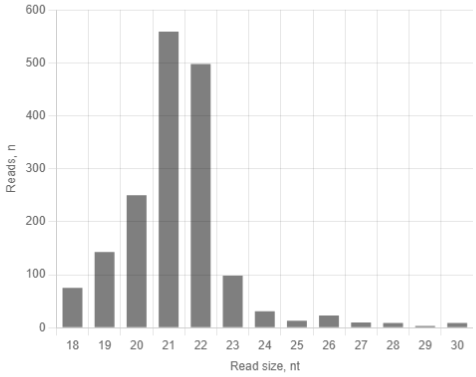

Length distribution of the reads
