# Supplementary material for: Analysis of Virus-Derived siRNAs in Strawberry Plants Co-Infected with Multiple Viruses and Their Genotypes
Source: Plants (Basel). 2023 Jul 6;12(13):2564. doi: 10.3390/plants12132564 (PMC10346599; doi:10.3390/plants12132564)
Supplement: Supplementary file 1 [file plants-12-02564-s001.zip › Figure S2.pdf]

Figure S2 Distribution of viral small interfering RNAs (reads) from the Fv-SMoV_BC_AC samples.

Plant Fv-SMoV_BC_AC-1 - datasets S1, S2

Plant Fv-SMoV_BC_AC-2 - datasets S3, S4, S92

Color legends show strand specificity of the mapped reads. Axes of abscissas show genomic position in nucleotides, axes of ordinary – sequence (mapping) coverage.

Mappings of the S1_L001 reads against the viral references. *qPCR detection: SMoV RNA1B,C & SMoV RNA2A,C*

Dataset	Input reads	Virus	Reference	Mapped reads	Mapped reads, %	RPKM	Forward/Reverse balance	Nonredundant, %	Strain specific	Strain specific, % from total mapped reads	Strain specific, RPKM
S1_L001	10000000	SMoV	SMoV_RNA1A	2553	0.026	36.3	1.6	42.3	5	0.2	0.1

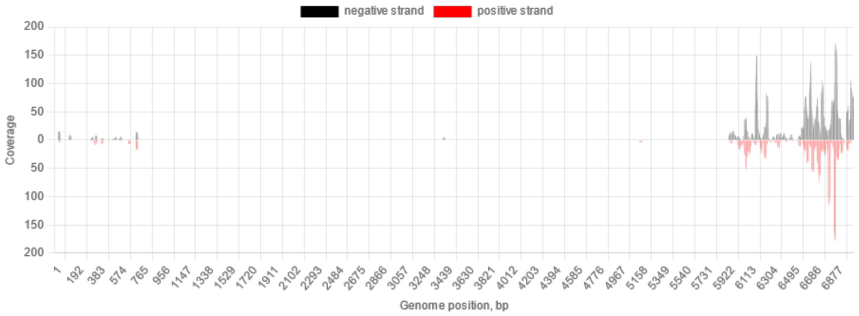

Mapping coverage of all reads matching SMoV_RNA1A (2553 reads)

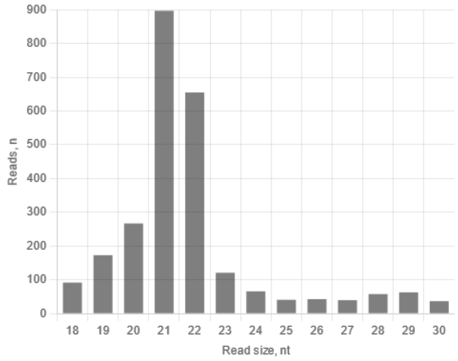

Length distribution of the reads

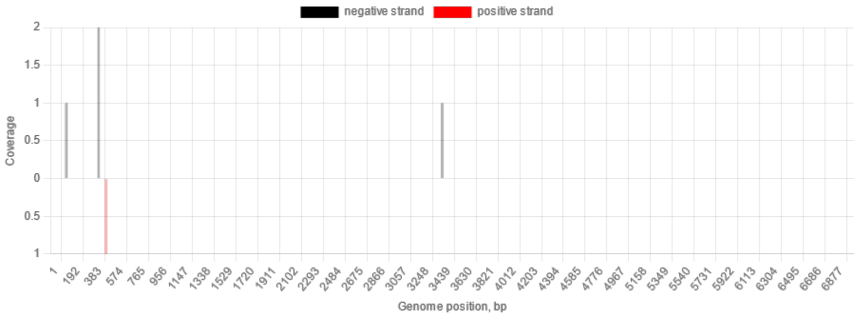

Mapping coverage of SMoV_RNA1A specific reads (5 reads)

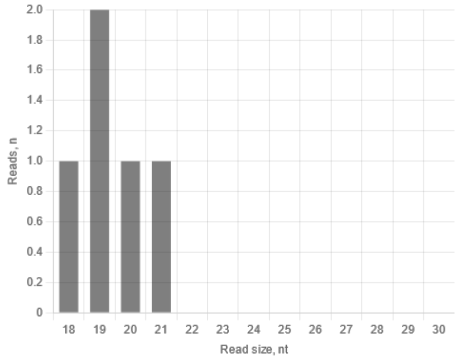

Length distribution of the reads

Dataset	Input reads	Virus	Reference	Mapped reads	Mapped reads, %	RPKM	Forward/Reverse balance	Nonredundant, %	Strain specific	Strain specific, % from total mapped reads	Strain specific, RPKM
S1_L001	10000000	SMoV	SMoV_RNA1B	4259	0.043	60.4	1.6	52.3	1654	38.8	23.5

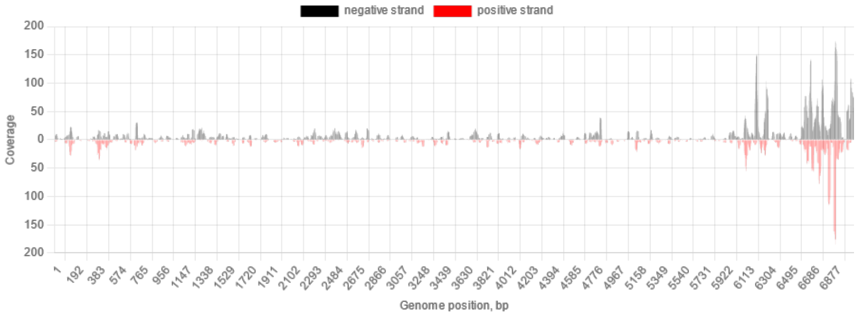

Mapping coverage of all reads matching SMoV_RNA1B (4259 reads)

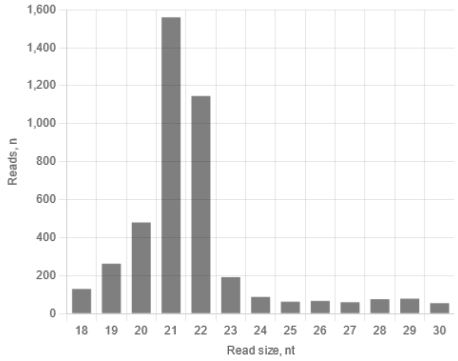

Length distribution of the reads

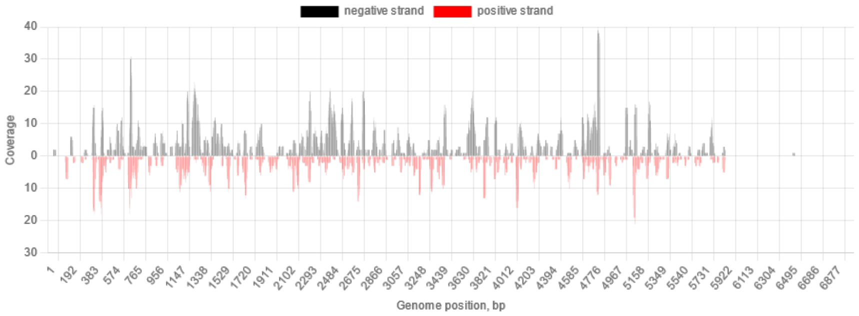

Mapping coverage of SMoV_RNA1B specific reads (1654 reads)

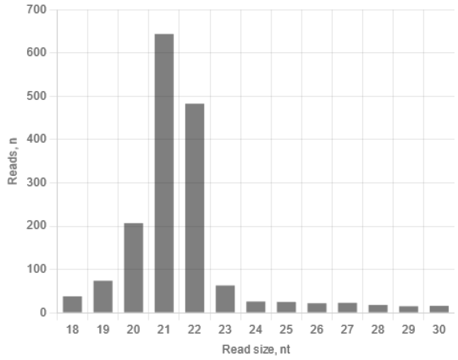

Length distribution of the reads

Dataset	Input reads	Virus	Reference	Mapped reads	Mapped reads, %	RPKM	Forward/Reverse balance	Nonredundant, %	Strain specific	Strain specific, % from total mapped reads	Strain specific, RPKM
S1_L001	10000000	SMoV	SMoV_RNA1C	6533	0.065	92.8	1.4	54.3	3924	60.1	55.7

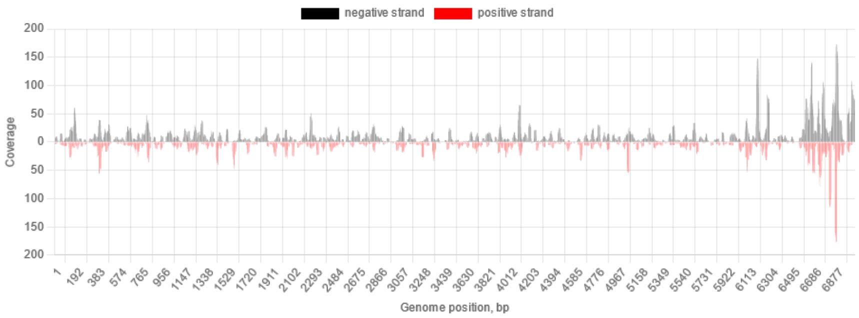

Mapping coverage of all reads matching SMoV_RNA1C (6533 reads)

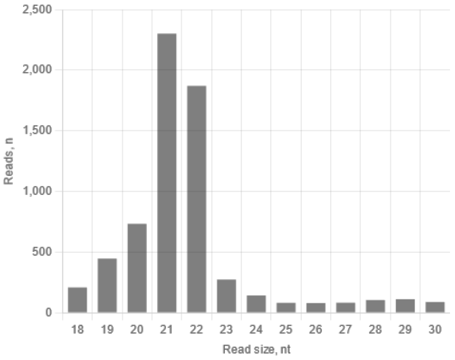

Length distribution of the reads

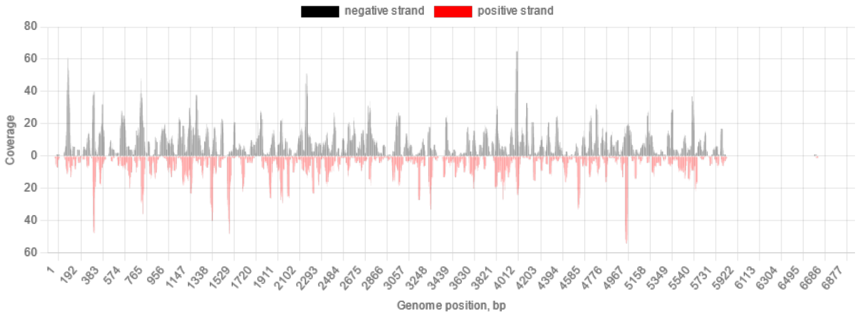

Mapping coverage of SMoV_RNA1C specific reads (3924 reads)

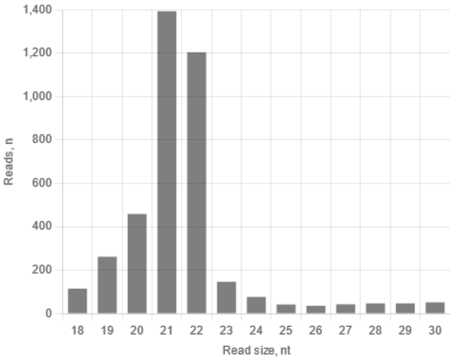

Length distribution of the reads

Dataset	Input reads	Virus	Reference	Mapped reads	Mapped reads, %	RPKM	Forward/Reverse balance	Nonredundant, %	Strain specific	Strain specific, % from total mapped reads	Strain specific, RPKM
S1_L001	10000000	SMoV	SMoV_RNA2A	4707	0.047	75.1	1.7	58.6	3373	71.7	53.8

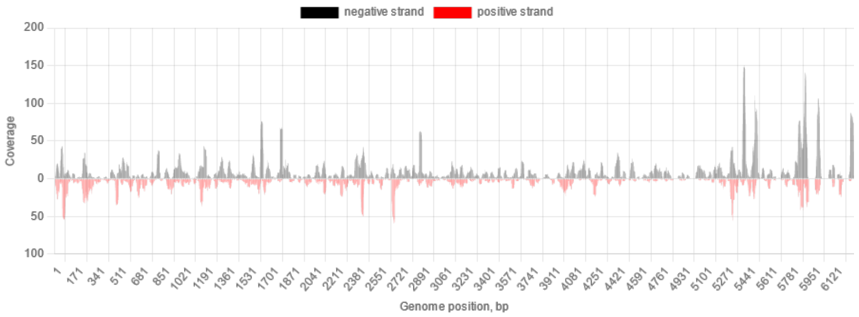

Mapping coverage of all reads matching SMoV_RNA2A (4707 reads)

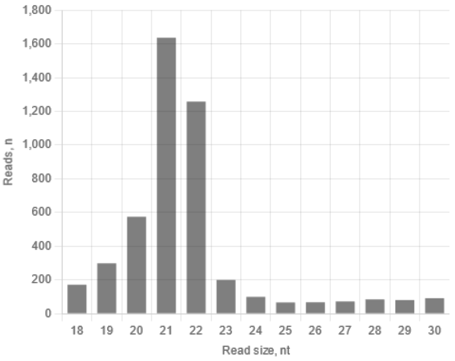

Length distribution of the reads

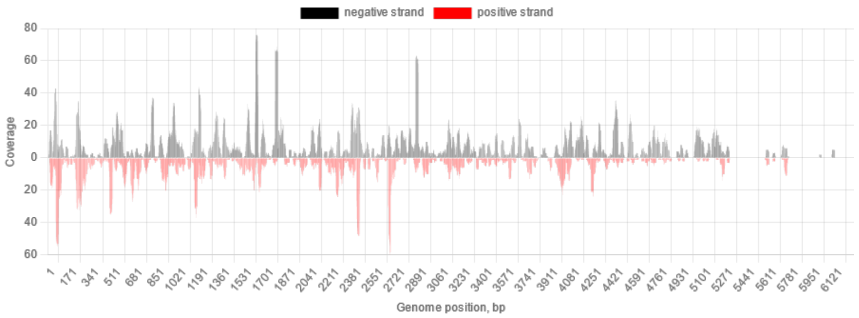

Mapping coverage of SMoV_RNA2A specific reads (3373 reads)

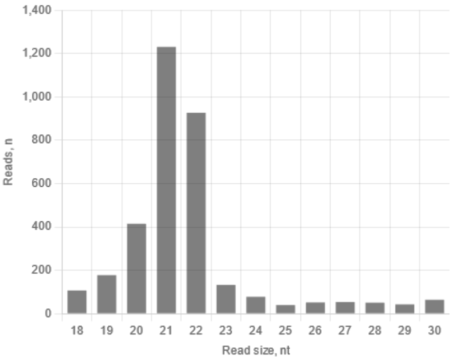

Length distribution of the reads

Dataset	Input reads	Virus	Reference	Mapped reads	Mapped reads, %	RPKM	Forward/Reverse balance	Nonredundant, %	Strain specific	Strain specific, % from total mapped reads	Strain specific, RPKM
S1_L001	10000000	SMoV	SMoV_RNA2B	1338	0.013	21.2	2.1	51.9	22	1.6	0.3

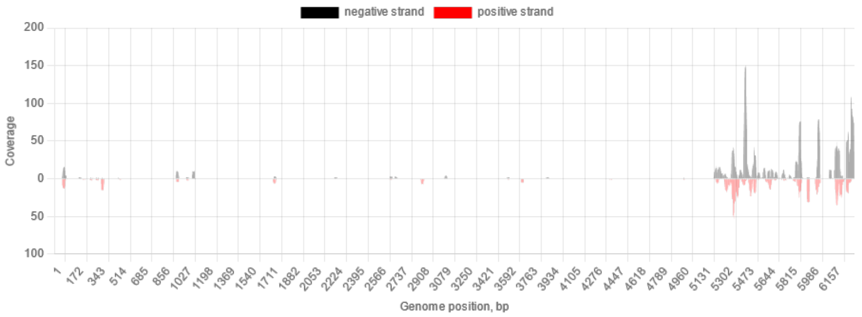

Mapping coverage of all reads matching SMoV_RNA2B (1338 reads)

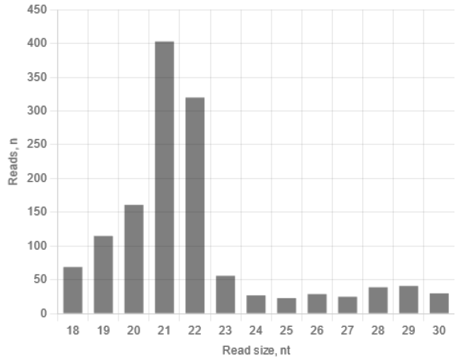

Length distribution of the reads

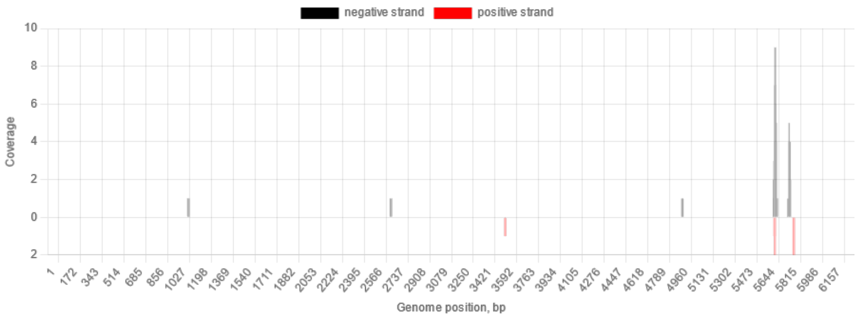

Mapping coverage of SMoV_RNA2B specific reads (22 reads)

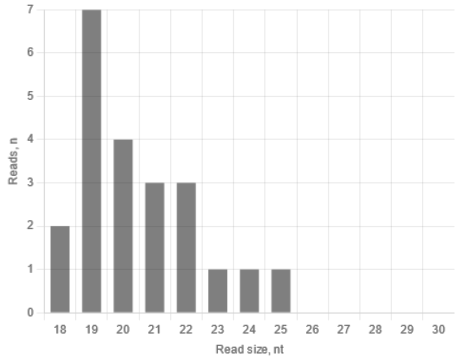

Length distribution of the reads

Dataset	Input reads	Virus	Reference	Mapped reads	Mapped reads, %	RPKM	Forward/Reverse balance	Nonredundant, %	Strain specific	Strain specific, % from total mapped reads	Strain specific, RPKM
S1_L001	10000000	SMoV	SMoV_RNA2C	5494	0.055	86.7	1.5	51.9	3052	55.6	48.2

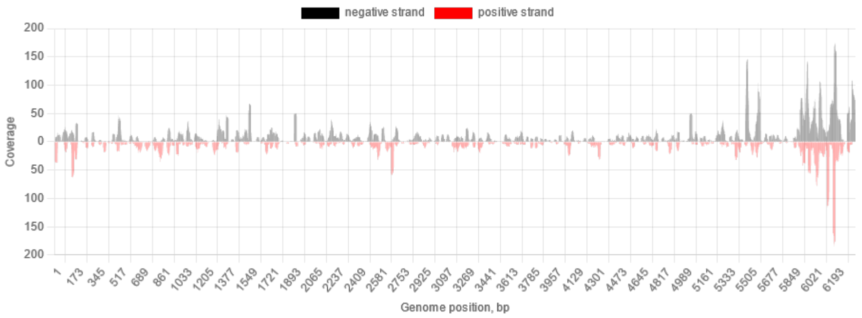

Mapping coverage of all reads matching SMoV_RNA2C (5494 reads)

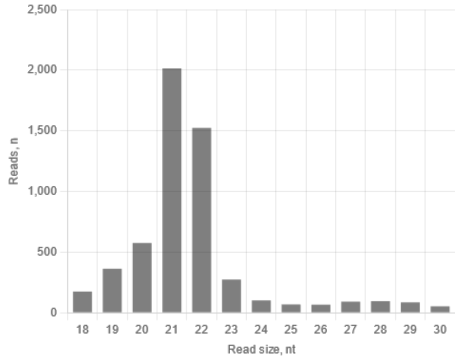

Length distribution of the reads

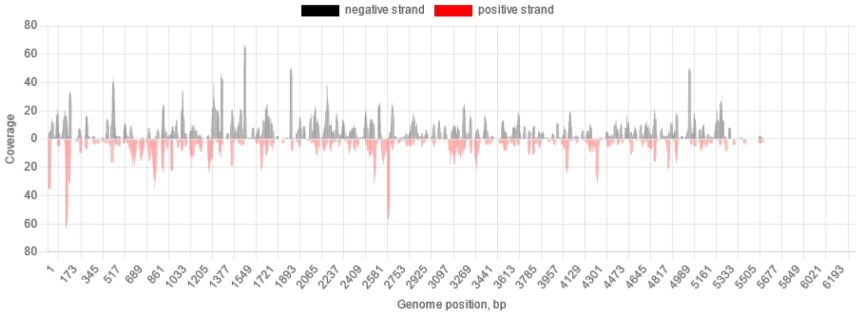

Mapping coverage of SMoV_RNA2C specific reads (3052 reads)

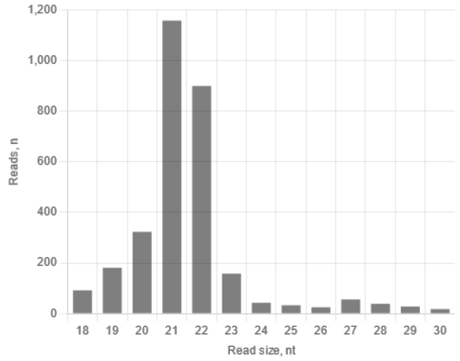

Length distribution of the reads

Mappings of the S2_L001 reads against the viral references. *qPCR detection: SMoV RNA1B,C & SMoV RNA2A,C*

Dataset	Input reads	Virus	Reference	Mapped reads	Mapped reads, %	RPKM	Forward/Reverse balance	Nonredundant, %	Strain specific	Strain specific, % from total mapped reads	Strain specific, RPKM
S2_L001	10000000	SMoV	SMoV_RNA1A	1629	0.016	23.2	1.8	44.6	1654	101.5	0.0

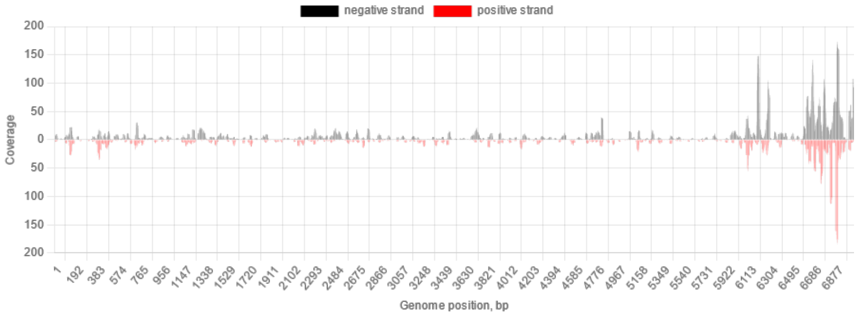

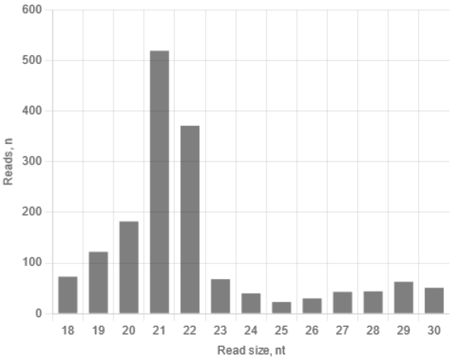

Mapping coverage of all reads matching SMoV_RNA1A (4259 reads)

Length distribution of the reads

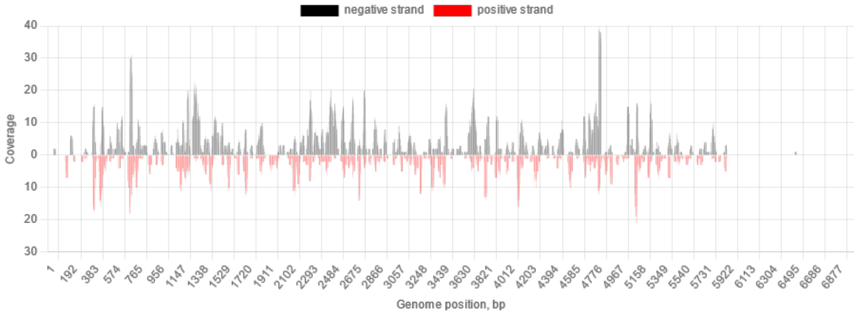

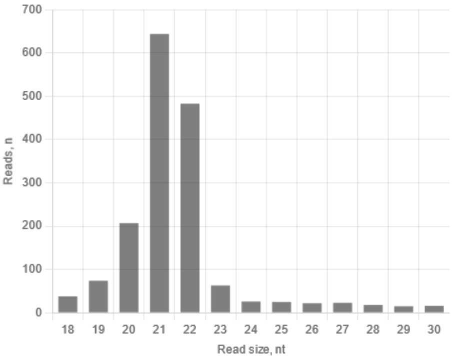

Mapping coverage of SMoV_RNA1A specific reads (1654 reads)

Length distribution of the reads

Dataset	Input reads	Virus	Reference	Mapped reads	Mapped reads, %	RPKM	Forward/Reverse balance	Nonredundant, %	Strain specific	Strain specific, % from total mapped reads	Strain specific, RPKM
S2_L001	10000000	SMoV	SMoV_RNA1B	3340	0.033	47.4	1.6	55.5	3924	117.5	23.2

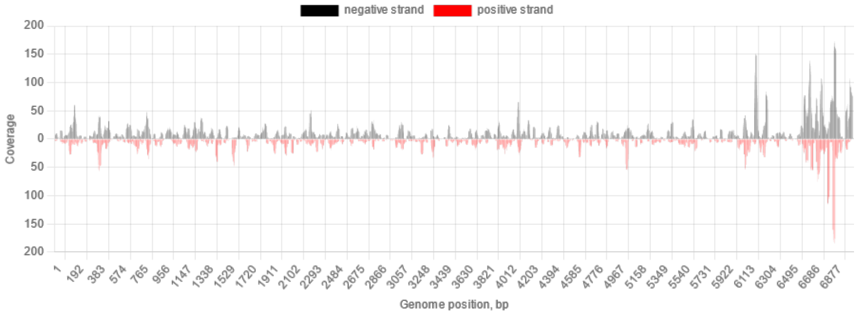

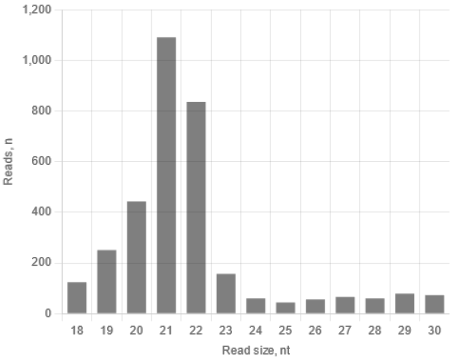

Mapping coverage of all reads matching SMoV_RNA1B (6533 reads)

Length distribution of the reads

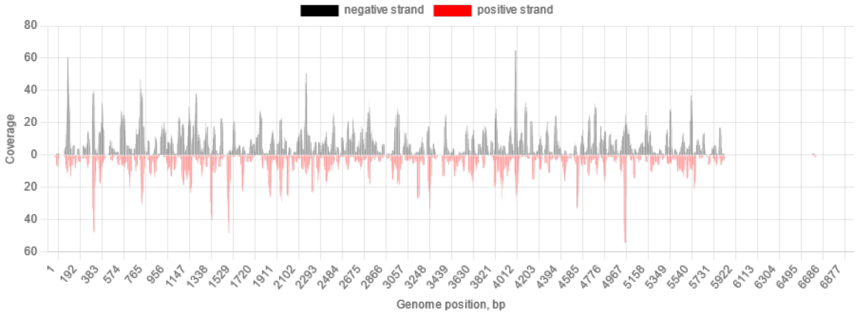

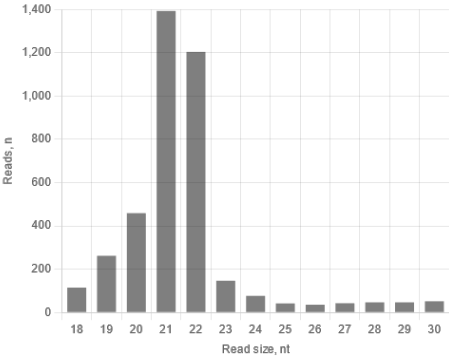

Mapping coverage of SMoV_RNA1B specific reads (3924 reads)

Length distribution of the reads

Dataset	Input reads	Virus	Reference	Mapped reads	Mapped reads, %	RPKM	Forward/Reverse balance	Nonredundant, %	Strain specific	Strain specific, % from total mapped reads	Strain specific, RPKM
S2_L001	10000000	SMoV	SMoV_RNA1C	3288	0.033	46.7	1.6	55.8	3373	102.6	23.2

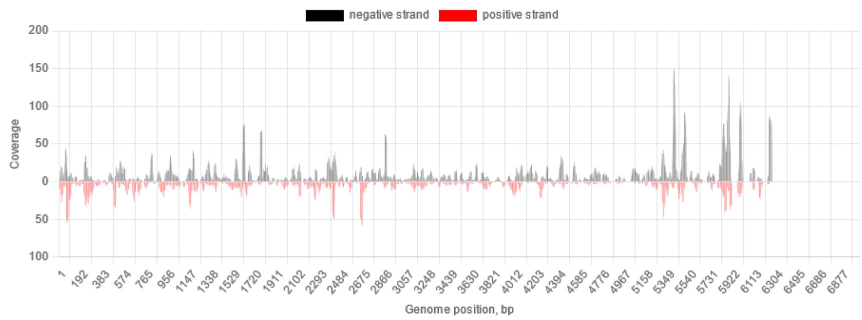

Mapping coverage of all reads matching SMoV_RNA1C (4707 reads)

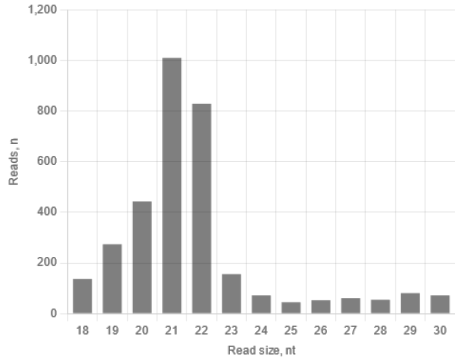

Length distribution of the reads

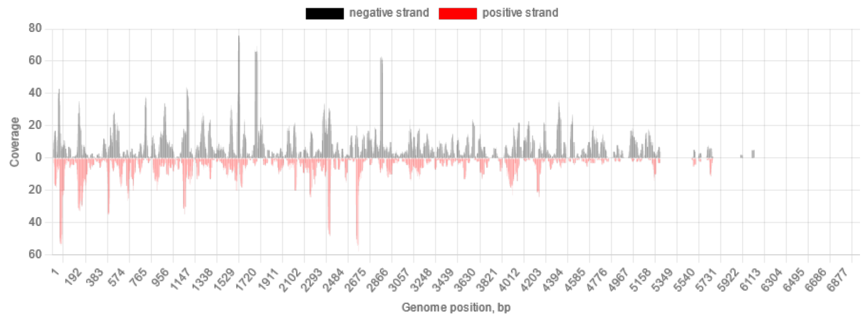

Mapping coverage of SMoV_RNA1C specific reads (3373 reads)

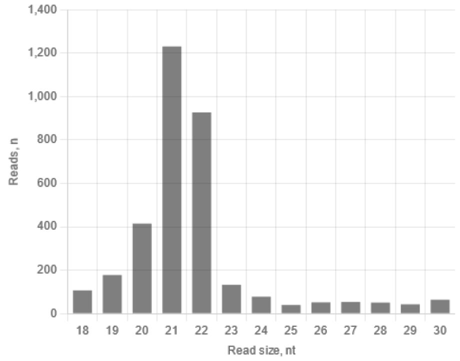

Length distribution of the reads

Dataset	Input reads	Virus	Reference	Mapped reads	Mapped reads, %	RPKM	Forward/Reverse balance	Nonredundant, %	Strain specific	Strain specific, % from total mapped reads	Strain specific, RPKM
S2_L001	10000000	SMoV	SMoV_RNA2A	3285	0.033	52.4	1.9	59.1	22	0.7	37.9

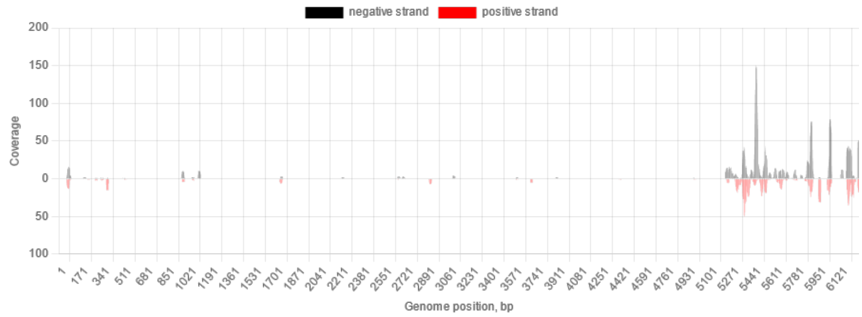

Mapping coverage of all reads matching SMoV_RNA2A (1338 reads)

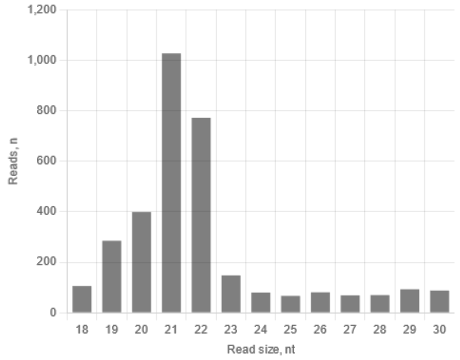

Length distribution of the reads

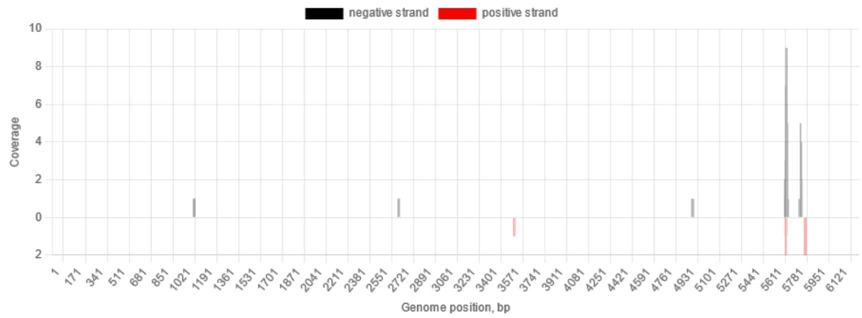

Mapping coverage of SMoV_RNA2A specific reads (22 reads)

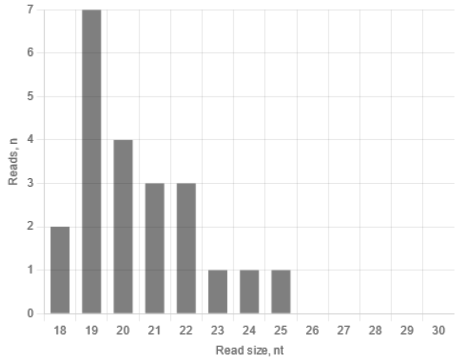

Length distribution of the reads

Dataset	Input reads	Virus	Reference	Mapped reads	Mapped reads, %	RPKM	Forward/Reverse balance	Nonredundant, %	Strain specific	Strain specific, % from total mapped reads	Strain specific, RPKM
S2_L001	10000000	SMoV	SMoV_RNA2B	896	0.009	14.2	2.9	52.1	3052	340.6	0.2

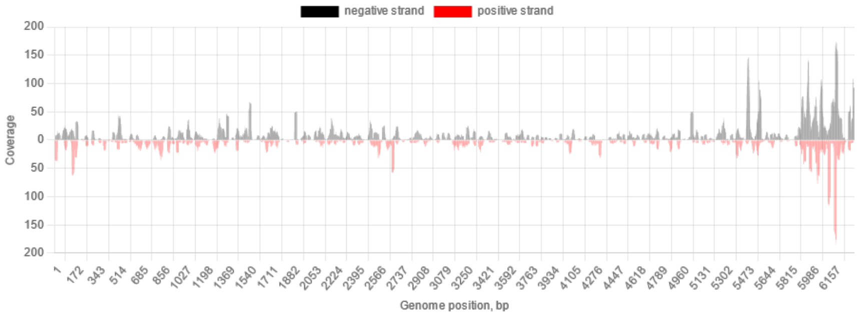

Mapping coverage of all reads matching SMoV_RNA2B (5494 reads)

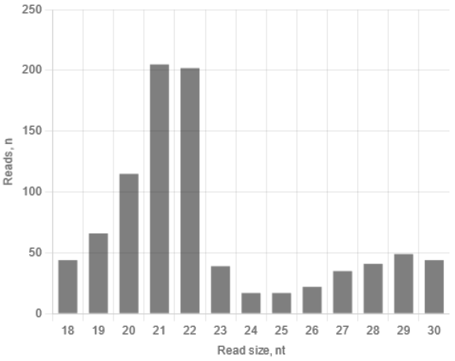

Length distribution of the reads

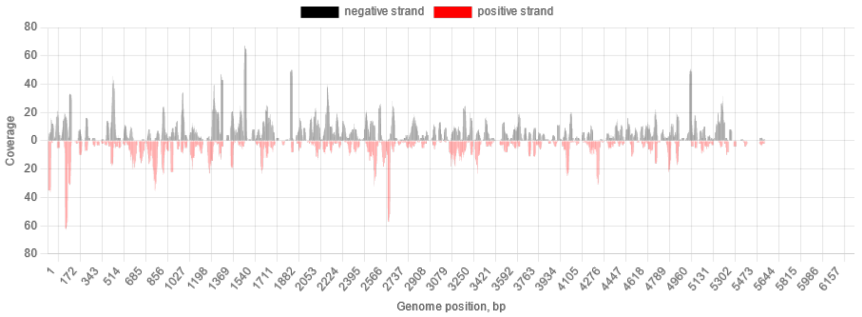

Mapping coverage of SMoV_RNA2B specific reads (3052 reads)

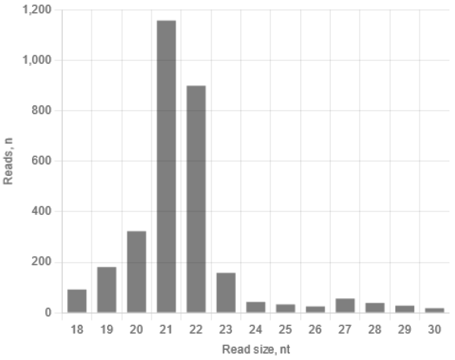

Length distribution of the reads

Dataset	Input reads	Virus	Reference	Mapped reads	Mapped reads, %	RPKM	Forward/Reverse balance	Nonredundant, %	Strain specific	Strain specific, % from total mapped reads	Strain specific, RPKM
S2_L001	10000000	SMoV	SMoV_RNA2C	2815	0.028	44.4	1.7	53.7	3	0.1	19.4

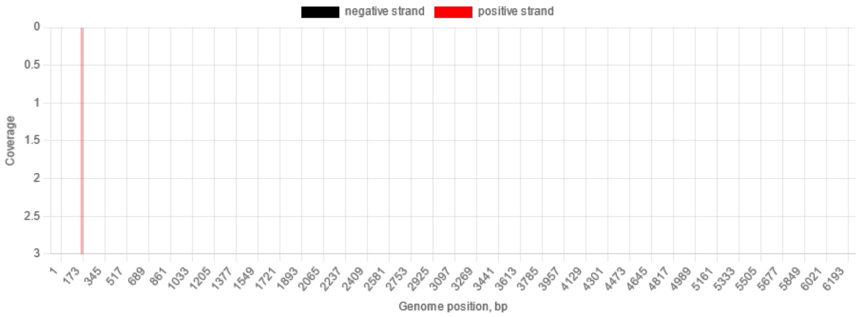

Mapping coverage of all reads matching SMoV_RNA2C (3 reads)

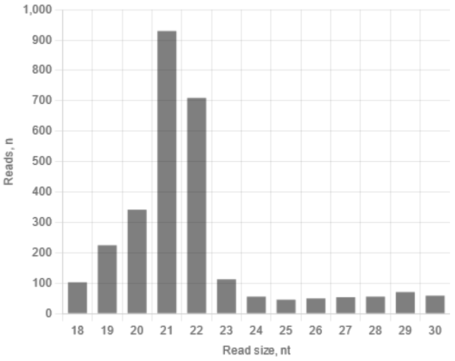

Length distribution of the reads

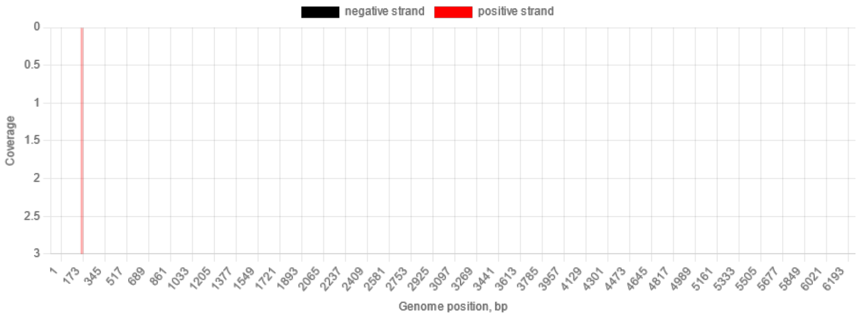

Mapping coverage of SMoV_RNA2C specific reads (3 reads)

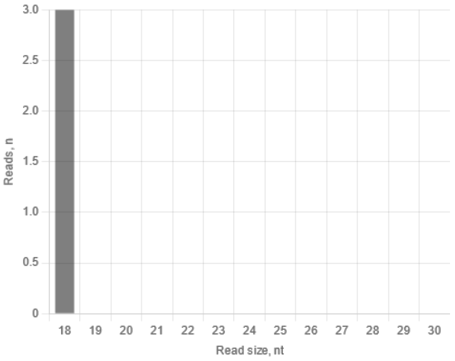

Length distribution of the reads

Mappings of the S3_L001 reads against the viral references.

Dataset	Input reads	Virus	Reference	Mapped reads	Mapped reads, %	RPKM	Forward/Reverse balance	Nonredundant, %	Strain specific	Strain specific, % from total mapped reads	Strain specific, RPKM
S3_L001	10000000	SMoV	SMoV_RNA1A	784	0.008	11.1	2.6	58.4	5	0.6	0.0

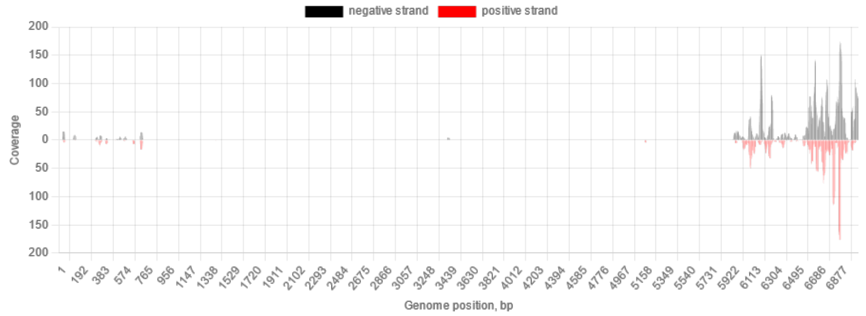

Mapping coverage of all reads matching SMoV_RNA1A (2553 reads)

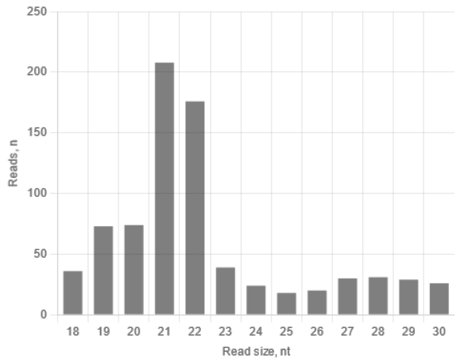

Length distribution of the reads

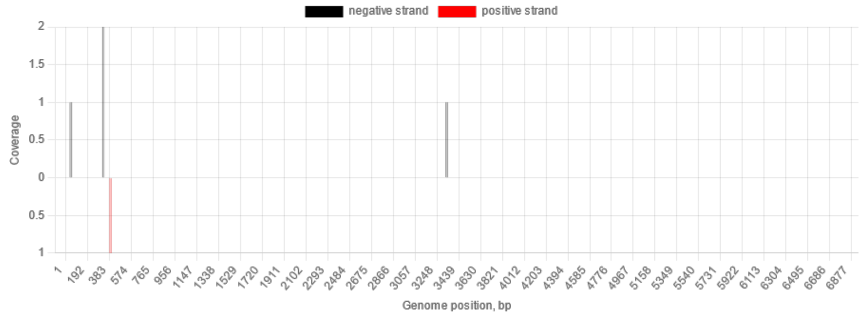

Mapping coverage of SMoV_RNA1A specific reads (5 reads)

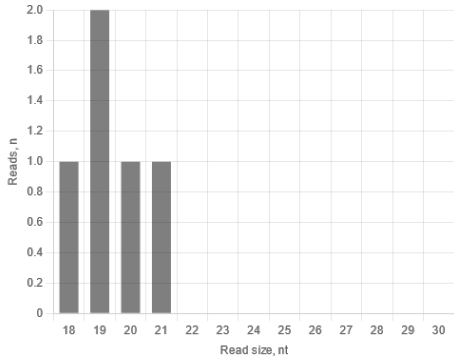

Length distribution of the reads

Dataset	Input reads	Virus	Reference	Mapped reads	Mapped reads, %	RPKM	Forward/Reverse balance	Nonredundant, %	Strain specific	Strain specific, % from total mapped reads	Strain specific, RPKM
S3_L001	10000000	SMoV	SMoV_RNA1B	1426	0.014	20.2	2.0	67.8	1654	116.0	8.7

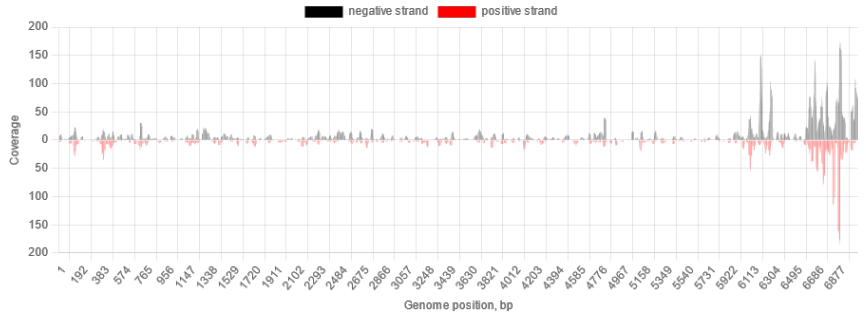

Mapping coverage of all reads matching SMoV_RNA1B (4259 reads)

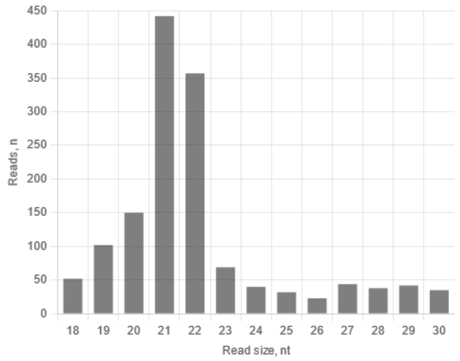

Length distribution of the reads

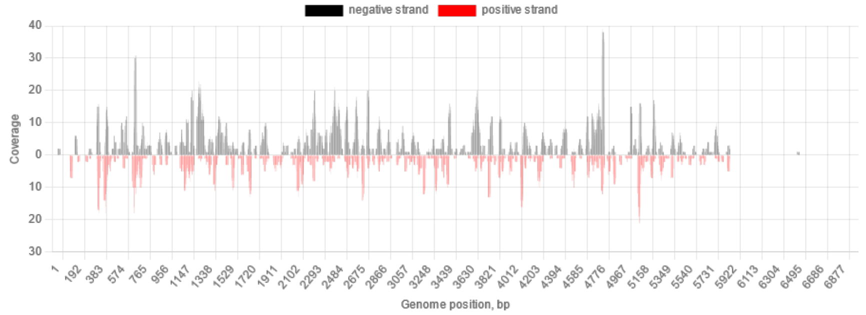

Mapping coverage of SMoV_RNA1B specific reads (1654 reads)

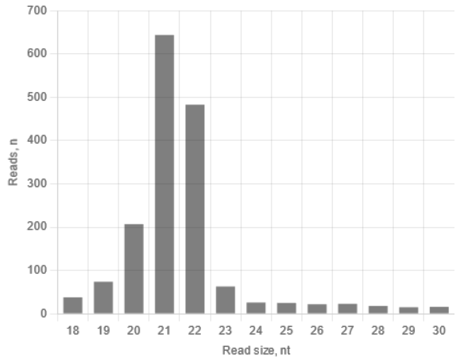

Length distribution of the reads

Dataset	Input reads	Virus	Reference	Mapped reads	Mapped reads, %	RPKM	Forward/Reverse balance	Nonredundant, %	Strain specific	Strain specific, % from total mapped reads	Strain specific, RPKM
S3_L001	10000000	SMoV	SMoV_RNA1C	1665	0.017	23.7	1.9	68.6	3924	235.7	12.3

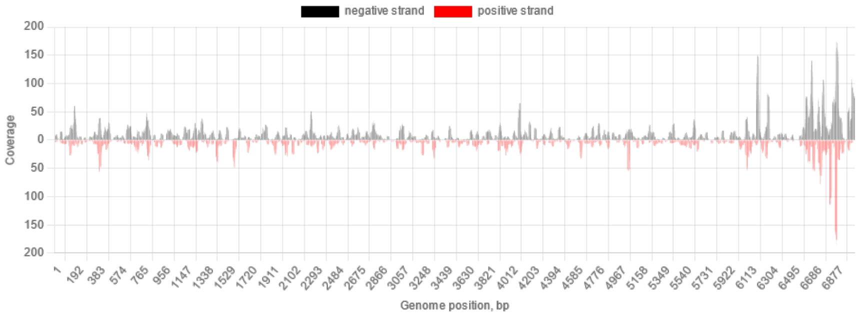

Mapping coverage of all reads matching SMoV_RNA1C (6533 reads)

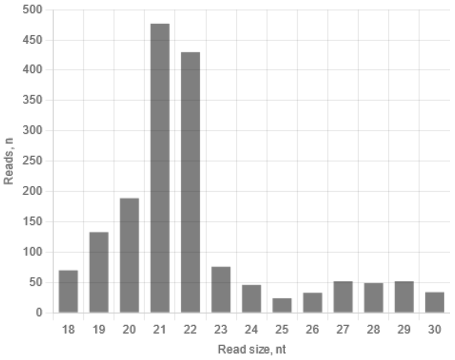

Length distribution of the reads

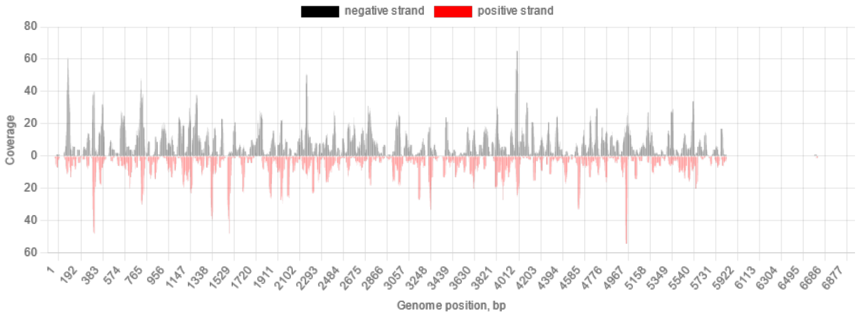

Mapping coverage of SMoV_RNA1C specific reads (3924 reads)

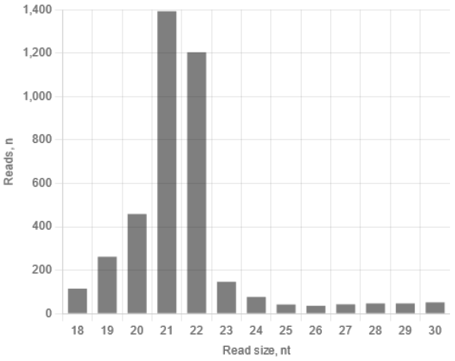

Length distribution of the reads

Dataset	Input reads	Virus	Reference	Mapped reads	Mapped reads, %	RPKM	Forward/Reverse balance	Nonredundant, %	Strain specific	Strain specific, % from total mapped reads	Strain specific, RPKM
S3_L001	10000000	SMoV	SMoV_RNA2A	1493	0.015	23.8	1.9	69.8	3373	225.9	17.1

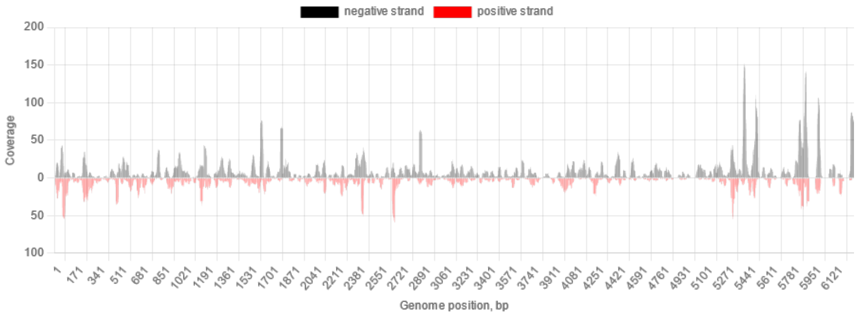

Mapping coverage of all reads matching SMoV_RNA2A (4707 reads)

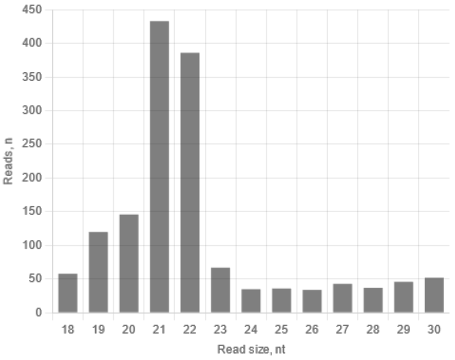

Length distribution of the reads

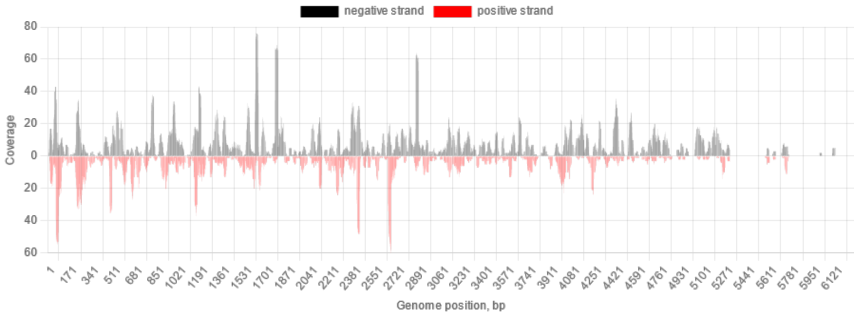

Mapping coverage of SMoV_RNA2A specific reads (3373 reads)

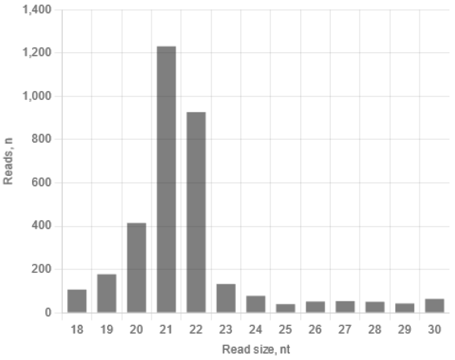

Length distribution of the reads

Dataset	Input reads	Virus	Reference	Mapped reads	Mapped reads, %	RPKM	Forward/Reverse balance	Nonredundant, %	Strain specific	Strain specific, % from total mapped reads	Strain specific, RPKM
S3_L001	10000000	SMoV	SMoV_RNA2B	416	0.004	6.6	2.9	66.3	22	5.3	0.1

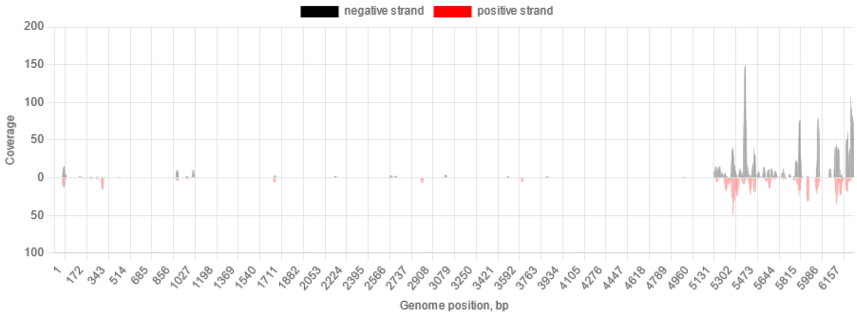

Mapping coverage of all reads matching SMoV_RNA2B (1338 reads)

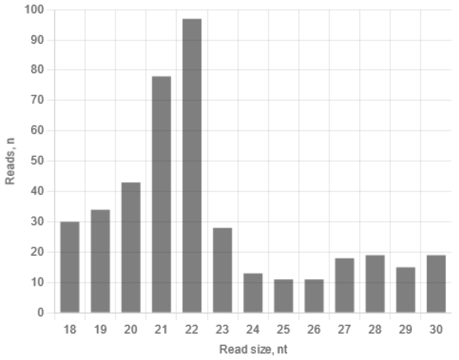

Length distribution of the reads

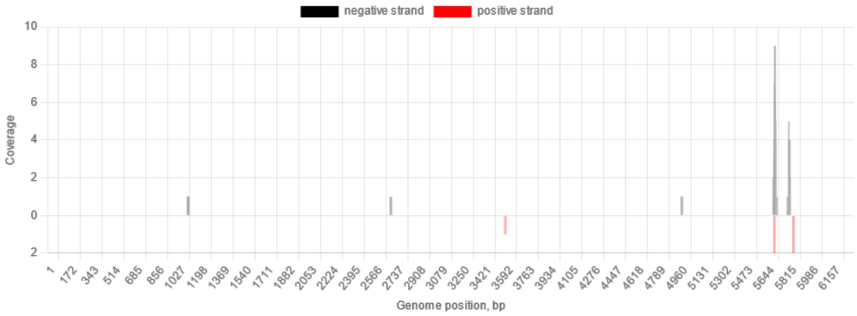

Mapping coverage of SMoV_RNA2B specific reads (22 reads)

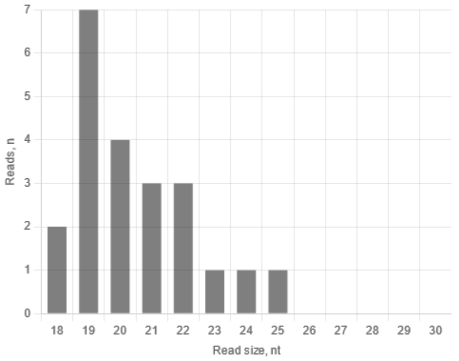

Length distribution of the reads

Dataset	Input reads	Virus	Reference	Mapped reads	Mapped reads, %	RPKM	Forward/Reverse balance	Nonredundant, %	Strain specific	Strain specific, % from total mapped reads	Strain specific, RPKM
S3_L001	10000000	SMoV	SMoV_RNA2C	1378	0.014	21.7	2.0	65.8	3052	221.5	10.0

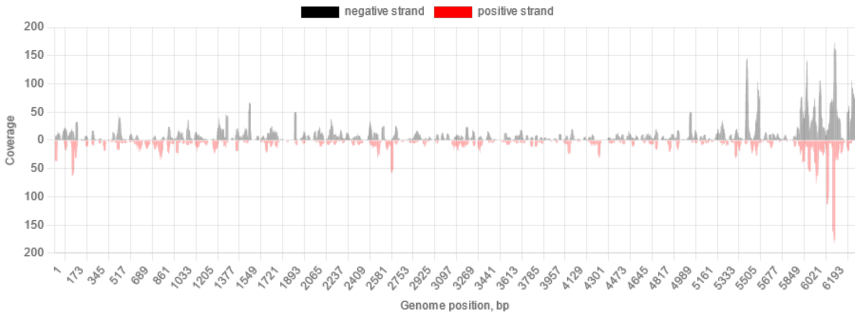

Mapping coverage of all reads matching SMoV_RNA2C (5494 reads)

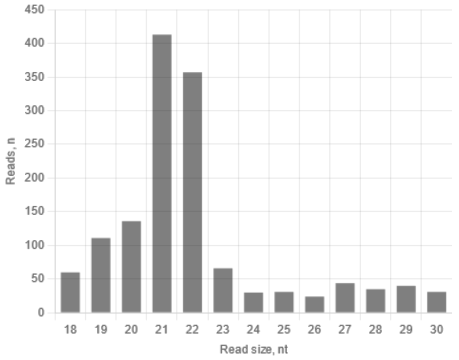

Length distribution of the reads

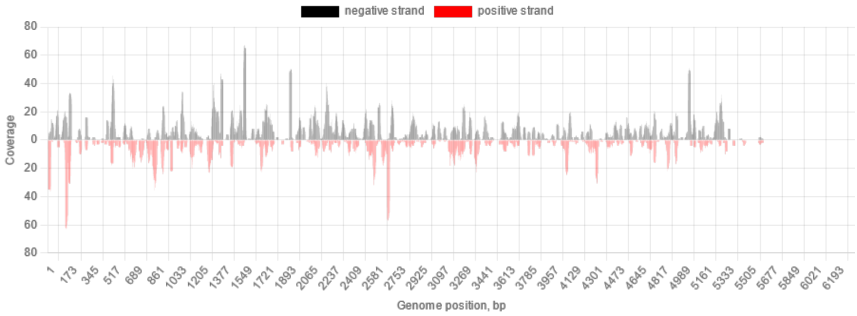

Mapping coverage of SMoV_RNA2C specific reads (3052 reads)

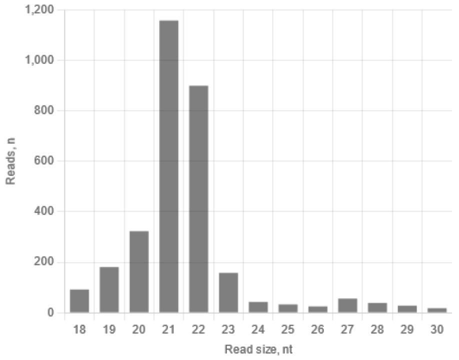

Length distribution of the reads

Mappings of the S4_L001 reads against the viral references. *qPCR detection: SMoV RNA1B,C & SMoV RNA2A,C*

Dataset	Input reads	Virus	Reference	Mapped reads	Mapped reads, %	RPKM	Forward/Reverse balance	Nonredundant, %	Strain specific	Strain specific, % from total mapped reads	Strain specific, RPKM
S4_L001	10000000	SMoV	SMoV_RNA1A	2763	0.028	39.3	1.4	39.0	5	0.2	0.0

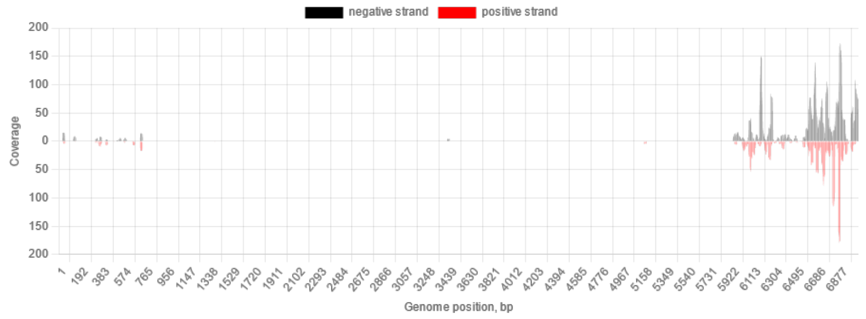

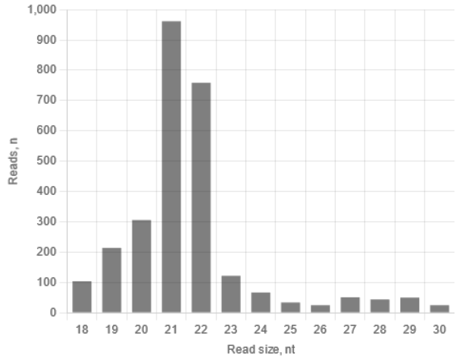

Mapping coverage of **all reads** matching SMoV_RNA1A (2553 reads) — Length distribution of the reads

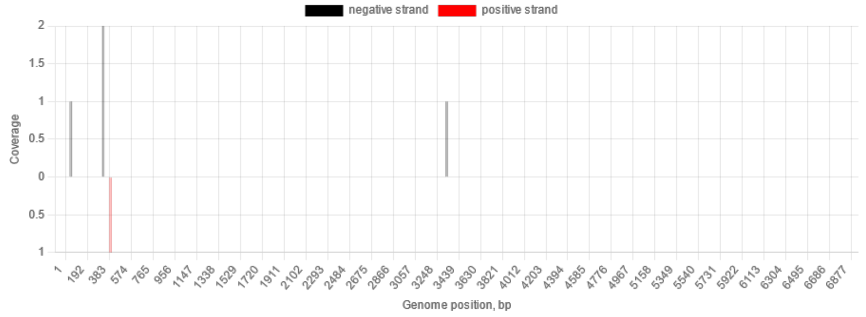

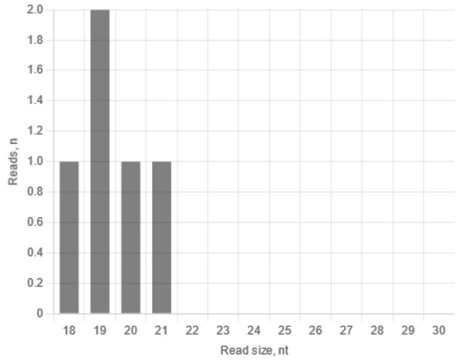

Mapping coverage of SMoV_RNA1A **specific reads** (5 reads) — Length distribution of the reads

Dataset	Input reads	Virus	Reference	Mapped reads	Mapped reads, %	RPKM	Forward/Reverse balance	Nonredundant, %	Strain specific	Strain specific, % from total mapped reads	Strain specific, RPKM
S4_L001	10000000	SMoV	SMoV_RNA1B	5012	0.050	71.1	1.3	49.3	1654	33.0	31.0

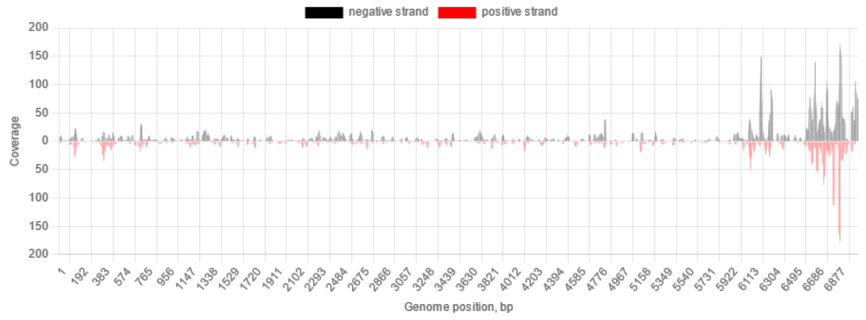

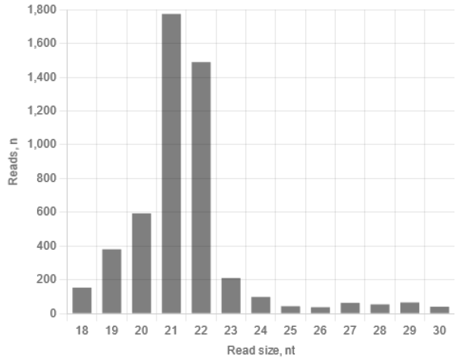

Mapping coverage of **all reads** matching SMoV_RNA1B (4259 reads) — Length distribution of the reads

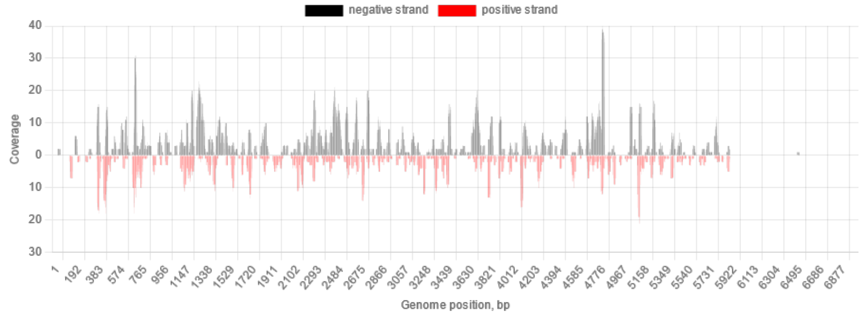

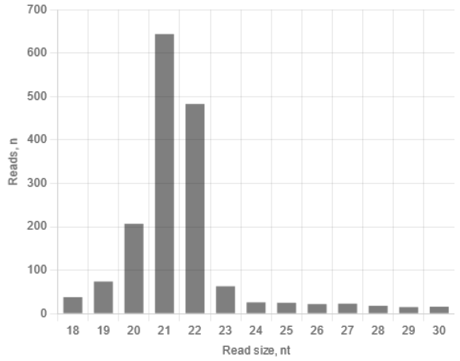

Mapping coverage of SMoV_RNA1B **specific reads** (1654 reads) — Length distribution of the reads

Dataset	Input reads	Virus	Reference	Mapped reads	Mapped reads, %	RPKM	Forward/Reverse balance	Nonredundant, %	Strain specific	Strain specific, % from total mapped reads	Strain specific, RPKM
S4_L001	10000000	SMoV	SMoV_RNA1C	6659	0.067	94.6	1.3	50.6	3924	58.9	54.5

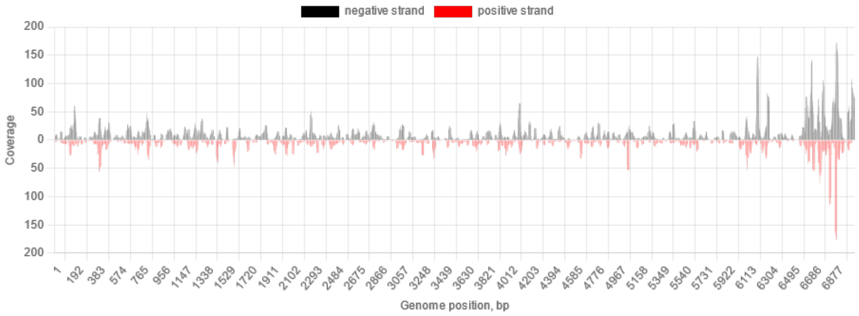

Mapping coverage of all reads matching SMoV_RNA1C (6533 reads)

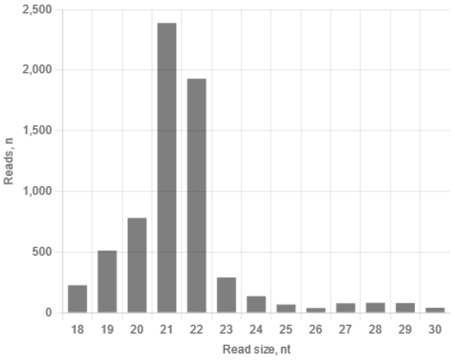

Length distribution of the reads

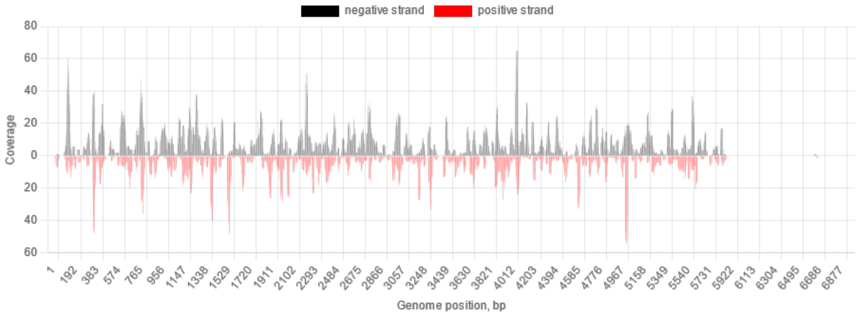

Mapping coverage of SMoV_RNA1C specific reads (3924 reads)

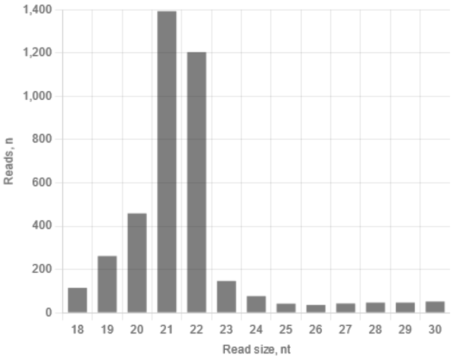

Length distribution of the reads

Dataset	Input reads	Virus	Reference	Mapped reads	Mapped reads, %	RPKM	Forward/Reverse balance	Nonredundant, %	Strain specific	Strain specific, % from total mapped reads	Strain specific, RPKM
S4_L001	10000000	SMoV	SMoV_RNA2A	4572	0.046	72.9	1.5	55.3	3373	73.8	51.5

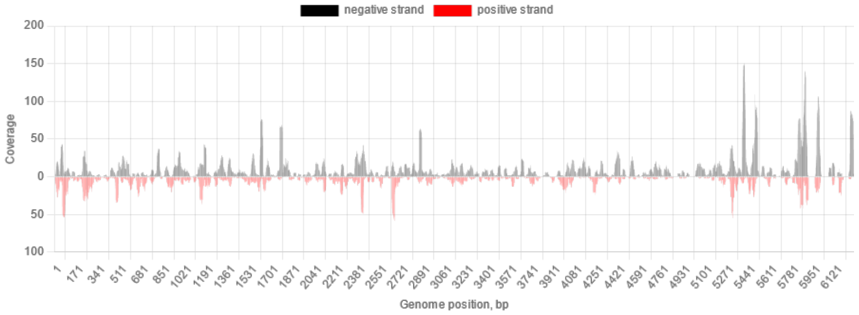

Mapping coverage of all reads matching SMoV_RNA2A (4707 reads)

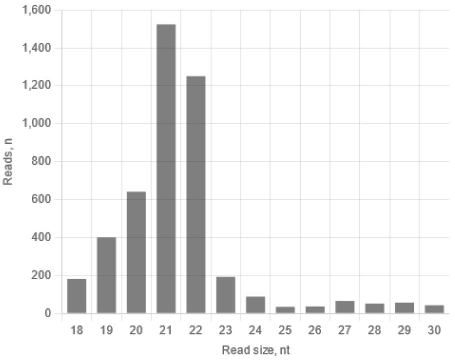

Length distribution of the reads

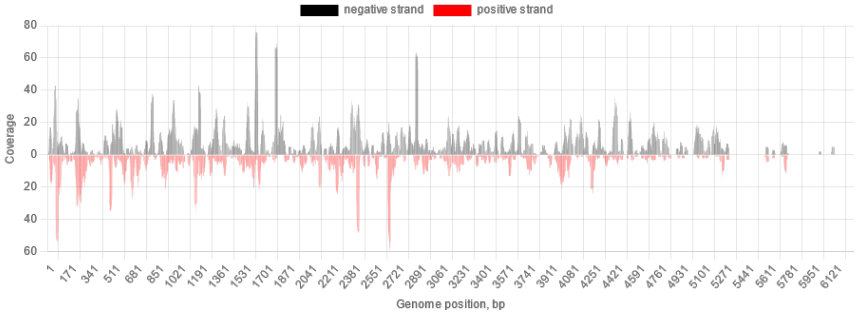

Mapping coverage of SMoV_RNA2A specific reads (3373 reads)

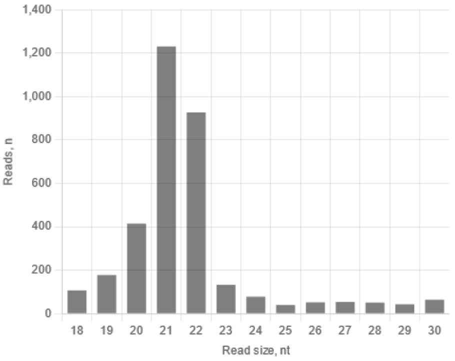

Length distribution of the reads

Dataset	Input reads	Virus	Reference	Mapped reads	Mapped reads, %	RPKM	Forward/Reverse balance	Nonredundant, %	Strain specific	Strain specific, % from total mapped reads	Strain specific, RPKM
S4_L001	10000000	SMoV	SMoV_RNA2B	1373	0.014	21.7	1.8	51.0	22	1.6	0.4

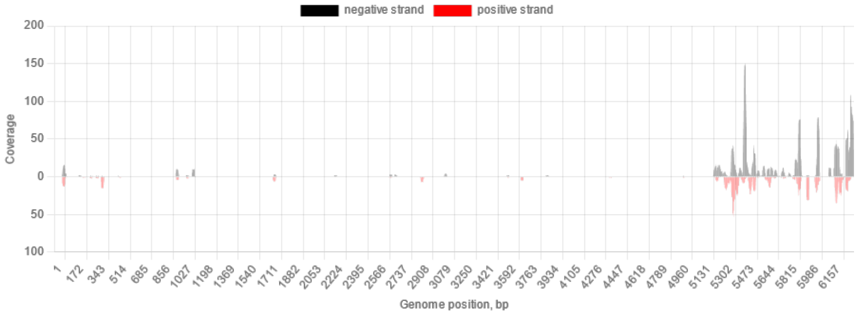

Mapping coverage of all reads matching SMoV_RNA2B (1338 reads)

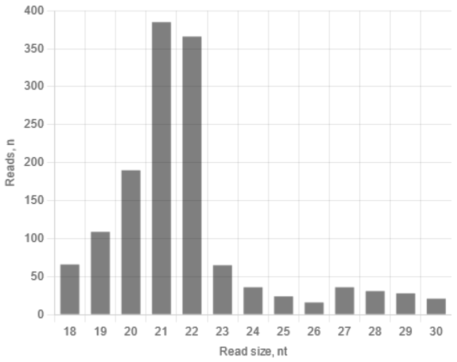

Length distribution of the reads

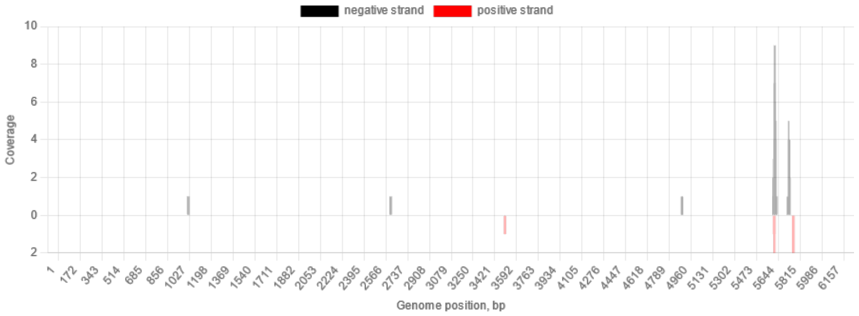

Mapping coverage of SMoV_RNA2B specific reads (22 reads)

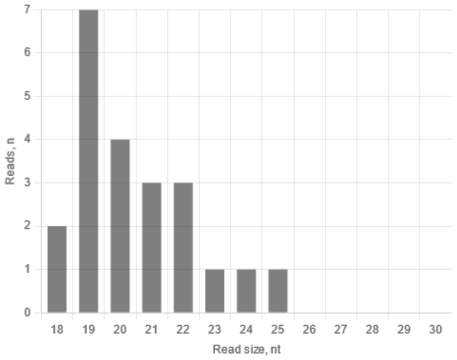

Length distribution of the reads

Dataset	Input reads	Virus	Reference	Mapped reads	Mapped reads, %	RPKM	Forward/Reverse balance	Nonredundant, %	Strain specific	Strain specific, % from total mapped reads	Strain specific, RPKM
S4_L001	10000000	SMoV	SMoV_RNA2C	5507	0.055	86.9	1.4	49.0	3052	55.4	44.8

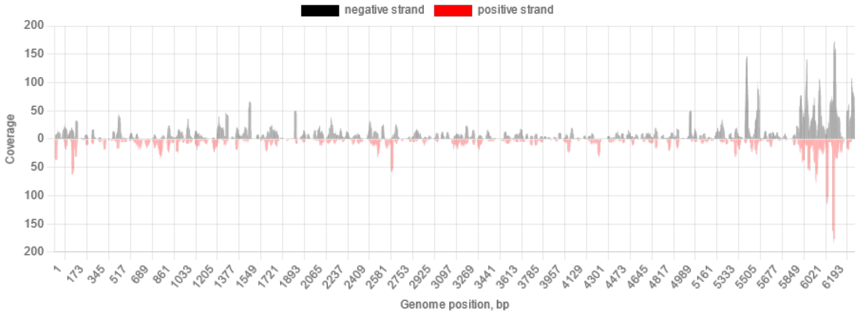

Mapping coverage of all reads matching SMoV_RNA2C (5494 reads)

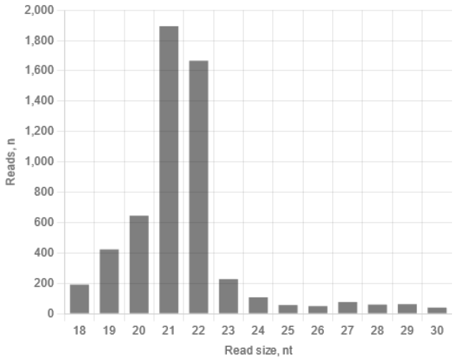

Length distribution of the reads

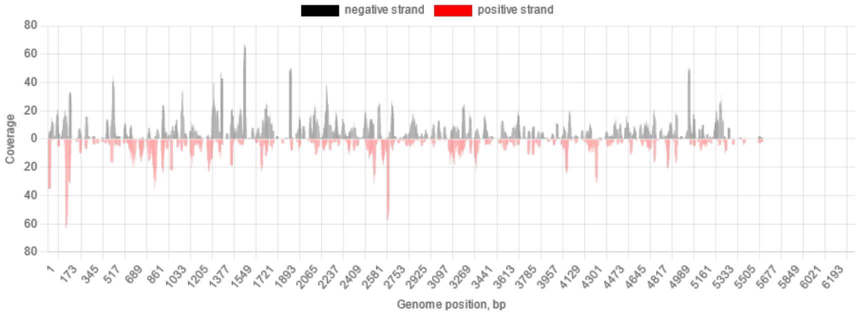

Mapping coverage of SMoV_RNA2C specific reads (3052 reads)

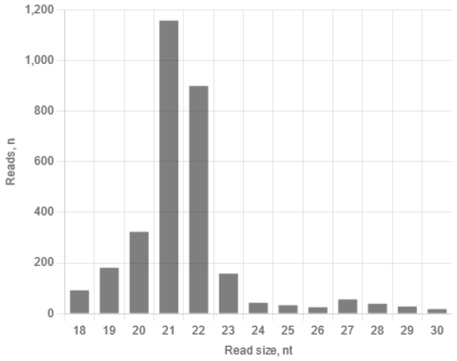

Length distribution of the reads

Mappings of the S92_L004 reads against the viral references. *qPCR detection: SMoV RNA1B,C & SMoV RNA2A,C*

Dataset	Input reads	Virus	Reference	Mapped reads	Mapped reads, %	RPKM	Forward/Reverse balance	Nonredundant, %	Strain specific	Strain specific, % from total mapped reads	Strain specific, RPKM
S92_L004	10000000	SMoV	SMoV_RNA1A	4093	0.041	58.2	1.2	31.3	5	0.1	0.5

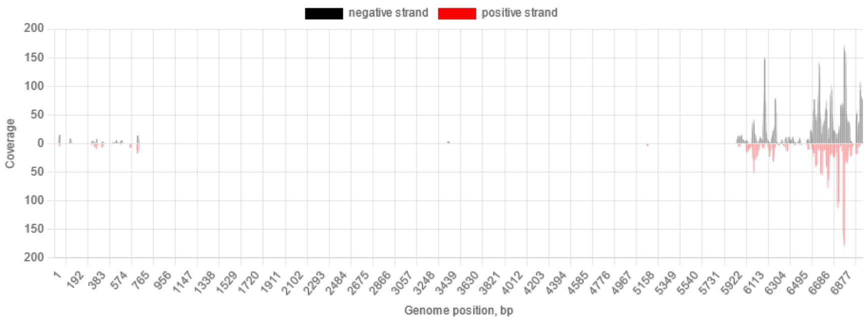

Mapping coverage of all reads matching SMoV_RNA1A (2553 reads)

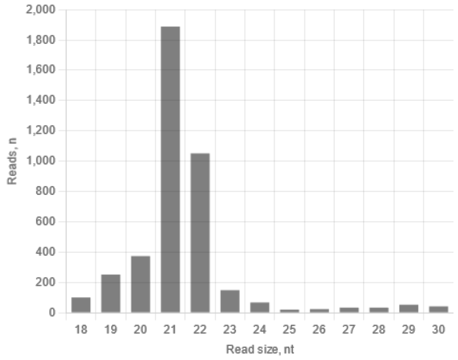

Length distribution of the reads

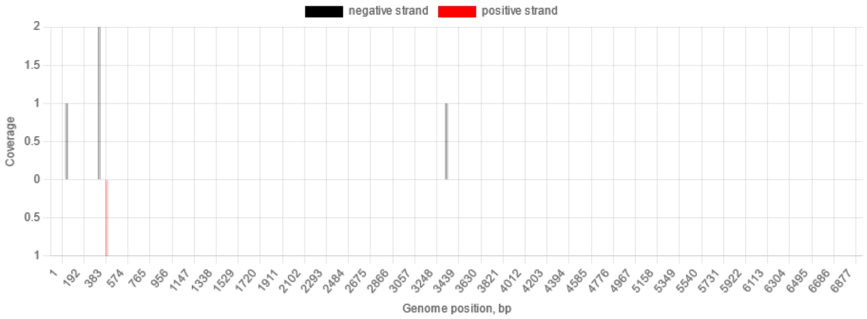

Mapping coverage of SMoV_RNA1A specific reads (5 reads)

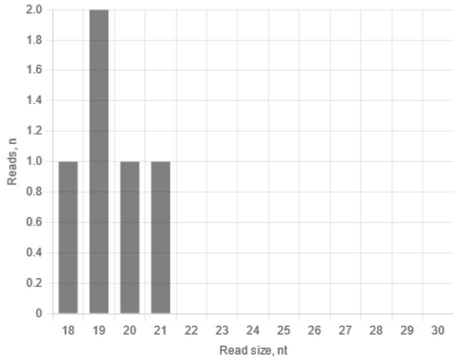

Length distribution of the reads

Dataset	Input reads	Virus	Reference	Mapped reads	Mapped reads, %	RPKM	Forward/Reverse balance	Nonredundant, %	Strain specific	Strain specific, % from total mapped reads	Strain specific, RPKM
S92_L004	10000000	SMoV	SMoV_RNA1B	9598	0.096	136.2	1.2	41.4	1654	17.2	76.2

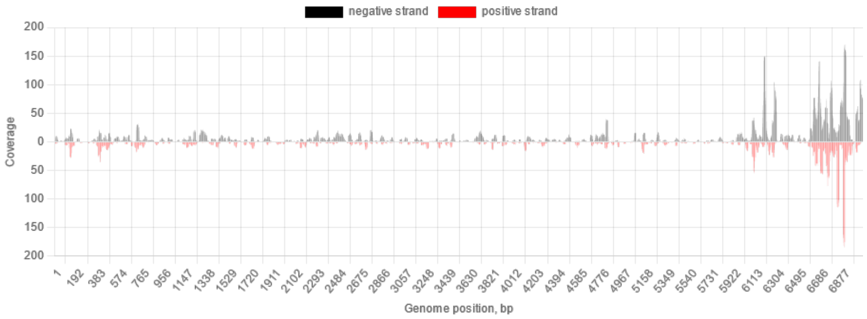

Mapping coverage of all reads matching SMoV_RNA1B (4259 reads)

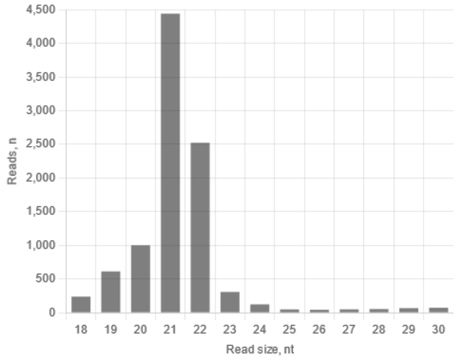

Length distribution of the reads

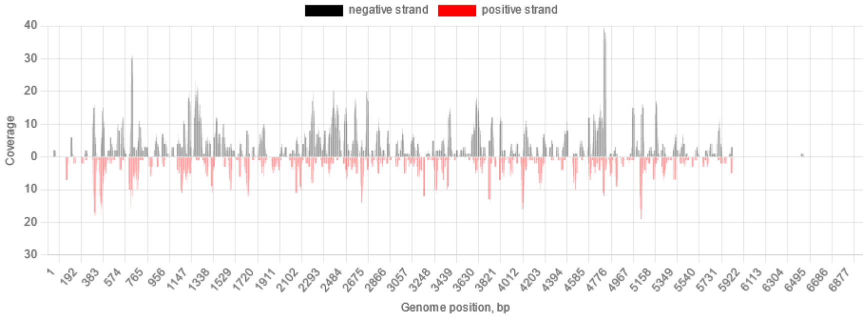

Mapping coverage of SMoV_RNA1B specific reads (1654 reads)

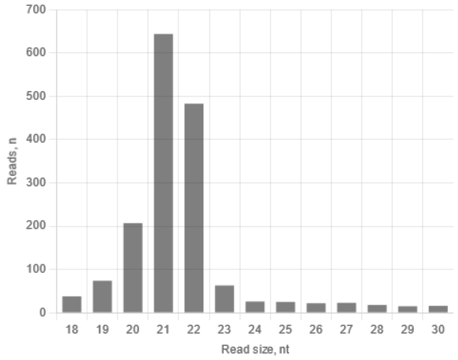

Length distribution of the reads

Dataset	Input reads	Virus	Reference	Mapped reads	Mapped reads, %	RPKM	Forward/Reverse balance	Nonredundant, %	Strain specific	Strain specific, % from total mapped reads	Strain specific, RPKM
S92_L004	10000000	SMoV	SMoV_RNA1C	10078	0.101	143.2	1.3	41.8	3924	38.9	84.7

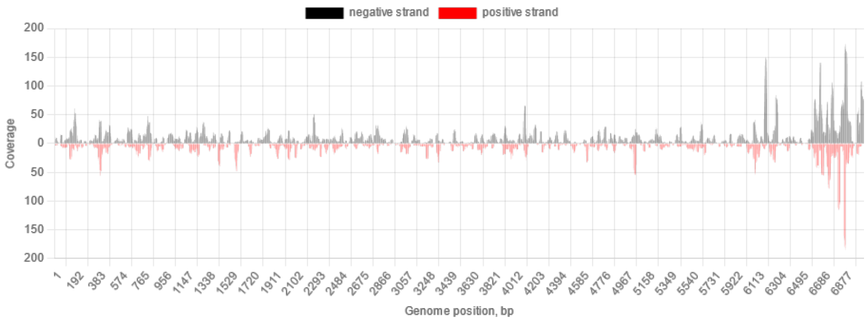

Mapping coverage of all reads matching SMoV_RNA1C (6533 reads)

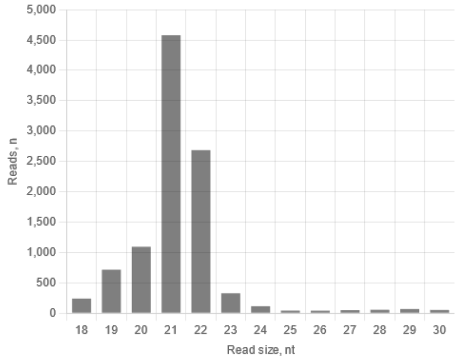

Length distribution of the reads

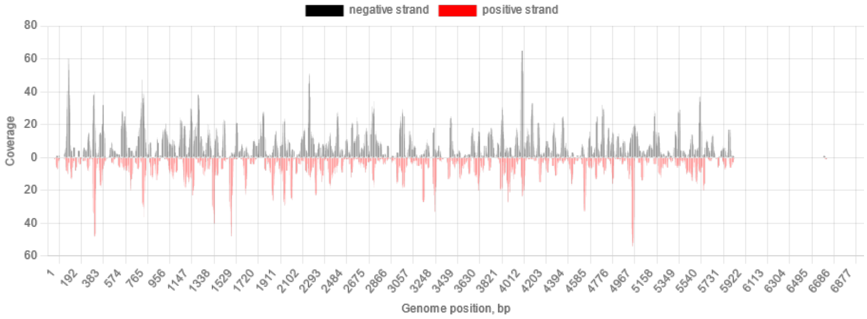

Mapping coverage of SMoV_RNA1C specific reads (3924 reads)

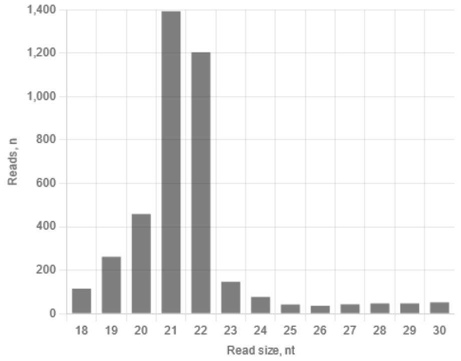

Length distribution of the reads

Dataset	Input reads	Virus	Reference	Mapped reads	Mapped reads, %	RPKM	Forward/Reverse balance	Nonredundant, %	Strain specific	Strain specific, % from total mapped reads	Strain specific, RPKM
S92_L004	10000000	SMoV	SMoV_RNA2A	6763	0.068	107.9	1.6	48.0	3373	49.9	77.7

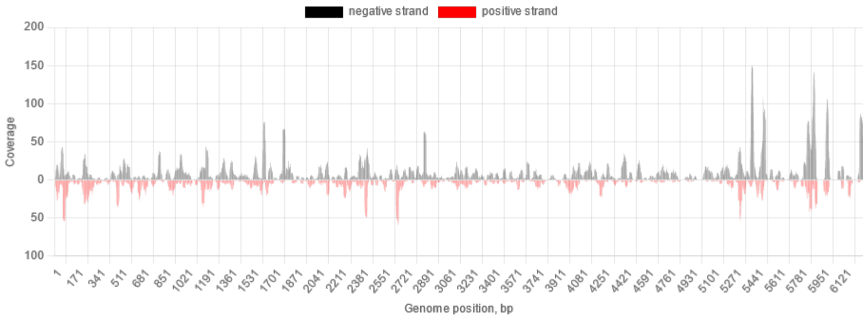

Mapping coverage of all reads matching SMoV_RNA2A (4707 reads)

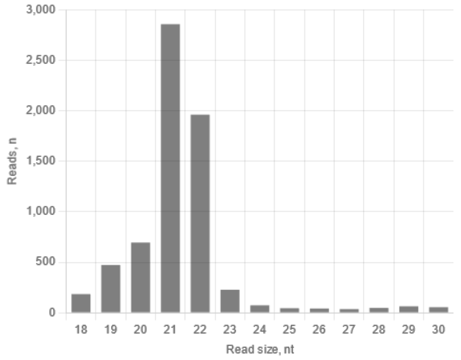

Length distribution of the reads

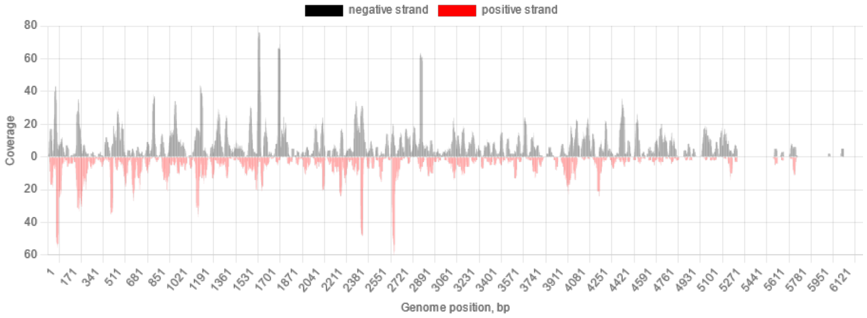

Mapping coverage of SMoV_RNA2A specific reads (3373 reads)

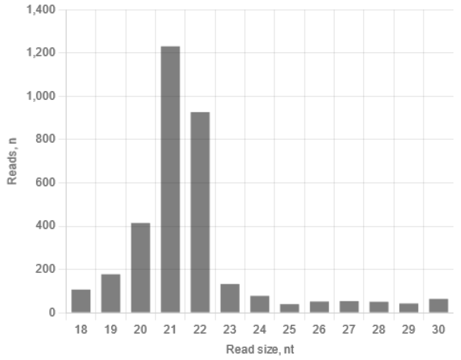

Length distribution of the reads

Dataset	Input reads	Virus	Reference	Mapped reads	Mapped reads, %	RPKM	Forward/Reverse balance	Nonredundant, %	Strain specific	Strain specific, % from total mapped reads	Strain specific, RPKM
S92_L004	10000000	SMoV	SMoV_RNA2B	2170	0.022	34.3	1.9	43.1	22	1.0	0.6

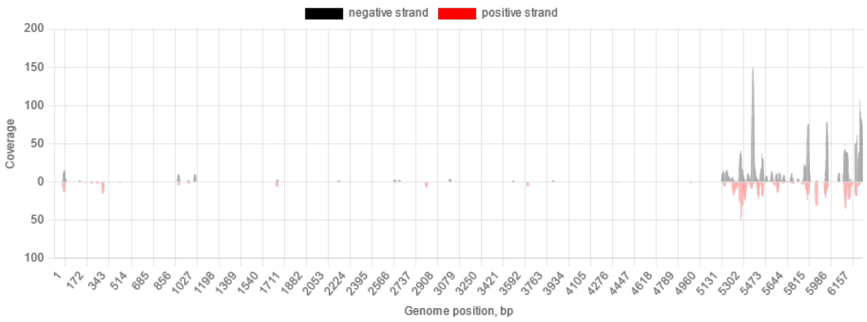

Mapping coverage of all reads matching SMoV_RNA2B (1338 reads)

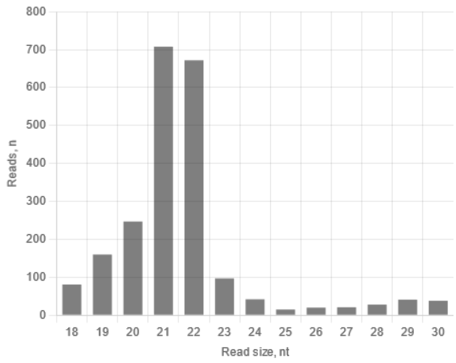

Length distribution of the reads

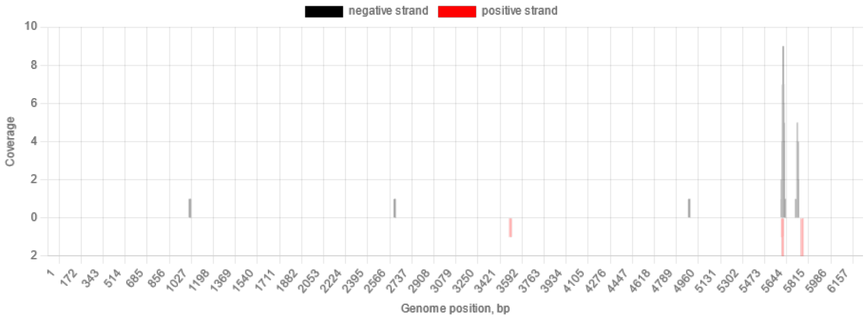

Mapping coverage of SMoV_RNA2B specific reads (22 reads)

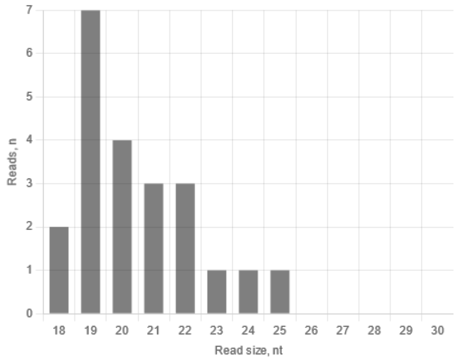

Length distribution of the reads

Dataset	Input reads	Virus	Reference	Mapped reads	Mapped reads, %	RPKM	Forward/Reverse balance	Nonredundant, %	Strain specific	Strain specific, % from total mapped reads	Strain specific, RPKM
S92_L004	10000000	SMoV	SMoV_RNA2C	11300	0.113	178.3	1.3	39.8	3052	27.0	114.2

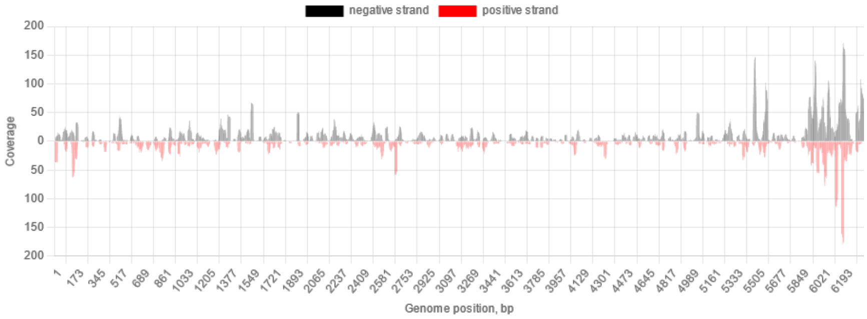

Mapping coverage of all reads matching SMoV_RNA2C (5494 reads)

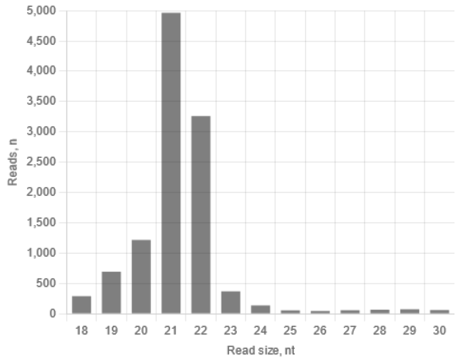

Length distribution of the reads

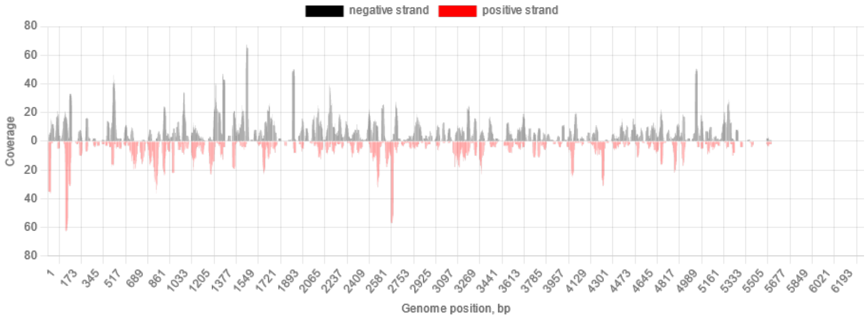

Mapping coverage of SMoV_RNA2C specific reads (3052 reads)

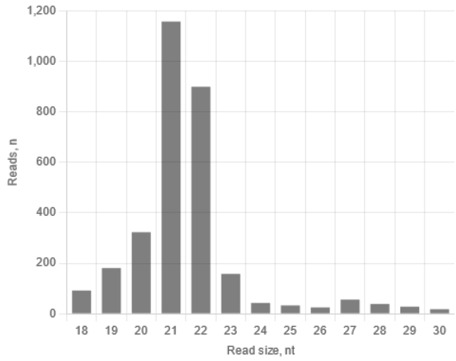

Length distribution of the reads

Dataset	Input reads	Virus	Reference	Mapped reads	Mapped reads, %	RPKM	Forward/Reverse balance	Nonredundant, %	Strain specific	Strain specific, % from total mapped reads	Strain specific, RPKM
S92_L004	10000000	StrV1	StrV1_A	38	0.000	0.3	0.7	97.4	3	7.9	0.2

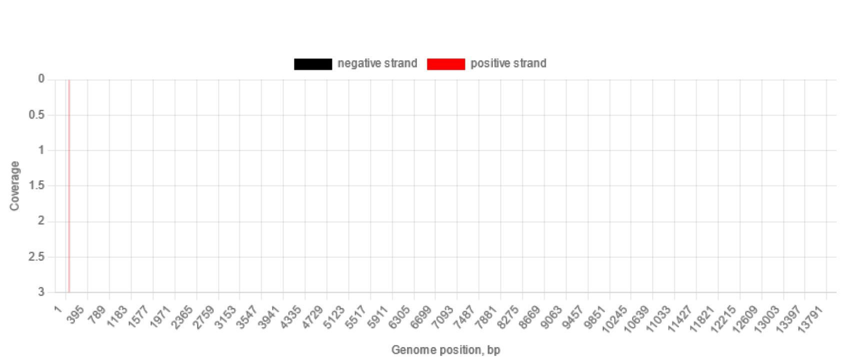

Mapping coverage of all reads matching StrV1_A (3 reads)

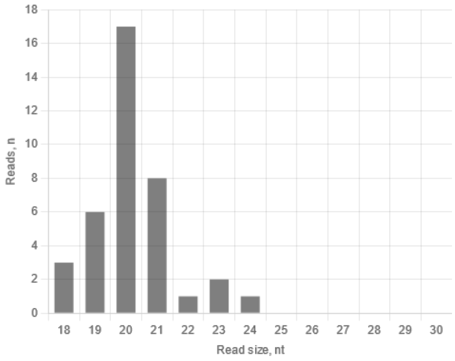

Length distribution of the reads

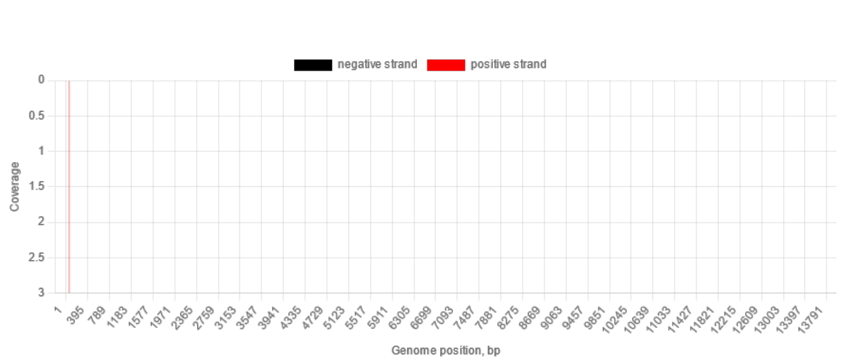

Mapping coverage of StrV1_A specific reads (3 reads)

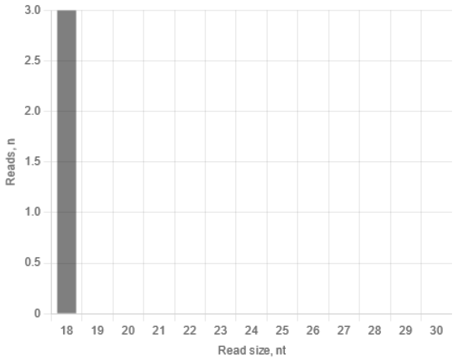

Length distribution of the reads

Dataset	Input reads	Virus	Reference	Mapped reads	Mapped reads, %	RPKM	Forward/Reverse balance	Nonredundant, %	Strain specific	Strain specific, % from total mapped reads	Strain specific, RPKM
S92_L004	10000000	StrV1	StrV1_B	20	0.000	0.1	1.5	100.0	0	0.0	0.1

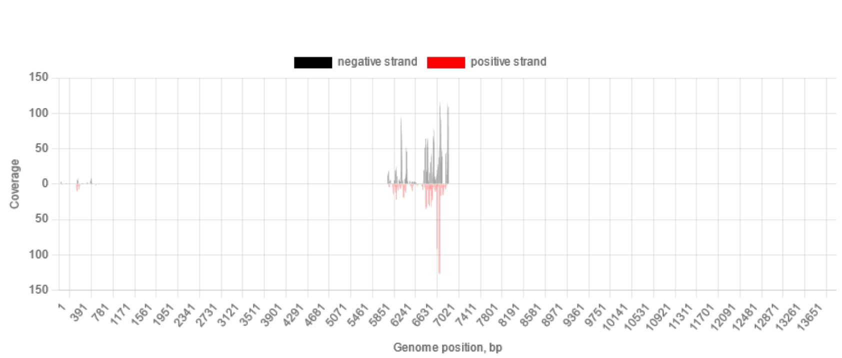

Mapping coverage of all reads matching StrV1_B (1629 reads)

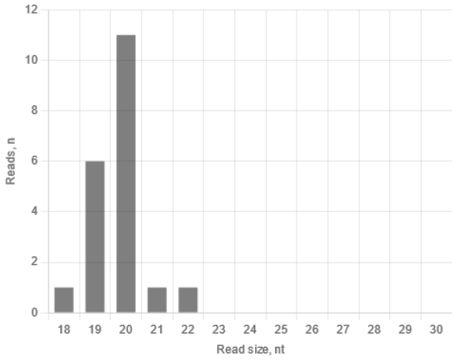

Length distribution of the reads

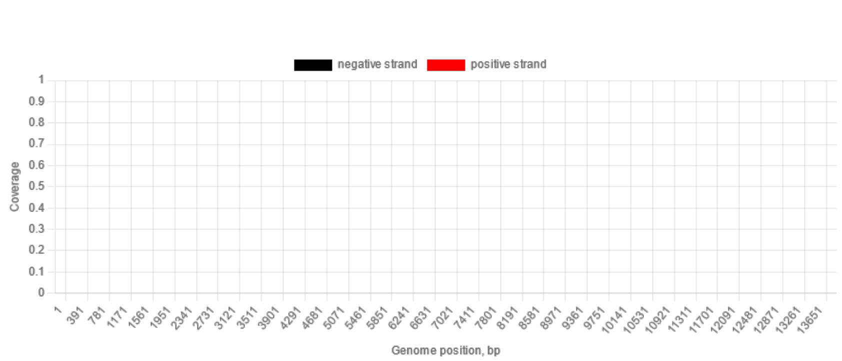

Mapping coverage of StrV1_B specific reads (0 reads)

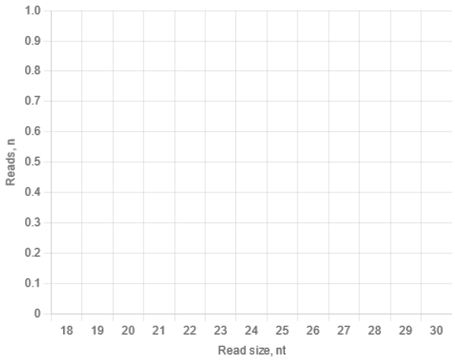

Length distribution of the reads
