# Supplementary material for: Analysis of Virus-Derived siRNAs in Strawberry Plants Co-Infected with Multiple Viruses and Their Genotypes
Source: Plants (Basel). 2023 Jul 6;12(13):2564. doi: 10.3390/plants12132564 (PMC10346599; doi:10.3390/plants12132564)
Supplement: Supplementary file 1 [file plants-12-02564-s001.zip › Figure S3.pdf]

Figure S3 Mapping and distribution plots of vsiRNAs (reads) from the Fv_StrV-1_A, Fv_StrV-1_B, and Fv_StrV-1_AB samples.

Plant Fv_StrV-1_A –datasets S95, S98

Plant Fv_StrV-1_B –datasets S99, S107, S108

Plant Fv_StrV-1_AB –dataset S101

Color legends show strand specificity of the mapped reads. Axes of abscissas show genomic positionin nucleotides, axes of ordinary – sequence (mapping) coverage.

Mappings of the S101 reads against the viral references.

Dataset	Input reads	Virus	Reference	Mapped reads	Mapped reads, %	Forward/Reverse balance	Nonredundant, %	Strain specific	Strain specific, %from total mapped reads
S101	10000000	SMoV	SMoV_RNA1A	73	0.001	1.6	89.0	11	15.1

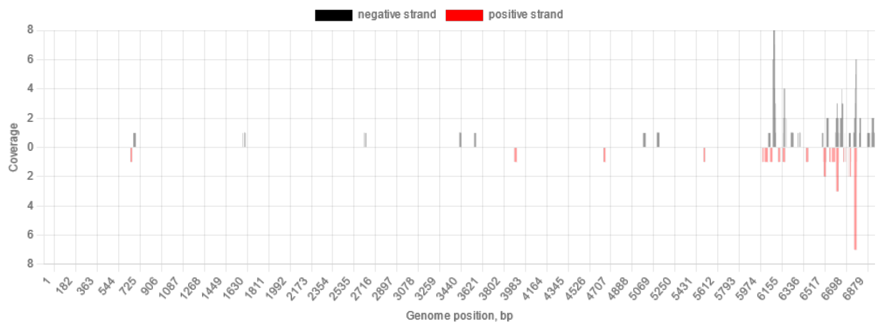

Mapping coverage of all reads matching SMoV_RNA1A (73 reads)

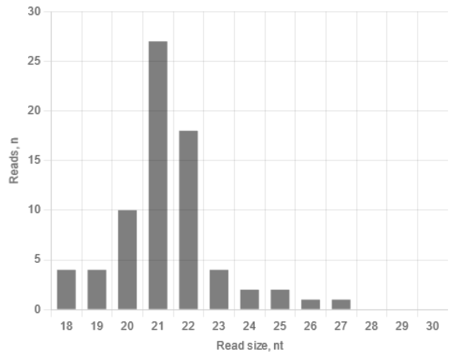

Length distribution of the reads

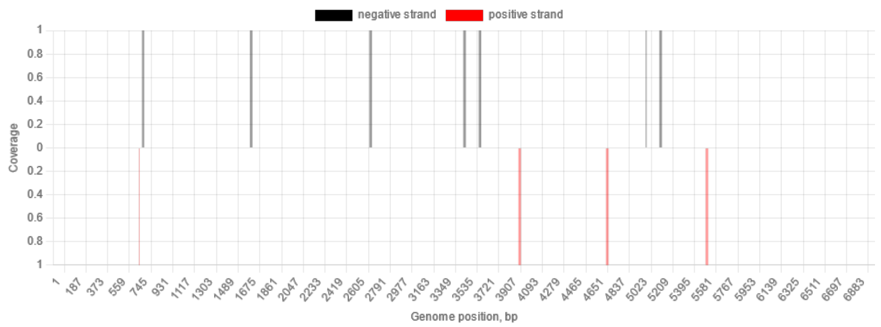

Mapping coverage of SMoV_RNA1A specific reads (11reads)

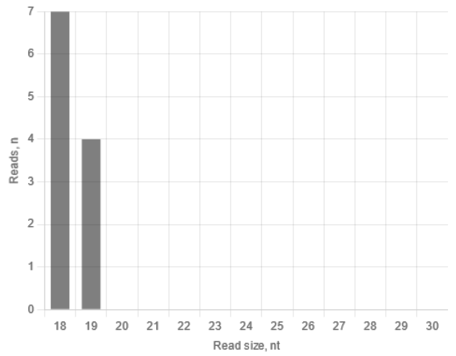

Length distribution of the reads

Dataset	Input reads	Virus	Reference	Mapped reads	Mapped reads, %	Forward/Reverse balance	Nonredundant, %	Strain specific	Strain specific, % from total mapped reads
S101	10000000	SMoV	SMoV_RNA1B	103	0.001	1.5	88.3	39	37.9

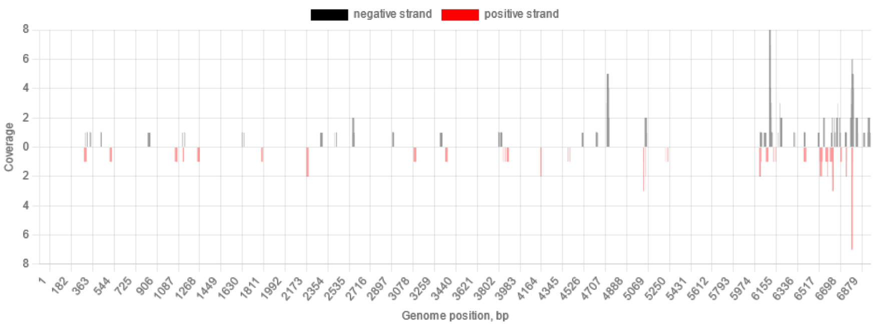

Mapping coverage of all reads matching SMoV_RNA1B (103 reads)

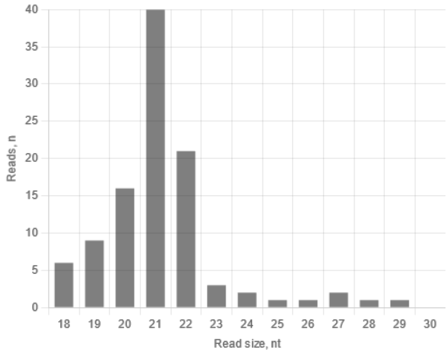

Length distribution of the reads

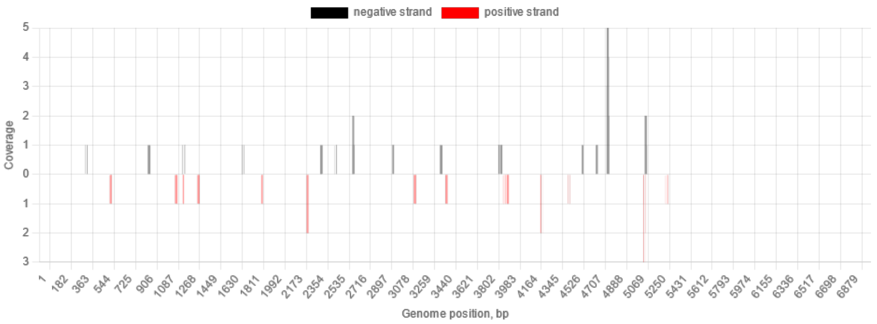

Mapping coverage of SMoV_RNA1B specific reads (39 reads)

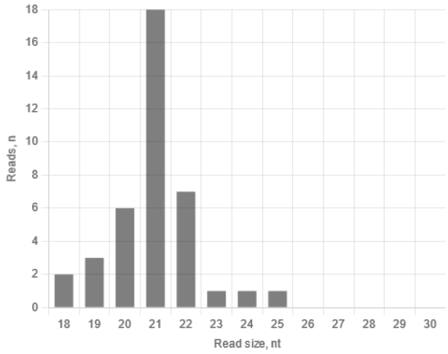

Length distribution of the reads

Dataset	Input reads	Virus	Reference	Mapped reads	Mapped reads, %	Forward/Reverse balance	Nonredundant, %	Strain specific	Strain specific, % from total mapped reads
S101	10000000	SMoV	SMoV_RNA1C	137	0.001	1.6	90.5	73	53.3

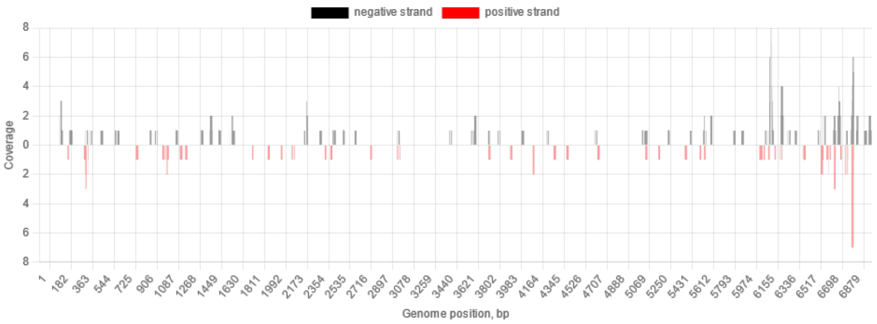

Mapping coverage of all reads matching SMoV_RNA1C (137 reads)

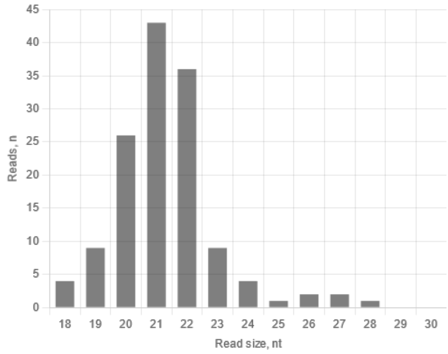

Length distribution of the reads

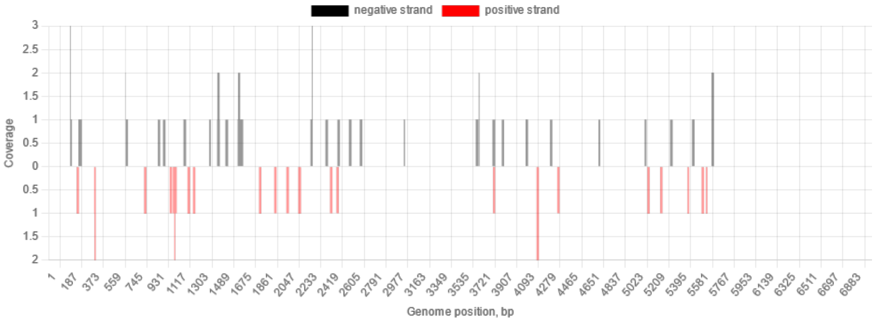

Mapping coverage of SMoV_RNA1C specific reads (73 reads)

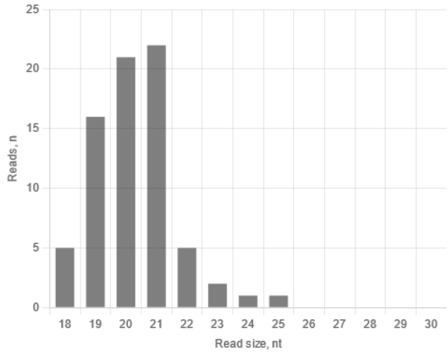

Length distribution of the reads

Dataset	Input reads	Virus	Reference	Mapped reads	Mapped reads, %	Forward/Reverse balance	Nonredundant, %	Strain specific	Strain specific, % from total mapped reads
S101	10000000	SMoV	SMoV_RNA2A	127	0.001	1.5	96.9	87	68.5

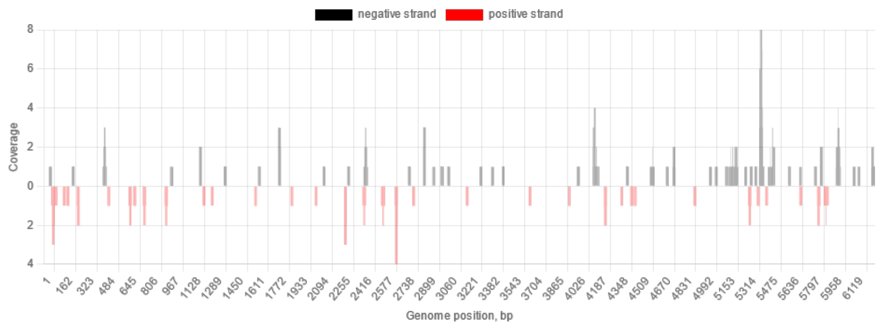

Mapping coverage of all reads matching SMoV_RNA2A (127reads)

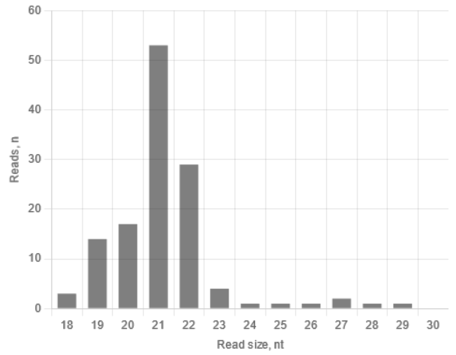

Length distribution of the reads

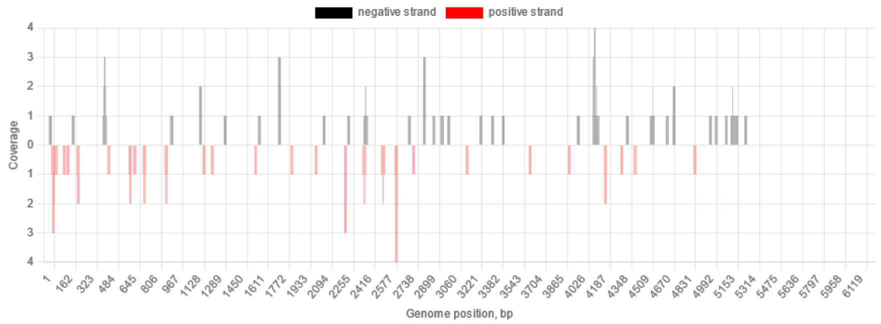

Mapping coverage of SMoV_RNA2A specific reads (87 reads)

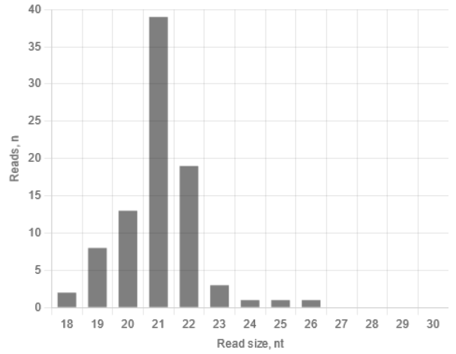

Length distribution of the reads

Dataset	Input reads	Virus	Reference	Mapped reads	Mapped reads, %	Forward/Reverse balance	Nonredundant, %	Strain specific	Strain specific, % from total mapped reads
S101	10000000	SMoV	SMoV_RNA2B	33	0.000	2.0	100.0	0	0.0

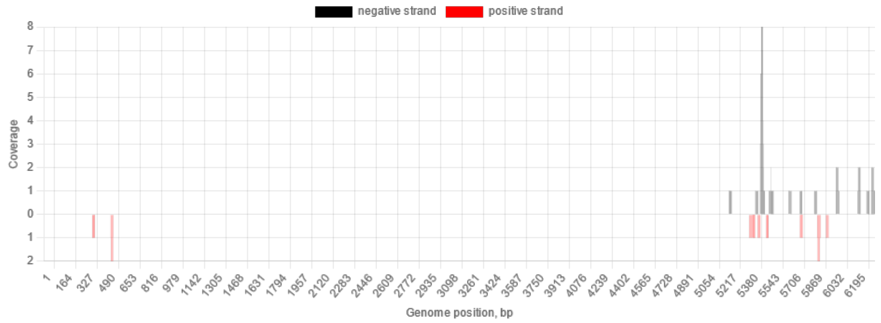

Mapping coverage of all reads matching SMoV_RNA2B (33 reads)

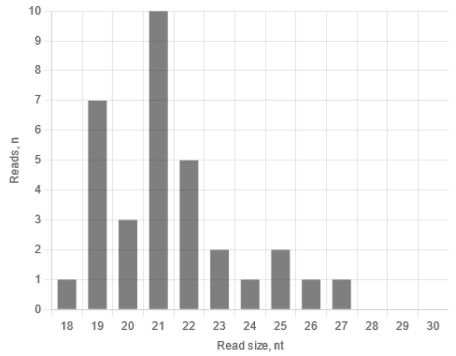

Length distribution of the reads

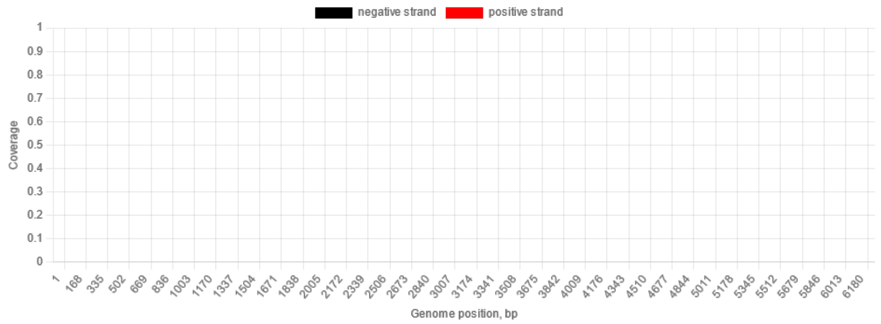

Mapping coverage of SMoV_RNA2B specific reads (0 reads)

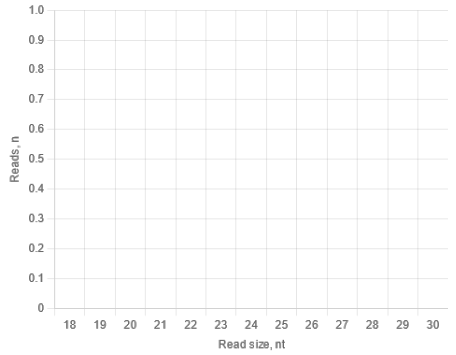

Length distribution of the reads

Dataset	Input reads	Virus	Reference	Mapped reads	Mapped reads, %	Forward/Reverse balance	Nonredundant, %	Strain specific	Strain specific, % from total mapped reads
S101	10000000	SMoV	SMoV_RNA2C	140	0.001	1.3	92.1	76	54.3

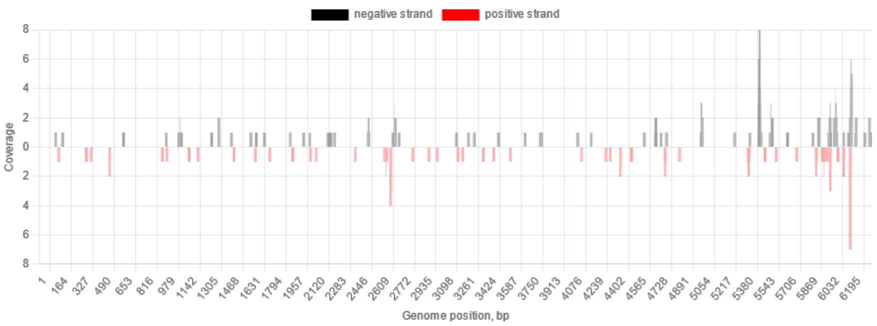

Mapping coverage of all reads matching SMoV_RNA2C (140reads)

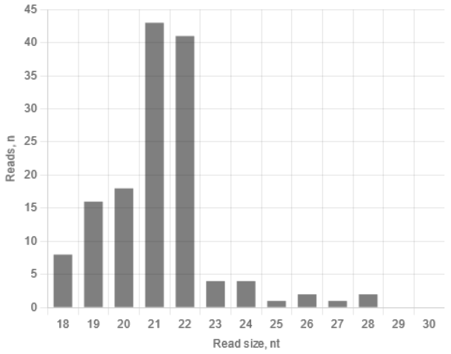

Length distribution of the reads

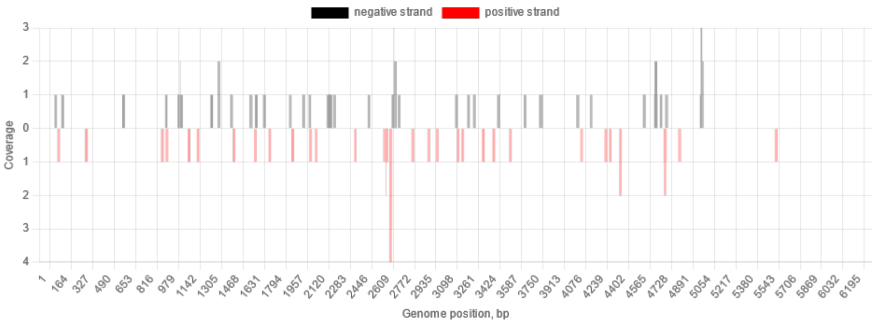

Mapping coverage of SMoV_RNA2C specific reads (76 reads)

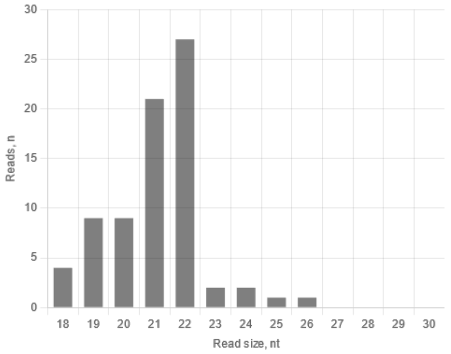

Length distribution of the reads

Dataset	Input reads	Virus	Reference	Mapped reads	Mapped reads, %	Forward/Reverse balance	Nonredundant, %	Strain specific	Strain specific, % from total mapped reads
S101	10000000	StrV1	StrV1_A	13594	0.136	0.7	4.8	11985	88.2

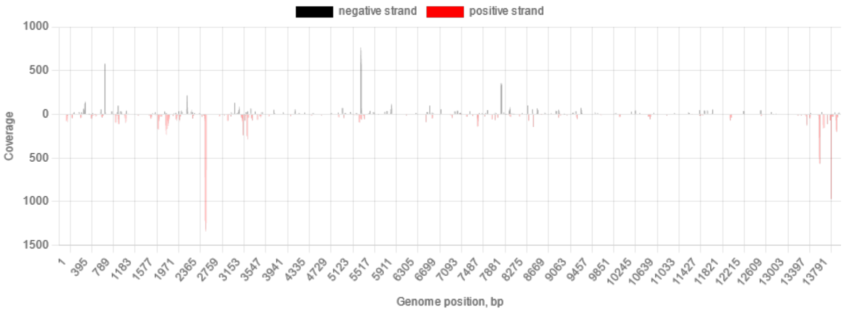

Mapping coverage of all reads matching StrV1_A (13594reads)

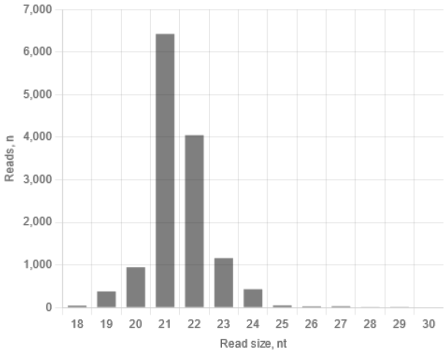

Length distribution of the reads

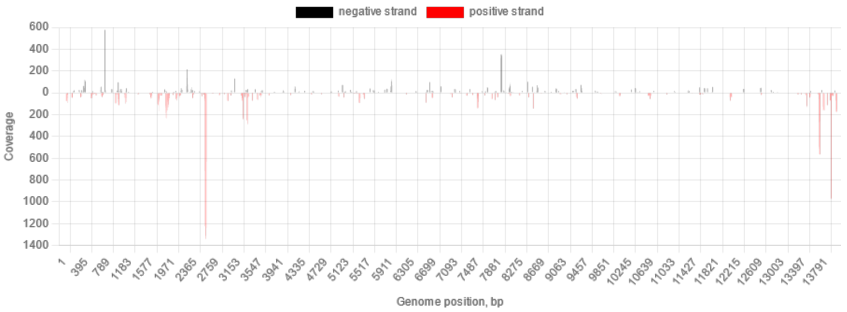

Mapping coverage of StrV1_A specific reads (11985reads)

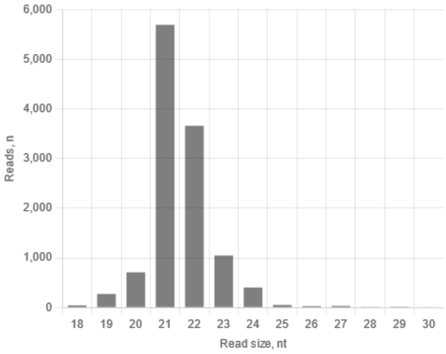

Length distribution of the reads

Dataset	Input reads	Virus	Reference	Mapped reads	Mapped reads, %	Forward/Reverse balance	Nonredundant, %	Strain specific	Strain specific, % from total mapped reads
S101	10000000	StrV1	StrV1_B	14411	0.144	1.7	4.7	13356	92.7

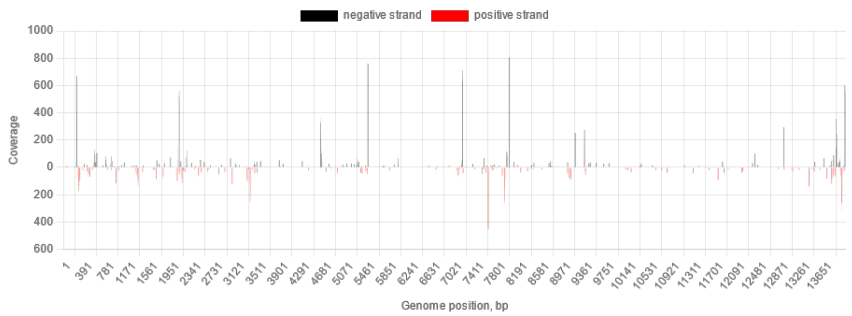

Mapping coverage of all reads matching StrV1_B (14411reads)

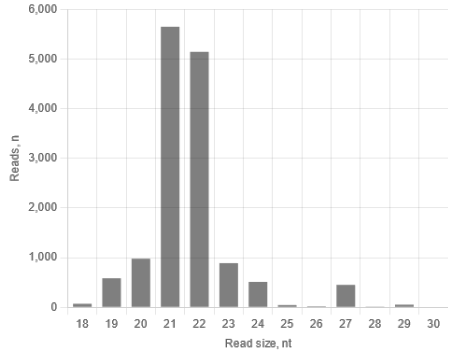

Length distribution of the reads

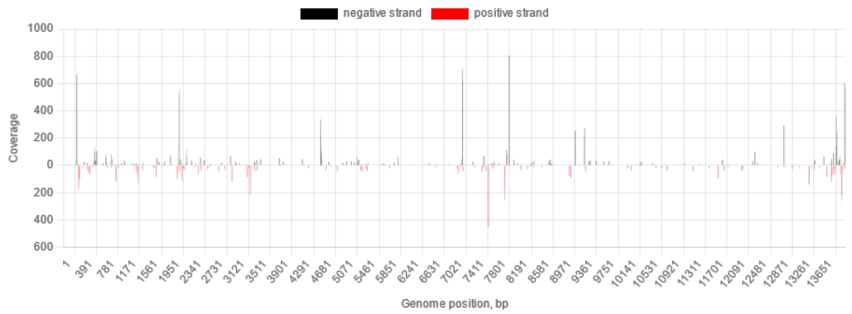

Mapping coverage of StrV1_B specific reads (13356reads)

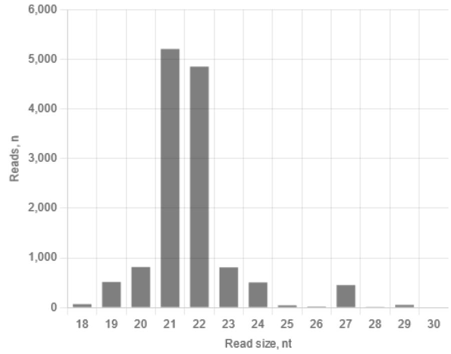

Length distribution of the reads

Dataset	Input reads	Virus	Reference	Mapped reads	Mapped reads, %	Forward/Reverse balance	Nonredundant, %	Strain specific	Strain specific, % from total mapped reads
S101	10000000	StrV1	StrV1_C	1549	0.015	3.6	5.2	60	3.9

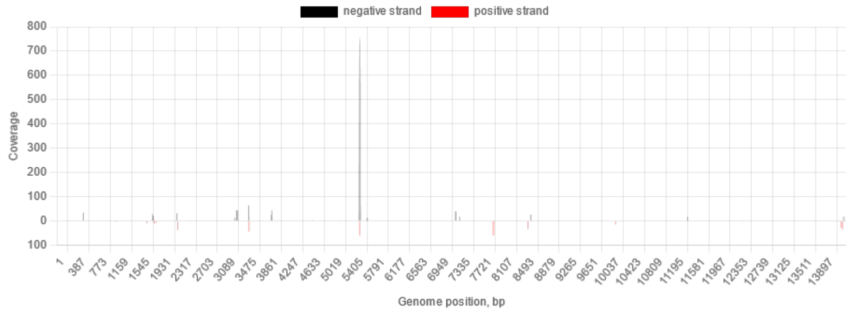

Mapping coverage of all reads matching StrV1_C (1549reads)

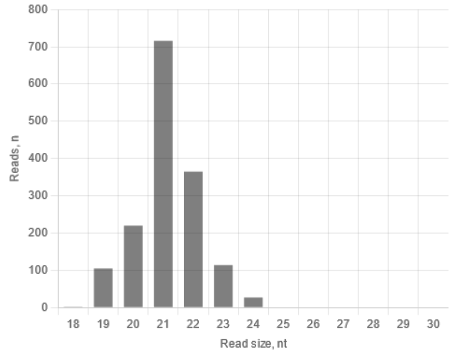

Length distribution of the reads

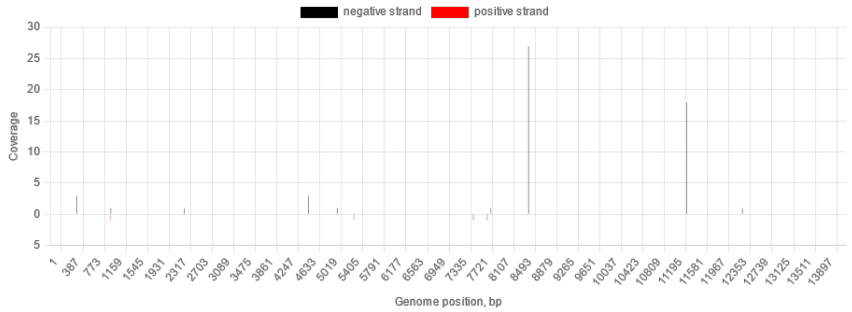

Mapping coverage of StrV1_C specific reads (60 reads)

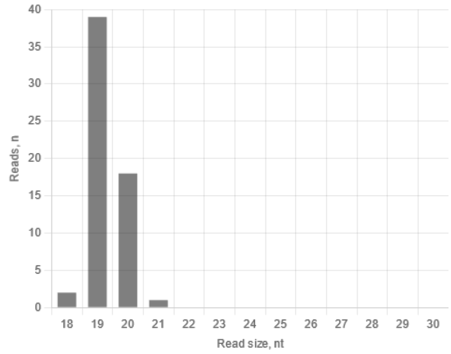

Length distribution of the reads

Mappings of the S107 reads against the viral references.

Dataset	Input reads	Virus	Reference	Mapped reads	Mapped reads, %	Forward/Reverse balance	Nonredundant, %	Strain specific	Strain specific, % from total mapped reads
S107	10000000	SCV	SCV_A	1	0.000	Infinity	100.0	11	1100.0

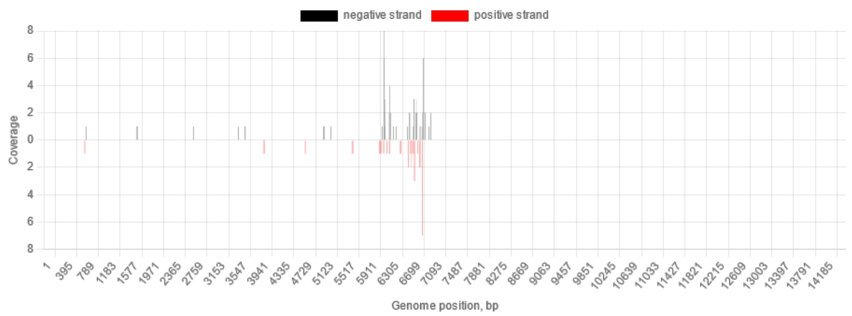

Mapping coverage of all reads matching SCV_A (73 reads)

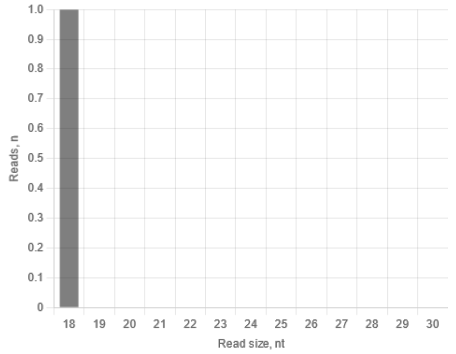

Length distribution of the reads

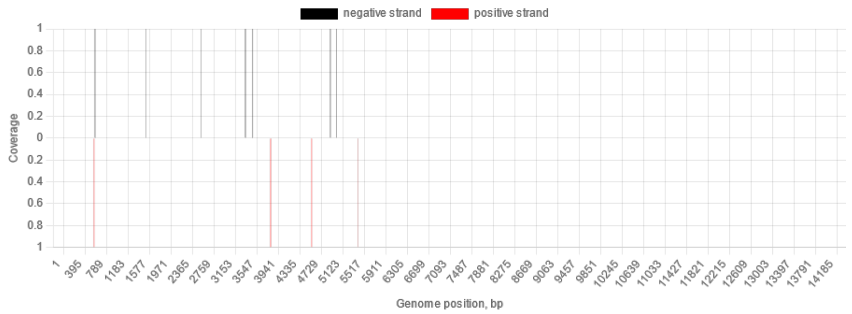

Mapping coverage of SCV_A specific reads (11 reads)

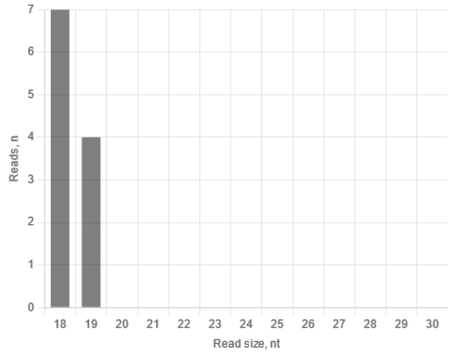

Length distribution of the reads

Dataset	Input reads	Virus	Reference	Mapped reads	Mapped reads, %	Forward/Reverse balance	Nonredundant, %	Strain specific	Strain specific, % from total mapped reads
S107	10000000	SCV	SCV_B	2	0.000	1.0	100.0	39	1950.0

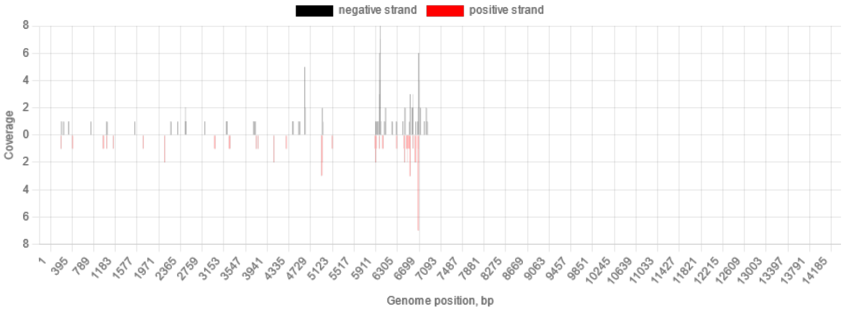

Mapping coverage of all reads matching SCV_B (103 reads)

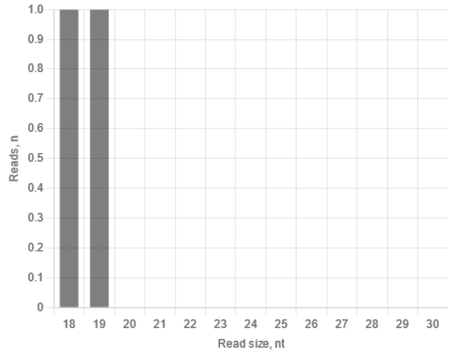

Length distribution of the reads

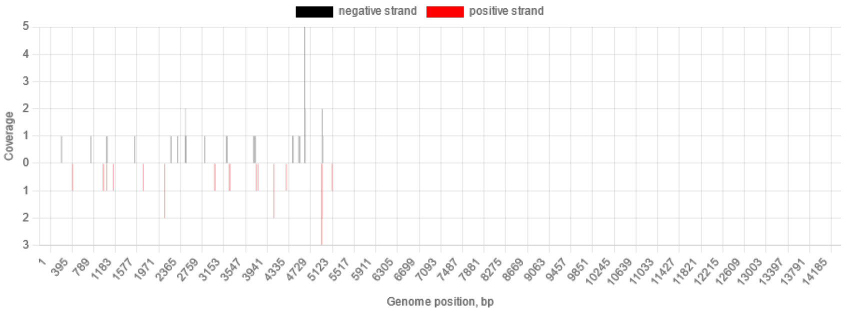

Mapping coverage of SCV_B specific reads (39 reads)

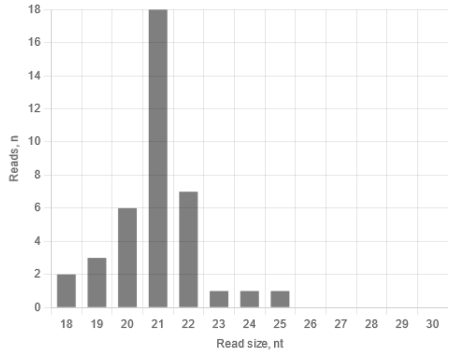

Length distribution of the reads

Dataset	Input reads	Virus	Reference	Mapped reads	Mapped reads, %	Forward/Reverse balance	Nonredundant, %	Strain specific	Strain specific, % from total mapped reads
S107	10000000.	SMoV	SMoV_RNA1A	298	0.003	1.6	82.6	73	24.5

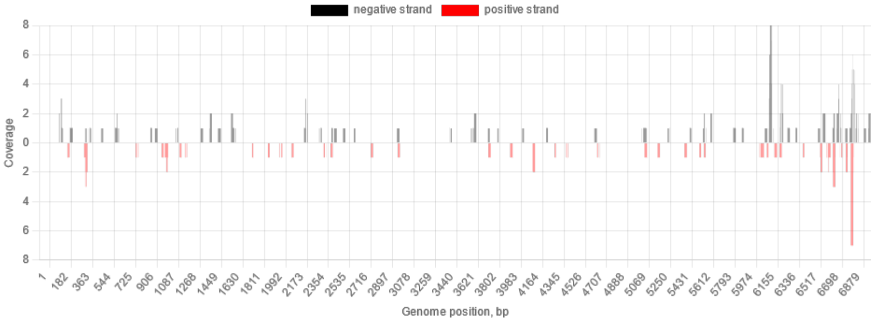

Mapping coverage of all reads matching SMoV_RNA1A (137 reads)

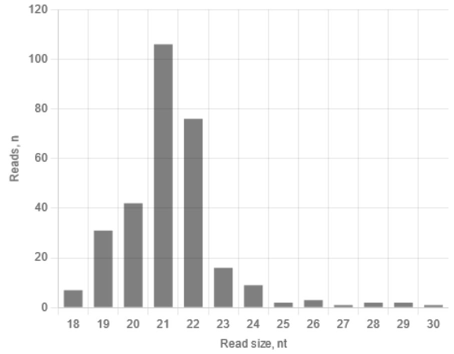

Length distribution of the reads

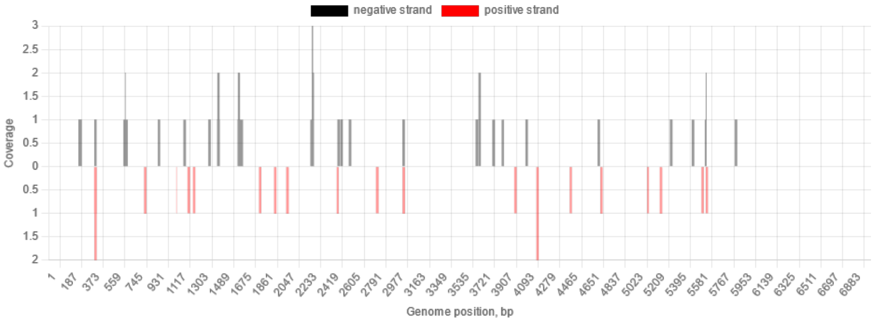

Mapping coverage of SMoV_RNA1A specific reads (73 reads)

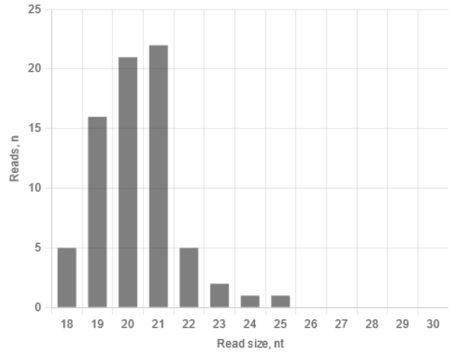

Length distribution of the reads

Dataset	Input reads	Virus	Reference	Mapped reads	Mapped reads, %	Forward/Reverse balance	Nonredundant, %	Strain specific	Strain specific, % from total mapped reads
S107	10000000.	SMoV	SMoV_RNA1B	206	0.002	1.4	79.1	87	42.2

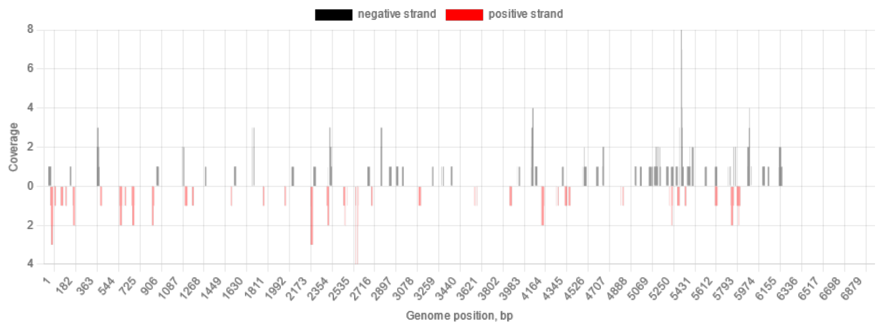

Mapping coverage of all reads matching SMoV_RNA1B (127reads)

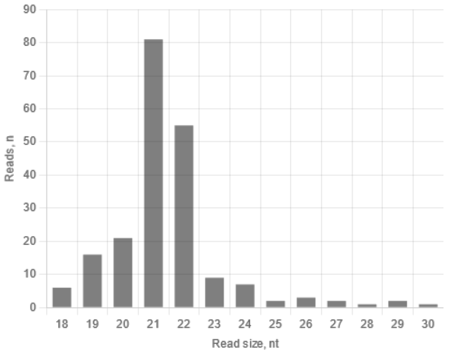

Length distribution of the reads

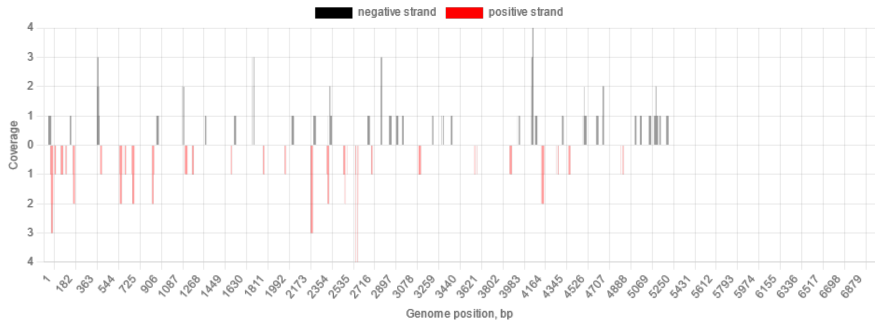

Mapping coverage of SMoV_RNA1B specific reads (87 reads)

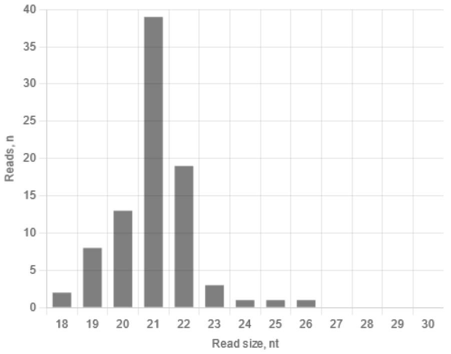

Length distribution of the reads

Dataset	Input reads	Virus	Reference	Mapped reads	Mapped reads, %	Forward/Reverse balance	Nonredundant, %	Strain specific	Strain specific, % from total mapped reads
S107	10000000.	SMoV	SMoV_RNA1C	404	0.004	1.5	83.9	0	0.0

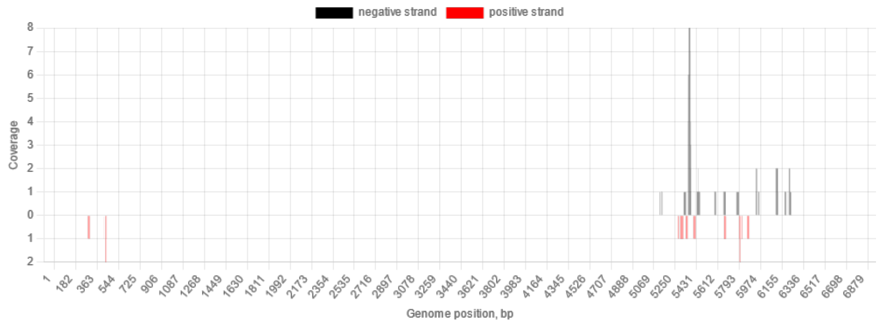

Mapping coverage of all reads matching SMoV_RNA1C (33 reads)

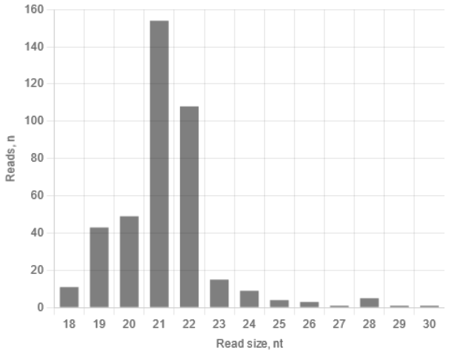

Length distribution of the reads

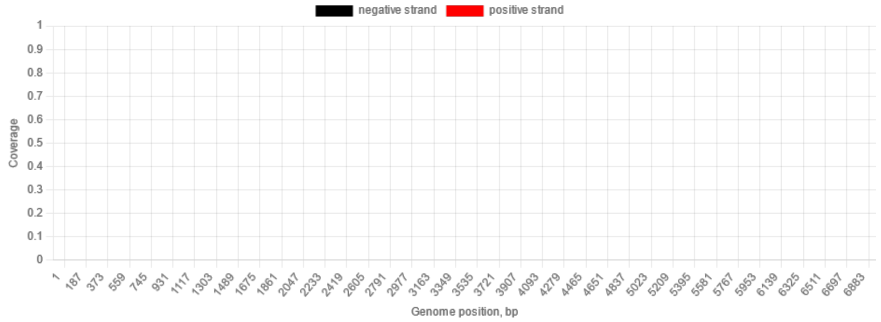

Mapping coverage of SMoV_RNA1C specific reads (0 reads)

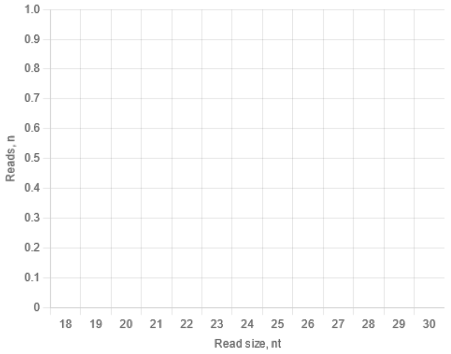

Length distribution of the reads

Dataset	Input reads	Virus	Reference	Mapped reads	Mapped reads, %	Forward/Reverse balance	Nonredundant, %	Strain specific	Strain specific, % from total mapped reads
S107	10000000.	SMoV	SMoV_RNA2A	319	0.003	1.3	85.0	76	23.8

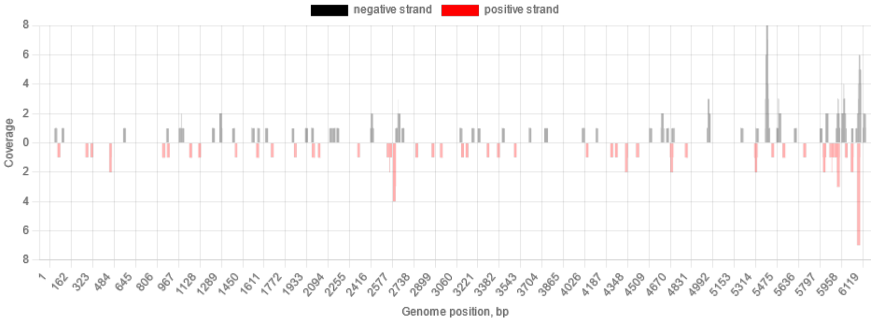

Mapping coverage of all reads matching SMoV_RNA2A (140reads)

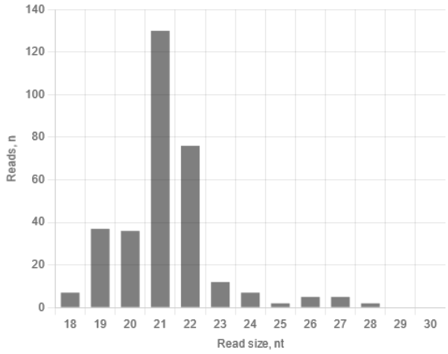

Length distribution of the reads

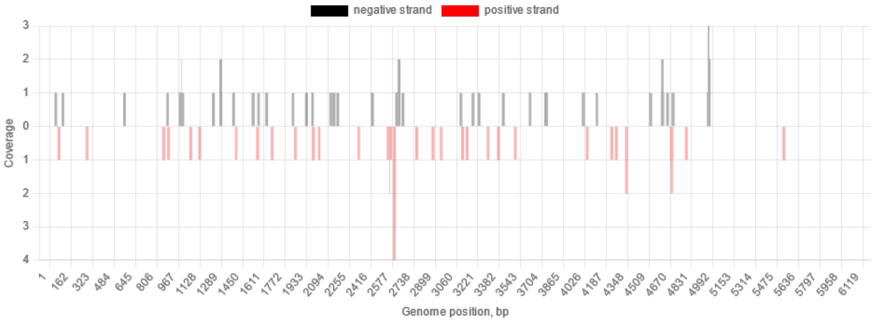

Mapping coverage of SMoV_RNA2A specific reads (76 reads)

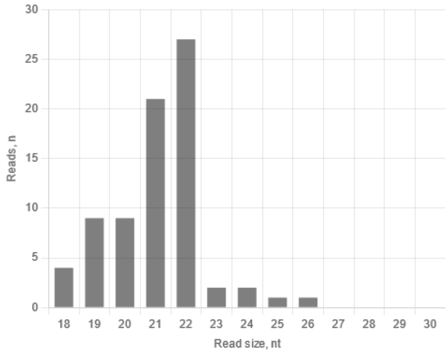

Length distribution of the reads

Dataset	Input reads	Virus	Reference	Mapped reads	Mapped reads, %	Forward/Reverse balance	Nonredundant, %	Strain specific	Strain specific, % from total mapped reads
S107	10000000.	SMoV	SMoV_RNA2B	124	0.001	1.8	89.5	11985	9665.3

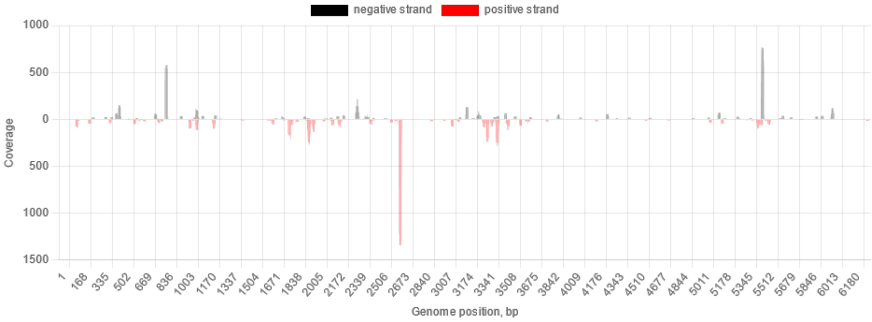

Mapping coverage of all reads matching SMoV_RNA2B (13594reads)

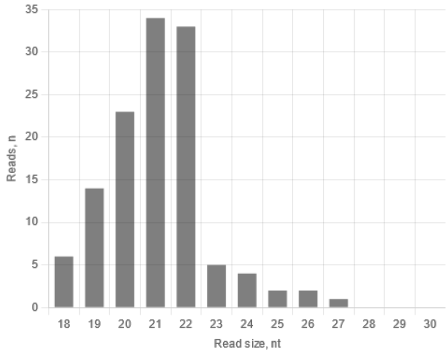

Length distribution of the reads

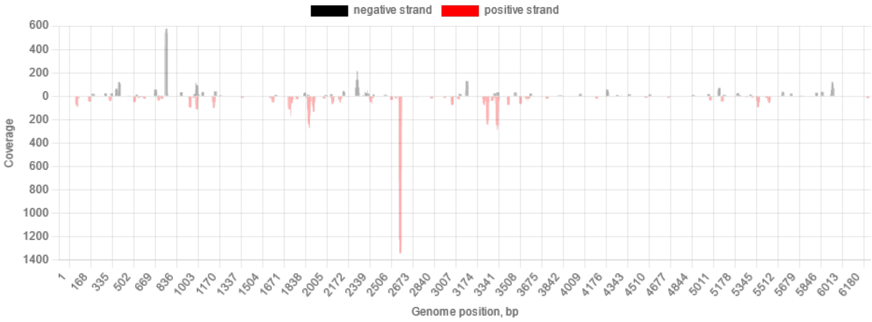

Mapping coverage of SMoV_RNA2B specific reads (11985reads)

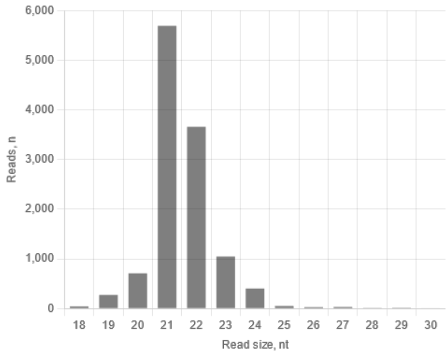

Length distribution of the reads

Dataset	Input reads	Virus	Reference	Mapped reads	Mapped reads, %	Forward/Reverse balance	Nonredundant, %	Strain specific	Strain specific, % from total mapped reads
S107	10000000.	SMoV	SMoV_RNA2C	373	0.004	1.6	81.0	13356	3580.7

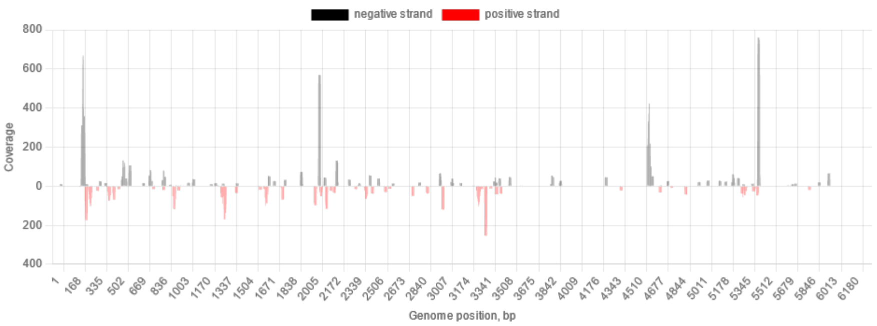

Mapping coverage of all reads matching SMoV_RNA2C (14411reads)

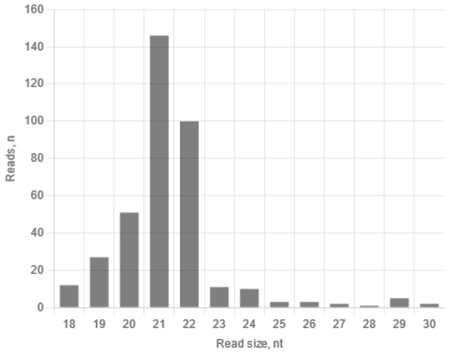

Length distribution of the reads

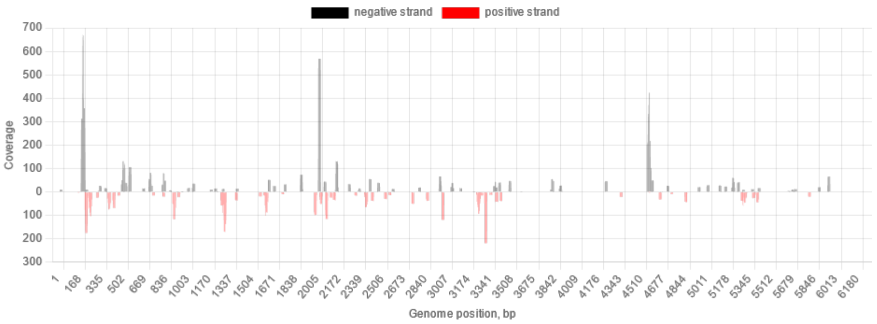

Mapping coverage of SMoV_RNA2C specific reads (13356reads)

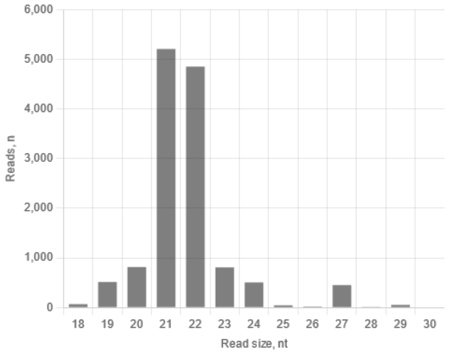

Length distribution of the reads

Dataset	Input reads	Virus	Reference	Mapped reads	Mapped reads, %	Forward/Reverse balance	Nonredundant, %	Strain specific	Strain specific, % from total mapped reads
S107	10000000	StrV1	StrV1_A	256	0.003	1.1	35.9	60	23.4

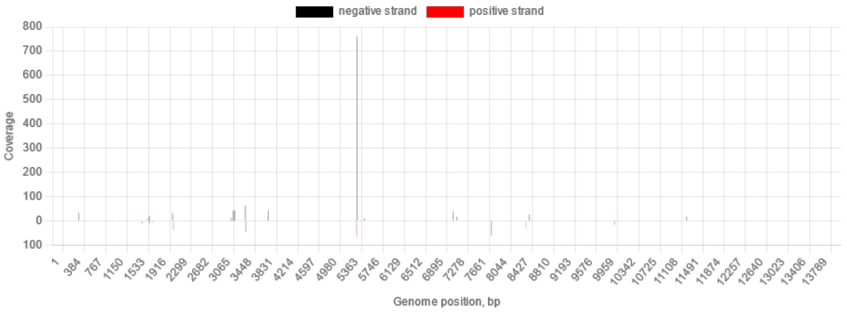

Mapping coverage of all reads matching StrV1_A (1549reads)

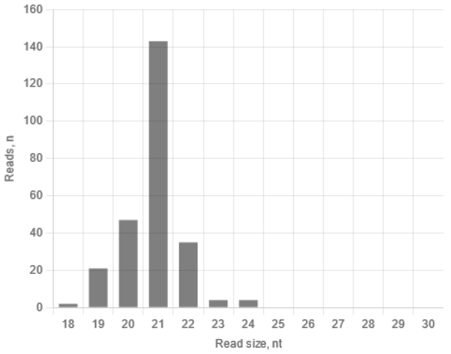

Length distribution of the reads

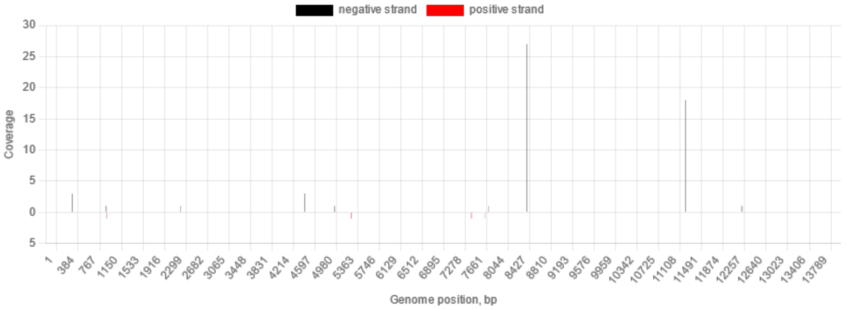

Mapping coverage of StrV1_A specific reads (60 reads)

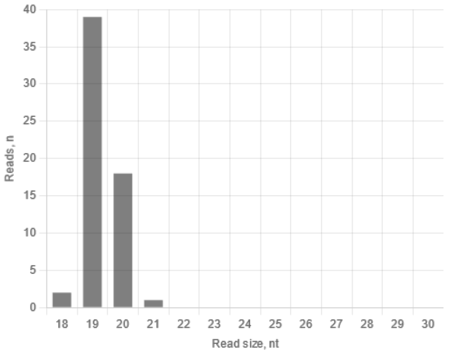

Length distribution of the reads

Dataset	Input reads	Virus	Reference	Mapped reads	Mapped reads, %	Forward/Reverse balance	Nonredundant, %	Strain specific	Strain specific, % from total mapped reads
S107	10000000	StrV1	StrV1_B	13321	0.133	2.2	35.7	1	0.0

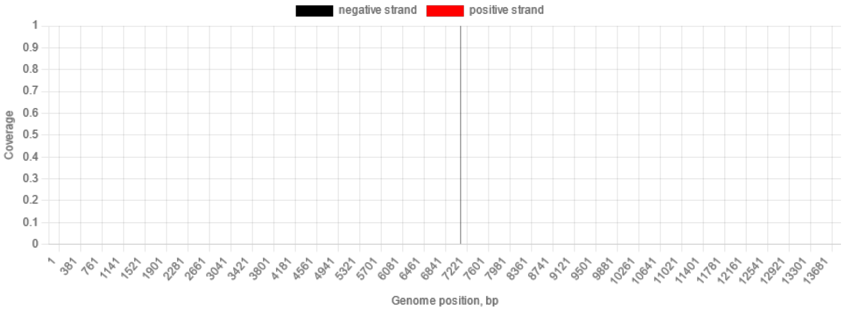

Mapping coverage of all reads matching StrV1_B (1 reads)

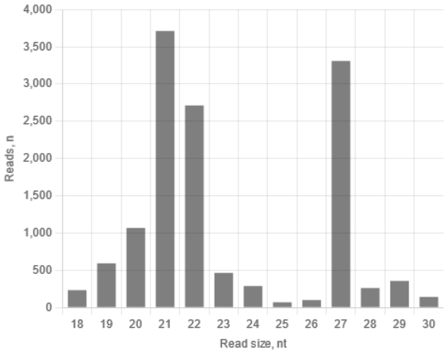

Length distribution of the reads

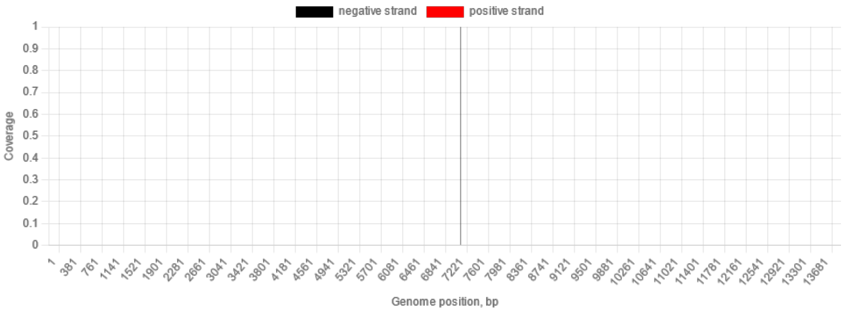

Mapping coverage of StrV1_B specific reads (1 reads)

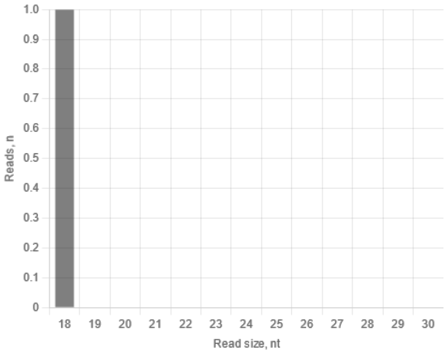

Length distribution of the reads

Dataset	Input reads	Virus	Reference	Mapped reads	Mapped reads, %	Forward/Reverse balance	Nonredundant, %	Strain specific	Strain specific, % from total mapped reads
S107	10000000	StrV1	StrV1_C	178	0.002	1.3	42.1	2	1.1

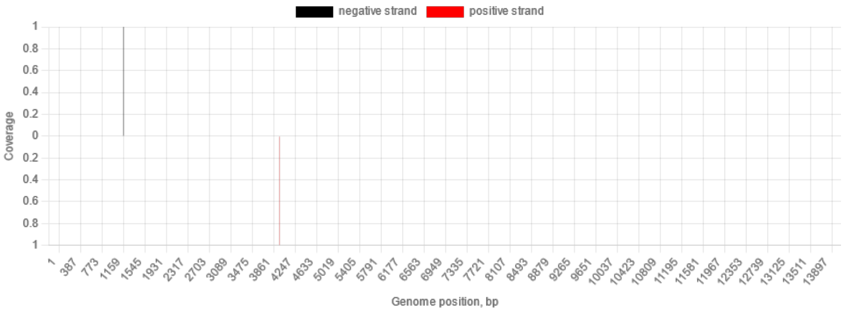

Mapping coverage of all reads matching StrV1_C (2 reads)

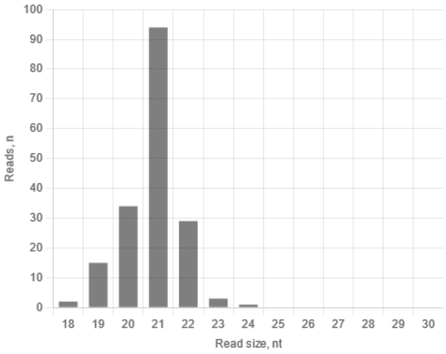

Length distribution of the reads

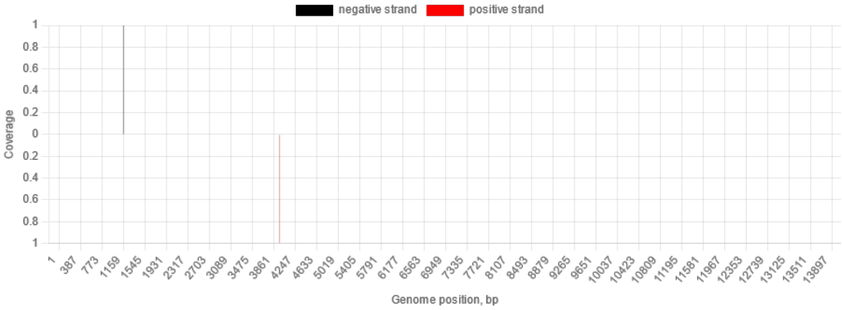

Mapping coverage of StrV1_C specific reads (2 reads)

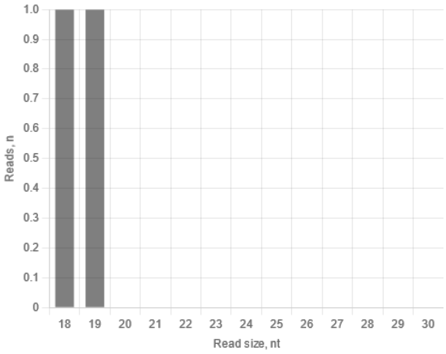

Length distribution of the reads

Mappings of the S108 reads against the viral references.

Dataset	Input reads	Virus	Reference	Mapped reads	Mapped reads, %	Forward/Reverse balance	Nonredundant, %	Strain specific	Strain specific, % from total mapped reads
S108	10000000	SCV	SCV_A	1	0.000	Infinity	100.0	11	1100.0

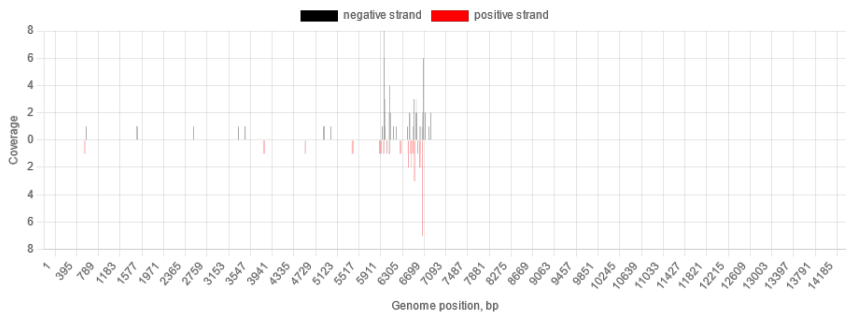

Mapping coverage of all reads matching SCV_A (73 reads)

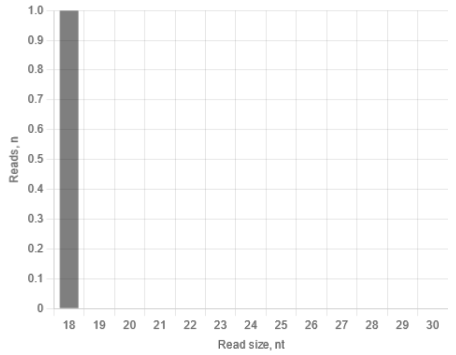

Length distribution of the reads

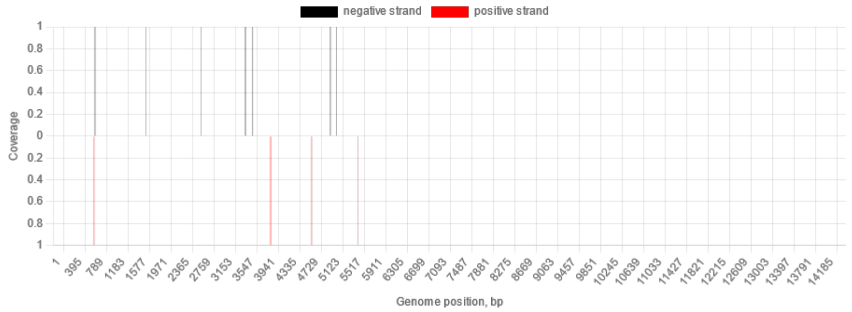

Mapping coverage of SCV_A specific reads (11 reads)

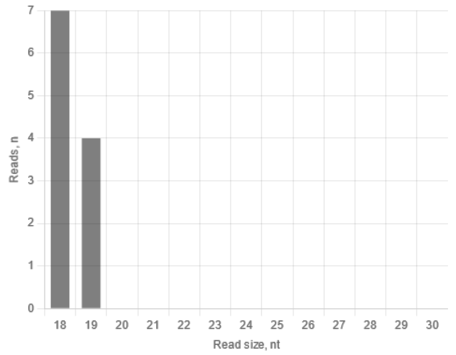

Length distribution of the reads

Dataset	Input reads	Virus	Reference	Mapped reads	Mapped reads, %	Forward/Reverse balance	Nonredundant, %	Strain specific	Strain specific, % from total mapped reads
S108	10000000	SCV	SCV_B	3	0.000	Infinity	100.0	39	1300.0

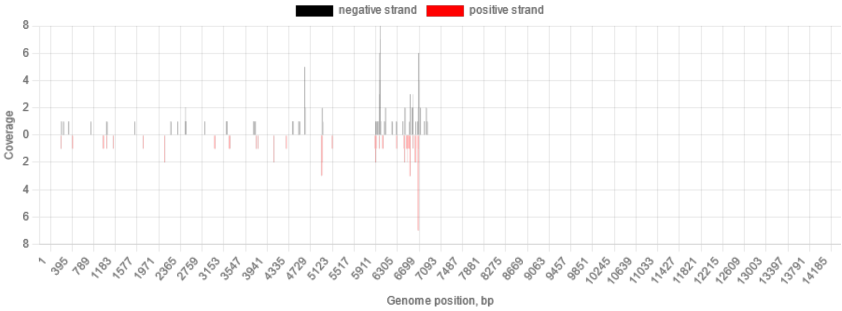

Mapping coverage of all reads matching SCV_B (103 reads)

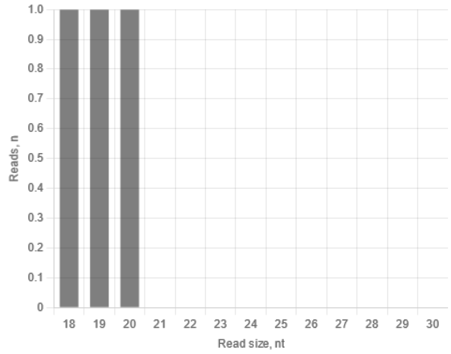

Length distribution of the reads

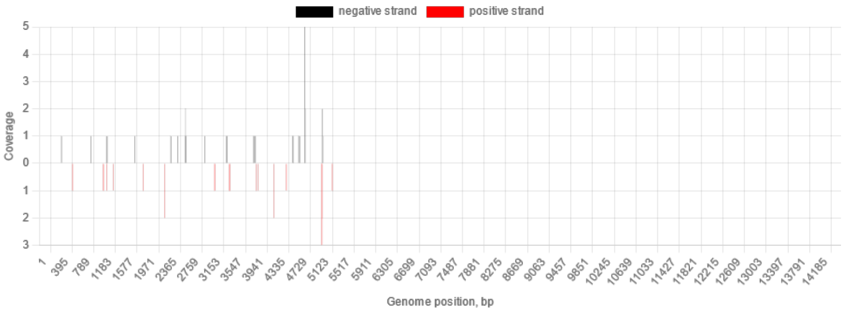

Mapping coverage of SCV_B specific reads (39 reads)

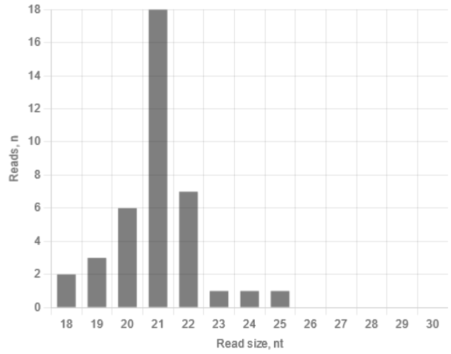

Length distribution of the reads

Dataset	Input reads	Virus	Reference	Mapped reads	Mapped reads, %	Forward/Reverse balance	Nonredundant, %	Strain specific	Strain specific, % from total mapped reads
S108	10000000	SMoV	SMoV_RNA1A	199	0.002	1.3	87.9	73	36.7

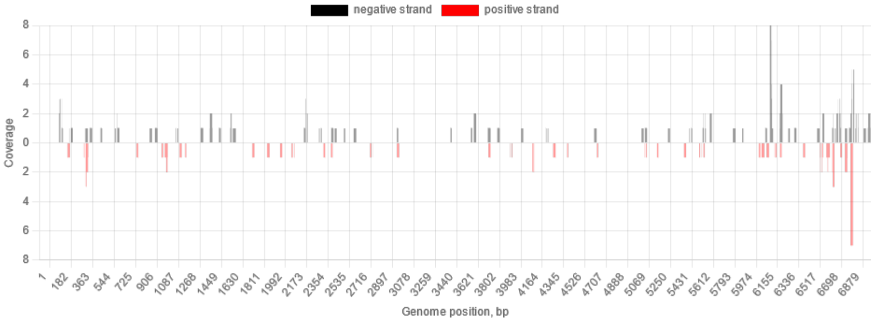

Mapping coverage of all reads matching SMoV_RNA1A (137 reads)

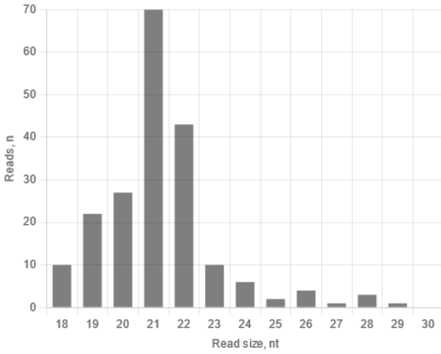

Length distribution of the reads

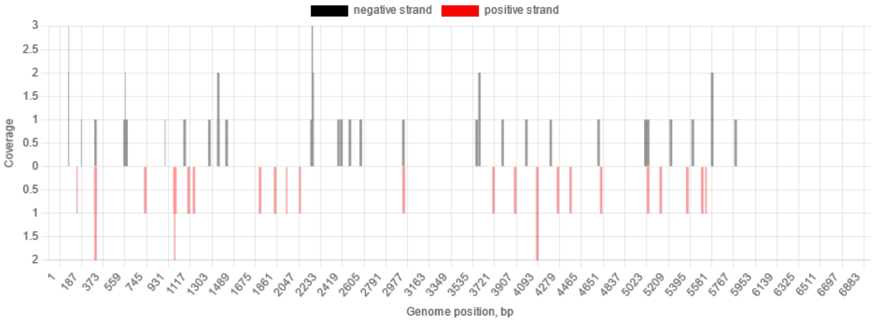

Mapping coverage of SMoV_RNA1A specific reads (73 reads)

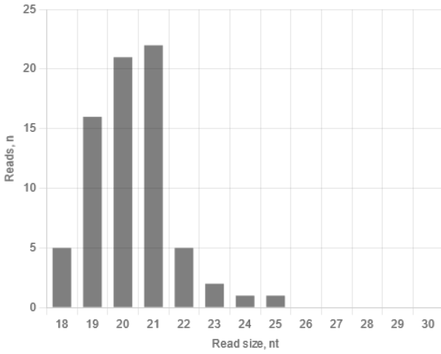

Length distribution of the reads

Dataset	Input reads	Virus	Reference	Mapped reads	Mapped reads, %	Forward/Reverse balance	Nonredundant, %	Strain specific	Strain specific, % from total mapped reads
S108	10000000	SMoV	SMoV_RNA1B	133	0.001	1.8	87.2	87	65.4

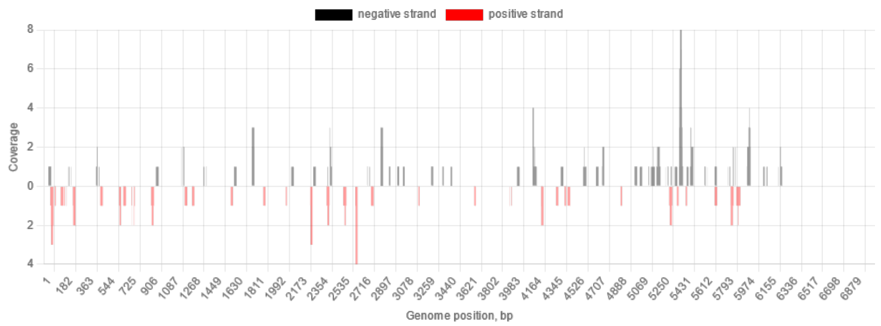

Mapping coverage of all reads matching SMoV_RNA1B (127reads)

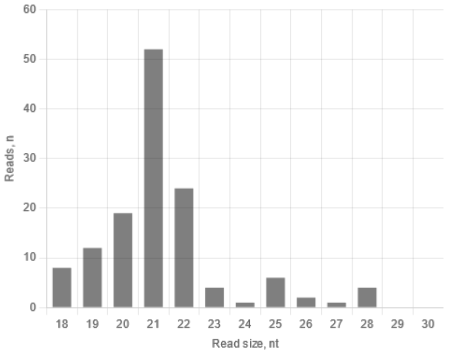

Length distribution of the reads

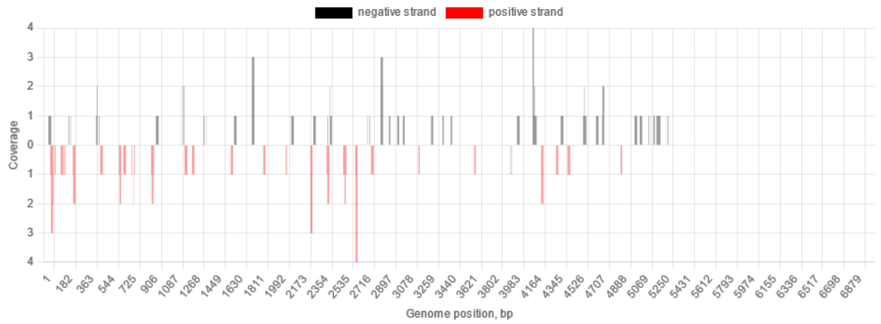

Mapping coverage of SMoV_RNA1B specific reads (87 reads)

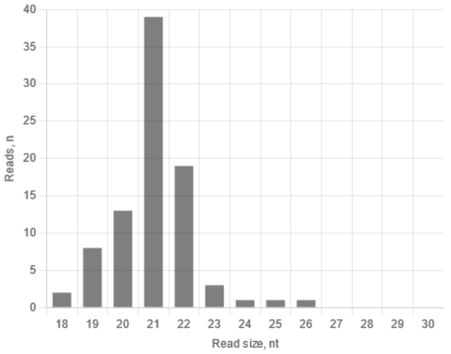

Length distribution of the reads

Dataset	Input reads	Virus	Reference	Mapped reads	Mapped reads, %	Forward/Reverse balance	Nonredundant, %	Strain specific	Strain specific, % from total mapped reads
S108	10000000	SMoV	SMoV_RNA1C	178	0.002	1.6	89.3	0	0.0

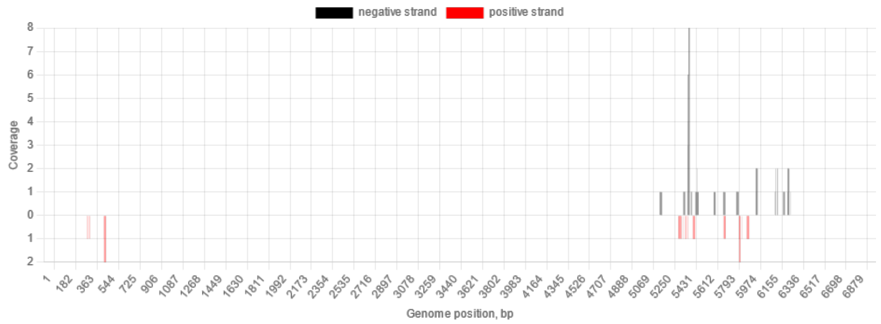

Mapping coverage of all reads matching SMoV_RNA1C (33 reads)

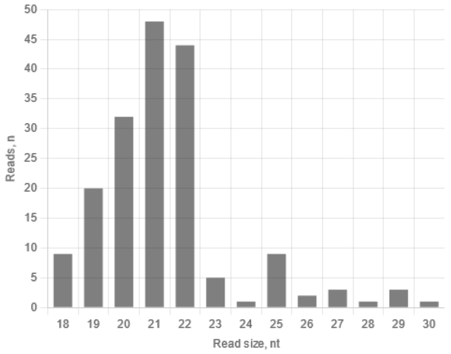

Length distribution of the reads

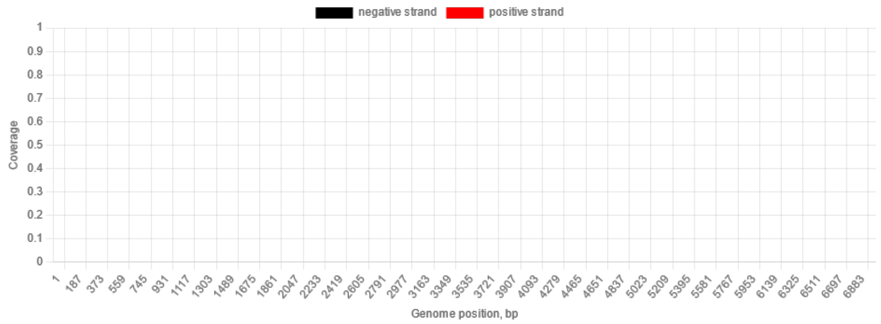

Mapping coverage of SMoV_RNA1C specific reads (0 reads)

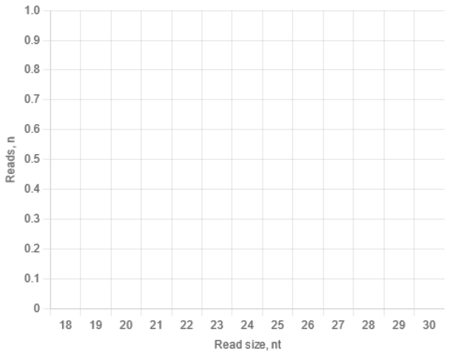

Length distribution of the reads

Dataset	Input reads	Virus	Reference	Mapped reads	Mapped reads, %	Forward/Reverse balance	Nonredundant, %	Strain specific	Strain specific, % from total mapped reads
S108	10000000	SMoV	SMoV_RNA2A	154	0.002	2.3	90.3	76	49.4

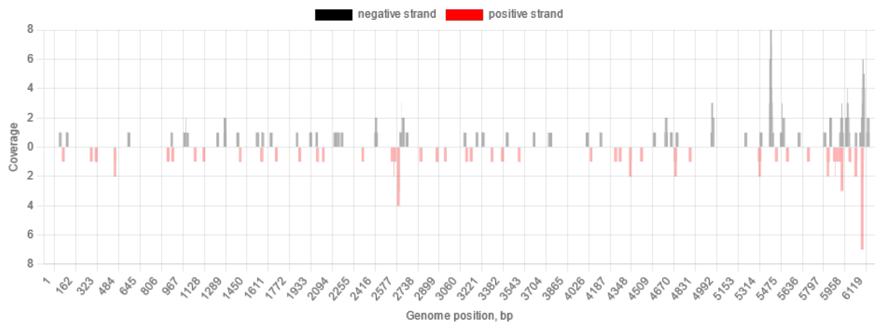

Mapping coverage of all reads matching SMoV_RNA2A (140reads)

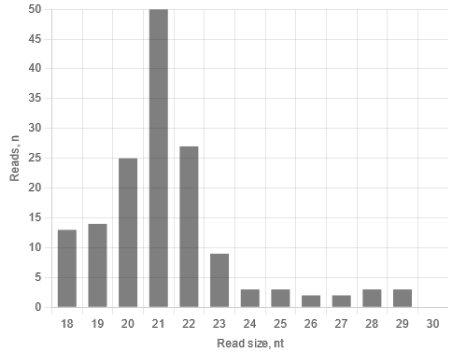

Length distribution of the reads

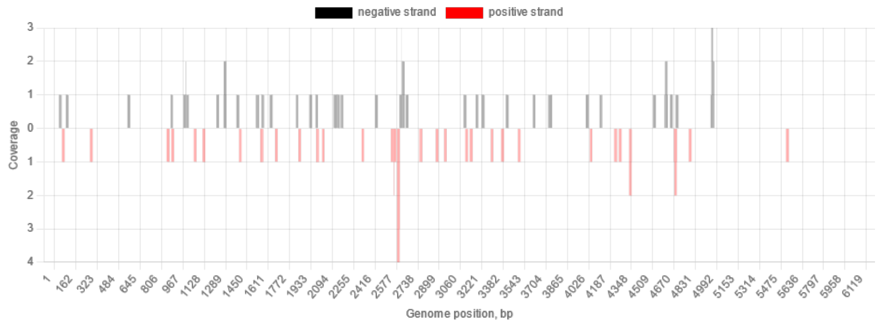

Mapping coverage of SMoV_RNA2A specific reads (76 reads)

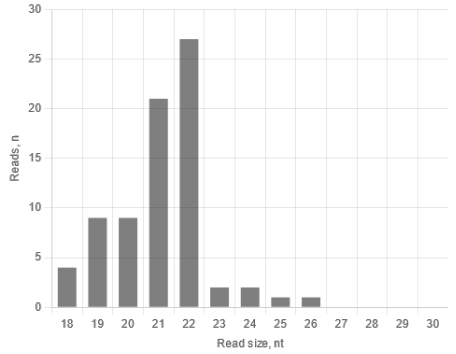

Length distribution of the reads

Dataset	Input reads	Virus	Reference	Mapped reads	Mapped reads, %	Forward/Reverse balance	Nonredundant, %	Strain specific	Strain specific, % from total mapped reads
S108	10000000	SMoV	SMoV_RNA2B	85	0.001	1.5	94.1	11985	14100.0

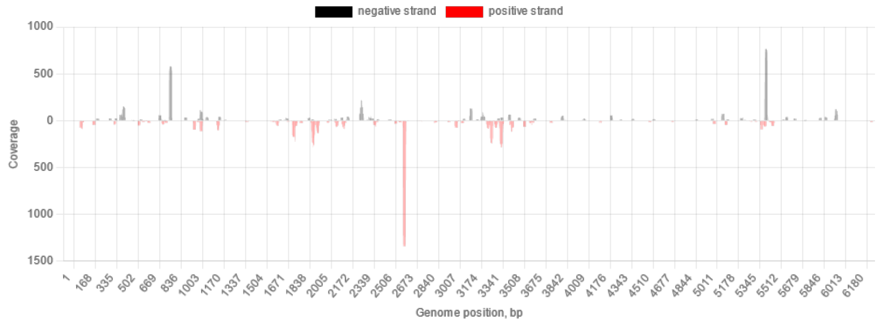

Mapping coverage of all reads matching SMoV_RNA2B (13594reads)

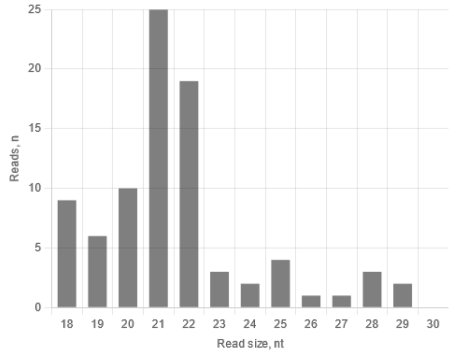

Length distribution of the reads

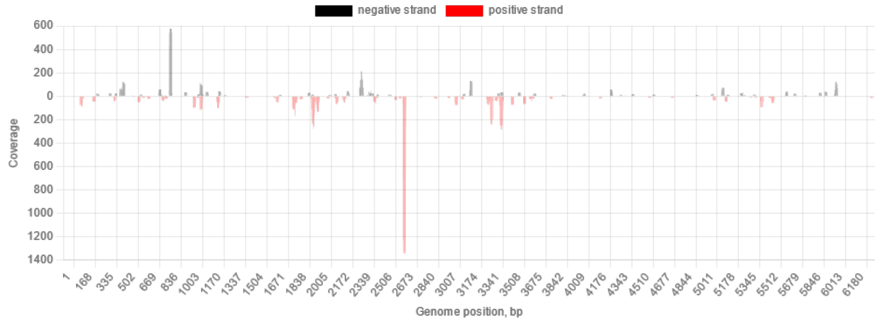

Mapping coverage of SMoV_RNA2B specific reads (11985reads)

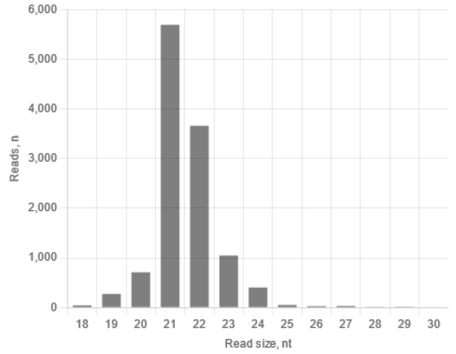

Length distribution of the reads

Dataset	Input reads	Virus	Reference	Mapped reads	Mapped reads, %	Forward/Reverse balance	Nonredundant, %	Strain specific	Strain specific, % from total mapped reads
S108	10000000	SMoV	SMoV_RNA2C	239	0.002	1.5	86.6	13356	5588.3

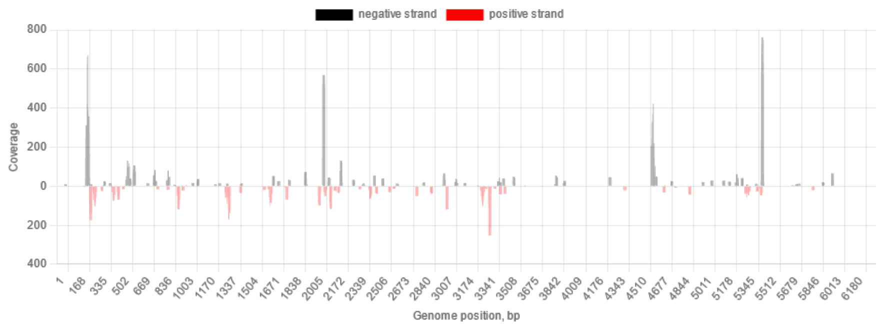

Mapping coverage of all reads matching SMoV_RNA2C (14411reads)

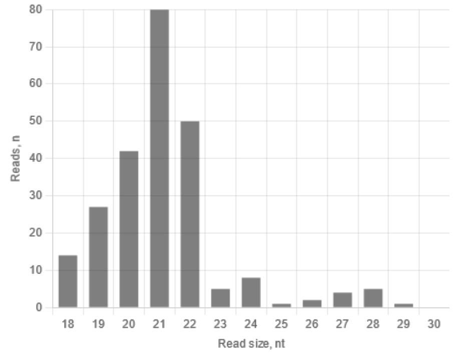

Length distribution of the reads

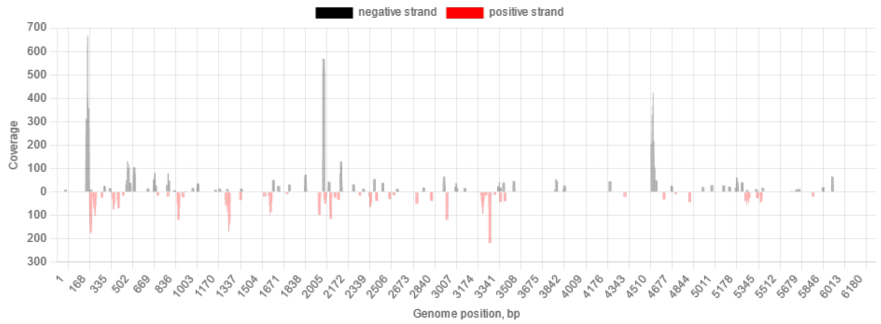

Mapping coverage of SMoV_RNA2C specific reads (13356reads)

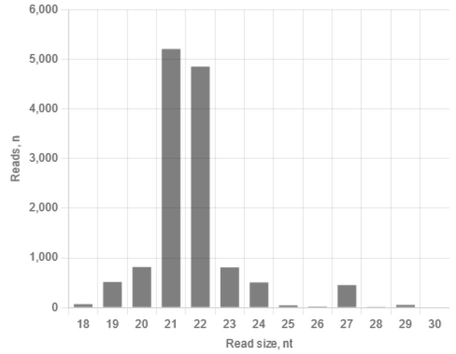

Length distribution of the reads

Dataset	Input reads	Virus	Reference	Mapped reads	Mapped reads, %	Forward/Reverse balance	Nonredundant, %	Strain specific	Strain specific, % from total mapped reads
S108	10000000	StrV1	StrV1_A	201	0.002	1.0	50.2	60	29.9

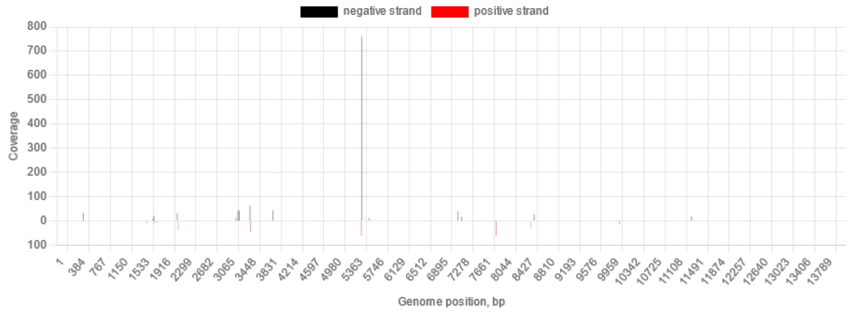

Mapping coverage of all reads matching StrV1_A (1549reads)

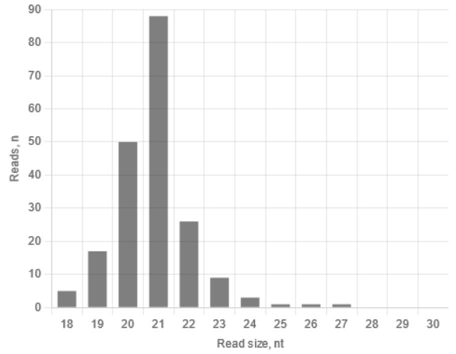

Length distribution of the reads

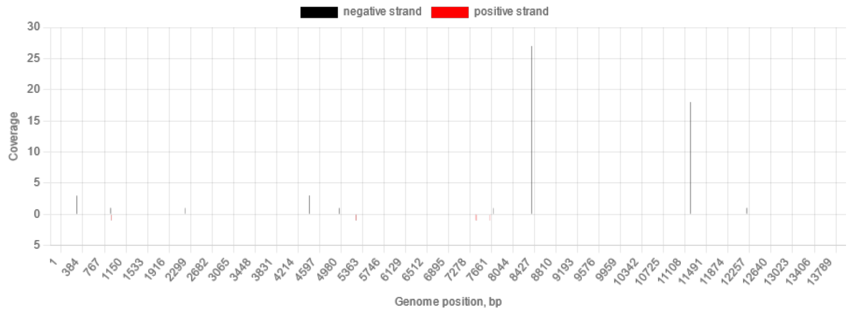

Mapping coverage of StrV1_A specific reads (60 reads)

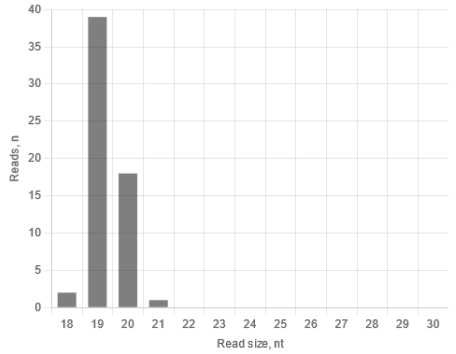

Length distribution of the reads

Dataset	Input reads	Virus	Reference	Mapped reads	Mapped reads, %	Forward/Reverse balance	Nonredundant, %	Strain specific	Strain specific, % from total mapped reads
S108	10000000	StrV1	StrV1_B	9775	0.098	2.3	38.1	1	0.0

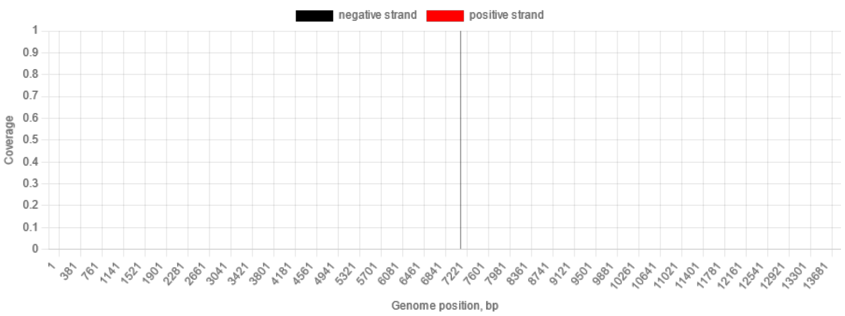

Mapping coverage of all reads matching StrV1_B (1 reads)

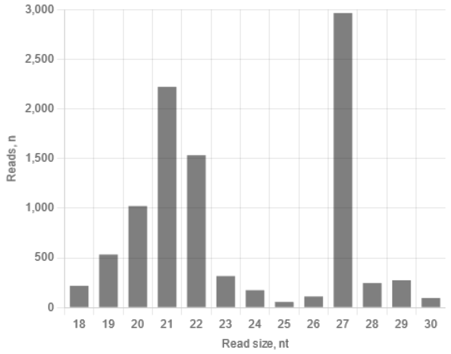

Length distribution of the reads

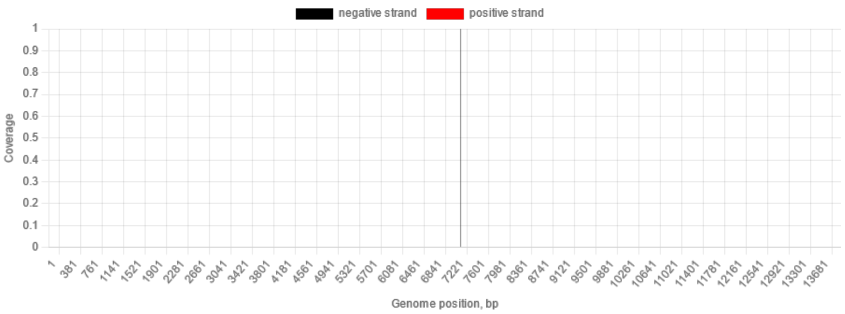

Mapping coverage of StrV1_B specific reads (1 reads)

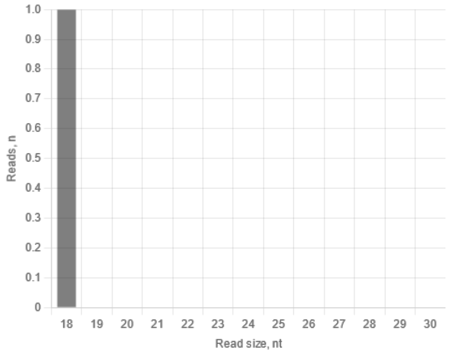

Length distribution of the reads

Dataset	Input reads	Virus	Reference	Mapped reads	Mapped reads, %	Forward/Reverse balance	Nonredundant, %	Strain specific	Strain specific, % from total mapped reads
S108	10000000	StrV1	StrV1_C	112	0.001	0.8	57.1	2	1.8

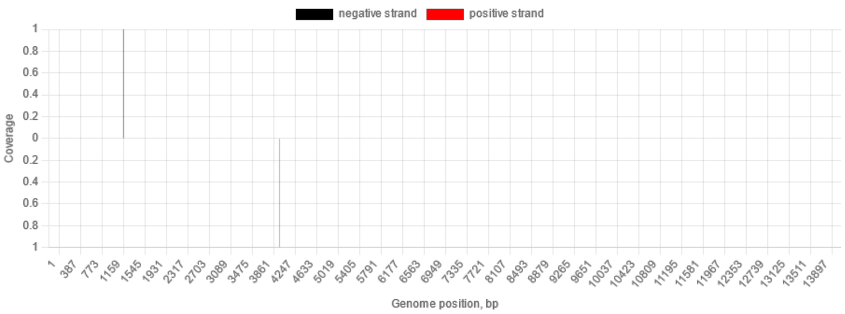

Mapping coverage of all reads matching StrV1_C (2 reads)

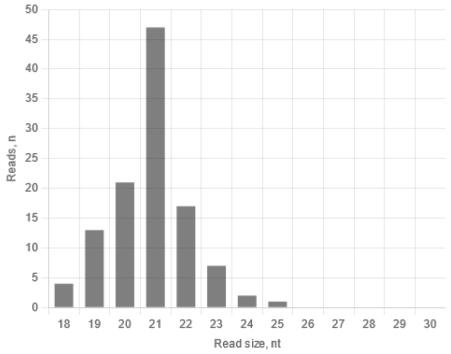

Length distribution of the reads

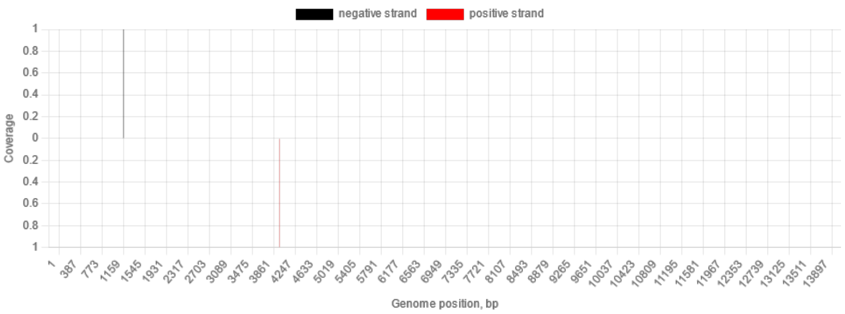

Mapping coverage of StrV1_C specific reads (2 reads)

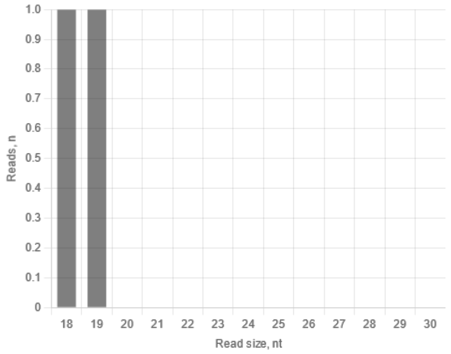

Length distribution of the reads

Mappings of the S95 reads against the viral references.

Dataset	Input reads	Virus	Reference	Mapped reads	Mapped reads, %	Forward/Reverse balance	Nonredundant, %	Strain specific	Strain specific, % from total mapped reads
S95	10000000	StrV1	StrV1_A	19940	0.199	1.0	46.2	11	0.1

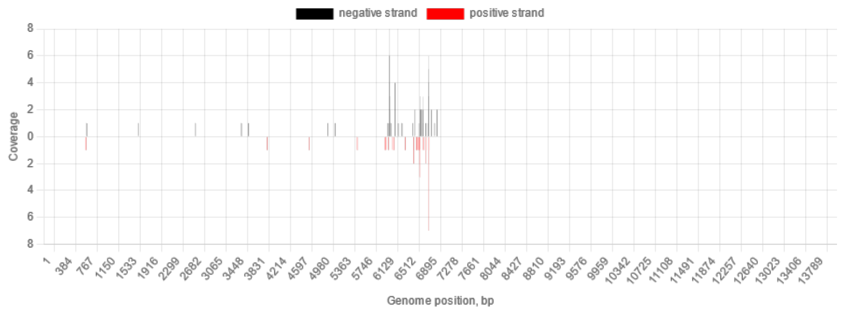

Mapping coverage of all reads matching StrV1_A (73 reads)

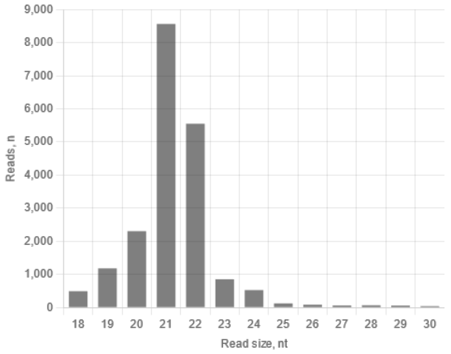

Length distribution of the reads

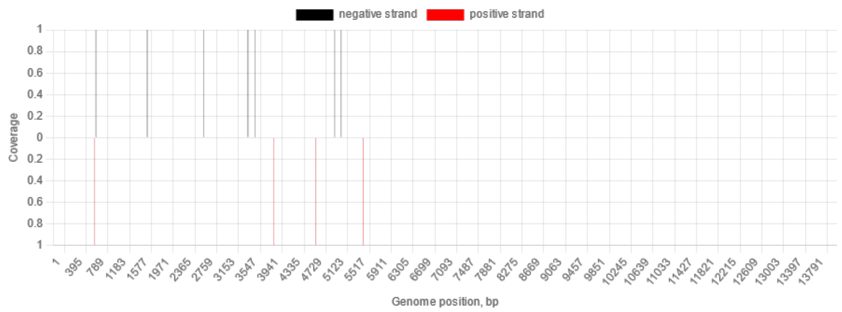

Mapping coverage of StrV1_A specific reads (11 reads)

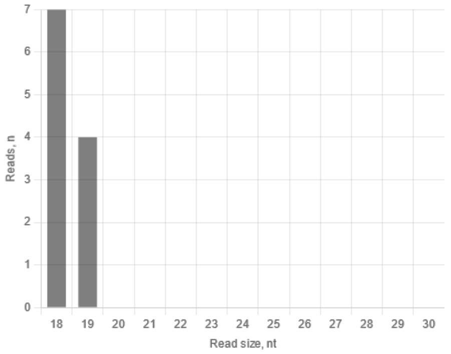

Length distribution of the reads

Dataset	Input reads	Virus	Reference	Mapped reads	Mapped reads, %	Forward/Reverse balance	Nonredundant, %	Strain specific	Strain specific, % from total mapped reads
S95	10000000	StrV1	StrV1_B	358	0.004	1.2	48.3	39	10.9

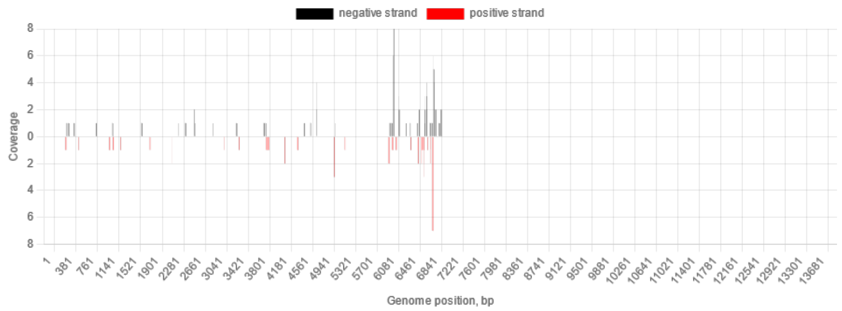

Mapping coverage of all reads matching StrV1_B (103 reads)

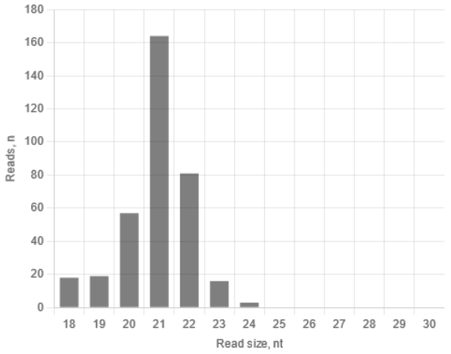

Length distribution of the reads

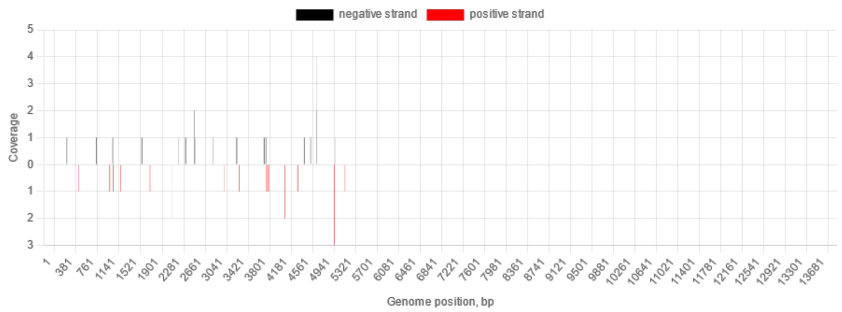

Mapping coverage of StrV1_B specific reads (39 reads)

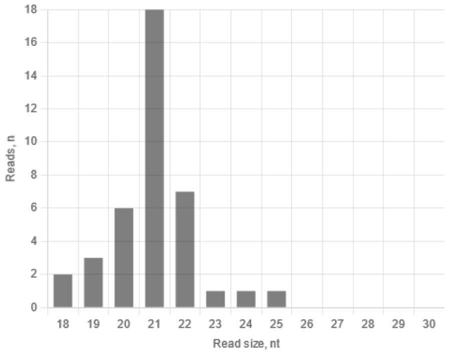

Length distribution of the reads

Dataset	Input reads	Virus	Reference	Mapped reads	Mapped reads, %	Forward/Reverse balance	Nonredundant, %	Strain specific	Strain specific, % from total mapped reads
S95	10000000	StrV1	StrV1_C	2174	0.022	1.3	41.7	73	3.4

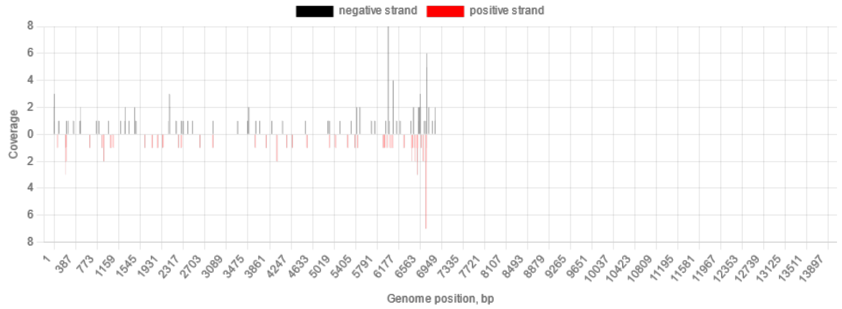

Mapping coverage of all reads matching StrV1_C (137 reads)

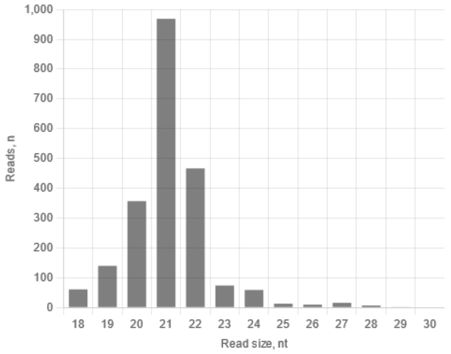

Length distribution of the reads

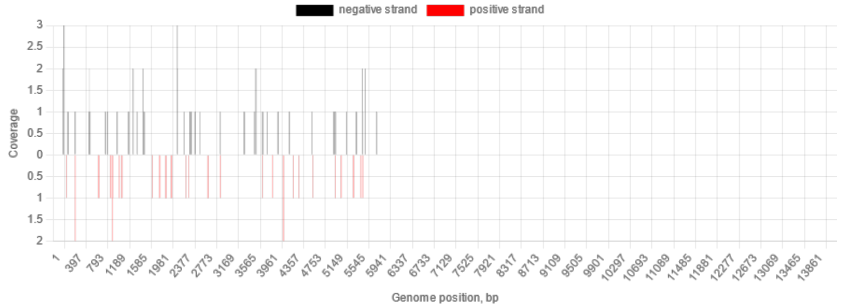

Mapping coverage of StrV1_C specific reads (73 reads)

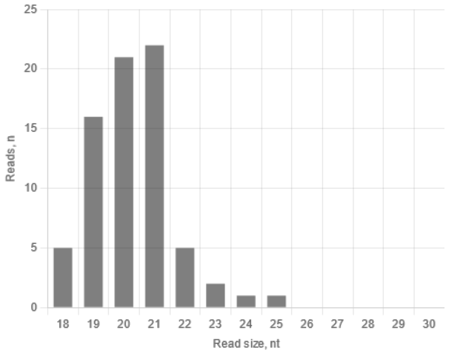

Length distribution of the reads

Mappings of the S98 reads against the viral references.

Dataset	Input reads	Virus	Reference	Mapped reads	Mapped reads, %	Forward/Reverse balance	Nonredundant, %	Strain specific	Strain specific, % from total mapped reads
S98	9901238	SMoV	SMoV_RNA1A	270	0.003	1.7	80.0	11	4.1

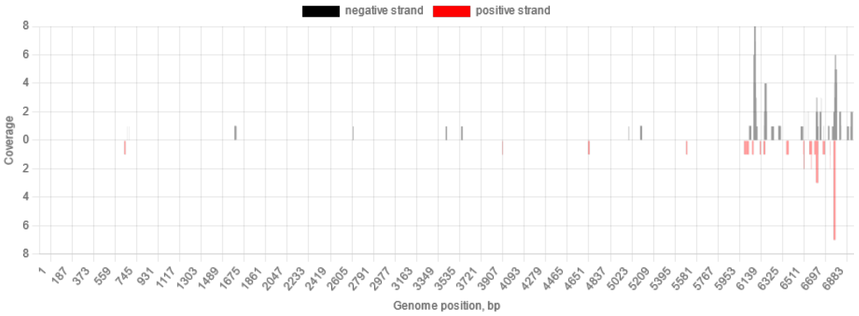

Mapping coverage of all reads matching SMoV_RNA1A (73 reads)

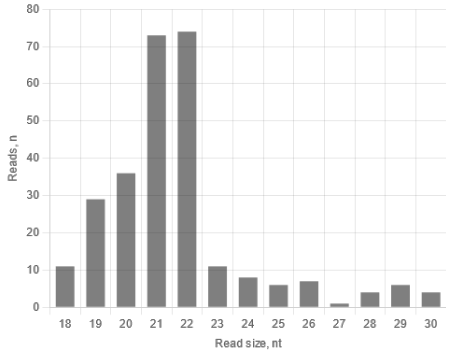

Length distribution of the reads

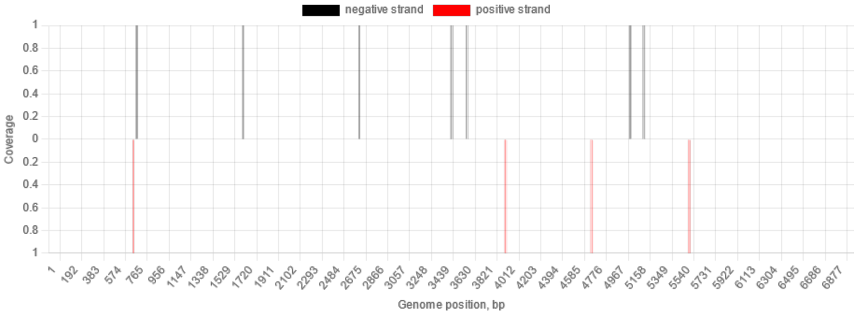

Mapping coverage of SMoV_RNA1A specific reads (11 reads)

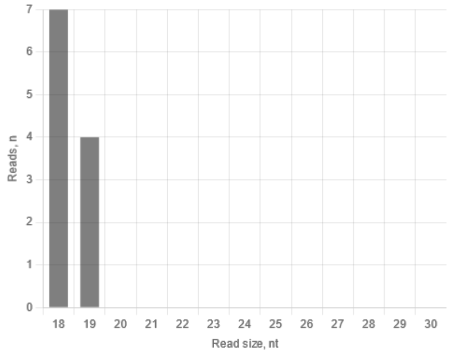

Length distribution of the reads

Dataset	Input reads	Virus	Reference	Mapped reads	Mapped reads, %	Forward/Reverse balance	Nonredundant, %	Strain specific	Strain specific, % from total mapped reads
S98	9901238	SMoV	SMoV_RNA1B	418	0.004	1.5	84.9	39	9.3

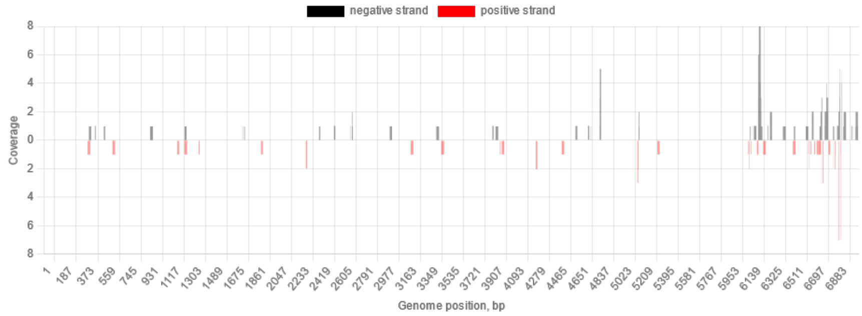

Mapping coverage of all reads matching SMoV_RNA1B (103 reads)

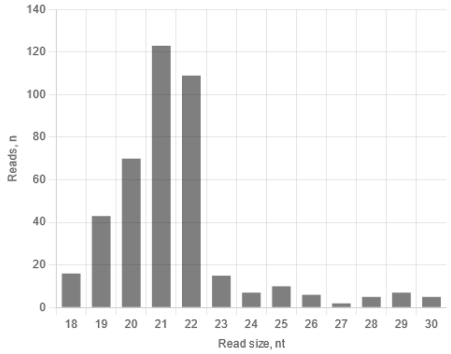

Length distribution of the reads

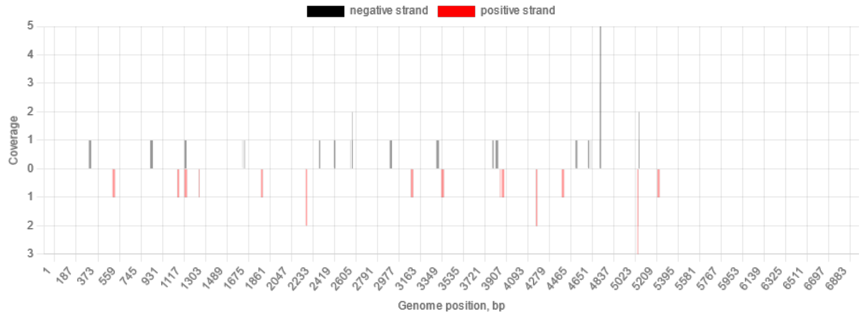

Mapping coverage of SMoV_RNA1B specific reads (39 reads)

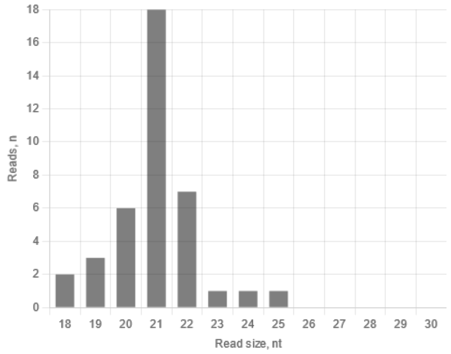

Length distribution of the reads

Dataset	Input reads	Virus	Reference	Mapped reads	Mapped reads, %	Forward/Reverse balance	Nonredundant, %	Strain specific	Strain specific, % from total mapped reads
S98	9901238	SMoV	SMoV_RNA1C	481	0.005	1.6	85.9	73	15.2

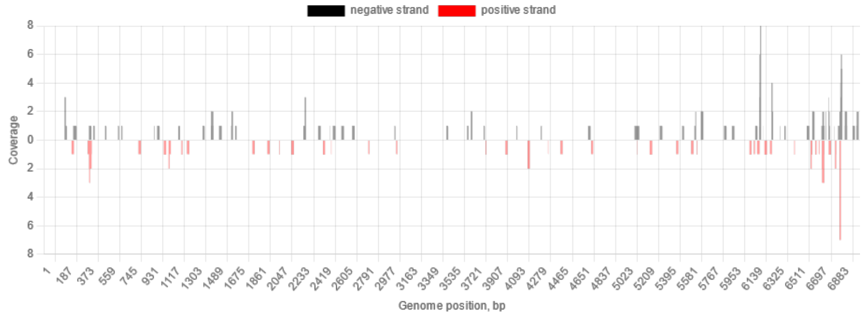

Mapping coverage of all reads matching SMoV_RNA1C (137 reads)

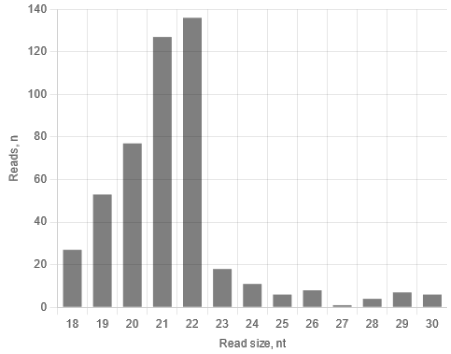

Length distribution of the reads

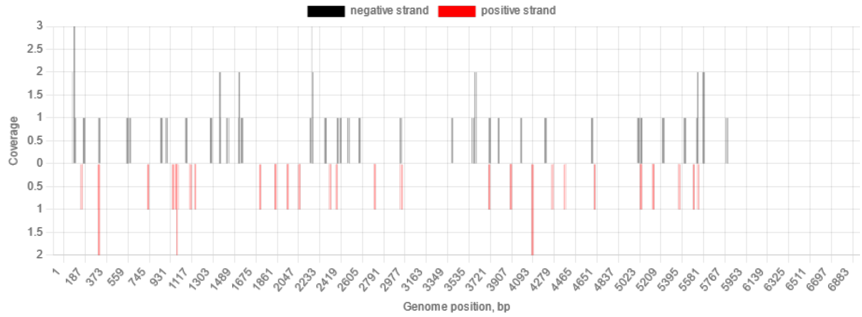

Mapping coverage of SMoV_RNA1C specific reads (73 reads)

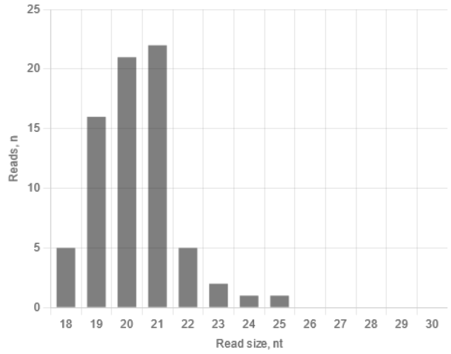

Length distribution of the reads

Dataset	Input reads	Virus	Reference	Mapped reads	Mapped reads, %	Forward/Reverse balance	Nonredundant, %	Strain specific	Strain specific, % from total mapped reads
S98	9901238	SMoV	SMoV_RNA2A	418	0.004	1.7	88.0	87	20.8

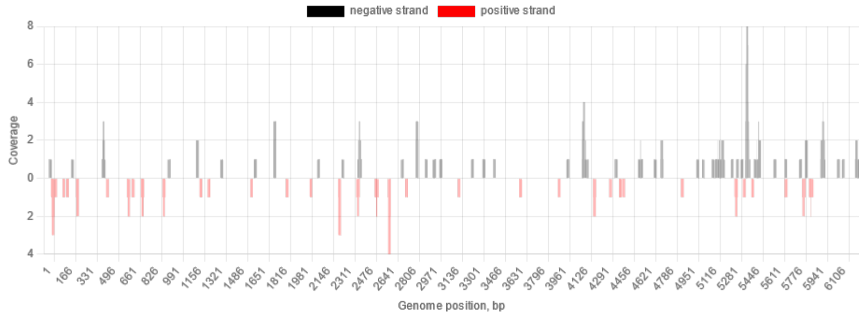

Mapping coverage of all reads matching SMoV_RNA2A (127 reads)

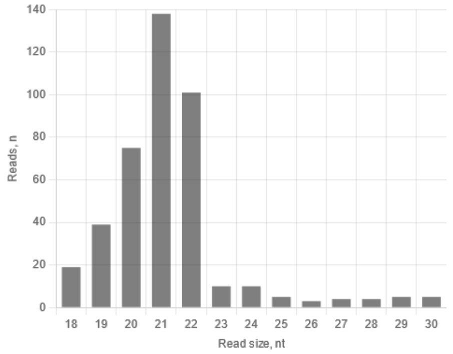

Length distribution of the reads

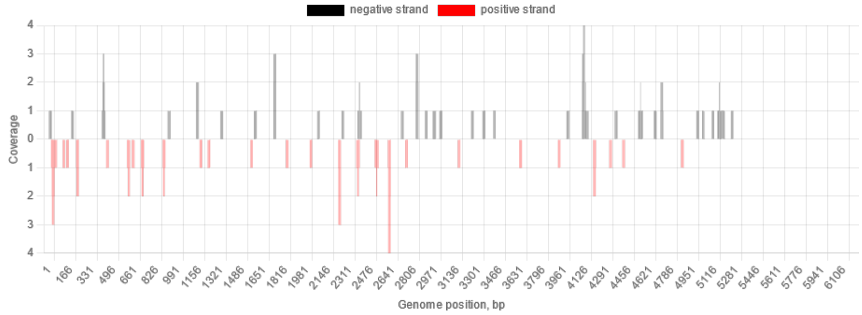

Mapping coverage of SMoV_RNA2A specific reads (87 reads)

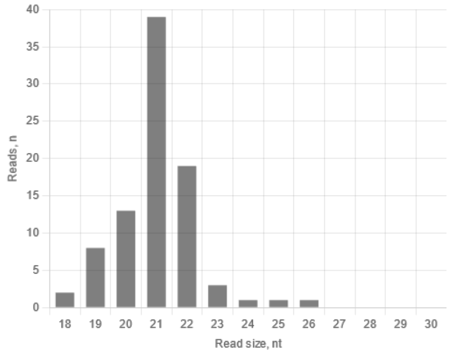

Length distribution of the reads

Dataset	Input reads	Virus	Reference	Mapped reads	Mapped reads, %	Forward/Reverse balance	Nonredundant, %	Strain specific	Strain specific, % from total mapped reads
S98	9901238	SMoV	SMoV_RNA2B	132	0.001	1.9	91.7	0	0.0

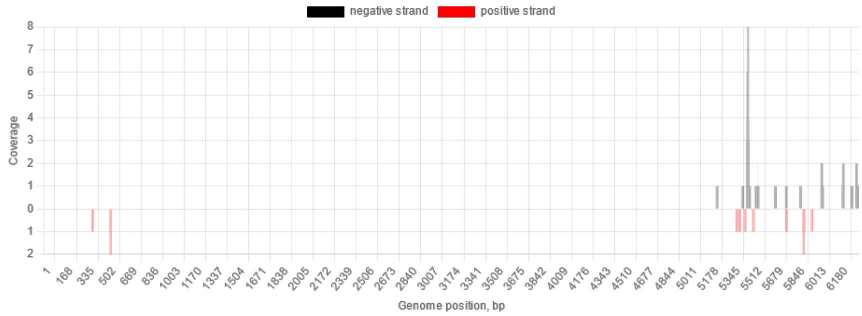

Mapping coverage of all reads matching SMoV_RNA2B (33 reads)

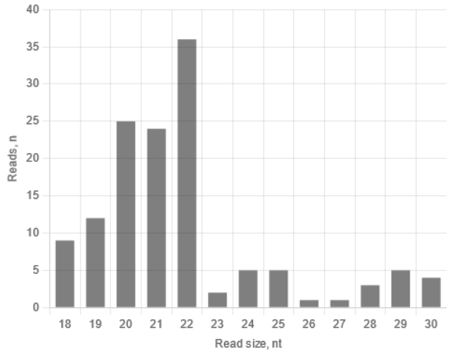

Length distribution of the reads

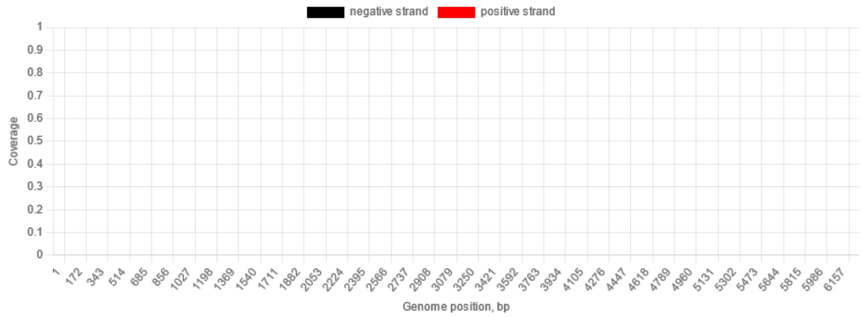

Mapping coverage of SMoV_RNA2B specific reads (0 reads)

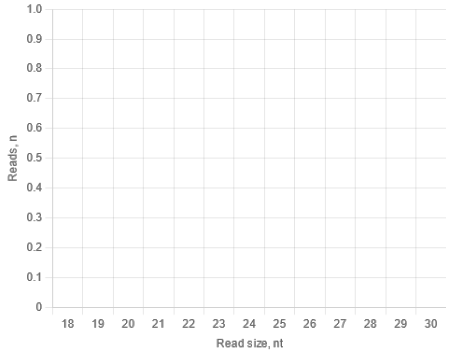

Length distribution of the reads

Dataset	Input reads	Virus	Reference	Mapped reads	Mapped reads, %	Forward/Reverse balance	Nonredundant, %	Strain specific	Strain specific, % from total mapped reads
S98	9901238	SMoV	SMoV_RNA2C	537	0.005	1.7	84.4	76	14.2

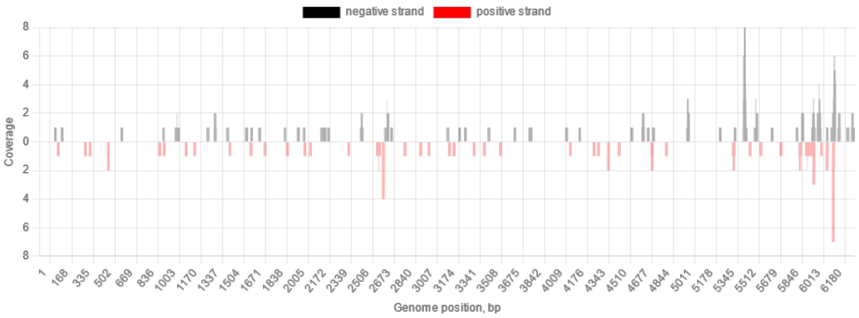

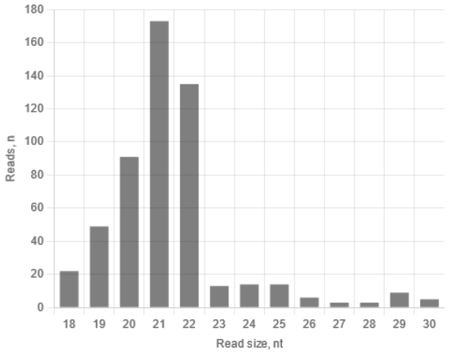

Mapping coverage of all reads matching SMoV_RNA2C (140reads)

Length distribution of the reads

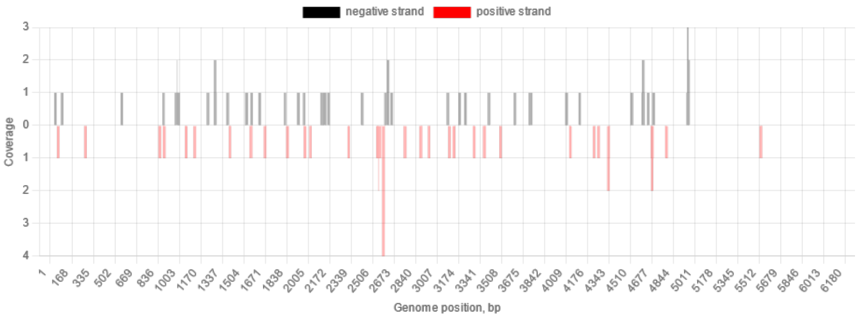

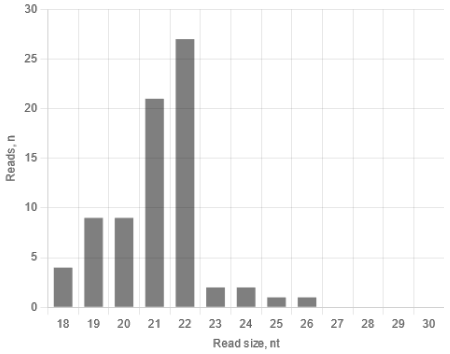

Mapping coverage of SMoV_RNA2C specific reads (76 reads)

Length distribution of the reads

Dataset	Input reads	Virus	Reference	Mapped reads	Mapped reads, %	Forward/Reverse balance	Nonredundant, %	Strain specific	Strain specific, % from total mapped reads
S98	9901238	StrV1	StrV1_A	15033	0.152	0.9	43.8	11985	79.7

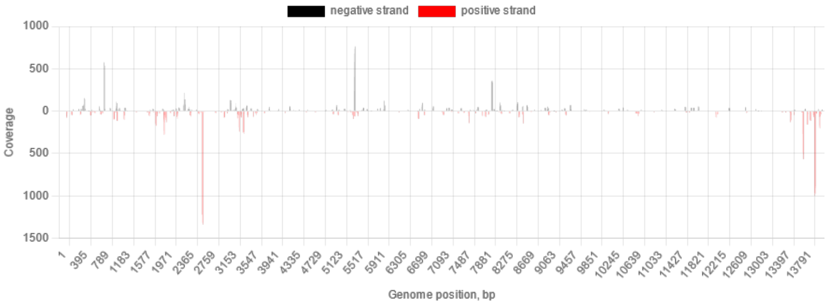

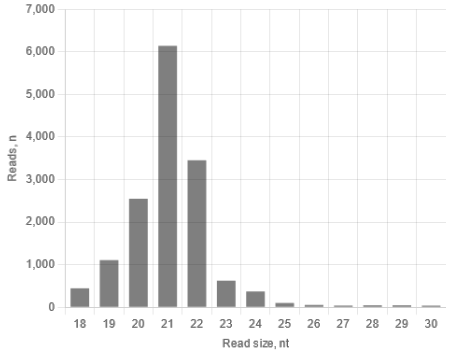

Mapping coverage of all reads matching StrV1_A (13594reads)

Length distribution of the reads

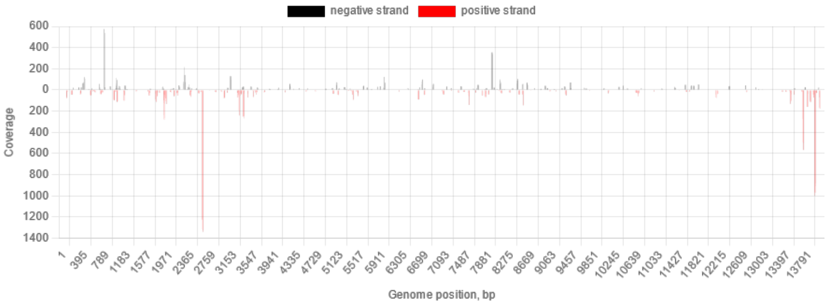

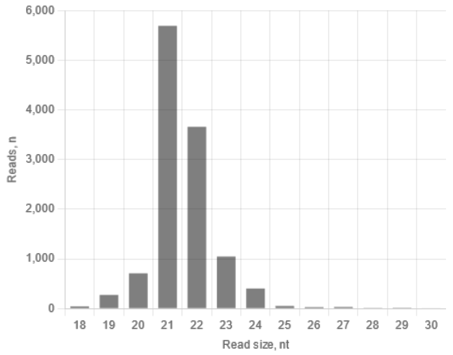

Mapping coverage of StrV1_A specific reads (11985reads)

Length distribution of the reads

Dataset	Input reads	Virus	Reference	Mapped reads	Mapped reads, %	Forward/Reverse balance	Nonredundant, %	Strain specific	Strain specific, % from total mapped reads
S98	9901238	StrV1	StrV1_B	470	0.005	0.9	48.7	13356	2841.7

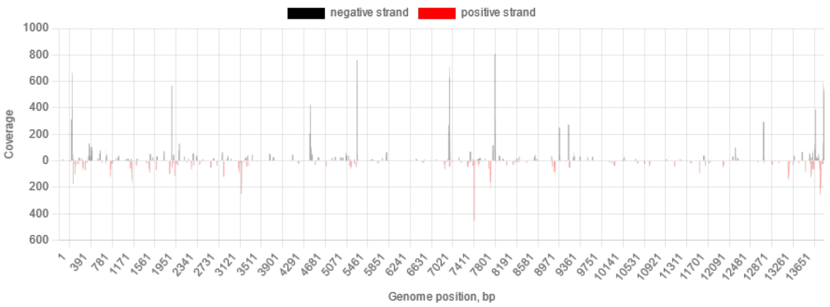

Mapping coverage of all reads matching StrV1_B (14411reads)

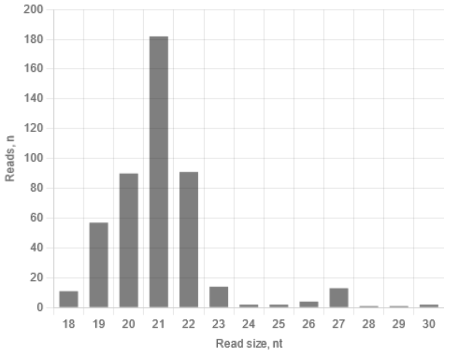

Length distribution of the reads

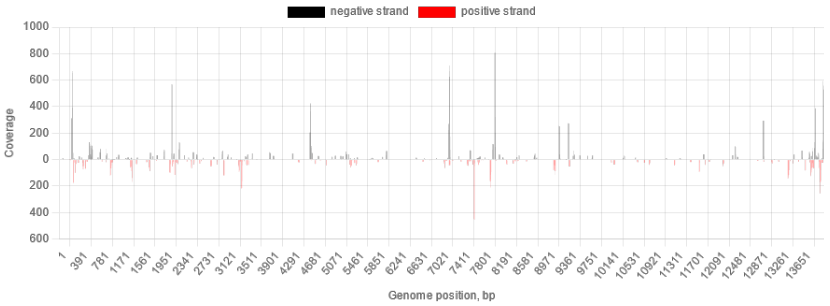

Mapping coverage of StrV1_B specific reads (13356reads)

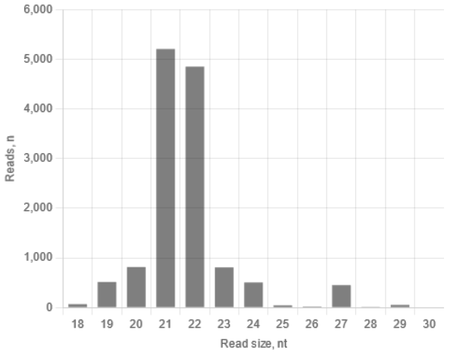

Length distribution of the reads

Dataset	Input reads	Virus	Reference	Mapped reads	Mapped reads, %	Forward/Reverse balance	Nonredundant, %	Strain specific	Strain specific, % from total mapped reads
S98	9901238	StrV1	StrV1_C	1986	0.020	1.0	36.0	60	3.0

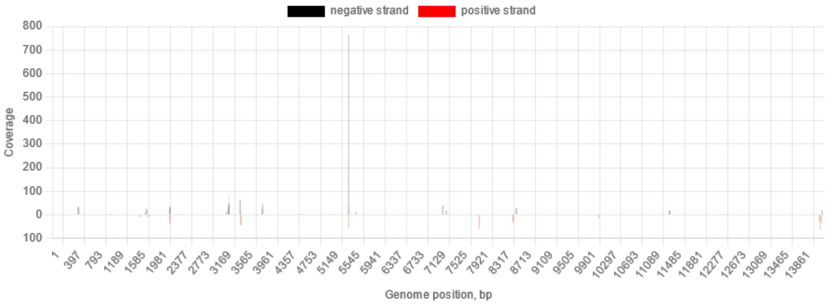

Mapping coverage of all reads matching StrV1_C (1549reads)

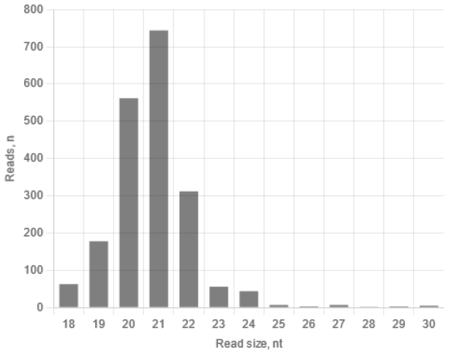

Length distribution of the reads

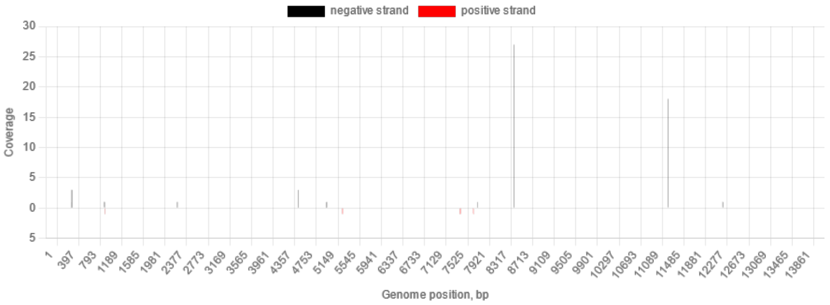

Mapping coverage of StrV1_C specific reads (60 reads)

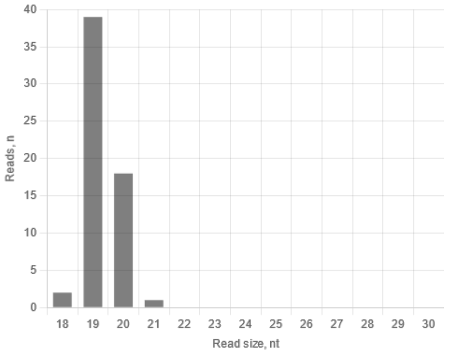

Length distribution of the reads

Mappings of the S99 reads against the viral references.

Dataset	Input reads	Virus	Reference	Mapped reads	Mapped reads, %	Forward/Reverse balance	Nonredundant, %	Strain specific	Strain specific, % from total mapped reads
S99	6772412	SMoV	SMoV_RNA1A	132	0.002	1.5	85.6	11	8.3

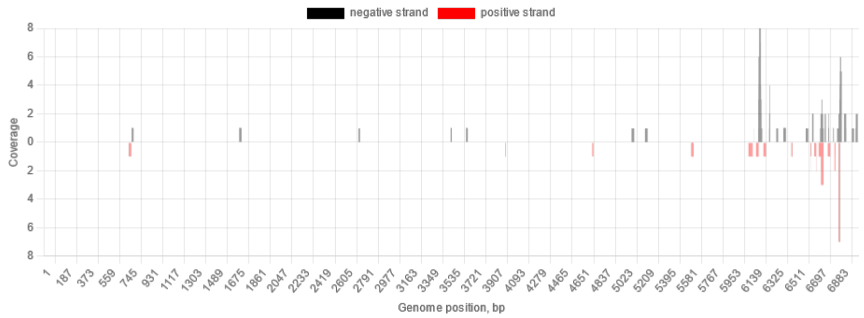

Mapping coverage of all reads matching SMoV_RNA1A (73 reads)

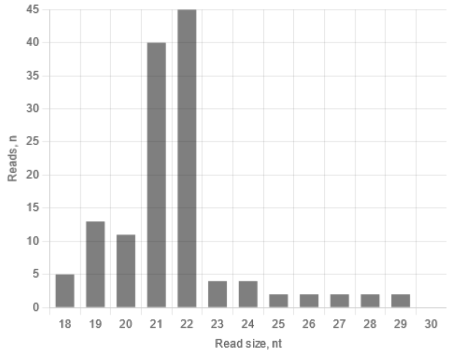

Length distribution of the reads

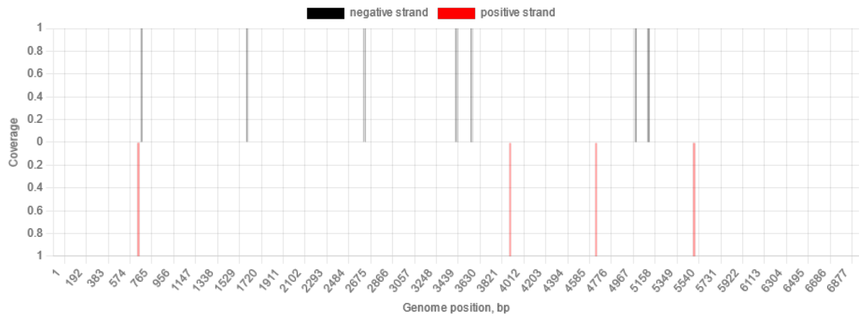

Mapping coverage of SMoV_RNA1A specific reads (11 reads)

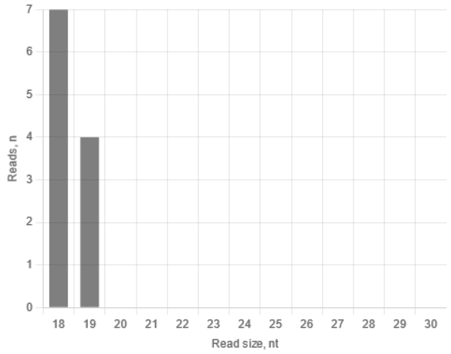

Length distribution of the reads

Dataset	Input reads	Virus	Reference	Mapped reads	Mapped reads, %	Forward/Reverse balance	Nonredundant, %	Strain specific	Strain specific, % from total mapped reads
S99	6772412	SMoV	SMoV_RNA1B	176	0.003	1.5	89.8	39	22.2

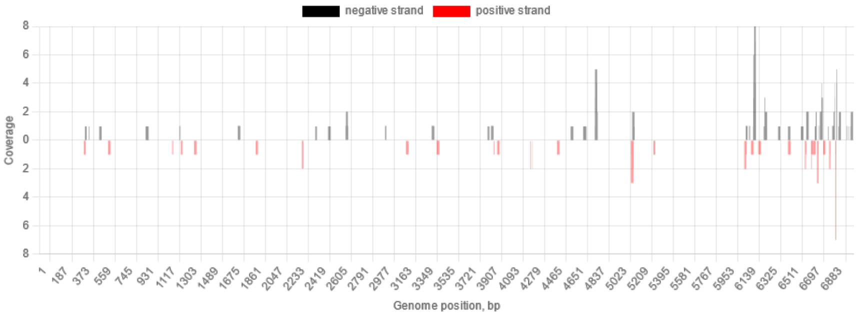

Mapping coverage of all reads matching SMoV_RNA1B (103 reads)

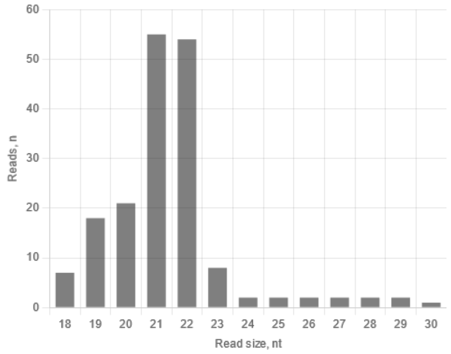

Length distribution of the reads

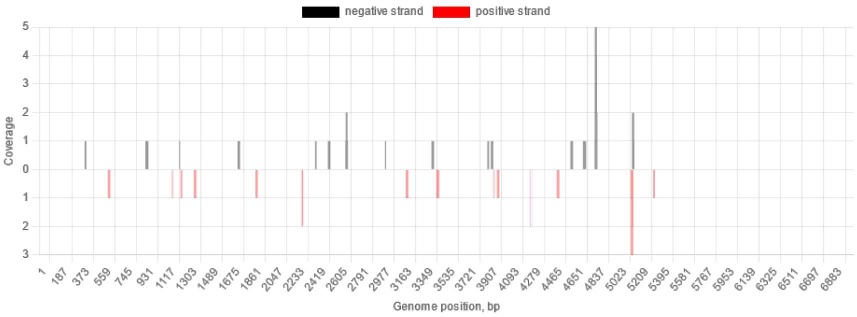

Mapping coverage of SMoV_RNA1B specific reads (39 reads)

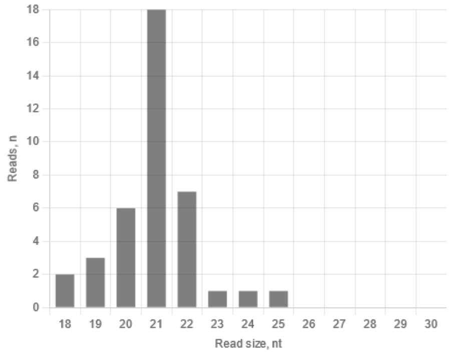

Length distribution of the reads

Dataset	Input reads	Virus	Reference	Mapped reads	Mapped reads, %	Forward/Reverse balance	Nonredundant, %	Strain specific	Strain specific, % from total mapped reads
S99	6772412	SMoV	SMoV_RNA1C	246	0.004	1.5	88.2	73	29.7

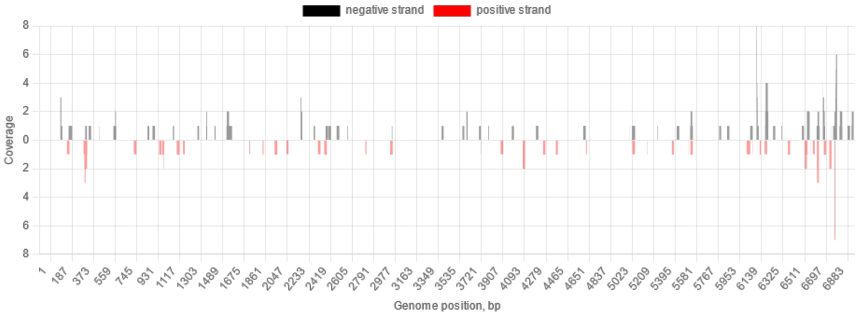

Mapping coverage of all reads matching SMoV_RNA1C (137 reads)

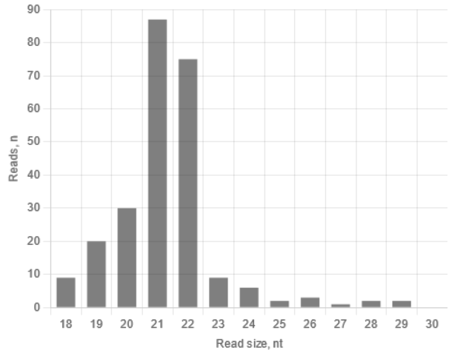

Length distribution of the reads

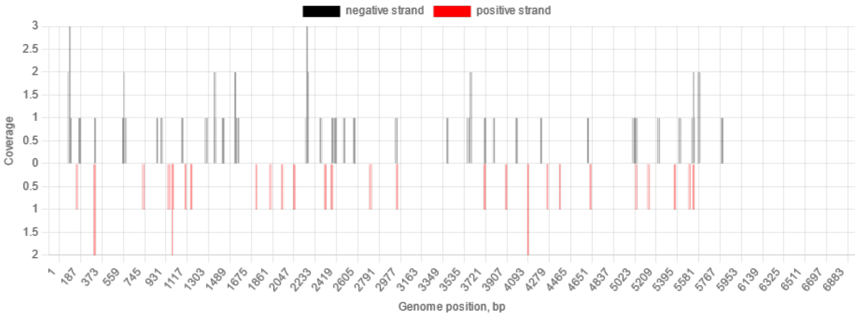

Mapping coverage of SMoV_RNA1C specific reads (73 reads)

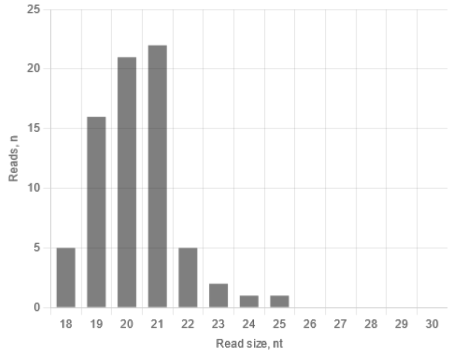

Length distribution of the reads

Dataset	Input reads	Virus	Reference	Mapped reads	Mapped reads, %	Forward/Reverse balance	Nonredundant, %	Strain specific	Strain specific, % from total mapped reads
S99	6772412	SMoV	SMoV_RNA2A	190	0.003	1.7	90.0	87	45.8

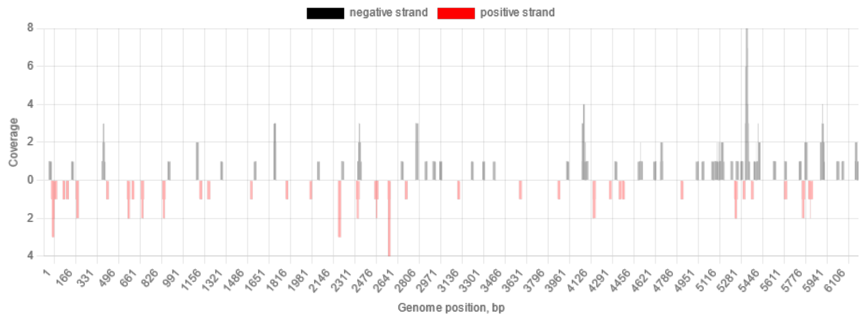

Mapping coverage of all reads matching SMoV_RNA2A (127 reads)

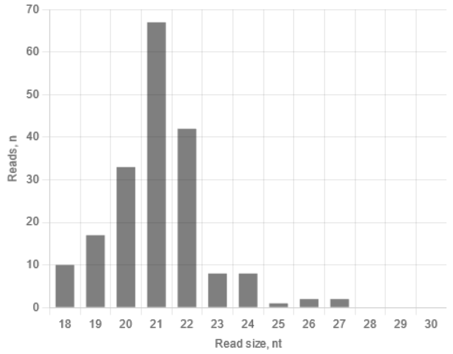

Length distribution of the reads

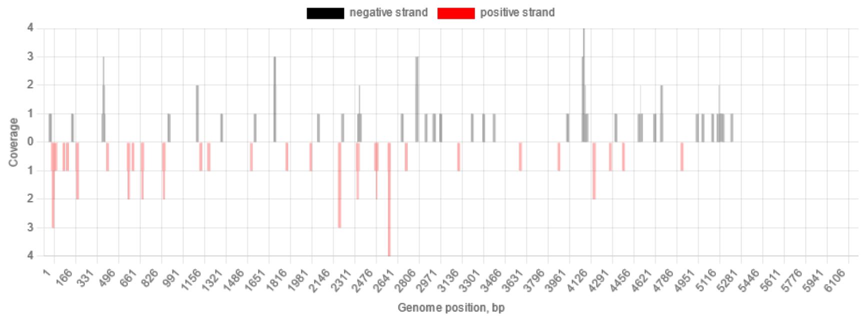

Mapping coverage of SMoV_RNA2A specific reads (87 reads)

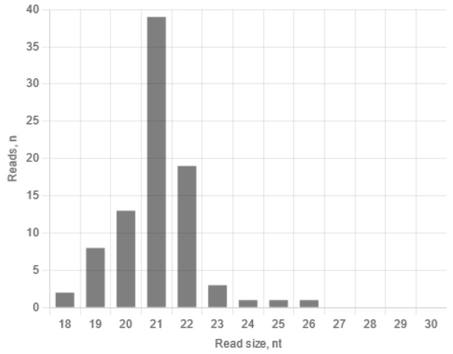

Length distribution of the reads

Dataset	Input reads	Virus	Reference	Mapped reads	Mapped reads, %	Forward/Reverse balance	Nonredundant, %	Strain specific	Strain specific, % from total mapped reads
S99	6772412	SMoV	SMoV_RNA2B	56	0.001	2.3	94.6	0	0.0

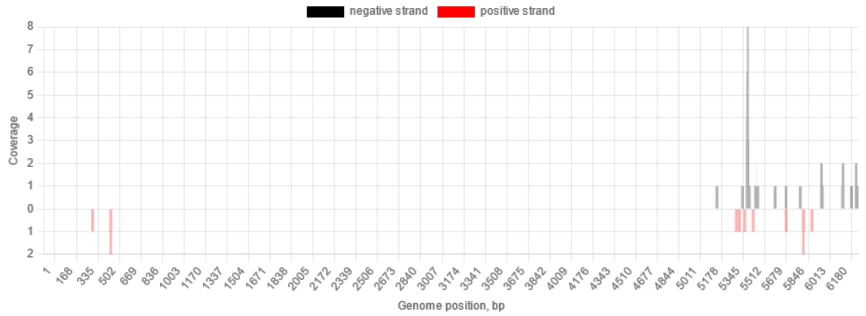

Mapping coverage of all reads matching SMoV_RNA2B (33 reads)

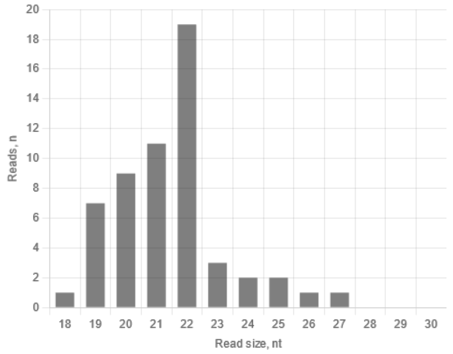

Length distribution of the reads

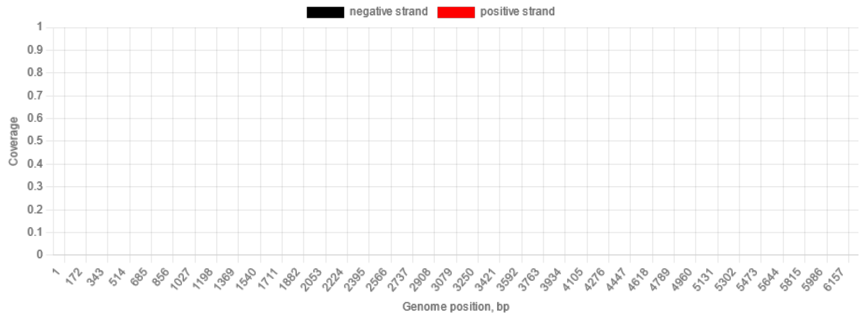

Mapping coverage of SMoV_RNA2B specific reads (0 reads)

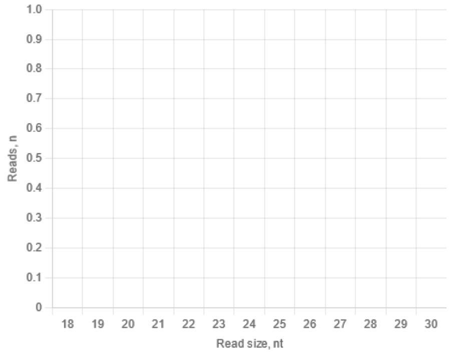

Length distribution of the reads

Dataset	Input reads	Virus	Reference	Mapped reads	Mapped reads, %	Forward/Reverse balance	Nonredundant, %	Strain specific	Strain specific, % from total mapped reads
S99	6772412	SMoV	SMoV_RNA2C	236	0.003	1.4	85.6	76	32.2

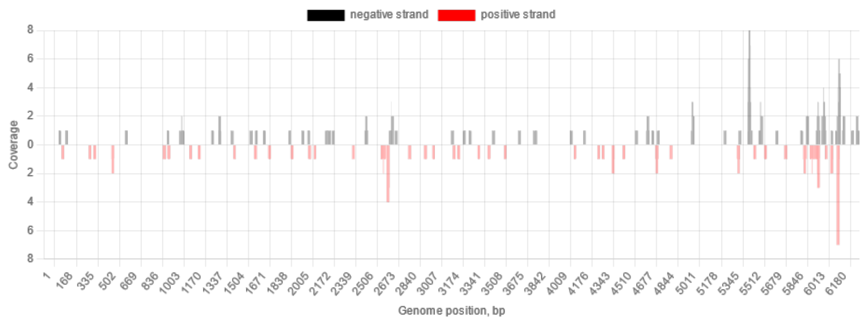

Mapping coverage of all reads matching SMoV_RNA2C (140reads)

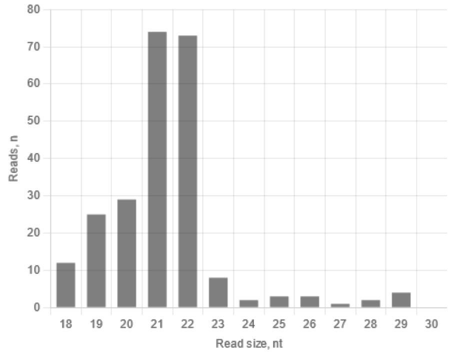

Length distribution of the reads

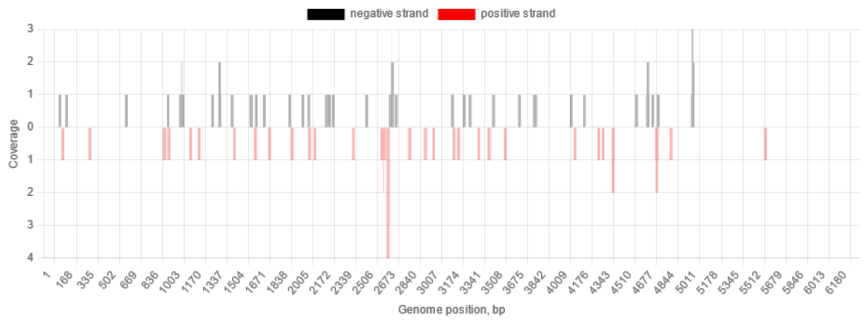

Mapping coverage of SMoV_RNA2C specific reads (76 reads)

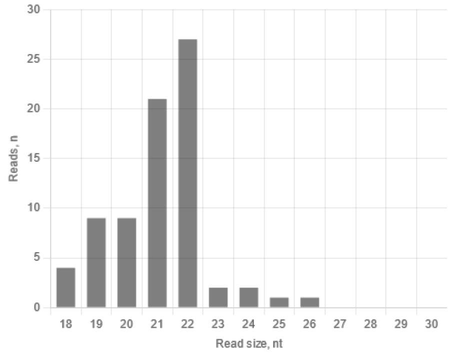

Length distribution of the reads

Dataset	Input reads	Virus	Reference	Mapped reads	Mapped reads, %	Forward/Reverse balance	Nonredundant, %	Strain specific	Strain specific, % from total mapped reads
S99	6772412	StrV1	StrV1_A	268	0.004	0.5	60.4	11985	4472.0

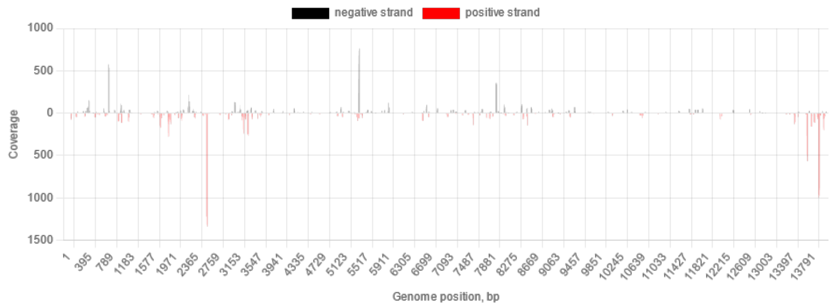

Mapping coverage of all reads matching StrV1_A (13594reads)

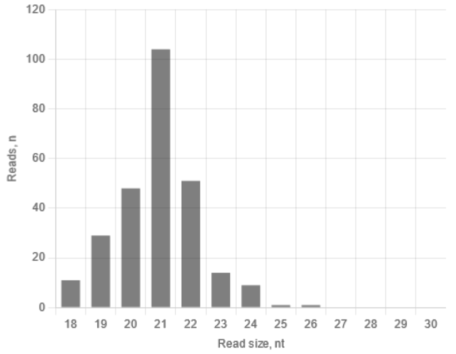

Length distribution of the reads

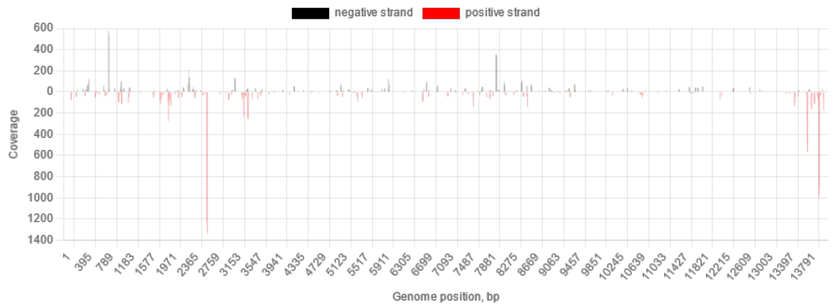

Mapping coverage of StrV1_A specific reads (11985reads)

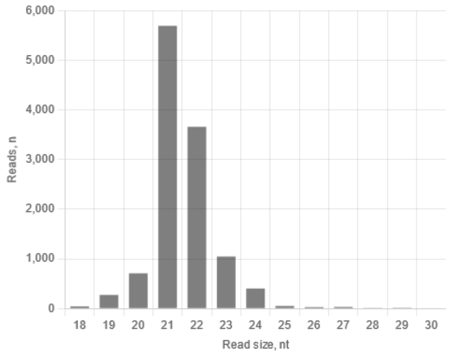

Length distribution of the reads

Dataset	Input reads	Virus	Reference	Mapped reads	Mapped reads, %	Forward/Reverse balance	Nonredundant, %	Strain specific	Strain specific, % from total mapped reads
S99	6772412	StrV1	StrV1_B	8361	0.123	1.2	45.6	13356	159.7

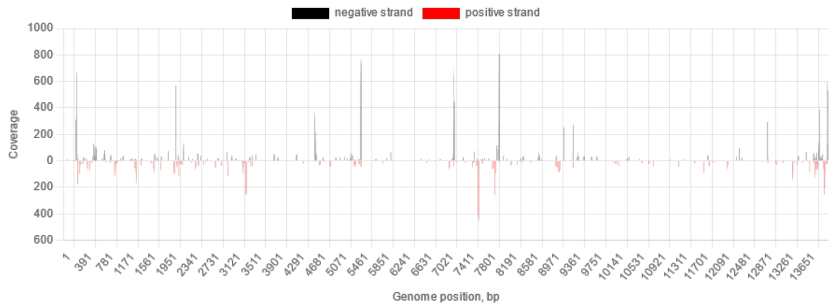

Mapping coverage of all reads matching StrV1_B (14411reads)

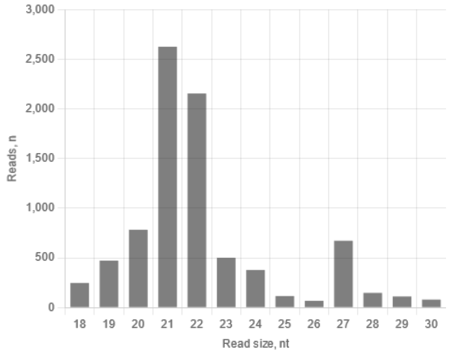

Length distribution of the reads

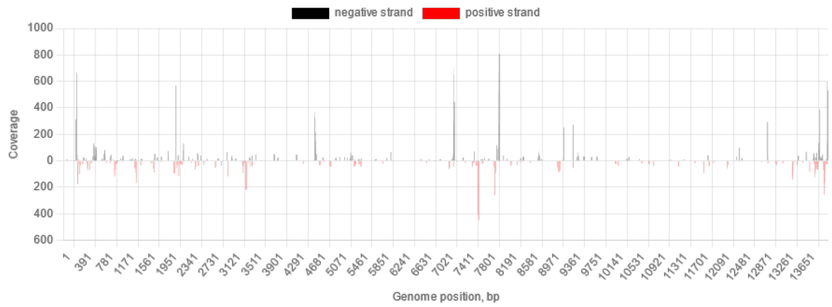

Mapping coverage of StrV1_B specific reads (13356reads)

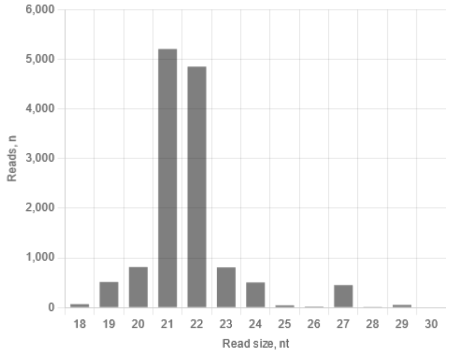

Length distribution of the reads

Dataset	Input reads	Virus	Reference	Mapped reads	Mapped reads, %	Forward/Reverse balance	Nonredundant, %	Strain specific	Strain specific, % from total mapped reads
S99	6772412	StrV1	StrV1_C	150	0.002	0.5	54.0	60	40.0

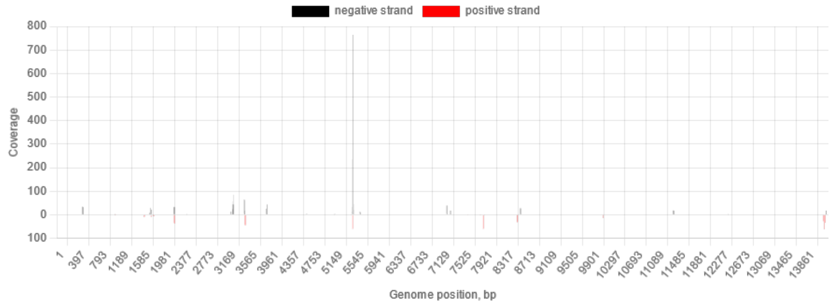

Mapping coverage of all reads matching StrV1_C (1549reads)

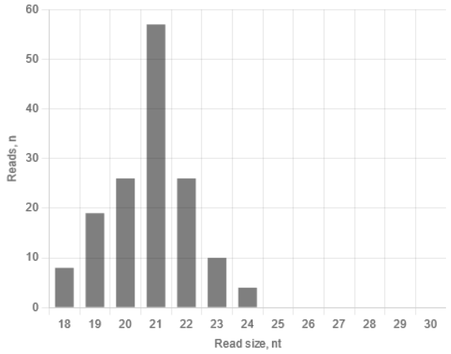

Length distribution of the reads

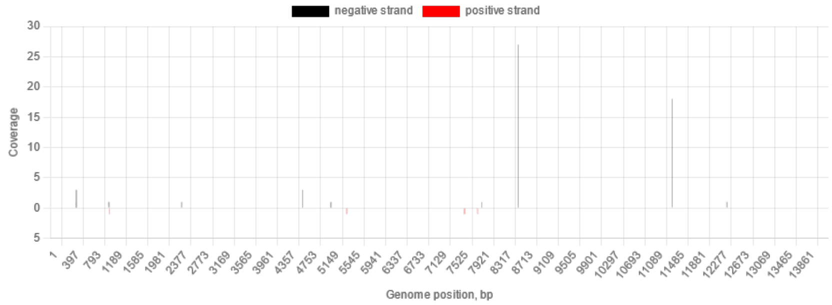

Mapping coverage of StrV1_C specific reads (60 reads)

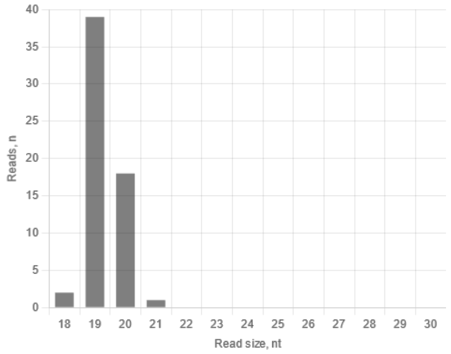

Length distribution of the reads
